# Environmental Risk Factors, Protective Factors, and Biomarkers for Allergic Rhinitis: A Systematic Umbrella Review of the Evidence

**DOI:** 10.1007/s12016-023-08964-2

**Published:** 2023-07-25

**Authors:** Xianpeng Xu, Xinghong Liu, Jiongke Li, Xinxing Deng, Tianrong Dai, Qingjie Ji, Dajing Xiong, Hui Xie

**Affiliations:** 1 Department of Otorhinolaryngology, Chengdu University of Traditional Chinese Medicine, Chengdu, 610075 China; 2 Department of Otorhinolaryngology, Hospital of Chengdu University of Traditional Chinese Medicine, Chengdu, 610072 China; 3 Department of Dermatology, Quzhou hospital of Traditional Chinese Medicine, 324000 Quzhou, China

**Keywords:** Allergic rhinitis, Environmental risk and protective factors, Biomarkers, Umbrella review

## Abstract

Many potential environmental risk factors, protective factors, and biomarkers of AR have been published, but so far, the strength and consistency of their evidence are unclear. We conducted a comprehensive review of environmental risk, protective factors, and biomarkers for AR to establish the evidence hierarchy. We systematically searched Embase, PubMed, Cochrane Library, and Web of Science electronic database from inception to December 31, 2022. We calculated summary effect estimate (odds ratio (OR), relative risk (RR), hazard ratio (HR), and standardized mean difference (SMD)), 95% confidence interval, random effects *p* value, *I*^2^ statistic, 95% prediction interval, small study effects, and excess significance biases, and stratification of the level of evidence. Methodological quality was assessed by AMSTAR 2 (A Measurement Tool to Assess Systematic Reviews 2). We retrieved 4478 articles, of which 43 met the inclusion criteria. The 43 eligible articles identified 31 potential environmental risk factors (10,806,206 total population, two study not reported), 11 potential environmental protective factors (823,883 total population), and 34 potential biomarkers (158,716 total population) for meta-analyses. The credibility of evidence was convincing (class I) for tic disorders (OR = 2.89, 95% CI 2.11–3.95); and highly suggestive (class II) for early-life antibiotic use (OR = 3.73, 95% CI 3.06–4.55), exposure to indoor dampness (OR = 1.49, 95% CI 1.27–1.75), acetaminophen exposure (OR = 1.54, 95% CI 1.41–1.69), childhood acid suppressant use (OR = 1.40, 95% CI 1.23–1.59), exposure to indoor mold (OR = 1.66, 95% CI 1.26–2.18), coronavirus disease 2019 (OR = 0.11, 95% CI 0.06–0.22), and prolonged breastfeeding (OR = 0.72, 95% CI 0.65–0.79). This study is registered in PROSPERO (CRD42022384320).

**Supplementary Information:**

The online version contains supplementary material available at 10.1007/s12016-023-08964-2.

## Introduction

Allergic rhinitis (AR) is a chronic inflammatory disease of the nasal mucosa, which is caused by the release of immunoglobulin E (IgE)-mediated mediators (mainly histamine) after allergic individuals are exposed to allergens, and a variety of immune active cells and cell factors are involved [1, 2]. In the USA, the prevalence of physician-diagnosed allergic rhinitis is about 15%, and patients self-report abnormal nasal symptoms up to 30% [3, 4]. A Danish study spanning three decades reported that the prevalence of allergic rhinitis in the Danish adult population in the last three decades increased from 16% in 1990–1991 to 29% in 2012–2015, and the prevalence of allergic rhinitis is likely to continue to increase in this century [5]. It has been estimated that 1 in 6 individuals in the USA may have AR, generating $2 billion to $5 billion in direct medical expenditures annually and up to $2 billion to $4 billion in lost productivity due to lost work and attendance [6]. Nasal symptoms, including nasal congestion, itchy nose, rhinorrhea (runny nose), and sneezing, are the signature symptoms of AR, and symptoms last or accumulate for more than 1 h per day [7]. This long-term chronic nasal symptom may further lead to the decline of brain function, affect the quality of life of patients, reduce the efficiency of work and study in adults and children, and also lead to the patient’s irritability, and even cause anxiety, depression, and other neuropsychiatric symptoms [8]. It is estimated that about 40% of AR patients will be accompanied by bronchial asthma, and about 30–71% of AR patients will have allergic conjunctivitis or conjunctival symptoms [9, 10].

Given that AR is caused by the introduction of allergens in sensitized individuals, and the immune and inflammatory response induced by the excessive production of IgE in response to environmental allergens in atopic individuals [11], how to better identify and understand the risk factors of the disease and prevent the occurrence of the disease through the identification and intervention of risk factors has become the key to the implementation of effective prevention and control measures [12]. The pathogenesis and etiology of allergic rhinitis are not completely clear, and can not be explained simply by some susceptible changes in human genes, which may be related to multiple factors such as genetics, climate, living habits, eating habits, and concomitant diseases [13]. AR is a disease with complex physiopathology and is thought to be caused by the interaction of more than 100 genetic loci with complex environmental factors, which reflects the association between many genetic and environmental factors and AR [14, 15]. Although there is growing evidence that environmental exposures, climate change, and lifestyle are important risk factors for AR [16], disease-related biomarkers and environmental factors remain challenging in the early diagnosis, therapeutic interventions, and potential pathogenesis of AR [17, 18]. Many meta-analyses and systematic reviews have elaborated, analyzed, and summarized AR risk factors and biomarkers in their respective fields, and reported the final results. However, the thematic analysis of these meta-analyses is often single, and meta-analyses estimates of mean effect sizes may be imprecise, which may overestimate effect sizes, especially if the meta-analysis is based on only a few studies, and the size of observed effects, especially in meta-analyses with limited evidence, is often exaggerated [19, 20]. Therefore, to avoid these shortcomings as much as possible, we comprehensively collected and evaluated meta-analyses and systematic reviews of AR-related environmental factors and biomarkers, and stratified the levels of evidence with a view to examining and confirming the realistic relationship between environmental factors and biomarkers and AR.

## Methods

We followed the latest PRISMA (Preferred Reporting Items for Systematic reviews and Meta-Analyses) [21] guidelines (Appendix p3–5) and conducted a comprehensive integration of currently published meta-analyses and systematic reviews of environmental risk factors and biomarkers associated with AR. Two researchers (XPX, XHL) independently screened the literature, extracted the data, evaluated the quality, and cross-checked the literature. In case of disagreement, a third person (HX) was consulted to assist in the judgment. This study has been registered with PROSPERO, registration number CRD42022384320.

### Search Strategy and Eligibility Criteria

We used computer to search Embase, PubMed, Cochrane Library, and Web of Science electronic database from inception to 31 December 2022, without any language restrictions. We used a combination of subject terms and free words for the search, with search terms including “allergic rhinitis”, “meta-analysis”, and “systematic review”. The search strategy was adapted to the different databases, and the full search strategy and search terms for each database are presented in the Appendix (p6). In addition, we manually traced the references of the included literature to supplement access to relevant studies.

The types of articles we included were systematic reviews and meta-analyses, which mainly studied the association of environmental factors or biomarkers with AR. The types of design included in the original study were limited to Cohort studies (cross-sectional and longitudinal) and case–control studies. Environmental factors (including risk and protective factors) and biomarkers (Appendix p7) are defined according to World Health Organization (WHO) [22, 23], and allergic rhinitis is defined according to clinical practice guideline [6].

We excluded articles that were not related to the research topic, which was not related to environmental factors, biomarkers and AR and their associations with each other. We excluded systematic reviews that did not perform a meta-analysis as well as meta-analyses that did not provide sufficient data. In addition, for systematic reviews and meta-analyses lacking sufficient data, we attempted to contact the corresponding authors to obtain the necessary data for re-analysis. We also excluded animal studies, duplicate publications, conference abstracts, protocols, posters, and letters. If two or more studies were on the same topic or assessed the same exposure and outcome measures, we considered these studies on the same topic to have the possibility of overlap, which could lead to bias of publication. Therefore, first, we prioritize meta-analyses with adjusted study estimates over those with crude estimates; next, we select meta-analyses with higher AMSTAR 2 (A Measurement Tool to Assess Systematic Reviews 2) [24] scores; and finally, we select meta-analyses with a higher number of included studies. Finally, we excluded some studies whose causal relationships were contrary to our topic, for example, a meta-analysis [25] investigating the risk of systemic lupus erythematosus (SLE) in patients with allergic rhinitis (AR), which was to analyze whether AR was a risk factor for SLE, which happened to be contrary to the topic of our study. The Appendix (p8–p12) provides a list of all potentially relevant studies that were read in full and explains the reasons for the exclusion of each article from the systematic review.

### Literature Screening and Data Extraction

Two researchers (XPX, XHL) independently screened the literature according to the inclusion and exclusion criteria, extracted data, and evaluated the evidence quality of the included studies. Firstly, the title and abstract of the articles were read to exclude obviously unqualified articles, and then, the full text of the articles after initial screening was further read to determine whether they were included. Standard data extraction tables were used to extract information.

The extracted contents included (1) basic information, including the first author, the year of publication, the number of included studies, and the study design; (2) baseline information of included studies, including the number of cases, total sample size, environmental risk factors, protective factors or biomarkers; (3) the results of meta-analysis, including the heterogeneity evaluation index, the combined effect size and confidence interval of the primary outcome; (4) methodological quality assessment methods and tools for original studies or evaluation methods for the quality of reported evidence.

### Data Analysis

We followed the previous umbrella review design and validated statistical methods in order to address the issues we encountered in our pooled summaries [26, 27]. Furthermore, we also refer to the metaumbrella application (https://www.metaumbrella.org/app) designed by Dr. Corentin J Gosling and Dr. Aleix Solanes et al., which automatically performs umbrella reviews (including, but not limited to, meta-analyses), layering evidence according to various criteria, and generating visual representations of the results [28].

We conducted a comprehensive integrated analysis of the meta-analyses that met the inclusion criteria, and extracted basic data information and study estimates. We selected random-effect models to incorporate pooled effect estimates, 95% confidence intervals, and *p* values [29, 30]. The DerSimonian and Laird (DL) model was selected for the random effects meta-analysis, which requires a simple summary of data from each study and is particularly suitable for providing estimates of population effects and describing the heterogeneity of effects across a range of studies [31]. To confirm the confidence of the evidence, we also calculated whether *p* value < 0.001 or < 0.000001 [32, 33]. Before combining the effect values, in order to clarify whether heterogeneity really exists between studies, we used the *Q* test, a common test for heterogeneity in meta-analysis, and calculated the statistic *I*^2^ [34]. Prediction is one of the most important results of meta-analysis, providing a convenient format for expressing the full range of uncertainty around inferences and for making inferences about studies not included in the meta-analysis; therefore, we estimated the 95% prediction interval, the range in which we expect the effect of association would lie for 95% of future studies [35]. We assessed the presence of small study effects using a simplified inverted funnel plot test developed by Egger and Colleagues [36]. We assessed the presence of small-study effects using a simple inverted funnel plot test developed by Egger and Colleagues that uses linear regression to measure the symmetry of the inverted funnel plot based on the natural logarithmic value of the ratio; if there is asymmetry, small-sample trials show effects that systematically deviate from those of larger samples, and the regression line does not pass the starting point (Egger *p* value < 0.1 for a small study effect was found). Selective analysis and reporting of selective results may lead to potential excess significance bias, which means that the number of statistically significant studies is suspicious to be too high. For this reason, Ioannidis’ test was used to evaluate whether there was a significant bias in the meta-analyses (*p* value < 0.05) [37]. We performed a subgroup analysis of children with identified AR symptoms, excluding adults older than 18 years of age, which further assessed the robustness of the evidence. Forest plots and funnel plots and their data processing were performed using R version 4.2.2. and its software package.

### Methodological Quality and Evidence Credibility

Methodological quality was assessed using AMSTAR 2 [24], and the included studies were evaluated on an item-by-item basis with “partially yes,” “yes,” and “no” according to the scale entries, and the results were finally summed for each component.

According to previously published umbrella reviews and the metaumbrella application [26, 27, 38], we graded the evidence derived from the studies against the evidence grading criteria, which are generally based on the following criteria: convincing (class I), highly suggestive (class II), suggestive (class III), weak (class IV), and not significant (NS). The criteria for grading evidence included: random effects *p* value, number of AR cases, *p* value of the largest study, heterogeneity (*I*^2^), small study effects, excess significance bias, 95% prediction interval, and 95% prediction interval of the largest study. However, since some of the variables in these criteria are continuous variables, there is an artificial demarcation of critical points. For example, a study involving 1001 patients with a highly significant *p* values < 0.000001 association between risk factors and outcomes may be classified as class I evidence according to the grading criteria of evidence, while another study with the same results in other aspects but only 1000 patients may be graded as class IV evidence. This may not be common, but researchers must be careful about the differences. Table 1 provides detailed grading criteria for evidence.

**Table 1:** Level of evidence for grading levels

	Convincing (class I)	Highly suggestive (class II)	Suggestive (class III)	Weak (class IV)	Not significant (NS)
Random effects *p* value	< 0·000001	< 0·000001	< 0·001	< 0·05	> 0·05
Number of AR cases	> 1000	> 1000	> 1000	** × **	** × **
The p value of the largest study	< 0.05	< 0.05	** × **	** × **	** × **
Heterogeneity (*I*^2^)	< 50%	** × **	** × **	** × **	** × **
Small study effects	Not detected	** × **	** × **	** × **	** × **
Excess significance bias	Not detected	** × **	** × **	** × **	** × **
95% prediction interval	Excludes the null	** × **	** × **	** × **	** × **
95% prediction interval of the largest study	Excludes the null	** × **	** × **	** × **	** × **

## Results

### Literature Search Results

**Fig. 1:**
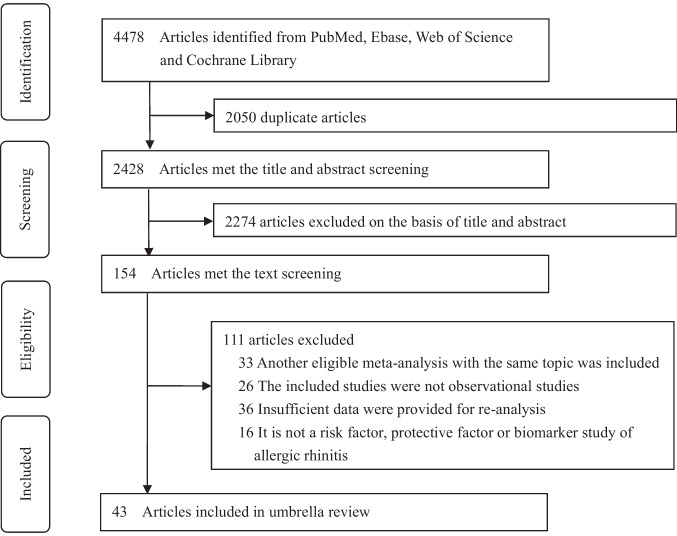
Flowchart of literature screening

We comprehensively searched four databases from inception to 31 December 2022 and retrieved a total of 4478 articles, 43 of which met the criteria by title, abstract, and full-text screening (Fig. 1). The 43 eligible articles (31 potential environmental factors articles and 11 potential biomarkers articles) identified 43 potential environmental factors (Table 2; Appendix p23–p68) and 34 potential biomarkers (Appendix p18–p20, p69–p103) for meta-analyses.

**Table 2:** Potential environmental risk and protective factors of AR, with details of statistical test results

Exposure	Source	Number of cases/total population	Number of study	Study design	Effect metrics	Random effects summary estimate (95% CI)	Random effects *p* value	*I*^2^	95% prediction interval	Egger *p* value	Large heterogeneity, small study effect, excess significance bias, 95% prediction interval excludes the null	AMSTAR 2
Convincing (class I)												
Tic disorders	Huang 2022 [39]	> 1000/56,696	5	Case–control	OR	2.89 (2.11–3.95)	1.64e-93	38%	(2.4–3.31)	0.82	None	Critically low
Highly suggestive (class II)												
Early-life antibiotic use	Liu 2022 [40]	> 1000/1,768,874	15	Cohort, cross-sectional	OR	3.73 (3.06–4.55)	5.06e-103	96%	(2.45–5.85)	0.02	Large heterogeneity, small study effect	Critically low
Exposure to indoor dampness	Jaakkola 2013 [41]	> 1000/28,904	7	Cohort, cross-sectional	OR	1.49 (1.27–1.75)	7.88e-08	93%	(0.95–2.23)	0.49	Large heterogeneity, excess significance bias, 95% prediction interval includes the null value	Low
Prolonged breastfeeding	Hoang 2022 [42]	> 1000/161,611	20	Cohort, cross-sectional	OR	0.72 (0.65–0.79)	2.32e-12	72%	(0.51–1.01)	0.06	Large heterogeneity, small study effect, excess significance bias, 95% prediction interval includes the null value	Moderate
Coronavirus disease 2019	Xu 2022 [43]	> 1000/294,622	8	Cohort, cross-sectional	OR	0.11 (0.06–0.22)	6.29e-15	99%	(0.02–0.84)	0.03	Large heterogeneity, small study effect	Critically low
Acetaminophen exposure	Zeng 2020 [44]	> 1000/870,492	18	Cohort, case–control, cross-sectional	OR	1.54 (1.41–1.69)	2.87e-14	95%	(0.89–2.67)	0.40	Large heterogeneity, excess significance bias, 95% prediction interval excludes the null value	Critically low
Childhood acid suppressant use	Muhammad 2023 [45]	> 1000/950,640	2	Cohort	HR	1.40 (1.23–1.59)	9.01e-08	97%	NA	NA	Large heterogeneity	Low
Exposure to indoor mold	Jaakkola 2013 [41]	> 1000/45,802	12	Cohort, cross-sectional	OR	1.66 (1.26–2.18)	4.93e-09	95%	(0.90–3.02)	0.005	Large heterogeneity, small study effect, 95% prediction interval excludes the null value	Low
Suggestive (class III)												
Nitrogen dioxide	Li 2022 [46]	> 1000/367,017	27	Cohort, case–control	OR	1.11 (1.07–1.16)	0.000012	75%	(0.89–1.44)	0.003	Large heterogeneity, small study effect, excess significance bias, 95% prediction interval includes the null value	Moderate
Farm milk consumption	Brick 2020 [47]	> 1000/64,685	6	Cohort, cross-sectional	OR	0.68 (0.57–0.82)	0.000043	0%	(0.56–0.84)	0.40	None	Critically low
Passive exposure to tobacco smoking	Saulyte 2014 [48]	> 1000/960,660	63	Cohort, case–control, cross-sectional	RR	1.10 (1.06–1.15)	0.000208	85%	(0.77–1.60)	0.51	Large heterogeneity, excess significance bias, 95% prediction interval includes the null value	Critically low
Early dietary introduction of fish	Ierodiakonou 2016 [49]	> 1000/13,423	4	Cohort	OR	0.64 (0.50–0.82)	0.000425	38%	(0.26–1.56)	0.06	Small study effect, excess significance bias, 95% prediction interval includes the null value	High
History of Kawasaki disease	Lei 2021 [50]	> 1000/NR	4	Case–control, cross-sectional	OR	1.73 (1.29–2.31)	0.000372	89%	(0.46–6.49)	0.20	Large heterogeneity, excess significance bias, 95% prediction interval includes the null value	Critically low
Educational level	Chong and Chew 2018 [51]	> 1000/17,604	2	Cross-sectional	OR	1.74 (1.34–2.27)	0.000229	6%	(0.82–4.19)	0.04	Small study effect, excess significance bias, 95% prediction interval includes the null value	Critically low
Family history of allergic diseases	Chong and Chew 2018 [51]	> 1000/29,122	5	Cross-sectional	OR	2.62 (1.51–4.56)	0.000061	95%	(0.48–14.31)	0.66	Large heterogeneity, 95% prediction interval includes the null value	Critically low
Ambient particulate matter (PM2.5)	Li 2022 [46]	> 1000/227,200	15	Cohort, case–control	OR	1.13 (1.04–1.22)	0.000622	84%	(0.92–1.38)	0.01	Large heterogeneity, small study effect, excess significance bias, 95% prediction interval includes the null value	Moderate
Weak (class IV)												
Ambient particulate matter(PM_10_)	Li 2022 [46]	> 1000/380,084	28	Cohort, case–control	OR	1.07 (1.04–1.11)	0.00309	79%	(0.78 –1.64)	0.01	Large heterogeneity, small study effect, excess significance bias, 95% prediction interval includes the null value	Moderate
Sulfur dioxide	Li 2022 [46]	> 1000/321,737	18	Cohort, case–control	OR	1.14 (1.08–1.21)	0.00239	74%	(0.84–1.51)	0.89	Large heterogeneity, 95% prediction interval includes the null value	Moderate
Ozone	Li 2022 [46]	> 1000/275,049	12	Cohort, case–control	OR	1.06 (1.03–1.08)	0.019	89%	(0.88–1.30)	0.11	Large heterogeneity, 95% prediction interval includes the null value	Moderate
Prenatal smoke exposure	Li 2022 [52]	> 1000/23,319	6	Cohort	OR	1.12 (1.04–1.21)	0.0223	0%	(0.95–1.31)	0.12	95% prediction interval includes the null value	Critically low
Early life food sensitization	Alduraywish 2016 [53]	< 1000/4386	4	Cohort	OR	3.06 (1.90–4.94)	0.000007	38%	(0.55–17.04)	0.86	95% prediction interval includes the null value	Critically low
Postpartum smoke exposure	Li 2022 [52]	> 1000/144,325	7	Cohort	OR	1.19 (1.03–1.39)	0.0279	77%	(0.71–2.05)	0.98	Large heterogeneity, 95% prediction interval includes the null value	Critically low
Vitamin D status	Aryan et al. 2017 [54]	> 1000/51,576	11	Cohort, cross-sectional	OR	0.78 (0.63–0.96)	0.0358	84%	(0.34–1.82)	0.47	Large heterogeneity, 95% prediction interval includes the null value	Critically low
Obstructive sleep apnea	Cao et al. 2018 [55]	> 1000/NR	17	Cohort, cross-sectional	OR	1.55 (1.16–2.06)	0.00292	65%	(0.59–4.06)	0.38	Large heterogeneity, 95% prediction interval includes the null value	Low
Prenatal maternal psychosocial stress	Flanigan et al. 2018 [56]	< 1000/7685	3	Cohort, cross-sectional	OR	1.36 (1.08–1.71)	0.00991	43%	(0.69–2.70)	0.50	Excess significance bias, 95% prediction interval includes the null value	Moderate
Maternal oral contraceptive pill	Bai et al. 2020 [57]	< 1000/5386	5	Cohort, case–control, cross-sectional	OR	1.34 (1.07–1.68)	0.0110	38%	(0.71–2.53)	0.91	95% prediction interval includes the null value	Critically low
Caesarean delivery	Bager 2008 [58]	> 1000/56,832	7	Cohort	OR	1.24 (1.08–1.43)	0.00272	43%	(0.92–1.69)	0.83	95% prediction interval includes the null value	Low
Attention deficit hyperactivity disorder	Miyazaki et al. 2017 [59]	> 1000/51,709	5	Case–control, cross-sectional	OR	1.59 (1.13–2.22)	0.00376	93%	(0.52–4.85)	0.22	95% prediction interval includes the null value	Low
House dust mite	Chong and Chew 2018 [51]	< 1000/1157	2	Cross-sectional	OR	2.00 (1.19–3.37)	0.00853	36%	NA	NA	None	Critically low
Not significant (NS)												
Exposure to cats	Gao et al. 2020 [60]	< 1000/4234	5	Cohort, case–control	RR	0.80 (0.49–1.29)	0.357	61%	(0.20–2.23)	0.99	Large heterogeneity, 95% prediction interval includes the null value	Critically low
Exposure to dogs	Gao et al. 2020 [60]	> 1000/11,624	4	Cohort	RR	0.76 (0.57–1.03)	0.0695	0%	(0.50–1.16)	0.002	Small study effect, 95% prediction interval includes the null value	Critically low
Greenness	Cao et al. 2023 [61]	> 1000/50,993	10	Cohort, cross-sectional	OR	1.00 (0.99–1.00)	0.07	53%	(0.99–1.00)	0.11	Large heterogeneity, excess significance bias, 95% prediction interval includes the null value	Moderate
Carbon monoxide	Li 2022 [46]	> 1000/244,782	10	Cohort, case–control	OR	1.03 (1.00–1.05)	0.0960	83%	(0.82–1.41)	0.22	Large heterogeneity, 95% prediction interval includes the null value	Moderate
Maternal fish intake during pregnancy	Zhang 2017 [62]	> 1000/21,042	3	Cohort	OR	0.94 (0.60–1.64)	0.769	45%	(0.17–5.56)	0.58	95% prediction interval includes the null value	Critically low
Prenatal exposure to vitamin D	Pacheco 2018 [63]	> 1000/26,147	6	Cohort	OR	0.98 (0.84–1.15)	0.843	0%	(0.79–1.23)	0.17	95% prediction interval includes the null value	Critically low
Neonatal jaundice	Kuniyoshi 2021 [64]	> 1000/12,213	3	Cohort, case–control	OR	3.01 (0.88–10.28)	0.0629	93%	(0–5,909,514)	0.46	Large heterogeneity, 95% prediction interval includes the null value	Low
Phototherapy	Kuniyoshi 2021 [64]	> 1000/83,060	2	Cohort	OR	1.38 (0.93–2.04)	0.111	56%	NA	NA	Large heterogeneity	Low
Active exposure to tobacco smoking	Saulyte 2014 [48]	> 1000/152,307	34	Cohort, case–control, cross-sectional	RR	1.03 (0.92–1.15)	0.673	94%	(0.55–1.92)	0.61	Large heterogeneity, excess significance bias, 95% prediction interval includes the null value	Critically low
Childhood type 1 diabetes	Cardwell 2003 [65]	> 1000/72,808	11	Case–control, cross-sectional	OR	0.88 (0.68–1.13)	0.254	56%	(0.43–1.77)	0.89	Large heterogeneity, 95% prediction interval includes the null value	Critically low
Children and adolescents exposed to pesticides	Rodrigues 2021 [66]	< 1000/3686	2	Cohort	OR	2.77 (0.13–61.40)	0.519	0%	NA	NA	None	Low
Exposure to perfluoroalkyl substances	Luo 2023 [67]	< 1000/3396	4	Cohort, cross-sectional	OR	1.08 (0.96–1.20)	0.168	37%	(0.81–1.43)	0.03	Small study effect, 95% prediction interval includes the null value	Critically low
Multiple sclerosis	Monteiro 2011 [68]	> 1000/263,722	6	Case–control, cross-sectional	OR	0.81 (0.59–1.12)	0.260	70%	(0.26–2.55)	0.57	Large heterogeneity, 95% prediction interval includes the null value	Critically low
Indoor microbial aerosols exposures	Fakunle 2021 [69]	< 1000/2082	4	Cohort	RR	1.18 (0.93–1.49)	0.192	70%	(0.38–3.83)	0.24	Large heterogeneity, 95% prediction interval includes the null value	Low

*AMSTAR 2* A Measurement Tool to Assess Systematic Reviews 2, *CI* confidence interval, *NR* not reported, *NA* not available, *OR* odds ratio, *RR* relative risk, *HR* hazard ratio

### Environmental Risk Factors and Protective Factors

The 43 meta-analyses of potential environmental factors were classified into 31 potential environmental risk factors (10,806,206 total population, two studies not reported) and 11 potential environmental protective factors (823,883 total population), of which 1 potential factor had no effect on the occurrence of the disease (OR = 1, 95% CI 0.99–1.00). Of the 43 meta-analyses, the largest included 63 original studies and the smallest included 2 original studies, in which effect metrics included OR, RR, and HR. Among them, the study designs of 35 meta-analyses included cohort studies, 17 included case–control studies, and 22 included cross-sectional studies. Of the 43 random effects *p* values for the meta-analyses, 29 (67%) had *p* values < 0.05, 16 (37%) had *p* values < 0.001, and 8 (19%) had *p* values < 0.000001. Only 15 of 43 meta-analyses showed less than 50% heterogeneity (*I*^2^ < 50%). The number of individual studies less than 3 could not conduct Egger’ s test, which resulted in our 4 of 43 meta-analyses not obtaining Egger’s test data. Among the 43 meta-analyses, only 8 (19%) meta-analyses did not include more than 1000 AR cases. Among the 43 meta-analyses (excluding not accessible data), the numbers of small study effects and excessive significance bias were 11 and 13, respectively. The 95% prediction interval and the 95% confidence interval of the largest study excluded null in 4 of 43 (9%) meta-analyses and 28 of 43 (65%) meta-analyses, respectively. Table 2 provides the detailed characteristics and statistical test results of potential environmental risk and protective factors of AR.

**Fig. 2:**
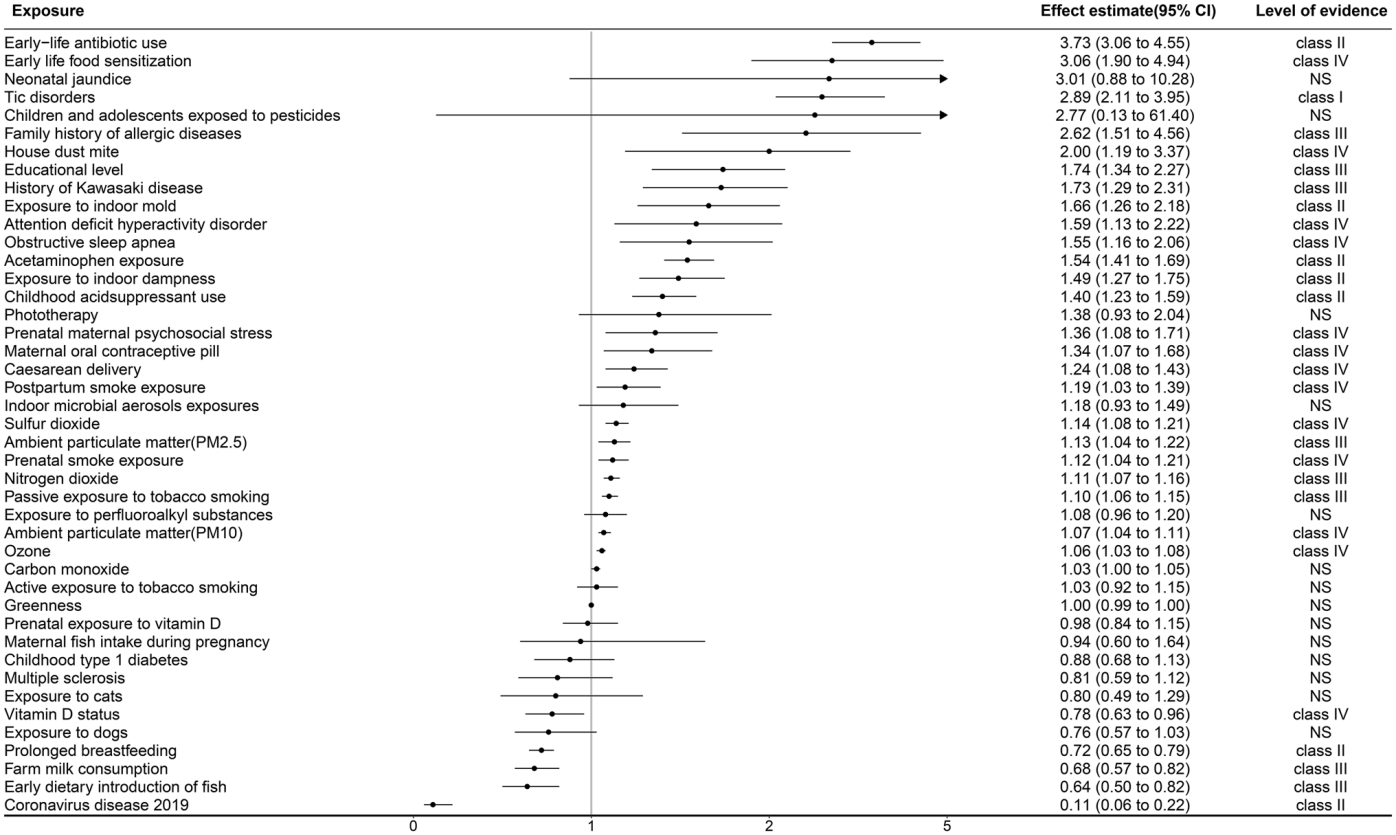
Summary estimates of the meta-analyses of potential environmental risk and protective factors for AR

Of all the environmental risk factors and protective factors, tic disorders (OR = 2.89, 95% CI 2.11–3.95) was the only one that was graded as convincing evidence (class I) that it was an environmental risk factor. Seven environmental risk factors and protective factors were graded as highly suggestive evidence (class II), of which early-life antibiotic use (OR = 3.73, 95% CI 3.06–4.55), exposure to indoor dampness (OR = 1.49, 95% CI 1.27–1.75), acetaminophen exposure (OR = 1.54, 95% CI 1.41–1.69), childhood acid suppressant use (OR = 1.40, 95% CI 1.23–1.59), and exposure to indoor mold (OR = 1.66, 95% CI 1.26–2.18) were environmental risk factors, and coronavirus disease 2019 (OR = 0.11, 95% CI 0.06–0.22) and prolonged breastfeeding (OR = 0.72, 95% CI 0.65–0.79) were environmental protective factors. Nitrogen dioxide and ambient particulate matter (PM_2.5_) from air pollution were graded as suggestive evidence (class III) as environmental risk factors, along with passive exposure to tobacco smoking, history of Kawasaki history, education level, and family history of allergies were also graded as recommended evidence. Farm milk consumption and early dietary introduction of fish as environmental protection factors were graded as suggestive evidence (class III). Ambient particulate matter (PM_10_), sulfur dioxide and ozone, as air pollution, were graded as weak evidence (class IV), and only vitamin D status as an environmental protective factor was rated as weak evidence (class IV). Figure 2 provides summary estimates of the meta-analyses of potential environmental risk and protective factors for AR.

In the subgroup analysis of children (Appendix p13–p14), except for the studies that could not be used for subgroup analysis, most subsets retained the evidence level, and only 4 changed the original evidence level. Exposure to indoor dampness was upgraded from class II to class I evidence, exposure to indoor mold was upgraded from class III to class I evidence, active exposure to tobacco smoking was upgraded from not significant to class II evidence, and vitamin D status was downgraded from class IV to not significant.

### Biomarkers

The 34 meta-analyses of potential biomarkers were classified into 32 single nucleotide polymorphism biomarkers (154,583 total population), one serum biomarker (915 total population), and one biomarker of nasal inflammation (3218 total population), the largest of which included 30 original studies and the smallest of which included 3 original studies. These meta-analyses included only case–control studies, and the effect metrics were based on OR and SMD. In the random-effects model, only 10 of 34 (29%) associations were statistically significant (*p* value < 0.05), of which only 3 (8%) had *p* values < 0.001 and only 1 (3%) had *p* value < 0.000001. Seven (70%) of 10 associations statistically significant meta-analyses included more than 1000 AR cases, but only two of the eight meta-analyses showed less than 50% heterogeneity (I^2^ < 50%). Four of 10 (40%) statistically significant meta-analyses had small study effects, and 2 of 10 (20%) had excessive significance bias. The 95% prediction interval and the 95% confidence interval of the largest study excluded null in 3 of 10 (30%) statistically significant meta-analyses and 4 of 10 (40%) meta-analyses, respectively.

There were no biomarkers that were found with convincing evidence (class I). The only biomarker graded as highly suggestive evidence (class II) was nasal nitric oxide in AR patients, and the only biomarker graded as suggestive evidence (class III) was interleukin 13 (IL-13) rs20541 polymorphism in AR patients. The Appendix (p18–p22) provides detailed test data, levels of evidence, and supplementary data for potential biomarkers of AR.

### AMSTAR 2 Quality Assessment

We conducted AMSTAR 2 quality assessments on all meta-analyses. Among potential environmental risk factors and protective factors, 1 of 43 (2%) meta-analyses was graded as high quality, 9 of 43 (21%) meta-analyses as moderate quality, 10 of 43 (23%) meta-analyses as low quality, and 23 of 43 (54%) meta-analyses as critically low quality. Of the potential biomarkers, none of the 34 meta-analyses were graded as high quality, one of the 34 (3%) meta-analyses was graded as moderate quality, 7 of the 34 (21%) meta-analyses were graded as low quality, and 26 of the 34 (76%) meta-analyses were graded as critically low quality. The methodological quality of most studies was flawed. No study provided complete search strategies, although by and large they appeared to be well developed. However, due to the lack of grey literature search, search registration, and consulting content experts in the field, it is difficult to give a very positive evaluation in the search strategy section.

## Discussion

To date, this study is the first comprehensive review in the field of AR to summarize the evidence using state-of-the-art evidence strategies, which comprehensively and systematically collect and re-analyze meta-analyses data on environmental risk factors, protective factors and biomarkers of AR, and stratifying the level of evidence. Only eight factors were graded as high credibility, of which tic disorders (class I), early-life antibiotic use, exposure to indoor dampness, acetaminophen exposure, childhood acid suppressant use and exposure to indoor mold were environmental risk factors (class II), and coronavirus disease 2019 and prolonged breastfeeding (class II) were environmental protection factors.

Tic disorder (TD) is the only environmental risk factor that has been graded as convincing evidence in AR. TD is a neuropsychiatric disorder with a clear genetic predisposition that begins in childhood and adolescence [70]. It includes a group of movement disorders of unknown cause and may be accompanied by hyperactivity, inattention, compulsive movements and thoughts, or other behavioral symptoms [71]. The first report on the association between TD and allergic reactions dates back to 1985, when Finegold [72] reported on four children who presented to allergy department, all of whom had high IgE levels, positive skin prick test, and allergic symptoms; three children were eventually diagnosed with Tourette syndrome (TS) and one child was highly suspected of having TS. One case–control study reported a doubling of the risk of TS in patients with allergic rhinitis in a model that considered all 4 allergic diseases simultaneously (adjusted OR = 2.18, 95% CI 1.83–2.59; *P* < 0.0001) [73]. Although the exact biological mechanisms have not been identified, TD may share genetic vulnerabilities with allergic disorders, which may include the dysregulation of excitability in cortical-basal ganglia (CBG) loops mediated by gamma-aminobutyric acid (GABA) transmission, a mediating role of cell adhesion molecules (CAMs) in hyperinflammation, and initiation of cytokines such as tumor necrosis factor and interleukins [39, 74–76]. Notably, the possession of a common etiology may be one of the important reasons for the association of AR and TD. In addition to shared familial genetic risk and environmental factors, pathogenic infections (e.g., enterovirus, Mycoplasma pneumoniae, group A streptococcus) are suspected to be important factors predisposing to co-morbidity between the two disease [77–79]. Our level of evidence suggests a strong association between AR and TD; however, we cannot infer a causal relationship between this association and the biological mechanisms between the two need to be further investigated.

Early-life antibiotic use is associated with AR and is an environmental risk factor for AR, which provides highly suggestive evidence. This association is noteworthy because antibiotics can prevent and cure many infections when the immune systems of infants and children are not fully developed, and because the cardiovascular and nervous systems of children are not fully developed, inappropriate antibiotic use may have an impact on future health outcomes [80, 81]. Regarding the potential mechanisms of their association, the interference of antibiotic exposure on the colonization of the intestinal flora is considered to be the most plausible mechanism at present [82]. The gut microbiome hypothesis has recently emerged as a link between antibiotic exposure and disease development, and the gut microbiome has also been shown to play an important role in the human immune system and in maintaining homeostasis [83]. In terms of AR, the gut microbiota is emerging as a new target of intervention and is becoming a hot research topic in the field of allergic diseases, the mechanisms of which may be related to gut ecological dysregulation and early disruption of immune regulation leading to the development of allergic diseases [84, 85]. Therefore, early-life antibiotic use may affect immature intestinal biota or affect their colonization numbers, resulting in changes in the developing immune system that could induce AR [83]. Although, the use of antibiotics in medical practice is inevitable, how to prevent antibiotic abuse and promote the rational use of antibiotics has become a current thorny issue in public health, which requires health care professionals or national health care planners to develop more comprehensive and efficient antibiotic stewardship initiatives, and the concept of rational antibiotic use needs to permeate all aspects of daily medical activities [86].

Childhood acid suppressant use is associated with AR and is an environmental risk factor for AR, which provides highly suggestive evidence. Gastric acid inhibiting drugs are thought to be one of the causes of allergic diseases, and the mechanism may be that the drug reduces gastric acid secretion, which directly leads to intestinal ecological dysregulation and increases the risk of allergy [87]. Although not fully understood, the underlying mechanisms by which acid-suppressive drugs and antibiotic use increase allergy include gut dysbiosis, a key part of which, in the case of acid-suppressive drugs, is reduced digestion of protein in the stomach. Increasing evidence from human and animal studies suggests that a diverse microbiome plays a central role in the development of a healthy immune system and that perturbations in the microbiome increase the risk of developing allergic diseases [88–90]. It is important to note that the association between gastroesophageal reflux disease (GERD) and food allergy cannot be completely discounted, since not all studies have adjusted for participants’ indications for acid inhibitors such as GERD, which suggests the possibility that confounding may account for the associations we observed [45]. Although Muhammad et al. [45] have conducted a subgroup analysis based on GERD status for the risk between GERD and allergic diseases, and the results also showed that the use of acid inhibitors is associated with a higher risk of allergy in GERD patients, due to the lack of corresponding data, no analysis of AR has been conducted, which makes it impossible for us to draw definitive conclusions.

Exposure to indoor mold and exposure to indoor dampness are associated with AR and are an environmental risk factor for AR, which provides highly suggestive evidence and upgrades to convincing evidence in the subgroup analysis of children. Indoor dampness and mold are important factors in residential indoor environment. On the one hand, it is related to high indoor water vapor content and insufficient ventilation; on the other hand, it is related to unreasonable construction and use of buildings that lead to excessive moisture or excessive water vapor accumulation in building materials, both of which create conditions for the breeding of moisture and mold [91]. Studies have found that self-reported problems with dampness or mold in buildings where people live or work are associated with respiratory or allergic diseases [92]. Signs of dampness, such as water leakage, water damage, visible mold, and mold odor, inevitably cause biological contamination, invasion of the respiratory tract, and allergic reactions [93]. In addition to indoor dampness and mold providing a good environment for microbial growth, the degradation of building materials by moisture and water vapor to volatilize chemicals may also be an important cause of allergy problems [94]. The age subgroup analysis showed that the level of evidence was upgraded from highly suggestive to convincing evidence in children, suggesting that children may be more susceptible to allergic reactions in indoor environments with dampness and mold, which may be related to the immature development of the immune system in children.

Acetaminophen exposure is associated with AR and is an environmental risk factor for AR, which provides highly suggestive evidence. Acetaminophen (paracetamol, APAP), one of the most widely used analgesics, is a classic dose-dependent liver injury drug and the most commonly used antipyretic analgesic drug in the world. Its antipyretic effect is slow and lasting, and it has the advantages of little irritation and few allergic reactions [95]. A multi-center, multi-country, cross-sectional study of children (6–7 and 13–14 years of age) from the International Study of Asthma and Allergy in Childhood (ISAAC) showed that acetaminophen use was associated with an increased risk of present symptoms of allergic rhinitis [96]. The mechanism of this association has not been unified, and the depletion of glutathione-S-transferase in the upper airway mucosa leading to increased oxidative stress, promoting the helper T-helper 2 (Th2) differentiation pathway and immunoglobulin E (IgE)-mediated response is one of the leading hypotheses [97–99]. Caballero et al. [100] identified lymphocyte aggregates in a group of paracetamol treated rats and determined that the inflammatory infiltrate was primarily composed of lymphoid aggregates and mast cells, suggesting that chronic exposure to paracetamol in a rat model is associated with the development of rhinitis. Although a clear causal relationship between acetaminophen and AR cannot be established from this study, we suggest that exposure to acetaminophen may be an important putative risk factor for the development of AR.

Prolonged breastfeeding is associated with AR and is an environmental protective factor for AR, which provides highly suggestive evidence. Breast milk is considered the ideal food for healthy full-term infants and contains a variety of biologically active substances with nutritional, immune and disease-fighting and growth-promoting metabolic functions; breastfeeding has health benefits for infants, including short-term (e.g., reduction of neonatal and child deaths from infectious diseases) and long-term (e.g., prevention of adult obesity, diabetes, cardiovascular disease and metabolic disorders) effects [101, 102]. The immune effect of breast milk is expressed through various immunoglobulin (sIgA, IgA, IgG, IgM, IgE, IgD) compounds that are derived from the maternal immune response, and this protective mechanism allows the newborn to develop high immunity directly against the infectious source recognized by the mother [103, 104]. Moreover, the microorganisms in breast milk are an important link in initiating the immune function of the infant gut and driving the colonization of intestinal microorganisms; its short-chain fatty acids, a metabolite produced by microorganisms, can affect peripheral blood T cells, especially regulatory T (Treg) cells, by inhibiting histone deacetylases to achieve immune effects [105]. The meta-analysis by Hoang et al. [42] included in this study showed that either extended exclusive breastfeeding or extended non-exclusive breastfeeding (≥ 6 months) reduced the risk of AR, which is in line with the WHO recommendation that every infant should be breastfed for at least 6 months [102].

Coronavirus disease 2019 (COVID-19) is associated with AR and is an environmental protective factor for AR, which provides highly suggestive evidence. From the studies [43], we included that the prevalence of allergic rhinitis in confirmed cases of COVID-19 was much lower than in the normal population; however, it is difficult to establish a clear causal relationship for this association. Data from the Korean Adolescent Risk Behavior Network Survey (KYRBWS-2019 and 2020) reported a decrease in the incidence of allergic rhinitis in 2020 compared with pre-COVID-19 [106]. During the COVID-19 pandemic, disposable medical masks have been widely used, which can prevent most pollen particles from inhaling into the nasal cavity, thereby reducing the incidence of nasal symptoms in patients [107]. Furthermore, the decline of allergic rhinitis could be related to the blockage of respiratory infections by COVID-19 prophylaxis, or the reduction of socioeconomic activities and environmental factors that trigger allergies, resulting in a reduction of particulate matter or air pollutants; and the maintenance of social distance also prevents respiratory infections to some extent and reduces the chance of AR [108]. However, the current studies cannot clearly explain whether the COVID-19 disease itself reduces the risk of AR or lifestyle changes during the COVID-19 period reduce the risk of AR, which may require rigorous case–control studies or randomized controlled studies to explain this phenomenon.

### Limitations

Although we have conducted standardized evidence hierarchy on the potential environmental risks, protective factors and biomarkers of AR, there are still some limitations. First, because the included studies were observational, the causality of these associations remains difficult to determine. Second, the diagnosis of AR in some children is based on questionnaires from parents and lacks objective clinical examination and professional diagnosis. Third, confounding factors present in observational studies are difficult to completely rule out, and these participating confounders may confound the final results. Fourth, this study focused only on meta-analyses that had already been published and may have missed associations that were not assessed in other systematic reviews or meta-analyses. Fifth, because of the level of evidence, an environmental factor or biomarker with a strong effect may be underestimated, such as early life food sensitization, since fewer than 1000 studies would be graded as class IV evidence. Finally, the umbrella review inherited most of the study limitations of the included studies. For example, if the latter evaluates the relationship between the study factors and morbidity rather than causation, then the umbrella review can also only evaluate the relationship between the study factors and morbidity but not causation.

## Conclusion

In this umbrella review, a total of 76 potential environmental risk factors, environmental protection factors and biomarkers for AR were established, forming a complete hierarchy of evidence. Among them, tic disorders (class I), early-life antibiotic use, exposure to indoor dampness, acetaminophen exposure, childhood acid suppressant use to exposure to indoor, coronavirus disease 2019 and prolonged breastfeeding (class II) were rated as high credibility. However, we could not infer causality for these associations, which would require a more rigorous design and high-quality studies to confirm this finding.

### Supplementary Information

Below is the link to the electronic supplementary material.

Supplementary file1 (PDF 6487 KB)

## Data Availability

Not applicable.
