# Supplementary material for: Environmental Risk Factors, Protective Factors, and Biomarkers for Allergic Rhinitis: A Systematic Umbrella Review of the Evidence
Source: Clin Rev Allergy Immunol. 2023 Jul 25;65(2):188–205. doi: 10.1007/s12016-023-08964-2 (PMC10567804; doi:10.1007/s12016-023-08964-2)

Supplementary material

Environmental risk factors, protective factors, and biomarkers for allergic rhinitis: a systematic umbrella review of the evidence

1. PRISMA checklist
2. Full search strategy
3. Definitions of environmental risk/protective factor and biomarker
4. The list of excluded articles by full text screening with exclusion reason
5. References of the excluded meta-analyses by full text screening
6. Subset analysis of potential environmental risk/protective factors for AR in children
7. Supplementary analyses result of environmental risk and protective factors
8. Potential biomarker of AR, with details of statistical test results
9. References of potential biomarkers
10. Supplementary analyses result of biomarker
11. Figure S1. Random-effects meta-analysis of the association between tic disorders and AR
12. Figure S2. Random-effects meta-analysis of the association between early-life antibiotic use and AR
13. Figure S3. Random-effects meta-analysis of the association between exposure to indoor dampness and AR
14. Figure S4. Random-effects meta-analysis of the association between prolonged breastfeeding and AR
15. Figure S5. Random-effects meta-analysis of the association between coronavirus disease 2019 and AR
16. Figure S6. Random-effects meta-analysis of the association between acetaminophen exposure and AR
17. Figure S7. Random-effects meta-analysis of the association between childhood acid suppressant use and AR
18. Figure S8. Random-effects meta-analysis of the association between exposure to indoor mold and AR
19. Figure S9. Random-effects meta-analysis of the association between Ambient particulate matter(PM10) and AR
20. Figure S10. Random-effects meta-analysis of the association between sulfur dioxide and AR
21. Figure S11. Random-effects meta-analysis of the association between nitrogen dioxide and AR
22. Figure S12. Random-effects meta-analysis of the association between farm milk consumption and AR
23. Figure S13. Random-effects meta-analysis of the association between passive exposure to tobacco smoking and AR
24. Figure S14. Random-effects meta-analysis of the association between early dietary introduction of fish and AR
25. Figure S15. Random-effects meta-analysis of the association between history of Kawasaki disease and AR
26. Figure S16. Random-effects meta-analysis of the association between educational level and AR
27. Figure S17. Random-effects meta-analysis of the association between family history of allergic diseases and AR
28. Figure S18. Random-effects meta-analysis of the association between ambient particulate matter(PM2.5) and AR
29. Figure S19. Random-effects meta-analysis of the association between carbon monoxide and AR
30. Figure S20. Random-effects meta-analysis of the association between ozone and AR
31. Figure S21. Random-effects meta-analysis of the association between prenatal smoke exposure and AR
32. Figure S22. Random-effects meta-analysis of the association between early life food sensitization and AR
33. Figure S23. Random-effects meta-analysis of the association between postpartum smoke exposure and AR
34. Figure S24. Random-effects meta-analysis of the association between vitamin D status and AR
35. Figure S25. Random-effects meta-analysis of the association between obstructive sleep apnea and AR
36. Figure S26. Random-effects meta-analysis of the association between prenatal maternal psychosocial stress and AR
37. Figure S27. Random-effects meta-analysis of the association between maternal oral contraceptive pill and AR
38. Figure S28. Random-effects meta-analysis of the association between caesarean delivery and AR
39. Figure S29. Random-effects meta-analysis of the association between attention deficit hyperactivity disorder and AR
40. Figure S30. Random-effects meta-analysis of the association between house dust mite and AR
41. Figure S31. Random-effects meta-analysis of the association between exposure to cats and AR
42. Figure S32. Random-effects meta-analysis of the association between exposure to dogs and AR
43. Figure S33. Random-effects meta-analysis of the association between greenness and AR
44. Figure S34. Random-effects meta-analysis of the association between maternal fish intake during pregnancy and AR
45. Figure S35. Random-effects meta-analysis of the association between prenatal exposure to vitamin D and AR
46. Figure S36. Random-effects meta-analysis of the association between neonatal jaundice and AR
47. Figure S37. Random-effects meta-analysis of the association between phototherapy and AR
48. Figure S38. Random-effects meta-analysis of the association between active exposure to tobacco smoking and AR
49. Figure S39. Random-effects meta-analysis of the association between childhood type 1 diabetes and AR
50. Figure S40. Random-effects meta-analysis of the association between children and adolescents exposed to pesticides and AR

51. Figure S41. Random-effects meta-analysis of the association between exposure to perfluoroalkyl substances and AR
52. Figure S42. Random-effects meta-analysis of the association between multiple sclerosis and AR
53. Figure S43. Random-effects meta-analysis of the association between indoor microbial aerosols exposures and AR
54. Figure S44. Random-effects meta-analysis of the association between nasal nitric oxide and AR
55. Figure S45. Random-effects meta-analysis of the association between IL-13 rs20541 polymorphism and AR
56. Figure S46. Random-effects meta-analysis of the association between serum IL-4 level and AR
57. Figure S47. Random-effects meta-analysis of the association between ADAM33 S2 polymorphism and AR
58. Figure S48. Random-effects meta-analysis of the association between ADAM33 V4 polymorphism and AR
59. Figure S49. Random-effects meta-analysis of the association between ADAM33 Q−1 polymorphism and AR
60. Figure S50. Random-effects meta-analysis of the association between ACEI/D polymorphism and AR
61. Figure S51. Random-effects meta-analysis of the association between TNF α rs1800629 polymorphism and AR
62. Figure S52. Random-effects meta-analysis of the association between CTLA-4 rs11571302 polymorphism and AR
63. Figure S53. Random-effects meta-analysis of the association between IL-4R rs1801275 polymorphism and AR
64. Figure S54. Random-effects meta-analysis of the association between ADAM33 T1 polymorphism and AR
65. Figure S55. Random-effects meta-analysis of the association between ADAM33 T2 polymorphism and AR
66. Figure S56. Random-effects meta-analysis of the association between IL-4 rs2243250 polymorphism and AR
67. Figure S57. Random-effects meta-analysis of the association between IL-4RA Ile50Val polymorphism and AR
68. Figure S58. Random-effects meta-analysis of the association between IL-4RA Ser478Pro polymorphism and AR
69. Figure S59. Random-effects meta-analysis of the association between IL-4RA Gln551Arg polymorphism and AR
70. Figure S60. Random-effects meta-analysis of the association between IL-13 rs1800925 polymorphism and AR
71. Figure S61. Random-effects meta-analysis of the association between CD14 rs2569190 polymorphism and AR
72. Figure S62. Random-effects meta-analysis of the association between TGF β1 rs1800469 polymorphism and AR
73. Figure S63. Random-effects meta-analysis of the association between CTLA-4 rs3087243 polymorphism and AR
74. Figure S64. Random-effects meta-analysis of the association between CTLA-4 rs231725 polymorphism and AR
75. Figure S65. Random-effects meta-analysis of the association between CTLA-4 rs11571315 polymorphism and AR
76. Figure S66. Random-effects meta-analysis of the association between IL-4 rs2227284 polymorphism and AR
77. Figure S67. Random-effects meta-analysis of the association between IL-4 rs2227284 polymorphism and AR
78. Figure S68. Random-effects meta-analysis of the association between FOX P3 rs3761548 polymorphism and AR
79. Figure S69 Random-effects meta-analysis of the association between FOX P3 rs2232365 polymorphism and AR
80. Figure S70 Random-effects meta-analysis of the association between IL-18 rs1946518 polymorphism and AR
81. Figure S71 Random-effects meta-analysis of the association between IL-18 rs187238 polymorphism and AR
82. Figure S72 Random-effects meta-analysis of the association between IL-18 rs4988359 polymorphism and AR
83. Figure S73 Random-effects meta-analysis of the association between Tim-3 rs10515746 polymorphism and AR
84. Figure S74 Random-effects meta-analysis of the association between Tim-3 rs10515746 polymorphism and AR
85. Figure S75 Random-effects meta-analysis of the association between TAP1 333 polymorphism and AR
86. Figure S76 Random-effects meta-analysis of the association between TAP1 637 polymorphism and AR
87. Figure S77 Random-effects meta-analysis of the association between TSL rs1898671 polymorphism and AR

PRISMA checklist

Section and Topic	Item #	Checklist item	Location where item is reported
TITLE			
Title	1	Identify the report as a systematic review.	Manuscript p 1
ABSTRACT			
Abstract	2	See the PRISMA 2020 for Abstract checklist.	Supplementary p 5
INTRODUCTION			
Rationale	3	Describe the rationale for the review in the context of existing knowledge.	Manuscript p 1-2
Objectives	4	Provide an explicit statement of the objective(s) or question(s) the review addresses.	Manuscript p 1-2
METHODS			
Eligibility criteria	5	Specify the inclusion and exclusion criteria for the review and how studies were grouped for the syntheses.	Manuscript p 2
Information sources	6	Specify all databases, registers, websites, organisations, reference lists and other sources searched or consulted to identify studies. Specify the date when each source was last searched or consulted.	Manuscript p 2, Figure 1
Search strategy	7	Present the full search strategies for all databases, registers and websites, including any filters and limits used.	Manuscript p 2, Supplementary material p 6
Selection process	8	Specify the methods used to decide whether a study met the inclusion criteria of the review, including how many reviewers screened each record and each report retrieved, whether they worked independently, and if applicable, details of automation tools used in the process.	Manuscript p 2-3
Data collection process	9	Specify the methods used to collect data from reports, including how many reviewers collected data from each report, whether they worked independently, any processes for obtaining or confirming data from study investigators, and if applicable, details of automation tools used in the process.	Manuscript p 2-3
Data items	10a	List and define all outcomes for which data were sought. Specify whether all results that were compatible with each outcome domain in each study were sought (e.g. for all measures, time points, analyses), and if not, the methods used to decide which results to collect.	Manuscript p 3-4
	10b	List and define all other variables for which data were sought (e.g. participant and intervention characteristics, funding sources). Describe any assumptions made about any missing or unclear information.	Manuscript p 3-4
Study risk of bias assessment	11	Specify the methods used to assess risk of bias in the included studies, including details of the tool(s) used, how many reviewers assessed each study and whether they worked independently, and if applicable, details of automation tools used in the process.	Manuscript p 3
Effect measures	12	Specify for each outcome the effect measure(s) (e.g. risk ratio, mean difference) used in the synthesis or presentation of results.	Manuscript p 3
Synthesis methods	13a	Describe the processes used to decide which studies were eligible for each synthesis (e.g. tabulating the study intervention characteristics and comparing against the planned groups for each synthesis (item #5)).	Manuscript p 3
	13b	Describe any methods required to prepare the data for presentation or synthesis, such as handling of missing summary statistics, or data conversions.	Manuscript p 3
	13c	Describe any methods used to tabulate or visually display results of individual studies and syntheses.	Manuscript p 3
	13d	Describe any methods used to synthesize results and provide a rationale for the choice(s). If meta-analysis was performed, describe the model(s), method(s) to identify the presence and extent of statistical heterogeneity, and software package(s) used.	Manuscript p 3
	13e	Describe any methods used to explore possible causes of heterogeneity among study results (e.g. subgroup analysis, meta-regression).	Manuscript p 3
	13f	Describe any sensitivity analyses conducted to assess robustness of the synthesized results.	Manuscript p 3
Reporting bias assessment	14	Describe any methods used to assess risk of bias due to missing results in a synthesis (arising from reporting biases).	Manuscript p 3
Certainty	15	Describe any methods used to assess certainty (or confidence) in the body of	Manuscript p 3

Section and Topic	Item #	Checklist item	Location where item is reported
assessment		evidence for an outcome.	
RESULTS			
Study selection	16a	Describe the results of the search and selection process, from the number of records identified in the search to the number of studies included in the review, ideally using a flow diagram.	Manuscript p 4 Figure 1
	16b	Cite studies that might appear to meet the inclusion criteria, but which were excluded, and explain why they were excluded.	Supplementary p 7-11
Study characteristics	17	Cite each included study and present its characteristics.	Table 2
Risk of bias in studies	18	Present assessments of risk of bias for each included study.	Table 2 Manuscript p 4
Results of individual studies	19	For all outcomes, present, for each study: (a) summary statistics for each group (where appropriate) and (b) an effect estimate and its precision (e.g. confidence/credible interval), ideally using structured tables or plots.	Table 2 Figure 2, S1-77
Results of syntheses	20a	For each synthesis, briefly summarise the characteristics and risk of bias among contributing studies.	Table 2 Figure 2, S1-77
	20b	Present results of all statistical syntheses conducted. If meta-analysis was done, present for each the summary estimate and its precision (e.g. confidence/credible interval) and measures of statistical heterogeneity. If comparing groups, describe the direction of the effect.	Table 2 Figure 2, S1-77
	20c	Present results of all investigations of possible causes of heterogeneity among study results.	Manuscript p 4-5, Supplementary p12-16
	20d	Present results of all sensitivity analyses conducted to assess the robustness of the synthesized results.	Manuscript p 4-5, Supplementary p12-16
Reporting biases	21	Present assessments of risk of bias due to missing results (arising from reporting biases) for each synthesis assessed.	Manuscript p 4-5, Supplementary p12-16
Certainty of evidence	22	Present assessments of certainty (or confidence) in the body of evidence for each outcome assessed.	Table 2, Figure 2
DISCUSSION			
Discussion	23a	Provide a general interpretation of the results in the context of other evidence.	Manuscript p 11-14
	23b	Discuss any limitations of the evidence included in the review.	Manuscript p 14
	23c	Discuss any limitations of the review processes used.	Manuscript p 14
	23d	Discuss implications of the results for practice, policy, and future research.	Manuscript p 14
OTHER INFORMATION			
Registration and protocol	24a	Provide registration information for the review, including register name and registration number, or state that the review was not registered.	Manuscript p 2
	24b	Indicate where the review protocol can be accessed, or state that a protocol was not prepared.	Manuscript p 2
	24c	Describe and explain any amendments to information provided at registration or in the protocol.	Not changed
Support	25	Describe sources of financial or non-financial support for the review, and the role of the funders or sponsors in the review.	Manuscript p 14
Competing interests	26	Declare any competing interests of review authors.	Manuscript p 14
Availability of data, code and other materials	27	Report which of the following are publicly available and where they can be found: template data collection forms; data extracted from included studies; data used for all analyses; analytic code; any other materials used in the review.	Manuscript p 14

From: Page MJ, McKenzie JE, Bossuyt PM, Boutron I, Hoffmann TC, Mulrow CD, et al. The PRISMA 2020 statement: an updated guideline for reporting systematic reviews. BMJ 2021;372:n71. doi: 10.1136/bmj.n71

PRISMA Abstract checklist

Some checklist items cannot be included in the abstract due to the word count restriction. (<170 words)

Section and Topic	Item #	Checklist item	Reported (Yes/No)
TITLE			
Title	1	Identify the report as a systematic review.	Yes
BACKGROUND			
Objectives	2	Provide an explicit statement of the main objective(s) or question(s) the review addresses.	Yes
METHODS			
Eligibility criteria	3	Specify the inclusion and exclusion criteria for the review.	Yes
Information sources	4	Specify the information sources (e.g. databases, registers) used to identify studies and the date when each was last searched.	Yes
Risk of bias	5	Specify the methods used to assess risk of bias in the included studies.	No
Synthesis of results	6	Specify the methods used to present and synthesise results.	No
RESULTS			
Included studies	7	Give the total number of included studies and participants and summarise relevant characteristics of studies.	Yes
Synthesis of results	8	Present results for main outcomes, preferably indicating the number of included studies and participants for each. If meta-analysis was done, report the summary estimate and confidence/credible interval. If comparing groups, indicate the direction of the effect (i.e. which group is favoured).	Yes
DISCUSSION			
Limitations of evidence	9	Provide a brief summary of the limitations of the evidence included in the review (e.g. study risk of bias, inconsistency and imprecision).	No
Interpretation	10	Provide a general interpretation of the results and important implications.	No
OTHER			
Funding	11	Specify the primary source of funding for the review.	No
Registration	12	Provide the register name and registration number.	Yes

From: Page MJ, McKenzie JE, Bossuyt PM, Boutron I, Hoffmann TC, Mulrow CD, et al. The PRISMA 2020 statement: an updated guideline for reporting systematic reviews. BMJ 2021;372:n71. doi: 10.1136/bmj.n71

Full search strategy (from inception to 31 December 2022)

PubMed
((Rhinitis Allergic[MeSH Terms]) OR (Allergic Rhinitides[Title/Abstract])) OR (Rhinitides, Allergic[Title/Abstract])) OR (Allergic Rhinitis[Title/Abstract])) OR (Seasonal Allergic Rhinitis[Title/Abstract])) OR (Seasonal Allergic Rhinitides[Title/Abstract])) OR (Pollen Allergy[Title/Abstract])) OR (Pollen Allergies[Title/Abstract])) OR (Hyperesthetic Rhinitis[Title/Abstract])) OR (Perennial Allergic Rhinitis[Title/Abstract])) AND ((Meta-Analy*) OR (Meta-Analysis[Publication Type])) OR (Meta-Analysis as Topic[MeSH Terms])) OR (Systematic Reviews as Topic[MeSH Terms])) OR (Systematic Review*)) OR (Systematic Review[Publication Type]))
**Ebase**
('allergic rhinitis'/exp OR 'allergic rhinitis':ab,ti OR 'allergic rhinitides':ab,ti OR 'seasonal allergic rhinitis':ab,ti OR 'seasonal allergic rhinitides':ab,ti OR 'pollen allergy':ab,ti OR 'pollen allergies':ab,ti OR 'hyperesthetic rhinitis':ab,ti OR 'perennial allergic rhinitis':ab,ti) AND (meta-analy*/exp OR 'meta analysis':ab,ti OR ('meta analysis':ab,ti AND topic:ab,ti) OR systematic review*/exp OR 'systematic review':ab,ti OR ('systematic reviews':ab,ti AND topic:ab,ti)
**Web of Science**
TS=('allergic rhinitis' OR 'allergic rhinitides' OR 'seasonal allergic rhinitis' OR 'seasonal allergic rhinitides' OR 'pollen allergy' OR 'pollen allergies' OR 'hyperesthetic rhinitis' OR 'perennial allergic rhinitis') AND TS=('meta-analy*' OR 'meta analysis' OR 'systematic review*')
**Cochrane Database of Systematic Reviews**
('allergic rhinitis' OR 'allergic rhinitides' OR 'seasonal allergic rhinitis' OR 'seasonal allergic rhinitides' OR 'pollen allergy' OR 'pollen allergies' OR 'hyperesthetic rhinitis' OR 'perennial allergic rhinitis')

Definitions of environmental risk/protective factor and biomarker

Environmental risk/protective factor
A risk factor is defined as any attribute, characteristic, or exposure of an individual that increases the likelihood of developing a disease or injury.<br><br>* Additionally, in our review, a protective factor was defined as any attribute, characteristic, or exposure of an individual that reduced the likelihood of developing disease or injury.<br><br>WHO. Health topics: risk factors. http://www.who.int/topics/risk_factors/en/ (accessed on Oct 30th, 2019).
Biomarker
A biomarker is defined as any substance, structure, or process that can be measured in vivo or in its products that can affect or predict the incidence of an outcome or disease.<br><br>WHO. WHO International Programme on Chemical Safety Biomarkers in Risk Assessment: validity and validation. http://www.inchem.org/documents/ehc/ehc/ehc222.htm (accessed on Oct 30th, 2019).

The list of excluded articles by full text screening with exclusion reason

1	Zou, et al. 2018	Another eligible meta-analysis with the same topic was included
2	Wang, et al. 2022	Another eligible meta-analysis with the same topic was included
3	Zhang, et al. 2022	Another eligible meta-analysis with the same topic was included
4	Rosario, et al. 2021	Another eligible meta-analysis with the same topic was included
5	Takkouche, et al. 2008	Another eligible meta-analysis with the same topic was included
6	Duong, et al. 2022	Another eligible meta-analysis with the same topic was included
7	Malmir, et al. 2022	Another eligible meta-analysis with the same topic was included
8	Zhou, et al. 2021	Another eligible meta-analysis with the same topic was included
9	Lin, et al. 2021	Another eligible meta-analysis with the same topic was included
10	Luo, et al. 2023	Another eligible meta-analysis with the same topic was included
11	Ye, et al. 2022	Another eligible meta-analysis with the same topic was included
12	Lambert, et al. 2017	Another eligible meta-analysis with the same topic was included
13	Wu, et al. 2022	Another eligible meta-analysis with the same topic was included
14	Mendell, et al. 2011	Another eligible meta-analysis with the same topic was included
15	Tischer, et al. 2011	Another eligible meta-analysis with the same topic was included
16	Lodge, et al. 2015	Another eligible meta-analysis with the same topic was included
17	Mimouni, et al. 2002	Another eligible meta-analysis with the same topic was included
18	Kim, et al. 2016	Another eligible meta-analysis with the same topic was included
19	Das, et al. 2015	Another eligible meta-analysis with the same topic was included
20	Tang, et al. 2020	Another eligible meta-analysis with the same topic was included
21	Bunyavanich, et al. 2011	Another eligible meta-analysis with the same topic was included
22	Ying, et al. 2013	Another eligible meta-analysis with the same topic was included
23	Ying, et al. 2012	Another eligible meta-analysis with the same topic was included
24	Li, et al. 2017	Another eligible meta-analysis with the same topic was included
25	Guo, et al. 2014	Another eligible meta-analysis with the same topic was included
26	Huang, et al. 2016	Another eligible meta-analysis with the same topic was included
27	Tang, et al. 2020	Another eligible meta-analysis with the same topic was included
28	Bunyavanich, et al. 2011	Another eligible meta-analysis with the same topic was included
29	Ying, et al. 2013	Another eligible meta-analysis with the same topic was included
30	Ying, et al. 2012	Another eligible meta-analysis with the same topic was included
31	Li, et al. 2017	Another eligible meta-analysis with the same topic was included
32	Guo, et al. 2014	Another eligible meta-analysis with the same topic was included
33	Huang, et al. 2016	Another eligible meta-analysis with the same topic was included
34	Wang, et al. 2021	Another eligible meta-analysis with the same topic was included
35	Lødrup, et al. 2012	It was not an observational systematic review and meta-analysis study
36	Burte, et al. 2018	It was not an observational systematic review and meta-analysis study
37	Kanazawa, et al. 2018	It was not an observational systematic review and meta-analysis study
38	Venter, et al. 2020	It was not an observational systematic review and meta-analysis study
39	Fuertes, et al. 2021	It was not an observational systematic review and meta-analysis study
40	Stratakis, et al. 2017	It was not an observational systematic review and meta-analysis study
41	Tischer, et al. 2013	It was not an observational systematic review and meta-analysis study
42	Savouré, et al. 2021	It was not an observational systematic review and meta-analysis study
43	Tareke, et al. 2020	It was not an observational systematic review and meta-analysis study
44	Yepes-Nuñez, et al. 2018	It was not an observational systematic review and meta-analysis study
45	Luo, et al. 2022	It was not an observational systematic review and meta-analysis study
46	Li, et al. 2022	It was not an observational systematic review and meta-analysis study
47	Tischer, et al. 2018	It was not an observational systematic review and meta-analysis study
48	Venter, et al. 2022	It was not an observational systematic review and meta-analysis study
49	Fuertes, et al. 2016	It was not an observational systematic review and meta-analysis study
50	Schindler, et al. 2016	It was not an observational systematic review and meta-analysis study
51	Idrose, et al. 2016	It was not an observational systematic review and meta-analysis study
52	Best, et al. 2016	It was not an observational systematic review and meta-analysis study
53	Bønnelykke, et al. 2013	It was not an observational systematic review and meta-analysis study
54	Eller, et al. 2008	It was not an observational systematic review and meta-analysis study
55	Hamizan, et al. 2019	It was not an observational systematic review and meta-analysis study
56	Bunyavanich, et al. 2014	It was not an observational systematic review and meta-analysis study
57	Shiue, et al. 2015	It was not an observational systematic review and meta-analysis study
58	Shaoqing, et al. 2011	It was not an observational systematic review and meta-analysis study

59	Waage, et al. 2018	It was not an observational systematic review and meta-analysis study
60	Tischer, et al. 2011	It was not an observational systematic review and meta-analysis study
61	Testa, et al. 2020	Insufficient data were provided for reanalysis
62	Patelarou, et al. 2011	Insufficient data were provided for reanalysis
63	Conlan, et al. 2021	Insufficient data were provided for reanalysis
64	Kremmyda, et al. 2011	Insufficient data were provided for reanalysis
65	Obbagy, et al. 2019	Insufficient data were provided for reanalysis
66	Farronato, et al. 2020	Insufficient data were provided for reanalysis
67	Güngör, et al. 2019	Insufficient data were provided for reanalysis
68	Yang, et al. 2016	Insufficient data were provided for reanalysis
69	Caillaud, et al. 2018	Insufficient data were provided for reanalysis
70	Güngör, et al. 2019	Insufficient data were provided for reanalysis
71	Tamasauskiene, et al. 2020	Insufficient data were provided for reanalysis
72	Huang, et al. 2021	Insufficient data were provided for reanalysis
73	Donovan, et al. 2020	Insufficient data were provided for reanalysis
74	Wang, et al. 2021	Insufficient data were provided for reanalysis
75	Güngör, et al. 2019	Insufficient data were provided for reanalysis
76	Andersson, et al. 2016	Insufficient data were provided for reanalysis
77	Hofmann, et al. 2021	Insufficient data were provided for reanalysis
78	Güngör, et al. 2019	Insufficient data were provided for reanalysis
79	Güngör, et al. 2019	Insufficient data were provided for reanalysis
80	Tarini, et al. 2006	Insufficient data were provided for reanalysis
81	Lin, et al. 2013	Insufficient data were provided for reanalysis
82	Gupta, et al. 2022	Insufficient data were provided for reanalysis
83	Hur, et al. 2014	Insufficient data were provided for reanalysis
84	Obbagy, et al. 2019	Insufficient data were provided for reanalysis
85	Khaleva, et al. 2019	Insufficient data were provided for reanalysis
86	Hu, et al. 2017	Insufficient data were provided for reanalysis
87	Feng, et al. 2023	Insufficient data were provided for reanalysis
88	Pang, et al. 2022	Insufficient data were provided for reanalysis
89	Xu, et al. 2014	Insufficient data were provided for reanalysis
90	Guo, et al. 2022	Insufficient data were provided for reanalysis
91	Zhang, et al. 2017	Insufficient data were provided for reanalysis
92	Yang, et al. 2022	Insufficient data were provided for reanalysis
93	Zheng, et al. 2019	Insufficient data were provided for reanalysis
94	Liu, et al. 2017	Insufficient data were provided for reanalysis
95	Zhang, et al. 2022	Insufficient data were provided for reanalysis
96	Tharabenjasin, et al. 2022	Insufficient data were provided for reanalysis
97	Wongtrakul, et al. 2020	It is not a risk factor, protective factor or biomarker study of allergic rhinitis
98	Rodrigues, et al. 2021	It is not a risk factor, protective factor or biomarker study of allergic rhinitis
99	Zhu, et al. 2020	It is not a risk factor, protective factor or biomarker study of allergic rhinitis
100	Chen, et al. 2021	It is not a risk factor, protective factor or biomarker study of allergic rhinitis
101	Campbell, et al. 2015	It is not a risk factor, protective factor or biomarker study of allergic rhinitis
102	Arroyave, et al. 2014	It is not a risk factor, protective factor or biomarker study of allergic rhinitis
103	He, et al. 2017	It is not a risk factor, protective factor or biomarker study of allergic rhinitis
104	Heinrich, et al. 2017	It is not a risk factor, protective factor or biomarker study of allergic rhinitis
105	Kitinoja, et al. 2020	It is not a risk factor, protective factor or biomarker study of allergic rhinitis
106	Liu, et al. 2020	It is not a risk factor, protective factor or biomarker study of allergic rhinitis
107	Baron, et al. 2020	It is not a risk factor, protective factor or biomarker study of allergic rhinitis
108	Knudgaard, et al. 2021	It is not a risk factor, protective factor or biomarker study of allergic rhinitis
109	Charoenngam, et al. 2021	It is not a risk factor, protective factor or biomarker study of allergic rhinitis
110	Liu, et al. 2022	It is not a risk factor, protective factor or biomarker study of allergic rhinitis
111	Schans, et al. 2017	It is not a risk factor, protective factor or biomarker study of allergic rhinitis

References of the excluded meta-analyses by full text screening

1. Zou QY, Shen Y, Ke X, Hong SL, Kang HY. Exposure to air pollution and risk of prevalence of childhood allergic rhinitis: A meta-analysis. Int J Pediatr Otorhinolaryngol. 2018;112:82-90. doi:10.1016/j.ijporl.2018.06.039.
2. Wang H, Li XB, Chu XJ, et al. Ambient air pollutants increase the risk of immunoglobulin E-mediated allergic diseases: a systematic review and meta-analysis. Environ Sci Pollut Res Int. 2022;29(33):49534-49552. doi:10.1007/s11356-022-20447-z.
3. Zhang S, Fu Q, Wang S, et al. Association between air pollution and the prevalence of allergic rhinitis in Chinese children: A systematic review and meta-analysis. Allergy Asthma Proc. 2022;43(5):e47-e57. doi:10.2500/aap.2022.43.220044.
4. Rosario Filho NA, Satoris RA, Scala WR. Allergic rhinitis aggravated by air pollutants in Latin America: A systematic review. World Allergy Organ J.

2021;14(8):100574. Published 2021 Aug 20. doi:10.1016/j.waojou.2021.100574.
5. Takkouche B, González-Barcala FJ, Etminan M, Fitzgerald M. Exposure to furry pets and the risk of asthma and allergic rhinitis: a meta-analysis. Allergy. 2008;63(7):857-864. doi:10.1111/j.1398-9995.2008.01732.x.
6. Duong QA, Pittet LF, Curtis N, Zimmermann P. Antibiotic exposure and adverse long-term health outcomes in children: A systematic review and meta-analysis [published correction appears in J Infect. 2023 Jan;86(1):118]. J Infect. 2022;85(3):213-300. doi:10.1016/j.jinf.2022.01.005.
7. Malmir H, Larijani B, Esmaillzadeh A. Fish consumption during pregnancy and risk of allergic diseases in the offspring: A systematic review and meta-analysis. Crit Rev Food Sci Nutr. 2022;62(27):7449-7459. doi:10.1080/10408398.2021.1914543.
8. Zhou Y, Chen J, Dong Y, et al. Maternal tobacco exposure during pregnancy and allergic rhinitis in offspring: A systematic review and meta-analysis. Medicine (Baltimore). 2021;100(34):e26986. doi:10.1097/MD.0000000000026986.
9. Lin L, Li T, Sun M, et al. Effect of particulate matter exposure on the prevalence of allergic rhinitis in children: A systematic review and meta-analysis. Chemosphere. 2021;268:128841. doi:10.1016/j.chemosphere.2020.128841.
10. Luo H, Zhang Q, Niu Y, Kan H, Chen R. Fine particulate matter and cardiorespiratory health in China: A systematic review and meta-analysis of epidemiological studies. J Environ Sci (China). 2023;123:306-316. doi:10.1016/j.jes.2022.04.026.
11. Ye T, Yu P, Wen B, et al. Greenspace and health outcomes in children and adolescents: A systematic review. Environ Pollut. 2022;314:120193. doi:10.1016/j.envpol.2022.120193.
12. Lambert KA, Bowatte G, Tham R, et al. Residential greenness and allergic respiratory diseases in children and adolescents - A systematic review and meta-analysis. Environ Res. 2017;159:212-221. doi:10.1016/j.envres.2017.08.002.
13. Wu B, Guo X, Liang M, et al. Association of individual green space exposure with the incidence of asthma and allergic rhinitis: a systematic review and meta-analysis. Environ Sci Pollut Res Int. 2022;29(59):88461-88487. doi:10.1007/s11356-022-23718-x.
14. Mendell MJ, Mirer AG, Cheung K, Tong M, Douwes J. Respiratory and allergic health effects of dampness, mold, and dampness-related agents: a review of the epidemiologic evidence. Environ Health Perspect. 2011;119(6):748-756. doi:10.1289/ehp.1002410.
15. Tischer C, Chen CM, Heinrich J. Association between domestic mould and mould components, and asthma and allergy in children: a systematic review. Eur Respir J. 2011;38(4):812-824. doi:10.1183/09031936.00184010.
16. Lodge CJ, Tan DJ, Lau MX, et al. Breastfeeding and asthma and allergies: a systematic review and meta-analysis. Acta Paediatr. 2015;104(467):38-53. doi:10.1111/apa.13132.
17. Mimouni Bloch A, Mimouni D, Mimouni M, Gdalevich M. Does breastfeeding protect against allergic rhinitis during childhood? A meta-analysis of prospective studies. Acta Paediatr. 2002;91(3):275-279. doi:10.1080/08035250252833914.
18. Kim YH, Kim KW, Kim MJ, et al. Vitamin D levels in allergic rhinitis: a systematic review and meta-analysis. Pediatr Allergy Immunol. 2016;27(6):580-590. doi:10.1111/pai.12599.
19. Das RR, Naik SS. Neonatal hyperbilirubinemia and childhood allergic diseases: a systematic review. Pediatr Allergy Immunol. 2015;26(1):2-11. doi:10.1111/pai.12281.
20. Tang L, Chen Y, Xiang Q, Xiang J, Tang Y, Li J. The association between IL18, FOXP3 and IL13 genes polymorphisms and risk of allergic rhinitis: a meta-analysis. Inflamm Res. 2020;69(9):911-923. doi:10.1007/s00011-020-01368-4.
21. Bunyavanich S, Shargorodsky J, Celedón JC. A meta-analysis of Th2 pathway genetic variants and risk for allergic rhinitis. Pediatr Allergy Immunol. 2011;22(4):378-387. doi:10.1111/j.1399-3038.2010.01124.x.
22. Ying XJ, Zhao SW, Wang GL, Xie J, Xu HM, Dong P. Association of interleukin-13 SNP rs20541 with allergic rhinitis risk: a meta-analysis. Gene. 2013;521(2):222-226. doi:10.1016/j.gene.2013.03.088.
23. Ying X, Zhang R, Yu S, Wu J, Wang H. Association of interleukin-13 SNP rs1800925 with allergic rhinitis risk: a meta-analysis based on 1,411 cases and 3169 controls. Gene. 2012;506(1):179-183. doi:10.1016/j.gene.2012.06.066.
24. Li P, Cao L, Han X. Angiotensin-converting enzyme (ACE) I/D polymorphism is a risk factor of allergic rhinitis. Cell Mol Biol (Noisy-le-grand). 2017;63(8):48-50. Published 2017 Aug 30. doi:10.14715/cmb/2017.63.8.11.
25. Guo M, Ma J, Han Y, Lu L. Angiotensin-converting enzyme gene insertion/deletion polymorphisms and the susceptibility to allergic rhinitis. Allergol Immunopathol (Madr). 2014;42(6):568-572. doi:10.1016/j.aller.2013.09.008.
26. Huang RF, Dong P, Zhang TZ, Ying XJ, Hu H. Angiotensin-converting enzyme insertion/deletion polymorphism and susceptibility to allergic rhinitis in Chinese populations: a systematic review and meta-analysis. Eur Arch Otorhinolaryngol. 2016;273(2):277-283. doi:10.1007/s00405-014-3350-6.
27. Tang L, Chen Y, Xiang Q, Xiang J, Tang Y, Li J. The association between IL18, FOXP3 and IL13 genes polymorphisms and risk of allergic rhinitis: a meta-analysis. Inflamm Res. 2020;69(9):911-923. doi:10.1007/s00011-020-01368-4.
28. Bunyavanich S, Shargorodsky J, Celedón JC. A meta-analysis of Th2 pathway genetic variants and risk for allergic rhinitis. Pediatr Allergy Immunol. 2011;22(4):378-387. doi:10.1111/j.1399-3038.2010.01124.x.
29. Ying XJ, Zhao SW, Wang GL, Xie J, Xu HM, Dong P. Association of interleukin-13 SNP rs20541 with allergic rhinitis risk: a meta-analysis. Gene. 2013;521(2):222-226. doi:10.1016/j.gene.2013.03.088.
30. Ying X, Zhang R, Yu S, Wu J, Wang H. Association of interleukin-13 SNP rs1800925 with allergic rhinitis risk: a meta-analysis based on 1,411 cases and 3169 controls. Gene. 2012;506(1):179-183. doi:10.1016/j.gene.2012.06.066.
31. Li P, Cao L, Han X. Angiotensin-converting enzyme (ACE) I/D polymorphism is a risk factor of allergic rhinitis. Cell Mol Biol (Noisy-le-grand). 2017;63(8):48-50. Published 2017 Aug 30. doi:10.14715/cmb/2017.63.8.11.
32. Guo M, Ma J, Han Y, Lu L. Angiotensin-converting enzyme gene insertion/deletion polymorphisms and the susceptibility to allergic rhinitis. Allergol Immunopathol (Madr). 2014;42(6):568-572. doi:10.1016/j.aller.2013.09.008.
33. Huang RF, Dong P, Zhang TZ, Ying XJ, Hu H. Angiotensin-converting enzyme insertion/deletion polymorphism and susceptibility to allergic rhinitis in Chinese populations: a systematic review and meta-analysis. Eur Arch Otorhinolaryngol. 2016;273(2):277-283. doi:10.1007/s00405-014-3350-6.
34. Wang B, Wu Z, Wang F, Yin Z, Shi L, Liu Y. Nasal nitric oxide testing for allergic rhinitis patients: Systematic review and meta-analysis. Immun Inflamm Dis. 2021;9(3):635-648. doi:10.1002/iid3.439.
35. Lødrup Carlsen KC, Roll S, Carlsen KH, et al. Does pet ownership in infancy lead to asthma or allergy at school age? Pooled analysis of individual participant data from 11 European birth cohorts. PLoS One. 2012;7(8):e43214. doi:10.1371/journal.pone.0043214.
36. Burte E, Leynaert B, Bono R, et al. Association between air pollution and rhinitis incidence in two European cohorts. Environ Int. 2018;115:257-266. doi:10.1016/j.envint.2018.03.021.
37. Kanazawa J, Masuko H, Yatagai Y, et al. Association analyses of eQTLs of the TYRO3 gene and allergic diseases in Japanese populations. Allergol Int. 2019;68(1):77-81. doi:10.1016/j.alit.2018.07.004.
38. Venter C, Agostoni C, Arshad SH, et al. Dietary factors during pregnancy and atopic outcomes in childhood: A systematic review from the European Academy of Allergy and Clinical Immunology. Pediatr Allergy Immunol. 2020;31(8):889-912. doi:10.1111/pai.13303.
39. Fuertes E, Sunyer J, Gehring U, et al. Associations between air pollution and pediatric eczema, rhinoconjunctivitis and asthma: A meta-analysis of European birth cohorts. Environ Int. 2020;136:105474. doi:10.1016/j.envint.2020.105474.
40. Stratakis N, Roumeliotaki T, Oken E, et al. Fish and seafood consumption during pregnancy and the risk of asthma and allergic rhinitis in childhood: a pooled analysis of 18 European and US birth cohorts. Int J Epidemiol. 2017;46(5):1465-1477. doi:10.1093/ije/dyx007.
41. Tischer CG, Gref A, Standl M, et al. Glutathione-S-transferase P1, early exposure to mould in relation to respiratory and allergic health outcomes in children from six birth cohorts. A meta-analysis. Allergy. 2013;68(3):339-346. doi:10.1111/all.12093.
42. Savouré M, Lequy É, Bousquet J, et al. Long-term exposures to PM2.5, black carbon and NO2 and prevalence of current rhinitis in French adults: The Constances Cohort. Environ Int. 2021;157:106839. doi:10.1016/j.envint.2021.106839.
43. Tareke AA, Hadgu AA, Ayana AM, Zerfu TA. Prenatal vitamin D supplementation and child respiratory health: A systematic review and meta-analysis of randomized controlled trials. World Allergy Organ J. 2020;13(12):100486. Published 2020 Nov 21. doi:10.1016/j.waojou.2020.100486.
44. Yepes-Nuñez JJ, Brożek JL, Fiocchi A, et al. Vitamin D supplementation in primary allergy prevention: Systematic review of randomized and non-randomized studies. Allergy. 2018;73(1):37-49. doi:10.1111/all.13241.
45. Luo C, Sun Y, Zeng Z, Liu Y, Peng S. Vitamin D supplementation in pregnant women or infants for preventing allergic diseases: a systematic review and meta-analysis of randomized controlled trials. Chin Med J (Engl). 2022;135(3):276-284. Published 2022 Jan 12. doi:10.1097/CM9.0000000000001951.
46. Li Q, Zhou Q, Zhang G, et al. Vitamin D Supplementation and Allergic Diseases during Childhood: A Systematic Review and Meta-Analysis. Nutrients. 2022;14(19):3947. Published 2022 Sep 23. doi:10.3390/nu14193947.
47. Tischer C, Dadvand P, Basagana X, et al. Urban upbringing and childhood respiratory and allergic conditions: A multi-country holistic study. Environ Res. 2018;161:276-283. doi:10.1016/j.envres.2017.11.013.

48. Venter C, Palumbo MP, Glueck DH, et al. The maternal diet index in pregnancy is associated with offspring allergic diseases: the Healthy Start study. Allergy. 2022;77(1):162-172. doi:10.1111/all.14949.
49. Fuertes E, Markevych I, Bowatte G, et al. Residential greenness is differentially associated with childhood allergic rhinitis and aeroallergen sensitization in seven birth cohorts. Allergy. 2016;71(10):1461-1471. doi:10.1111/all.12915.
50. Schindler T, Sinn JK, Osborn DA. Polyunsaturated fatty acid supplementation in infancy for the prevention of allergy. Cochrane Database Syst Rev. 2016;10(10):CD010112. Published 2016 Oct 28. doi:10.1002/14651858.CD010112.pub2.
51. Idrose NS, Walters EH, Zhang J, et al. Outdoor pollen-related changes in lung function and markers of airway inflammation: A systematic review and meta-analysis. Clin Exp Allergy. 2021;51(5):636-653. doi:10.1111/cea.13842.
52. Best KP, Gold M, Kennedy D, Martin J, Makrides M. Omega-3 long-chain PUFA intake during pregnancy and allergic disease outcomes in the offspring: a systematic review and meta-analysis of observational studies and randomized controlled trials. Am J Clin Nutr. 2016;103(1):128-143. doi:10.3945/ajcn.115.111104.
53. Bønnelykke K, Matheson MC, Pers TH, et al. Meta-analysis of genome-wide association studies identifies ten loci influencing allergic sensitization. Nat Genet. 2013;45(8):902-906. doi:10.1038/ng.2694.
54. Eller E, Roll S, Chen CM, et al. Meta-analysis of determinants for pet ownership in 12 European birth cohorts on asthma and allergies: a GA2LEN initiative. Allergy. 2008;63(11):1491-1498. doi:10.1111/j.1398-9995.2008.01790.x.
55. Hamizan AW, Rimmer J, Husain S, et al. Local specific Immunoglobulin E among patients with nonallergic rhinitis: a systematic review. Rhinology. 2019;57(1):10-20. doi:10.4193/Rhin18.074.
56. Bunyavanich S, Schadt EE, Himes BE, et al. Integrated genome-wide association, coexpression network, and expression single nucleotide polymorphism analysis identifies novel pathway in allergic rhinitis. BMC Med Genomics. 2014;7:48. Published 2014 Aug 2. doi:10.1186/1755-8794-7-48.
57. Shiue I. Indoor mildew odour in old housing was associated with adult allergic symptoms, asthma, chronic bronchitis, vision, sleep and self-rated health: USA NHANES, 2005-2006. Environ Sci Pollut Res Int. 2015;22(18):14234-14240. doi:10.1007/s11356-015-4671-8.
58. Shaoqing Y, Ruxin Z, Yingjian C, Jianqiu C, Yanshen W, Genhong L. A meta-analysis of the association of exhaled carbon monoxide on asthma and allergic rhinitis. Clin Rev Allergy Immunol. 2011;41(1):67-75. doi:10.1007/s12016-009-8195-1.
59. Waage J, Standl M, Curtin JA, et al. Genome-wide association and HLA fine-mapping studies identify risk loci and genetic pathways underlying allergic rhinitis [published correction appears in Nat Genet. 2018 Sep;50(9):1343]. Nat Genet. 2018;50(8):1072-1080. doi:10.1038/s41588-018-0157-1.
60. Tischer CG, Hohmann C, Thiering E, et al. Meta-analysis of mould and dampness exposure on asthma and allergy in eight European birth cohorts: an ENRIECO initiative. Allergy. 2011;66(12):1570-1579. doi:10.1111/j.1398-9995.2011.02712.x.
61. Testa D, DI Bari M, Nunziata M, et al. Allergic rhinitis and asthma assessment of risk factors in pediatric patients: A systematic review. Int J Pediatr Otorhinolaryngol. 2020;129:109759. doi:10.1016/j.ijporl.2019.109759.
62. Patelarou E, Giourgouli G, Lykeridou A, et al. Association between biomarker-quantified antioxidant status during pregnancy and infancy and allergic disease during early childhood: a systematic review. Nutr Rev. 2011;69(11):627-641. doi:10.1111/j.1753-4887.2011.00445.x.
63. Conlan N, Maher GM, Al Khalaf SY, McCarthy FP, Khashan AS. Association between hypertensive disorders of pregnancy and the risk of asthma, eczema and allergies in offspring: A systematic review and meta-analysis. Clin Exp Allergy. 2021;51(1):29-38. doi:10.1111/cea.13754.
64. Kremmyda LS, Vlachava M, Noakes PS, Diaper ND, Miles EA, Calder PC. Atopy risk in infants and children in relation to early exposure to fish, oily fish, or long-chain omega-3 fatty acids: a systematic review. Clin Rev Allergy Immunol. 2011;41(1):36-66. doi:10.1007/s12016-009-8186-2.
65. Obbagy JE, English LK, Wong YP, et al. Complementary feeding and food allergy, atopic dermatitis/eczema, asthma, and allergic rhinitis: a systematic review. Am J Clin Nutr. 2019;109(Suppl_7):890S-934S. doi:10.1093/ajcn/nqy220.
66. Farronato M, Lanteri V, Fama A, Maspero C. Correlation between Malocclusion and Allergic Rhinitis in Pediatric Patients: A Systematic Review. Children (Basel). 2020;7(12):260. Published 2020 Nov 27. doi:10.3390/children7120260.
67. Güngör D, Nadaud P, Dreibelbis C, et al. Feeding a Lower Versus Higher Intensity, Proportion, or Amount of Human Milk to Mixed-Fed Infants and Food Allergies, Allergic Rhinitis, Atopic Dermatitis, and Asthma: A Systematic Review. Alexandria (VA): USDA Nutrition Evidence Systematic Review; April 2019.
68. Yang HJ. Impact of perinatal environmental tobacco smoke on the development of childhood allergic diseases. Korean J Pediatr. 2016;59(8):319-327. doi:10.3345/kjp.2016.59.8.319.
69. Caillaud D, Leynaert B, Keirsbulck M, Nadif R; mould ANSES working group. Indoor mould exposure, asthma and rhinitis: findings from systematic reviews and recent longitudinal studies. Eur Respir Rev. 2018;27(148):170137. Published 2018 May 15. doi:10.1183/16000617.0137-2017.
70. Güngör D, Nadaud P, LaPergola CC, et al. Infant milk-feeding practices and food allergies, allergic rhinitis, atopic dermatitis, and asthma throughout the life span: a systematic review [published correction appears in Am J Clin Nutr. 2019 Oct 1;110(4):1041]. Am J Clin Nutr. 2019;109(Suppl_7):772S-799S. doi:10.1093/ajcn/nqy283.
71. Tamasauskiene L, Sitkauskiene B. Interleukin-22 in Allergic Airway Diseases: A Systematic Review. J Interferon Cytokine Res. 2020;40(3):125-130. doi:10.1089/jir.2019.0094.
72. Huang PY, Huang YH, Guo MM, Chang LS, Kuo HC. Kawasaki Disease and Allergic Diseases. Front Pediatr. 2021;8:614386. Published 2021 Jan 7. doi:10.3389/fped.2020.614386.
73. Donovan S, Dewey K, Novotny R, et al. Maternal Diet during Pregnancy and Lactation and Risk of Child Food Allergies and Atopic Allergic Diseases: A Systematic Review. Alexandria (VA): USDA Nutrition Evidence Systematic Review; July 2020.
74. Wang J. Meta-Analysis of the Association Study between Allergic Rhinitis and HLA-II Gene (DQB1) in Northern China. J Healthc Eng. 2021;2021:4356770. Published 2021 Sep 23. doi:10.1155/2021/4356770.
75. Güngör D, Nadaud P, Dreibelbis C, et al. Never Versus Ever Feeding Human Milk and Food Allergies, Allergic Rhinitis, Atopic Dermatitis, and Asthma: A Systematic Review. Alexandria (VA): USDA Nutrition Evidence Systematic Review; April 2019..
76. Andersson NW, Hansen MV, Larsen AD, Hougaard KS, Kolstad HA, Schlünssen V. Prenatal maternal stress and atopic diseases in the child: a systematic review of observational human studies. Allergy. 2016;71(1):15-26. doi:10.1111/all.12762.
77. Hofmann MA, Fluhr JW, Ruwwe-Glösenkamp C, Stevanovic K, Bergmann KC, Zuberbier T. Role of IL-17 in atopy-A systematic review. Clin Transl Allergy. 2021;11(6):e12047. Published 2021 Aug 13. doi:10.1002/clt2.12047.
78. Güngör D, Nadaud P, Dreibelbis C, et al. Shorter Versus Longer Durations of Any Human Milk Feeding and Food Allergies, Allergic Rhinitis, Atopic Dermatitis, and Asthma: A Systematic Review. Alexandria (VA): USDA Nutrition Evidence Systematic Review; April 2019.
79. Güngör D, Nadaud P, Dreibelbis C, et al. Shorter Versus Longer Durations of Exclusive Human Milk Feeding Prior to the Introduction of Infant Formula and Food Allergies, Allergic Rhinitis, Atopic Dermatitis, and Asthma: A Systematic Review. Alexandria (VA): USDA Nutrition Evidence Systematic Review; April 2019.
80. Tarini BA, Carroll AE, Sox CM, Christakis DA. Systematic review of the relationship between early introduction of solid foods to infants and the development of allergic disease. Arch Pediatr Adolesc Med. 2006;160(5):502-507. doi:10.1001/archpedi.160.5.502.
81. Lin SY, Melvin TA, Boss EF, Ishman SL. The association between allergic rhinitis and sleep-disordered breathing in children: a systematic review. Int Forum Allergy Rhinol. 2013;3(6):504-509. doi:10.1002/alr.21123.
82. Gupta A, Singh A, Fernando RL, Dharmage SC, Lodge CJ, Waidyatillake NT. The association between sugar intake during pregnancy and allergies in offspring: a systematic review and a meta-analysis of cohort studies. Nutr Rev. 2022;80(4):904-918. doi:10.1093/nutrit/nuab052.
83. Hur K, Liang J, Lin SY. The role of secondhand smoke in allergic rhinitis: a systematic review. Int Forum Allergy Rhinol. 2014;4(2):110-116. doi:10.1002/alr.21246.
84. Obbagy JE, English LK, Psota TL, et al. Timing of Introduction of Complementary Foods and Beverages and Food Allergy, Atopic Dermatitis/Eczema, Asthma, and Allergic Rhinitis: A Systematic Review. Alexandria (VA): USDA Nutrition Evidence Systematic Review; April 2019.
85. Khaleva E, Gridneva Z, Geddes DT, et al. Transforming growth factor beta in human milk and allergic outcomes in children: A systematic review. Clin Exp Allergy. 2019;49(9):1201-1213. doi:10.1111/cea.13409.
86. Hu SJ, Wei P, Kou W, et al. Lin Chung Er Bi Yan Hou Tou Jing Wai Ke Za Zhi. 2017;31(19):1485-1491. doi:10.13201/j.issn.1001-1781.2017.19.006.
87. Feng H, Chen Y, Xiong X, et al. Association of nutrients intake during pregnancy with the risk of allergic disease in offspring: a meta-analysis of prospective cohort studies[J]. Food Science and Human Wellness, 2023, 12(3): 711-719. http://doi.org/10.1016/j.fshw.2022.09.004.
88. Pang K, Li G, Li M, et al. Prevalence and Risk Factors for Allergic Rhinitis in China: A Systematic Review and Meta-Analysis. Evid Based Complement Alternat Med. 2022;2022:7165627. Published 2022 Sep 23. doi:10.1155/2022/7165627.
89. Xu Y, Wang J. Association of CD14 gene -159C/T polymorphism with allergic rhinitis risk: a meta-analysis. Eur Arch Otorhinolaryngol. 2014;271(6):1601-1607. doi:10.1007/s00405-013-2793-5.

90. Guo H, Peng T, Luo P, et al. Association of FcεRIβ polymorphisms with risk of asthma and allergic rhinitis: evidence based on 29 case-control studies [retracted in: Biosci Rep. 2022 Sep 30;42(9):]. Biosci Rep. 2018;38(4):BSR20180177. Published 2018 Jul 31. doi:10.1042/BSR20180177.
91. Zhang G, Zhang D, Shi W, Sun P, Lin P. The Impact of FOXP3 Polymorphism on the Risk of Allergic Rhinitis: A Meta-Analysis. Ann Hum Genet. 2017;81(6):284-291. doi:10.1111/ahg.12205.
92. Yang Y, Xiao J, Tang L, et al. Effects of IL-6 Polymorphisms on Individual Susceptibility to Allergic Diseases: A Systematic Review and Meta-Analysis. Front Genet. 2022;13:822091. Published 2022 Mar 16. doi:10.3389/fgene.2022.822091.
93. Zheng L, Li X, Song Q, Hou C, Chen X, Li B. PAI-1 Gene Polymorphism Was Associated with an Increased Risk of Allergic Diseases: Evidence from a Meta-Analysis of 14 Case-Control Studies. Int Arch Allergy Immunol. 2019;180(4):255-263. doi:10.1159/000502522.
94. Liu R, Chen X, Qi J. Associations of TAP1 genetic polymorphisms with atopic diseases: asthma, rhinitis and dermatitis. Oncotarget. 2017;9(2):1553-1562. Published 2017 Dec 20. doi:10.18632/oncotarget.23458.
95. Zhang L, Zhang S, He C, Wang X. VDR Gene Polymorphisms and Allergic Diseases: Evidence from a Meta-analysis. Immunol Invest. 2020;49(1-2):166-177. doi:10.1080/08820139.2019.1674325.
96. Tharabenjasin P, Pabalan N, Jarjanazi H, Poachanukoon O. Influence of Polymorphisms in the Interleukin-18 Gene on Allergic Rhinitis: A Meta-Analysis. Int Arch Allergy Immunol. 2020;181(5):375-384. doi:10.1159/000506010.
97. Wongtrakul W, Charoenngam N, Ponvilawan B, Ungprasert P. Allergic rhinitis and risk of systemic lupus erythematosus: A systematic review and meta-analysis. Int J Rheum Dis. 2020;23(11):1460-1467. doi:10.1111/1756-185X.13928.
98. Rodrigues J, Franco-Pego F, Sousa-Pinto B, Bousquet J, Raemdonck K, Vaz R. Anxiety and depression risk in patients with allergic rhinitis: a systematic review and meta-analysis. Rhinology. 2021;59(4):360-373. doi:10.4193/Rhin21.087.
99. Zhu J, Song J, Liu Z, et al. Association between allergic conditions and risk of prostate cancer: A Prisma-Compliant Systematic Review and Meta-Analysis. Sci Rep. 2016;6:35682. Published 2016 Oct 21. doi:10.1038/srep35682.
100. Chen Z, Xing Y, Yu X, Dou Y, Ma D. Effect of Folic Acid Intake on Infant and Child Allergic Diseases: Systematic Review and Meta-Analysis. Front Pediatr. 2021;8:615406. Published 2021 Jan 18. doi:10.3389/fped.2020.615406.
101. Campbell BE, Lodge CJ, Lowe AJ, Burgess JA, Matheson MC, Dharmage SC. Exposure to 'farming' and objective markers of atopy: a systematic review and meta-analysis. Clin Exp Allergy. 2015;45(4):744-757. doi:10.1111/cea.12429.
102. Arroyave WD, Rabito FA, Carlson JC, Friedman EE, Stinebaugh SJ. Impermeable dust mite covers in the primary and tertiary prevention of allergic disease: a meta-analysis. Ann Allergy Asthma Immunol. 2014;112(3):237-248. doi:10.1016/j.anai.2014.01.006.
103. He G, Zou X, Chen X, et al. Meta-analysis of the association between five single nucleotide polymorphisms in the BDNF gene and allergic inflammation susceptibility[J]. Genes & Genomics, 2017, 39: 747-758. doi:10.1007/s13258-017-0538-3.
104. Heinrich J. Modulation of allergy risk by breast feeding. Curr Opin Clin Nutr Metab Care. 2017;20(3):217-221. doi:10.1097/MCO.0000000000000366.
105. Kitinoja MA, Hugg TT, Siddika N, Rodriguez Yanez D, Jaakkola MS, Jaakkola JJK. Short-term exposure to pollen and the risk of allergic and asthmatic manifestations: a systematic review and meta-analysis. BMJ Open. 2020;10(1):e029069. Published 2020 Jan 10. doi:10.1136/bmjopen-2019-029069.
106. Liu J, Zhang X, Zhao Y, Wang Y. The association between allergic rhinitis and sleep: A systematic review and meta-analysis of observational studies. PLoS One. 2020;15(2):e0228533. Published 2020 Feb 13. doi:10.1371/journal.pone.0228533.
107. Baron R, Taye M, der Vaart IB, et al. The relationship of prenatal antibiotic exposure and infant antibiotic administration with childhood allergies: a systematic review. BMC Pediatr. 2020;20(1):312. Published 2020 Jun 27. doi:10.1186/s12887-020-02042-8.
108. Knudgaard MH, Andreasen TH, Ravnborg N, et al. Rhinitis prevalence and association with atopic dermatitis: A systematic review and meta-analysis. Ann Allergy Asthma Immunol. 2021;127(1):49-56.e1. doi:10.1016/j.anai.2021.02.026.
109. Charoenngam N, Ponvilawan B, Rittiphairoj T, et al. The association between allergic rhinitis and risk of rheumatoid arthritis: A systematic review and meta-analysis. J Evid Based Med. 2021;14(1):27-39. doi:10.1111/jebm.12393.
110. Liu L, Luo C, Zhang M, Ao X, Liu H, Peng S. Relationship between allergic diseases and mental disorders in women: A systematic review and meta-analysis. Front Psychiatry. 2022;13:1026032. Published 2022 Nov 9. doi:10.3389/fpsyt.2022.1026032.
111. Schans JV, Çiçek R, de Vries TW, Hak E, Hoekstra PJ. Association of atopic diseases and attention-deficit/hyperactivity disorder: A systematic review and meta-analyses. Neurosci Biobehav Rev. 2017;74(Pt A):139-148. doi:10.1016/j.neubiorev.2017.01.011.

Subset analysis of potential environmental risk/protective factors for AR in children

Exposure	Source	Number of cases/ total population	Number of study	Study design	Effect metrics	Random effects summary estimate (95% CI)	Random effects p value	I^2	95 % prediction interval	Egger p value	Large heterogeneity, small study effect, loss of significance under 10 % credibility ceiling, or evidential value not found under p-curve analysis	Level of evidence
**Convincing (class I)**												
Tic disorders	Huang 2022	>1000/56696	5	Case-control	OR	2.82 (2.55–3.11)	1.64e-93	38%	(2.4–3.31)	0.82	None	Convincing retained
Exposure to indoor dampness	Maritta 2013	>1000/25730	7	Cohort, Cross-sectional	OR	1.56 (1.39–1.75)	3.01e-13	17%	(1.28–1.91)	0.27	None	Highly suggestive to convincing
Exposure to indoor mold	Maritta 2013	>1000/31961	10	Cohort, Cross-sectional	OR	1.50 (1.39–1.62)	1.47e-25	0%	(1.38–1.64)	0.21	None	Suggestive to convincing
**Highly suggestive (class II)**												
Early-life antibiotic use	Liu 2022	>1000/1622594	13	Cohort, Cross-sectional	OR	3.51 (3.18–3.89)	4.99e-147	62%	(2.69–4.69)	0.20	Large heterogeneity	Highly suggestive retained
Farm milk consumption	Brick 2020	>1000/64685	6	Cohort, Cross-sectional	OR	0.68 (0.57–0.82)	0.000043	0%	(0.56–0.84)	0.40	None	Highly suggestive retained
Prolonged breastfeeding	Hoang 2021	>1000/161611	20	Cohort, Cross-sectional	OR	0.72 (0.65–0.79)	2.32E-12	72%	(0.51–1.01)	0.06	Large heterogeneity, small study effect, excess significance bias, 95% prediction interval includes the null value	Highly suggestive retained
Acetaminophen exposure	Zeng 2020	>1000/857083	17	Cohort, Case-control, Cross-sectional	OR	1.55 (1.41–1.70)	9.78e-12	95%	(0.76–3.38)	0.47	Large heterogeneity, 95% prediction interval includes the null value	Highly suggestive retained
Childhood acid suppressant use	Muhammad 2022	>1000/950640	2	Cohort	HR	1.40 (1.23–1.59)	9.01e-08	97%	NA	NA	Large heterogeneity	Highly suggestive retained
Active exposure to tobacco smoking	Saulyte 2014	>1000/79556	10	Cohort, Case-control, Cross-sectional	RR	1.41 (1.24–1.59)	9.57e-08	86%	(0.92–2.15)	0.61	Large heterogeneity, 95% prediction interval includes the null value	Not significant to highly suggestive
**Suggestive (class III)**												
Passive exposure to tobacco smoking	Saulyte 2014	>1000/931571	50	Cohort, Case-control, Cross-sectional	RR	1.09 (1.04–1.14)	0.00234	85%	(0.78–1.56)	0.77	Large heterogeneity, 95% prediction interval includes the null value	Suggestive retained
Early dietary introduction of fish	Ierodiakonou 2016	>1000/13423	4	Cohort	OR	0.64 (0.50–0.82)	0.000425	38%	(0.26–1.56)	0.06	Small study effect, excess significance bias, 95% prediction interval includes the null value	Suggestive retained
History of Kawasaki disease	Lei 2021	>1000/NR	4	Case-control, Cross-sectional	OR	1.73 (1.29–2.31)	0.000372	89%	(0.46–6.49)	0.20	Large heterogeneity, excess significance bias, 95% prediction interval includes the null value	Suggestive retained
**Weak (class IV)**												

Prenatal smoke exposure	Li 2022	>1000/23319	6	Cohort	OR	1.12 (1.04–1.21)	0.0223	0%	(0.95–1.31)	0.12	95% prediction interval includes the null value	Weak retained
Early life food sensitization	Alduraywish 2016	<1000/4386	4	Cohort	OR	3.06 (1.90–4.94)	0.000007	38%	(0.55–17.04)	0.86	95% prediction interval includes the null value	Weak retained
Postpartum smoke exposure	Li 2022	>1000/144325	8	Cohort	OR	1.19 (1.03–1.39)	0.0279	77%	(0.71–2.05)	0.98	Large heterogeneity, 95% prediction interval includes the null value	Weak retained
Prenatal maternal psychosocial stress	Flanigan 2018	<1000/7685	3	Cohort, Cross-sectional	OR	1.36 (1.08–1.71)	0.00991	43%	(0.69–2.70)	0.50	Excess significance bias, 95% prediction interval includes the null value	Weak retained
Maternal oral contraceptive pill	Bai 2020	<1000/5386	5	Cohort, Case-control, Cross-sectional	OR	1.34 (1.07–1.68)	0.00991	38%	(0.69–2.70)	0.50	Excess significance bias, 95% prediction interval includes the null value	Weak retained
Obstructive sleep apnea	Cao 2018	>1000/NR	11	Cohort, Cross-sectional	OR	1.61 (1.15–2.26)	0.00362	62%	(0.65–4.01)	0.69	Large heterogeneity, 95% prediction interval includes the null value	Weak retained
**Not significant (NS)**												
Vitamin D status	Aryan 2017	>1000/25965	5	Cohort, Cross-sectional	OR	1.06 (0.77–1.47)	0.701	72%	(0.34–3.37)	0.78	Large heterogeneity, 95% prediction interval includes the null value	Weak to not significant
Greenness	Cao 2022	>1000/50993	10	Cohort, Cross-sectional	OR	1.00 (0.99–1.00)	0.07	53%	(0.99–1.00)	0.11	Excess significance bias, 95% prediction interval includes the null value	Not significant retained
Maternal fish intake during pregnancy	Zhang 2016	>1000/21042	3	Cohort	OR	0.94 (0.60–1.64)	0.769	45%	(0.17–5.56)	0.58	95% prediction interval includes the null value	Not significant retained
Prenatal exposure to vitamin D	Pacheco 2018	>1000/26147	6	Cohort	OR	0.99 (0.84–1.16)	0.843	0%	(0.79–1.23)	0.17	95% prediction interval includes the null value	Not significant retained
Neonatal jaundice	Kuniyoshi 2021	>1000/12213	3	Cohort, Case-control	OR	3.01 (0.88–10.28)	0.0629	93%	(0–5909514)	0.46	Large heterogeneity, 95% prediction interval includes the null value	Not significant retained
Phototherapy	Kuniyoshi 2021	>1000/83060	2	Cohort	OR	1.38 (0.93–2.04)	0.111	56%	NA	NA	Large heterogeneity	Not significant retained
Childhood type 1 diabetes	Cardwell 2003	>1000/72808	11	Case-control, Cross-sectional	OR	0.88 (0.68–1.13)	0.254	56%	(0.43–1.77)	0.89	Large heterogeneity, 95% prediction interval includes the null value	Not significant retained
Children and adolescents exposed to pesticides	Rodrigues 2021	<1000/3686	2	Cohort	OR	2.77 (0.13–61.40)	0.519	0%	NA	NA	None	Not significant retained
Exposure to perfluoroalkyl substances	Luo 2020	<1000/3396	4	Cohort, Cross-sectional	OR	1.08 (0.96–1.20)	0.168	37%	(0.81–1.43)	0.03	Small study effect, 95% prediction interval includes the null value	Not significant retained
Indoor microbial aerosols exposures	Fakunle 2021	<1000/2082	4	Cohort	RR	1.18 (0.93–1.49)	0.192	70%	(0.38–3.83)	0.24	Large heterogeneity, 95% prediction interval includes the null value	Not significant retained
Abbreviations: CI, confidence interval; NR, not reported; NA, not available; OR, odds ratio; RR, relative risk; HR, hazard ratio												

Supplementary analyses result of environmental risk and protective factors

Exposure	Source	Effect metrics	Effect estimate of the largest study (95% CI)	The largest p value in the meta-analysis	Excess significance bias (p > 0.1)	Whether the 95% PI includes the null value	Whether the 95% CI of the largest study includes the null
Tic disorders	Huang 2022	OR	2.82 (2.54–3.12)	6.46e-04	6.69e-01	Not null	Not null
Ambient particulate matter(PM_{10})	Li 2022	OR	1.02 (1.02–1.03)	6.45e-03	5.15e-09	Null	Not null
Sulfur dioxide	Li 2022	OR	1.14 (1.11–1.17)	8.98e-03	6.01e-01	Null	Not null
Nitrogen dioxide	Li 2022	OR	1.04 (1.03–1.04)	4.53e-05	5.26e-10	Null	Not null
Early-life antibiotic use	Liu 2022	OR	5.75 (5.58–5.93)	2.71e-86	4.58e-01	Not null	Not null
Farm milk consumption	Brick 2020	OR	0.63 (0.46–0.87)	1.25e-02	5.26e-01	Not null	Not null
Exposure to indoor dampness	Maritta 2013	OR	1.51 (1.31–1.74)	3.30e-06	2.35e-04	Null	Not null
Prolonged breastfeeding	Hoang 2021	OR	0.61 (0.46–0.81)	1.02e-10	4.59e-02	Null	Not null
Coronavirus disease 2019	Xu 2022	OR	0.30 (0.29–0.31)	2.12e-11	1.85e-01	Not null	Not null
Acetaminophen Exposure	Zeng 2020	OR	1.34 (1.28–1.40)	2.38e-13	5.36e-02	Null	Not null
Childhood acid suppressant use	Muhammad 2022	HR	1.49 (1.45–1.52)	4.04e-54	NA	NA	Not null
Ozone	Li 2022	OR	1.02 (1.02–1.03)	5.65e-02	2.12e-01	Null	Not null
Passive Exposure to Tobacco Smoking	Saulyte 2014	RR	1.10 (1.08–1.12)	4.41e-04	1.87e-02	Null	Not null
Early dietary introduction of fish	Ierodiakonou 2016	OR	0.49 (0.27–0.89)	6.81e-03	1.40e-05	Null	Not null
History of Kawasaki disease	Lei 2021	OR	1.28 (1.21–1.36)	1.49e-02	9.58e-05	Null	Not null
Educational level	Chong 2018	OR	1.42 (1.05 – 1.93)	1.38e-03	4.53e-02	Null	Not null
Family history of allergic diseases	Chong 2018	OR	1.30 (1.02–1.66)	1.52e-03	1.79e-01	Null	Not null

Exposure to indoor mold	Maritta 2013	OR	1.51 (1.31–1.74)	8.70e-08	1.00e+00	Null	Not null
Ambient particulate matter(PM2.5)	Li 2022	OR	1.02 (1.02–1.03)	6.80e-03	1.91e-06	Null	Not null
Carbon monoxide	Li 2022	OR	1.01 (1.00–1.01)	1.36e-01	3.59e-01	Null	Not null
Prenatal smoke exposure	Li 2022	OR	1.16 (1.06–1.26)	8.29e-01	5.78e-01	Null	Not null
Early life food sensitization	Alduraywish 2016	OR	2.20 (1.30–3.71)	1.70e-04	4.10e-01	Null	Not null
Postpartum smoke exposure	Li 2022	OR	1.20 (1.00–1.50)	7.80e-02	5.84e-01	Null	Null
Vitamin D status	Aryan 2017	OR	0.75 (0.63–0.89)	9.32e-02	4.50e-01	Null	Null
Obstructive sleep apnea	Cao 2018	OR	2.27 (1.78–2.90)	1.28e-02	4.51e-01	Null	Not null
Prenatal maternal psychosocial stress	Flanigan 2018	OR	1.75 (1.08–2.84)	6.14e-02	1.57e-02	Null	Null
Maternal oral contraceptive pill	Bai 2020	OR	1.50 (0.96–2.20)	6.70e-02	7.60e-01	Null	Null
Caesarean delivery	Bager 2008	OR	1.01 (0.85–1.21)	2.54e-02	6.62e-01	Null	Null
Attention deficit hyperactivity disorder	Miyazaki 2017	OR	2.22 (2.06–2.35)	3.02e-02	8.87e-01	Null	Not null
House dust mite	Chong 2018	OR	1.66(1.08–2.56)	2.14e-02	NA	NA	Not null
Exposure to cats	Gao 2020	RR	0.5 (0.2–1.4)	7.05e-01	1.22e-01	Null	Null
Exposure to dogs	Gao 2020	RR	0.8(0.4–1.6)	1.70e-01	8.06e-01	Null	Null
Greenness	Cao 2022	OR	1.00 (1.00–1.01)	1.04e-01	3.16e-03	Null	Null
Maternal fish intake during pregnancy	Zhang 2017	OR	0.92 (0.67–1.28)	9.77e-01	6.30e-01	Null	Null
Prenatal exposure to vitamin D	Pacheco 2018	OR	0.96 (0.79–1.16)	8.90e-01	6.64e-01	Null	Null
Neonatal jaundice	Kuniyoshi 2021	OR	1.46 (1.24–1.72)	1.71e-01	1.33e-01	Null	Not null
Phototherapy	Kuniyoshi 2021	OR	1.56 (1.48–1.64)	9.98e-01	NA	NA	Not null

Active Exposure to Tobacco Smoking	Saulyte 2014	RR	1.30 (1.23–1.36)	9.05e-01	4.90e-14	Null	Not null
Childhood Type 1 Diabetes	Cardwell 2003	OR	0.33 (0.05–2.42)	8.37e-01	8.74e-01	Null	Null
Children and adolescents exposed to pesticides	Rodrigues 2021	OR	4.85 (0.08–286.21)	9.08e-01	NA	NA	Null
Exposure to perfluoroalkyl substances	Luo 2020	OR	0.80 (0.44–1.45)	3.62e-01	5.36e-01	Null	Null
Multiple sclerosis	Monteiro 2011	OR	1.32(0.78–2.24)	5.06e-01	5.17e-01	Null	Null
Indoor microbial aerosols exposures	Fakunle 2021	RR	0.97 (0.80–1.18)	6.62e-01	4.98e-01	Null	Null
Abbreviations: PI, prediction interval; CI, confidence interval; NR, not reported; NA, not available; OR, odds ratio; RR, relative risk; HR, hazard ratio							

Potential biomarker of AR, with details of statistical test results

Potential biomarker	Source	Number of cases/ total population	Number of study	Study design	Effect metrics	Random effects summary estimate (95% CI)	Random effects p value	I^2	95 % prediction interval	Egger p value	Large heterogeneity, small study effect, loss of significance under 10 % credibility ceiling, or evidential value not found under p-curve analysis	AMSTAR 2
**Highly suggestive (class II)**												
Nasal nitric oxide	Ambrosino 2020[1]	>1000/3218	30	Case-control	SMD	1.31 (0.84–1.78)	2.61e-08	97%	(-1.55–4.15)	0.45	Large heterogeneity, 95% prediction interval includes the null value	Moderate
**Suggestive (class III)**												
IL-13 rs20541 polymorphism	Wang 2016[2]	>1000/8722	10	Case-control	OR	1.24 (1.09–1.41)	0.000020	41%	(1.09–1.34)	0.07	Small study effect, excess significance bias	Critically low
**Weak (class IV)**												
Serum IL-4 level	Li 2014[5]	<1000/915	14	Case-control	OR	2.41 (1.87–3.11)	0.000056	90%	(0.47–9.60)	7.26e-06	Large heterogeneity, small study effect, 95% prediction interval includes the null value	Critically low
ADAM33 S2 polymorphism	Li 2016[3]	>1000/2589	4	Case-control	OR	1.40 (1.08–1.81)	0.0128	70%	(0.64–4.24)	0.18	Large heterogeneity, excess significance bias	Low
ADAM33 V4 polymorphism	Li 2016[3]	>1000/2608	4	Case-control	OR	1.67 (1.02–2.74)	0.0117	93%	(0.07–38.87)	0.30	Large heterogeneity, 95% prediction interval includes the null value	Low
ADAM33 Q−1 polymorphism	Xu 2015[4]	<1000/1973	4	Case-control	OR	1.91 (1.11–3.29)	0.0219	74%	(0.18–20.96)	0.21	Large heterogeneity, 95% prediction interval includes the null value	Critically low
ACE I/D polymorphism	Lin 2014[11]	<1000/1410	6	Case-control	OR	1.46 (1.14–1.86)	0.00204	54%	(0.74–2.85)	0.32	Large heterogeneity, 95% prediction interval includes the null value	Low
TNF α rs1800629 polymorphism	Xiang 2022[7]	>1000/3816	5	Case-control	OR	1.56 (1.00–2.43)	0.0389	74%	(0.37–6.67)	0.69	Large heterogeneity, 95% prediction interval includes the null value	Critically low
CTLA-4 rs11571302 polymorphism	Xiang 2022[7]	>1000/46366	3	Case-control	OR	1.34 (1.09–1.64)	0.00582	45%	(0.16–10.75)	0.05	Small study effect, 95% prediction interval includes the null value	Critically low
IL-4R rs1801275 polymorphism	Xiang 2022[7]	>1000/3688	9	Case-control	OR	1.48 (1.07–2.04)	0.0209	65%	(0.52–4.30)	0.03	Large heterogeneity, small study effect, 95% prediction interval includes the null value	Critically low
**Not significant (NS)**												
ADAM33 T1 polymorphism	Xu 2015[4]	>1000/2700	6	Case-control	OR	1.52 (0.67–3.44)	0.409	94%	(0.04–53.92)	0.76	Large heterogeneity, 95% prediction interval includes the null value	Critically low
ADAM33 T2 polymorphism	Xu 2015[4]	>1000/2700	6	Case-control	OR	1.46 (0.68–3.16)	0.452	92%	(0.04–54.51)	0.52	Large heterogeneity, 95% prediction interval includes the null value	Critically low

IL-4 rs2243250 polymorphism	Jiang 2021[8]	>1000/3549	9	Case-control	OR	1.30 (0.95–1.77)	0.167	86%	(0.33–d5.04)	0.62	Large heterogeneity, 95% prediction interval includes the null value	Low
IL-4RA Ile50Val polymorphism	Xu 2014[6]	<1000/907	4	Case-control	OR	0.84 (0.60–1.19)	0.328	53%	(0.23–3.16)	0.71	Large heterogeneity, 95% prediction interval includes the null value	Critically low
IL-4RA Ser478Pro polymorphism	Xu 2014[6]	<1000/219	3	Case-control	OR	0.86 (0.63–1.18)	0.359	0%	(0.12–6.46)	0.86	95% prediction interval includes the null value	Critically low
IL-4RA Gln551Arg polymorphism	Xu 2014[6]	>1000/3025	10	Case-control	OR	1.38 (1.00–1.91)	0.0596	74%	(0.43–4.58)	0.05	Large heterogeneity, small study effect, loss of significance under 10 % credibility ceiling	Critically low
IL-13 rs1800925 polymorphism	Chen 2018[9]	>1000/9261	5	Case-control	OR	1.05 (0.91–1.21)	0.374	14%	(0.84–1.33)	0.21	95% prediction interval includes the null value	Low
CD14 rs2569190 polymorphism	Chen 2018[9]	>1000/2301	9	Case-control	OR	1.05 (0.80–1.37)	0.784	78%	(0.37–2.96)	0.31	Large heterogeneity, 95% prediction interval includes the null value	Low
TGF β1 rs1800469 polymorphism	Xiang 2022[7]	>1000/2158	4	Case-control	OR	0.88 (0.36–2.17)	0.804	95%	(0.01–81.32)	0.68	Large heterogeneity, excess significance bias, 95% prediction interval includes the null value	Critically low
CTLA-4 rs3087243 polymorphism	Xiang 2022[7]	>1000/4362	3	Case-control	OR	1.06 (0.93–1.21)	0.368	0%	(0.46–2.45)	0.17	95% prediction interval includes the null value	Critically low
CTLA-4 rs231725 polymorphism	Xiang 2022[7]	>1000/4350	3	Case-control	OR	0.81 (0.63–1.05)	0.106	63%	(0.06–12.12)	0.13	Large heterogeneity, 95% prediction interval includes the null value	Critically low
CTLA-4 rs11571315 polymorphism	Xiang 2022[7]	>1000/4370	3	Case-control	OR	1.06 (0.86–1.30)	0.566	49%	(0.13–8.94)	0.13	95% prediction interval includes the null value	Critically low
IL-4 rs2227284 polymorphism	Xiang 2022[7]	>1000/3270	3	Case-control	OR	1.56 (0.63–3.81)	0.379	96%	(0–475919.57)	0.35	Large heterogeneity, 95% prediction interval includes the null value	Critically low
IL-4 rs2070874 polymorphism	Xiang 2022[7]	>1000/3118	3	Case-control	OR	0.68 (0.34–1.36)	0.229	87%	(0–1034.96)	0.28	Large heterogeneity, 95% prediction interval includes the null value	Critically low
FOX P3 rs3761548 polymorphism	Xiang 2022[7]	>1000/3720	5	Case-control	OR	0.89 (0.65–1.21)	0.437	73%	(0.32–2.49)	0.94	Large heterogeneity, 95% prediction interval includes the null value	Critically low
FOX P3 rs2232365 polymorphism	Xiang 2022[7]	>1000/3118	4	Case-control	OR	0.98 (0.82–1.16)	0.690	16%	(0.70–1.35)	0.51	95% prediction interval includes the null value	Critically low
IL-18 rs1946518 polymorphism	Xiang 2022[7]	>1000/4390	4	Case-control	OR	1.04 (0.89–1.21)	0.731	22%	(0.78–1.35)	0.09	Small study effect, 95% prediction interval includes the null value	Critically low
IL-18 rs187238 polymorphism	Xiang 2022[7]	>1000/3738	3	Case-control	OR	0.91 (0.78–1.06)	0.233	0%	(0.34–2.44)	0.29	95% prediction interval includes the null value	Critically low
IL-18 rs4988359 polymorphism	Xiang 2022[7]	>1000/3738	3	Case-control	OR	0.89 (0.77–1.03)	0.313	0%	(0.34–2.33)	0.04	Small study effect, 95% prediction interval includes the null value	Critically low

Tim-3 rs10515746 polymorphism	Xiang 2022[7]	>1000/2524	4	Case-control	OR	1.30 (0.61–2.76)	0.604	43%	(0.23–5.87)	0.10	Excess significance bias, 95% prediction interval includes the null value	Critically low
IL-4R rs1805010 polymorphism	Xiang 2022[7]	<1000/752	3	Case-control	OR	0.85 (0.63–1.13)	0.259	0%	(0.13–5.60)	0.70	95% prediction interval includes the null value	Critically low
TAP1 333 polymorphism	Xiang 2022[7]	<1000/1006	4	Case-control	OR	1.26 (0.50–3.15)	0.626	86%	(0.02–91.72)	0.64	95% prediction interval includes the null value	Critically low
TAP1 637 polymorphism	Xiang 2022[7]	<1000/948	4	Case-control	OR	0.72 (0.31–1.67)	0.439	86%	(0.02–34.05)	0.98	Large heterogeneity, 95% prediction interval includes the null value	Critically low
TSL rs1898671 polymorphisms	Sun 2015[10]	>1000/16835	3	Case-control	OR	1.09 (0.96–1.23)	0.118	45%	(0.36–3.33)	0.28	95% prediction interval includes the null value	Low

Abbreviations: AMSTAR 2, A Measurement Tool to Assess Systematic Reviews 2; CI, confidence interval; NR,not reported; NA, not available; OR, odds ratio; IL, Interleukin; ADAM, a disintegrin and metalloproteinase; IL-4RA, interleukin-4 receptor a-chain; ACE I/D, Angiotensin-converting enzyme insertion/ deletion; TNF, tumor necrosis factor; CTLA, Cytotoxic T lymphocyte-associated antigen; CD, cluster of differentiation; TGF, Transforming growth factor; FOX, forkhead box; Tim,T cells immunoglobulin domain and mucin domain protein; TAP, Recombinant Antigen Peptide Transporter; TSL,thymic stromal lymphopoietin

References of potential biomarkers

1. Ambrosino P, Parrella P, Formisano R, et al. Clinical application of nasal nitric oxide measurement in allergic rhinitis: A systematic review and meta-analysis. Ann Allergy Asthma Immunol. 2020;125(4):447-459.e5.
2. Wang M, Liu J, Tian X, et al. Association of IL-13 rs20541 polymorphism and risk of allergic rhinitis: evidence from a metaanalysis[J]. International Journal of Clinical and Experimental Medicine, 2016, 9(8): 15914-20.
3. Li Z, Yan F, Yang Z, Zhou J, Chen Y, Ding Z. Association between ADAM33 S2 and V4 polymorphisms and susceptibility to allergic rhinitis: A meta-analysis. Allergol Immunopathol (Madr). 2016;44(2):170-176.
4. Xu Y, Zhang JX. ADAM33 polymorphisms and susceptibility to allergic rhinitis: a meta-analysis. Eur Arch Otorhinolaryngol. 2015;272(3):597-605.
5. Li ZP, Yin LL, Wang H, Liu LS. Association between promoter polymorphisms of interleukin-4 gene and allergic rhinitis risk: a meta-analysis. J Huazhong Univ Sci Technolog Med Sci. 2014;34(3):306-313.
6. Xu Y, Zhang JX. Interleukin-4 receptor α-chain polymorphisms and susceptibility to allergic rhinitis: a meta-analysis. Eur Arch Otorhinolaryngol. 2014;271(8):2205-2212.
7. Xiang F, Zeng Z, Wang L, Yang YP, Zhang QX. Polymorphisms and AR: A Systematic Review and Meta-Analyses. Front Genet. 2022;13:899923.
8. Jiang F, Yan A. IL-4 rs2243250 polymorphism associated with susceptibility to allergic rhinitis: a meta-analysis. Biosci Rep. 2021;41(4):BSR20210522.
9. Chen ML, Zhao H, Huang QP, Xie ZF. Single nucleotide polymorphisms of IL-13 and CD14 genes in allergic rhinitis: a meta-analysis. Eur Arch Otorhinolaryngol. 2018;275(6):1491-1500.
10. Sun Q, Liu Y, Zhang S, et al. Thymic stromal lymphopoietin polymorphisms and allergic rhinitis risk: a systematic review and meta-analysis with 6351 cases and 11472 controls. Int J Clin Exp Med. 2015;8(9):15752-15758.
11. Lin H, Lin D, Zheng CQ. Angiotensin-converting enzyme insertion/deletion polymorphism associated with allergic rhinitis susceptibility: evidence from 1410 subjects. J Renin Angiotensin Aldosterone Syst. 2014;15(4):593-600.

Supplementary analyses result of biomarker

Potential biomarker	Source	Effect metrics	Effect estimate of the largest study (95% CI)	The largest p value in the meta-analysis	Excess significance bias (p > 0.1)	whether the 95% PI includes the null value	whether the 95% CI of the largest study includes the null
Nasal nitric oxide	Ambrosino 2020	SMD	5.03 (4.67–5.39)	3.75e-07	5.24e-01	Null	Not null
ADAM33 S2 polymorphism	Li 2016	OR	1.04 (0.85–1.26)	7.58e-02	7.13e-03	Null	Null
ADAM33 V4 polymorphism	Li 2016	OR	1.28 (1.07–1.54)	1.87e-01	4.83e-01	Null	Not null
ADAM33 T1 polymorphism	Xu 2014	OR	0.94 (0.71–1.23)	8.18e-01	4.14e-01	Null	Null
ADAM33 T2 polymorphism	Xu 2014	OR	0.91 (0.69–1.19)	9.94e-01	2.19e-01	Null	Null
ADAM33 Q−1 polymorphism	Xu 2014	OR	1.23 (0.93–1.64)	7.50e-02	7.27e-01	Null	Null
IL-4 rs2243250 polymorphism	Jiang 2021	OR	1.12 (0.93–1.34)	2.99e-01	6.87e-01	Null	Null
IL-13 rs20541 polymorphism	Wang 2016	OR	1.11 (0.95–1.31)	1.91e-04	9.92e-02	Not null	Null
Serum IL-4 level	Li 2014	OR	4.42 (3.63–5.21)	3.70e-04	9.93e-01	Null	Not null
IL-4RA Gln551Arg polymorphism	Xu 2013	OR	1.11 (0.80–1.54)	1.39e-01	1.23e-01	Null	Null
IL-4RA Ile50Val polymorphism	Xu 2013	OR	1.18 (0.80–1.74)	9.60e-01	3.65e-01	Null	Null
IL-4RA Ser478Pro polymorphism	Xu 2013	OR	0.97 (0.60–1.59)	6.94e-01	6.94e-01	Null	Null
IL-13 rs1800925 polymorphism	Chen 2018	OR	1.14 (0.97–1.34)	7.95e-01	7.29e-01	Null	Null
CD14 rs2569190 polymorphism	Chen 2018	OR	1.13 (0.90–1.42)	9.84e-01	6.25e-01	Null	Null
TNFα rs1800629 polymorphism	Xiang 2022	OR	1.12 (0.77–1.64)	1.26e-01	6.24e-01	Null	Null
TGFβ1 rs1800469 polymorphism	Xiang 2022	OR	1.33 (1.04–1.70)	7.14e-01	4.22e-04	Null	Not null
CTLA-4 rs3087243 polymorphism	Xiang 2022	OR	1.11 (0.95–1.29)	6.82e-01	6.87e-01	Null	Null

CTLA-4 rs231725 polymorphism	Xiang 2022	OR	0.97 (0.84–1.12)	3.42e-01	4.00e-01	Null	Null
CTLA-4 rs11571302 polymorphism	Xiang 2022	OR	1.53 (1.31–1.78)	2.04e-01	8.71e-01	Null	Not null
CTLA-4 rs11571315 polymorphism	Xiang 2022	OR	0.93 (0.81–1.08)	7.61e-01	7.16e-01	Null	Null
IL-4 rs2227284 polymorphism	Xiang 2022	OR	0.83 (0.68–1.01)	5.47e-01	6.12e-01	Null	Null
IL-4 rs2070874 polymorphism	Xiang 2022	OR	1.14 (0.95–1.38)	6.69e-01	3.62e-01	Null	Null
FOXP3 rs3761548 polymorphism	Xiang 2022	OR	0.90 (0.68–1.19)	7.78e-01	5.94e-01	Null	Null
FOXP3 rs2232365 polymorphism	Xiang 2022	OR	0.95 (0.76–1.18)	9.49e-01	6.56e-01	Null	Null
IL-18 rs1946518 polymorphism	Xiang 2022	OR	0.96 (0.79–1.16)	9.03e-01	6.51e-01	Null	Null
IL-18 rs187238 polymorphism	Xiang 2022	OR	0.88 (0.71–1.09)	6.06e-01	7.17e-01	Null	Null
IL-18 rs4988359 polymorphism	Xiang 2022	OR	0.87 (0.71–1.07)	8.81e-01	7.54e-01	Null	Null
Tim-3 rs10515746 polymorphism	Xiang 2022	OR	20.93(1.18–372.62)	9.94e-01	4.29e-02	Null	Not null
IL-4R rs1801275 polymorphism	Xiang 2022	OR	0.95 (0.68–1.33)	5.68e-02	3.14e-01	Null	Null
IL-4R rs1805010 polymorphism	Xiang 2022	OR	0.79 (0.52–1.19)	6.40e-01	7.13e-01	Null	Null
TAP1 333 polymorphism	Xiang 2022	OR	1.07 (0.65–1.77)	7.12e-01	6.79e-01	Null	Null
TAP1 637 polymorphism	Xiang 2022	OR	1.03 (0.64–1.66)	6.46e-01	6.78e-01	Null	Null
TSL rs1898671 polymorphisms	Sun 2015	OR	1.15 (1.08–1.22)	9.56e-01	6.21e-01	Null	Not null
ACE I/D polymorphism	Lin 2014	OR	1.21 (0.89–1.64)	2.57e-02	6.58e-01	Null	Null

Abbreviations: AMSTAR 2, A Measurement Tool to Assess Systematic Reviews 2; CI, confidence interval; NR,not reported; NA, not available; OR, odds ratio; IL, Interleukin; ADAM, a disintegrin and metalloproteinase; IL-4RA, interleukin-4 receptor a-chain; ACE I/D, Angiotensin-converting enzyme insertion/ deletion; TNF, tumor necrosis factor; CTLA, Cytotoxic T lymphocyte-associated antigen; CD, cluster of differentiation; TGF, Transforming growth factor; FOX, forkhead box; Tim,T cells immunoglobulin domain and mucin domain protein; TAP, Recombinant Antigen Peptide Transporter; TSL,thymic stromal lymphopoietin

Figure S1. Random-effects meta-analysis of the association between tic disorders and AR (forest plot and funnel plot)

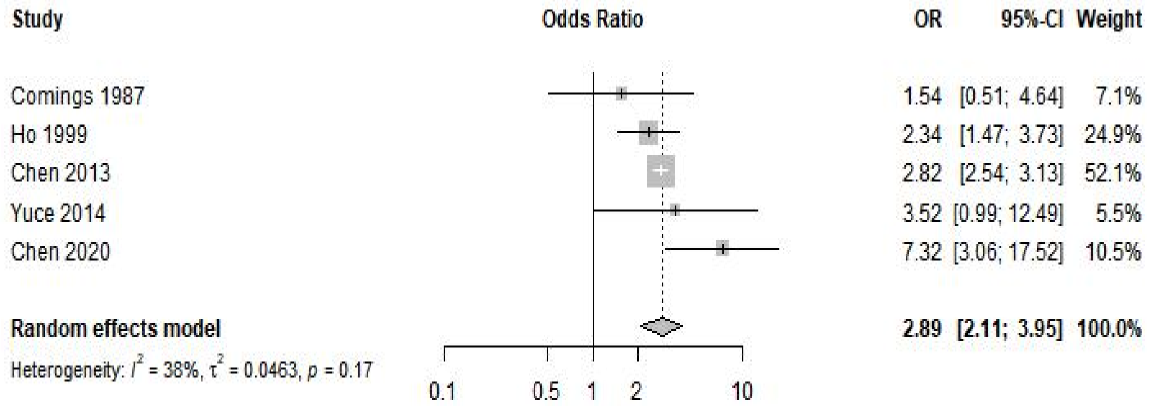

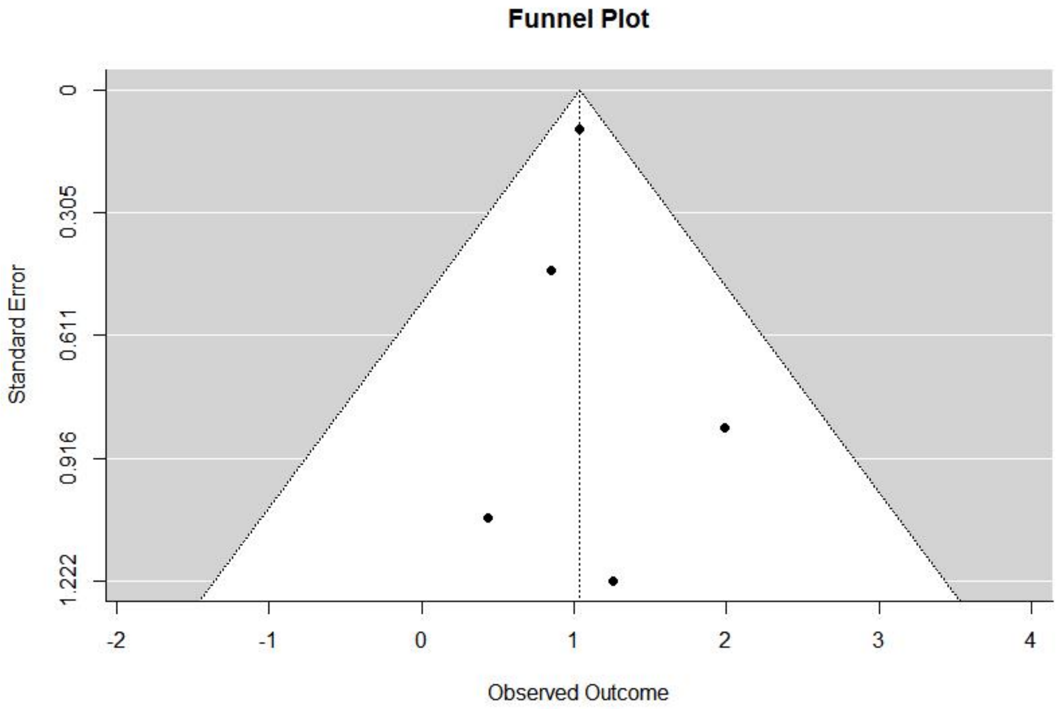

Figure S2. Random-effects meta-analysis of the association between early-life antibiotic use and AR (forest plot and funnel plot)

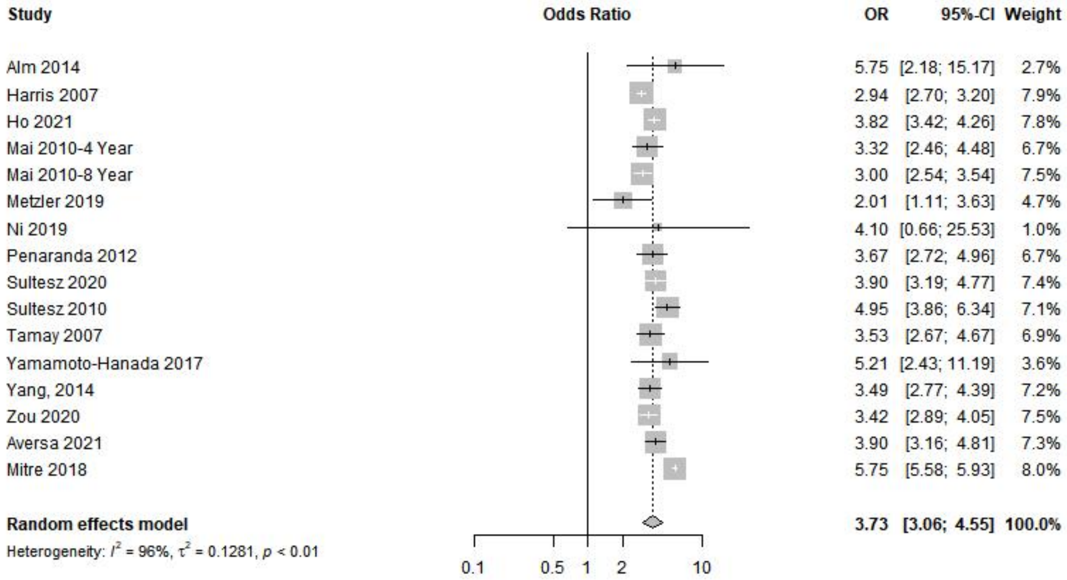

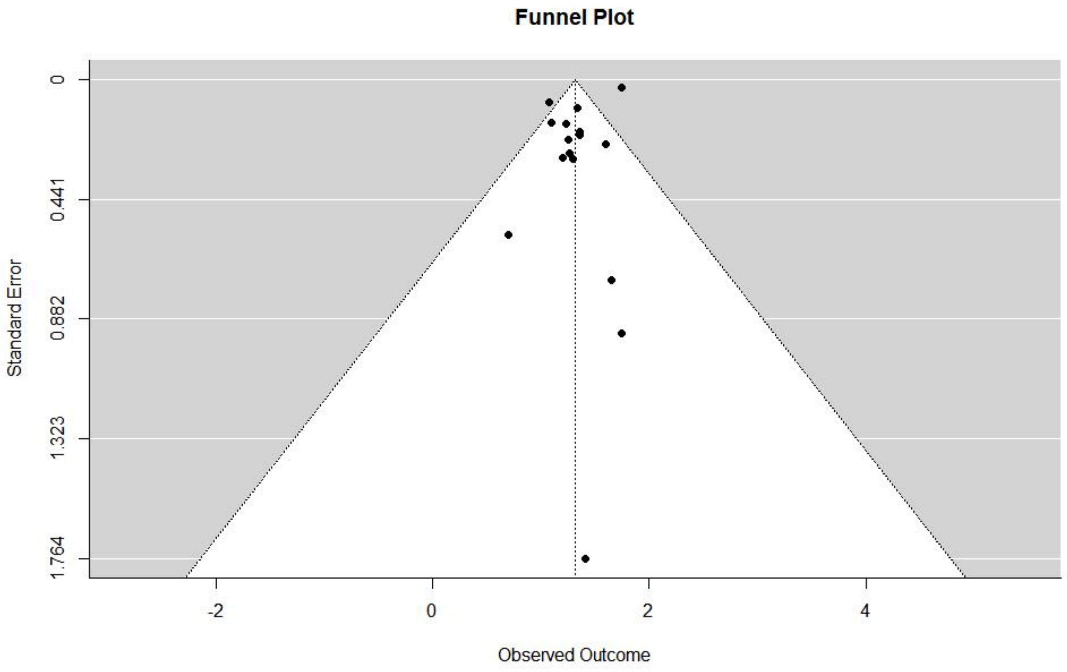

Figure S3. Random-effects meta-analysis of the association between exposure to indoor dampness and AR (forest plot and funnel plot)

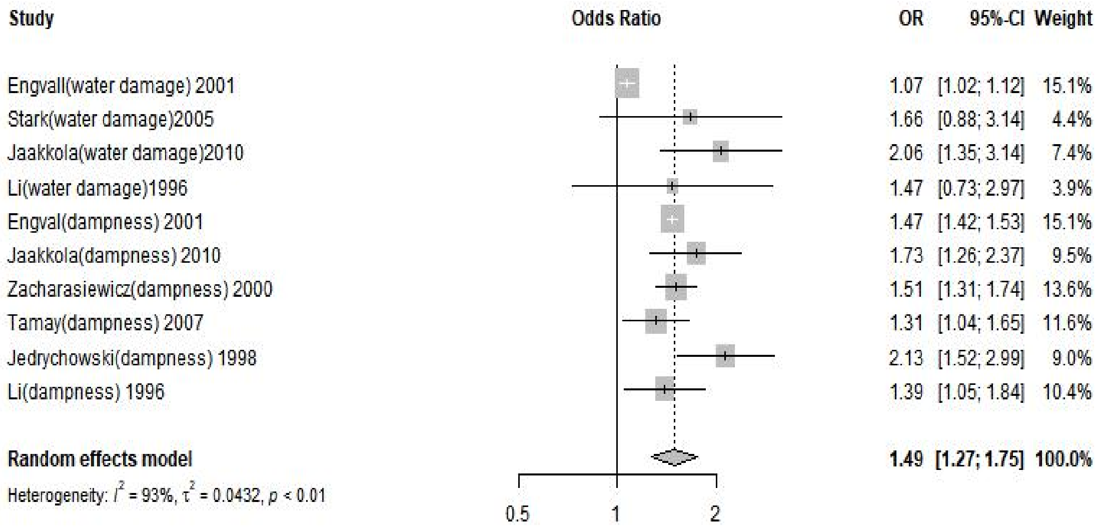

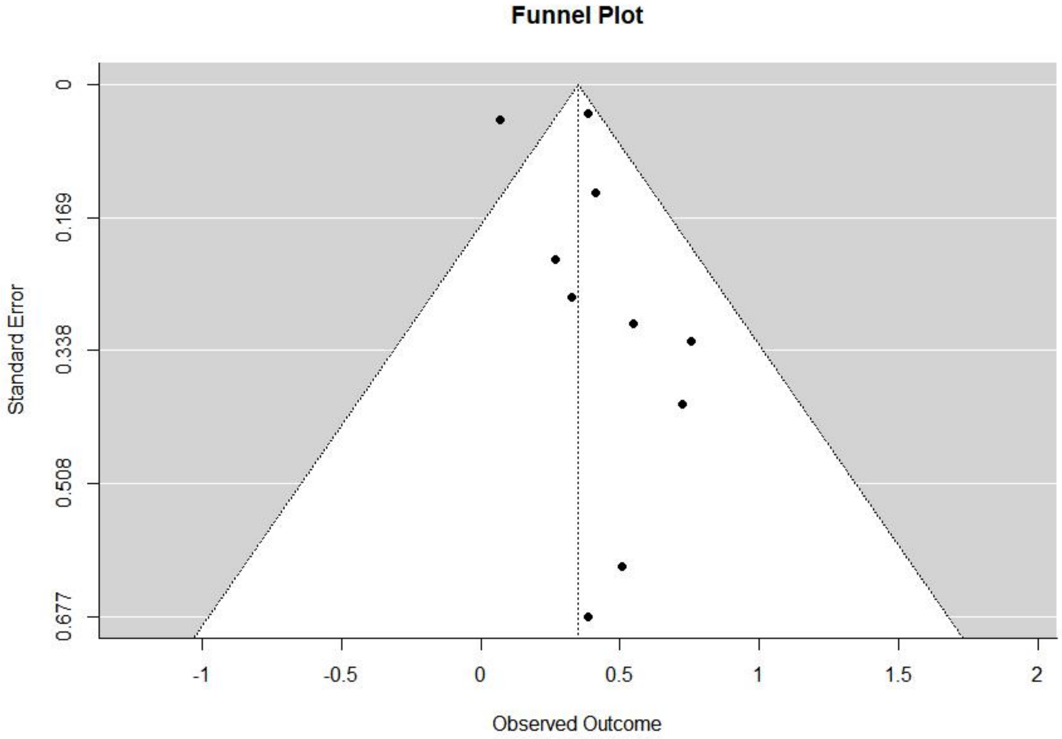

Figure S4. Random-effects meta-analysis of the association between prolonged breastfeeding and AR (forest plot and funnel plot)

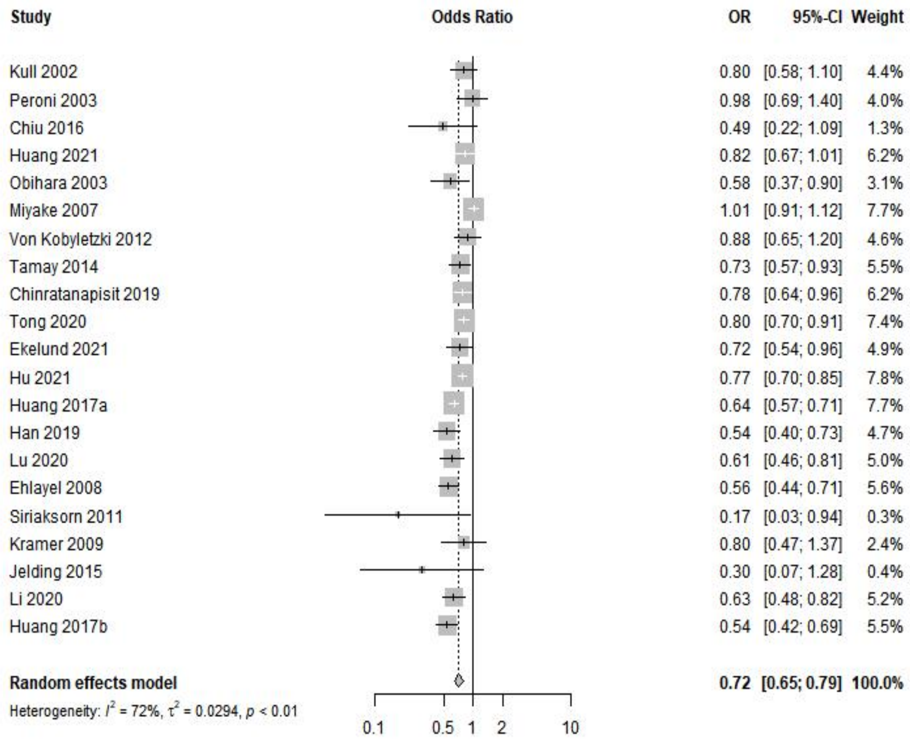

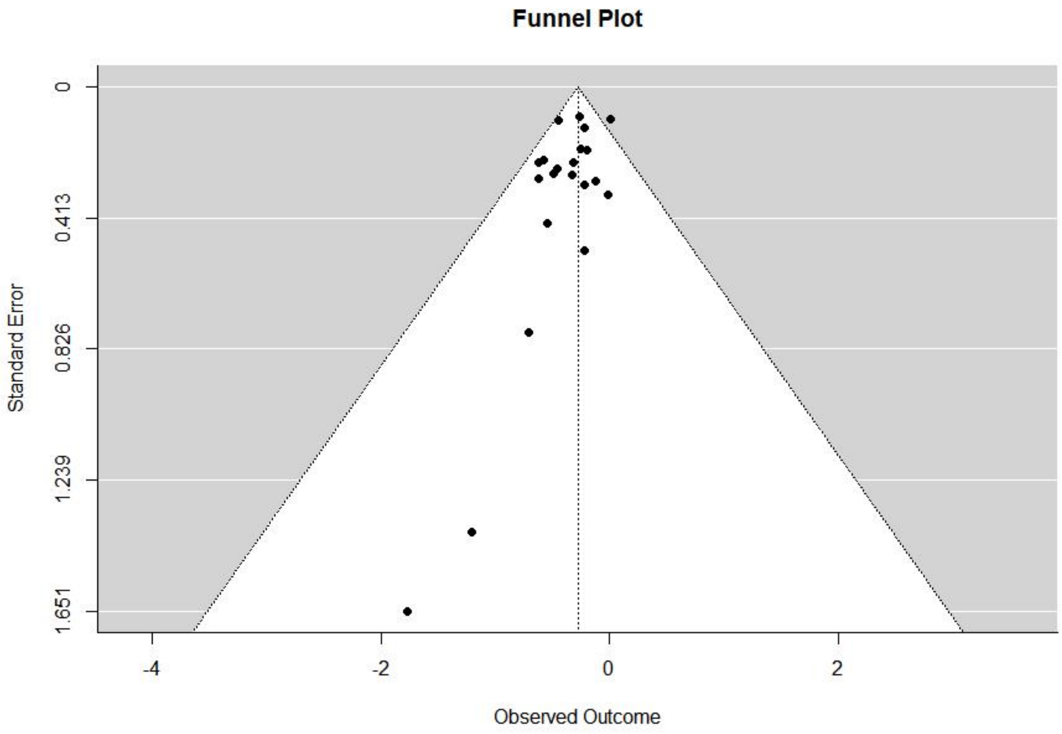

Figure S5. Random-effects meta-analysis of the association between coronavirus disease 2019 and AR (forest plot and funnel plot)

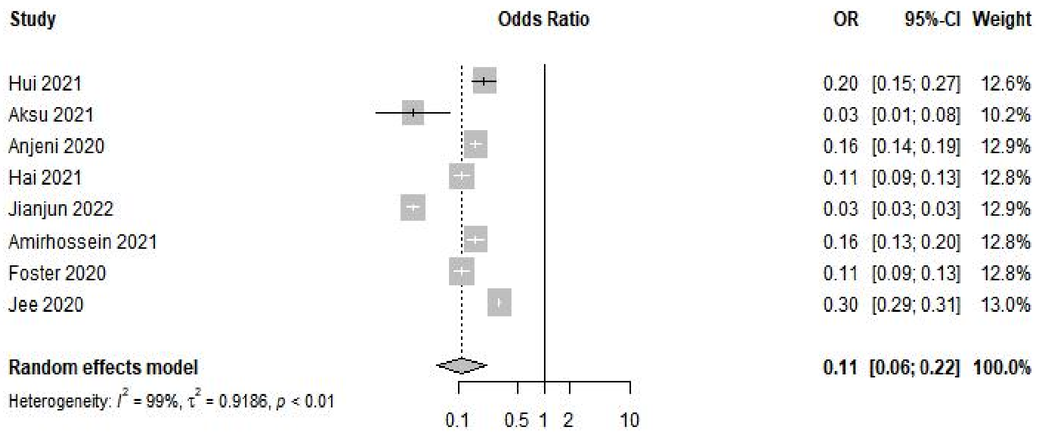

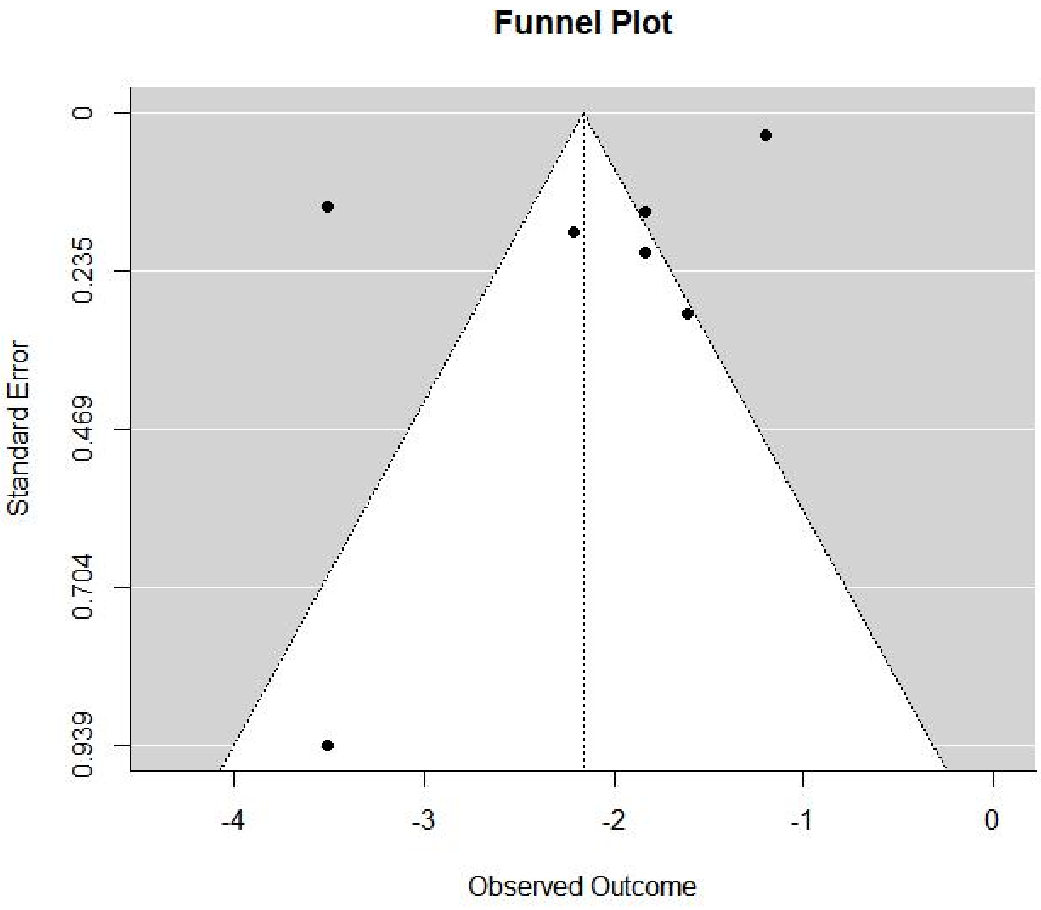

Figure S6. Random-effects meta-analysis of the association between acetaminophen exposure and AR (forest plot and funnel plot)

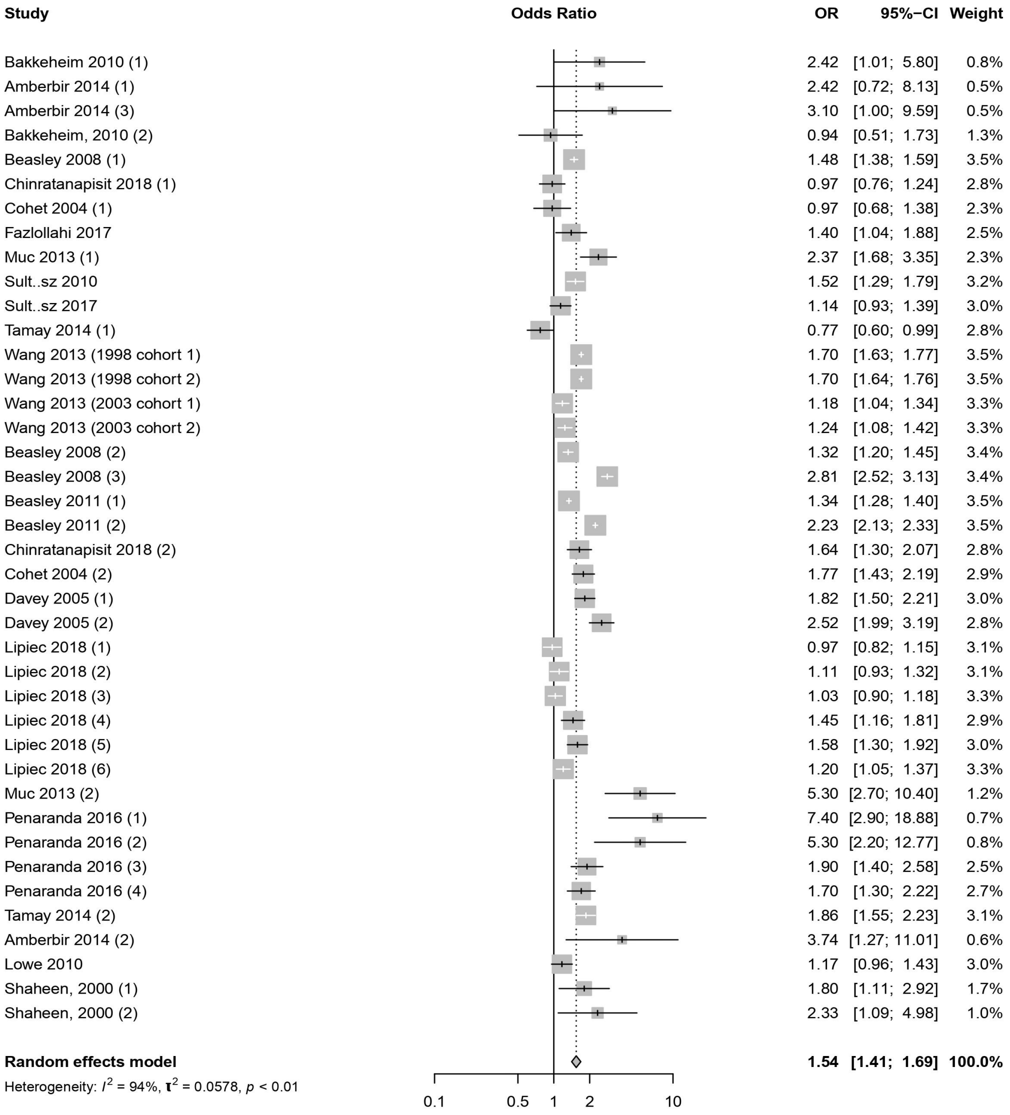

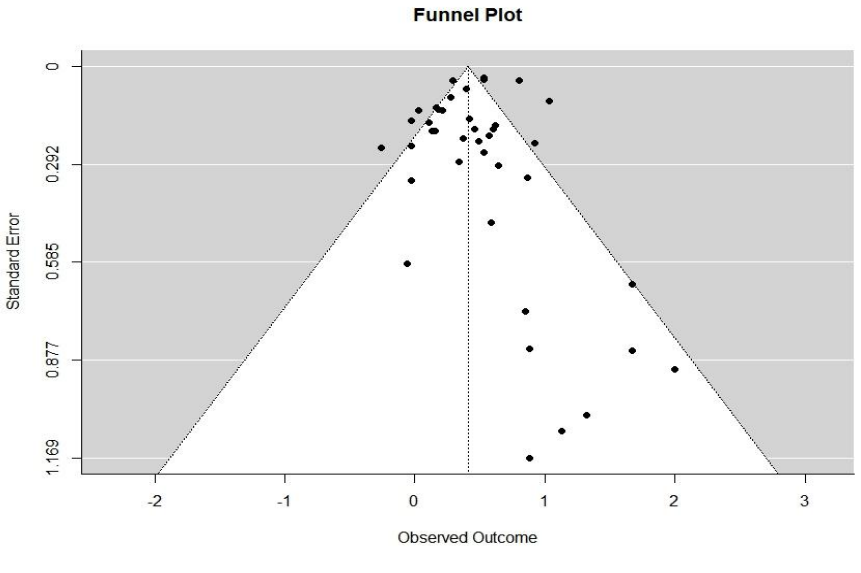

Figure S7. Random-effects meta-analysis of the association between childhood acid suppressant use and AR (forest plot and funnel plot)

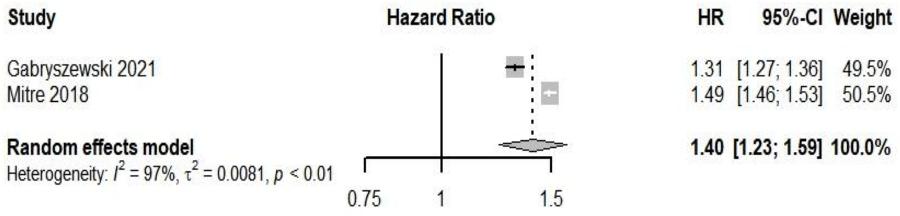

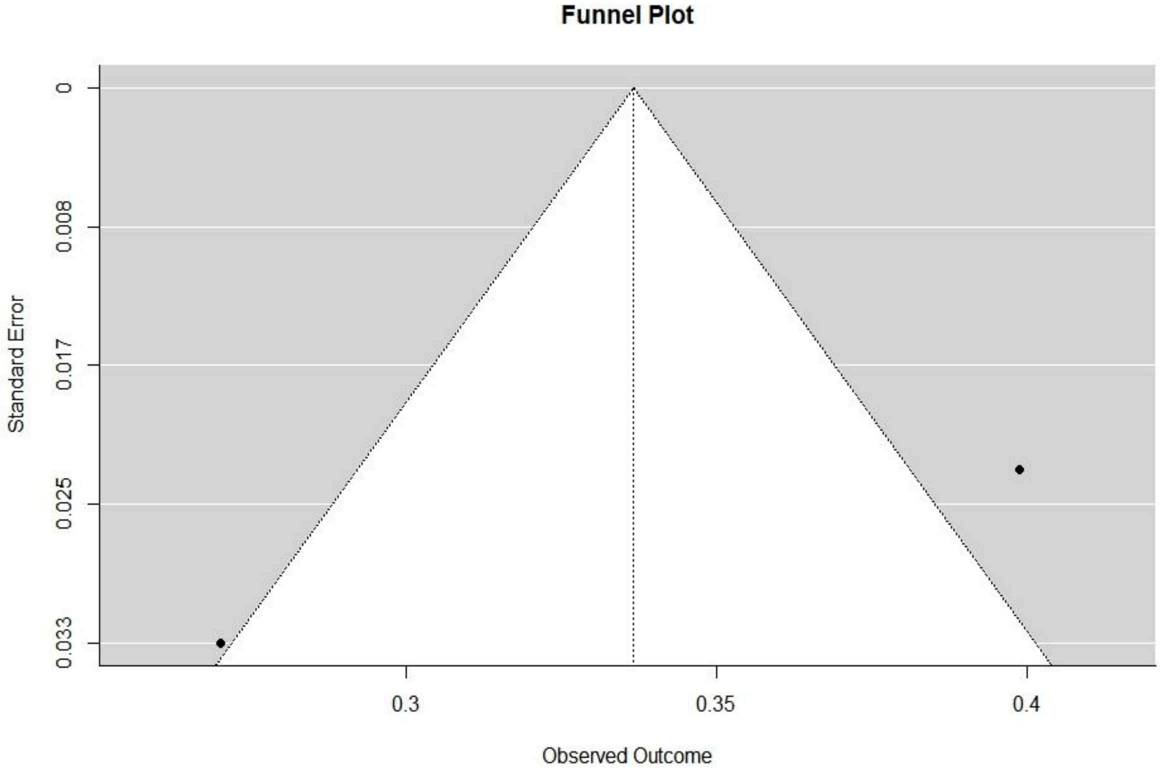

Figure S8. Random-effects meta-analysis of the association between exposure to indoor mold and AR (forest plot and funnel plot)

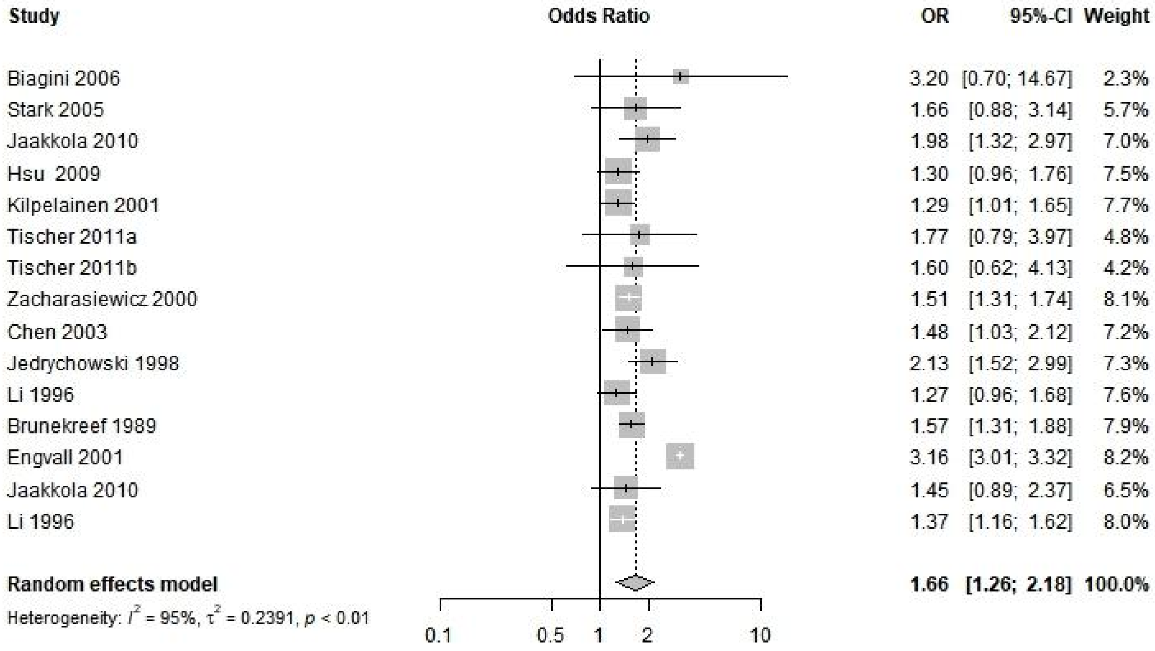

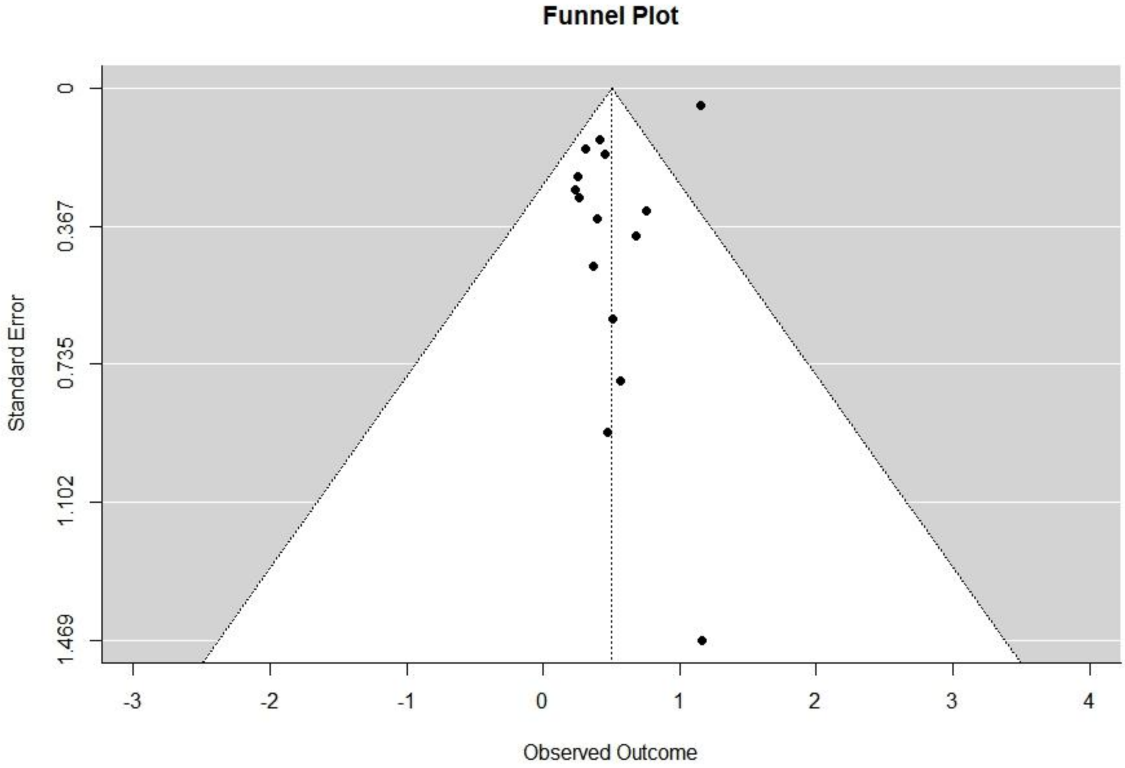

Figure S9. Random-effects meta-analysis of the association between Ambient particulate matter(PM10) and AR (forest plot and funnel plot)

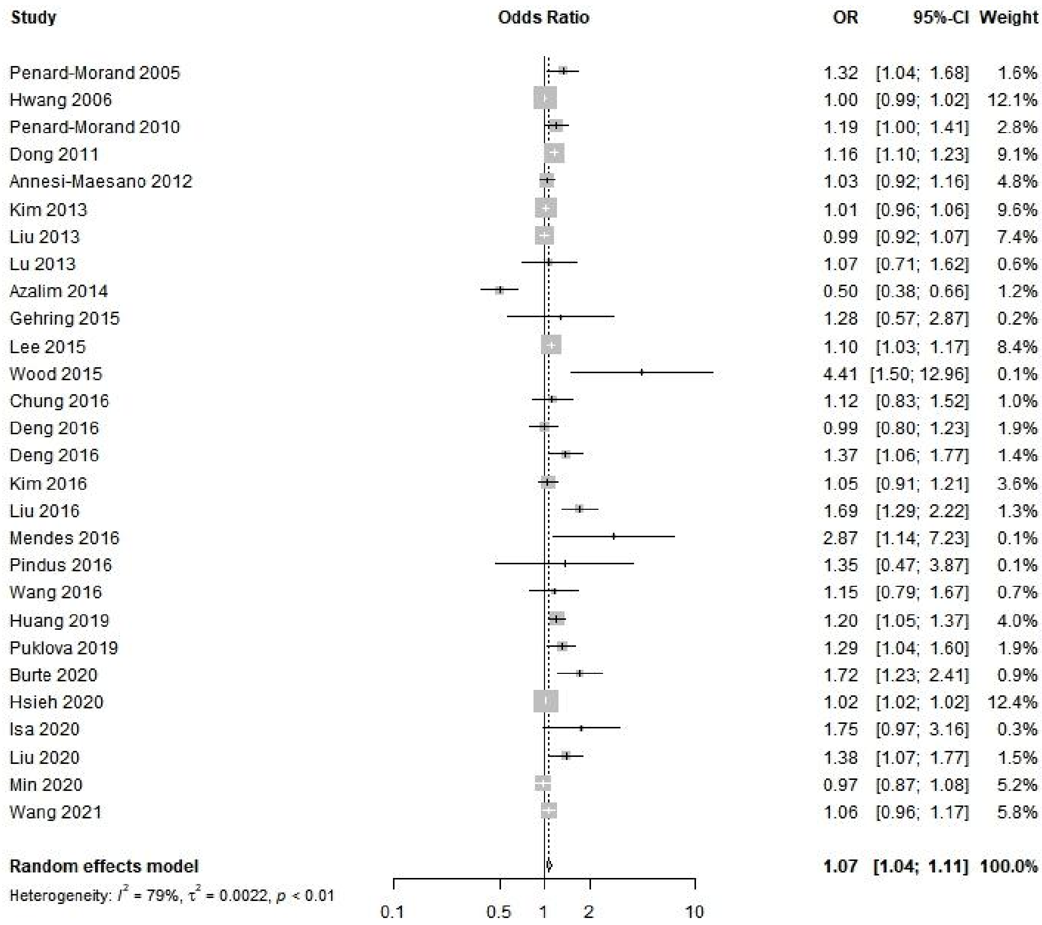

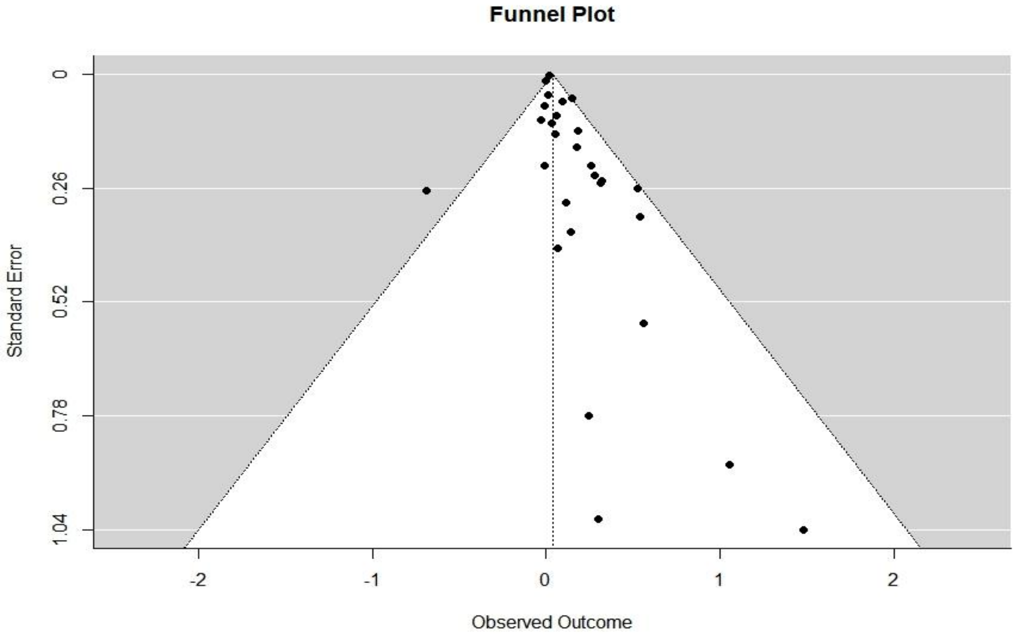

Figure S10. Random-effects meta-analysis of the association between sulfur dioxide and AR (forest plot and funnel plot)

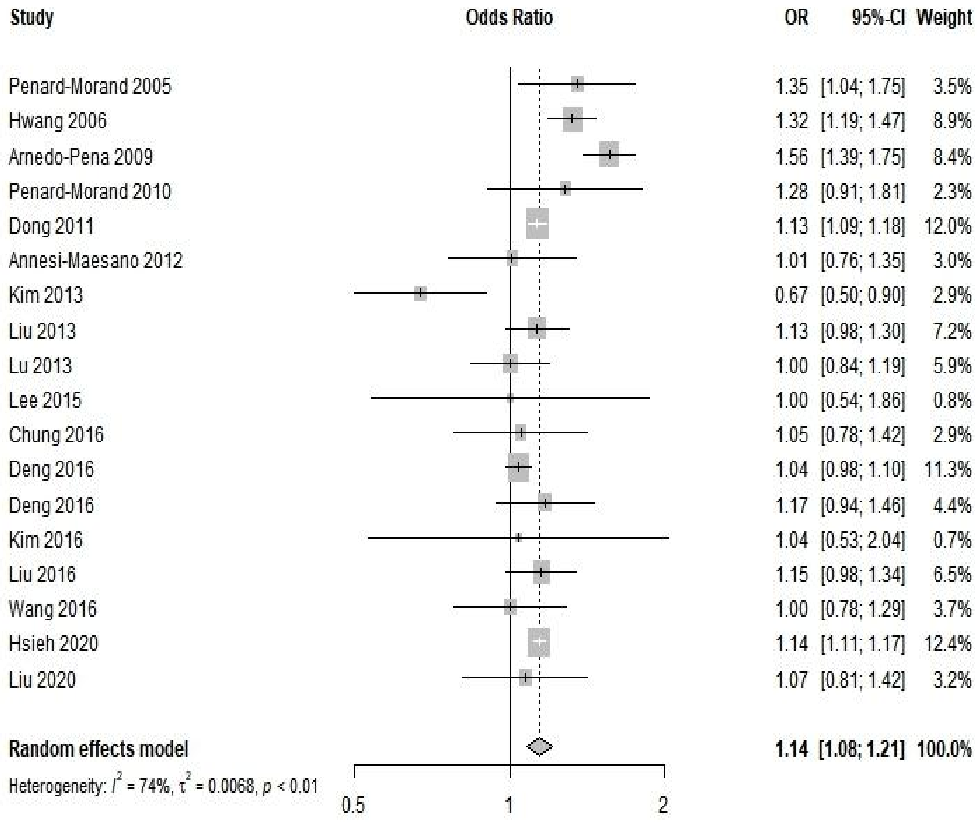

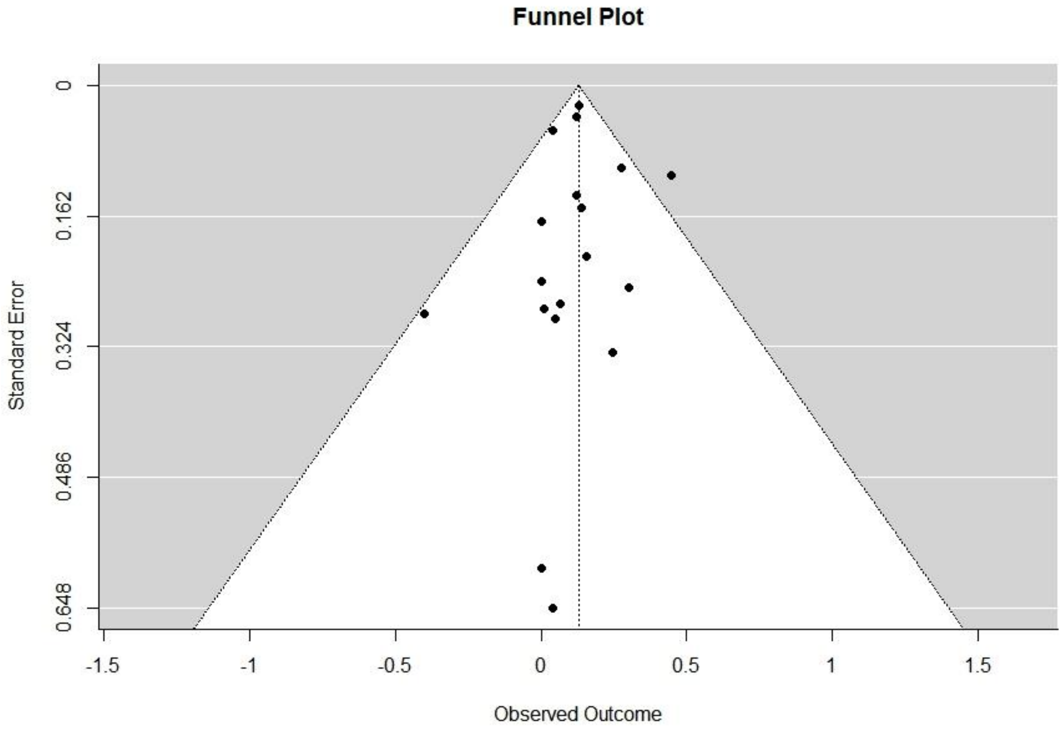

Figure S11. Random-effects meta-analysis of the association between nitrogen dioxide and AR (forest plot and funnel plot)

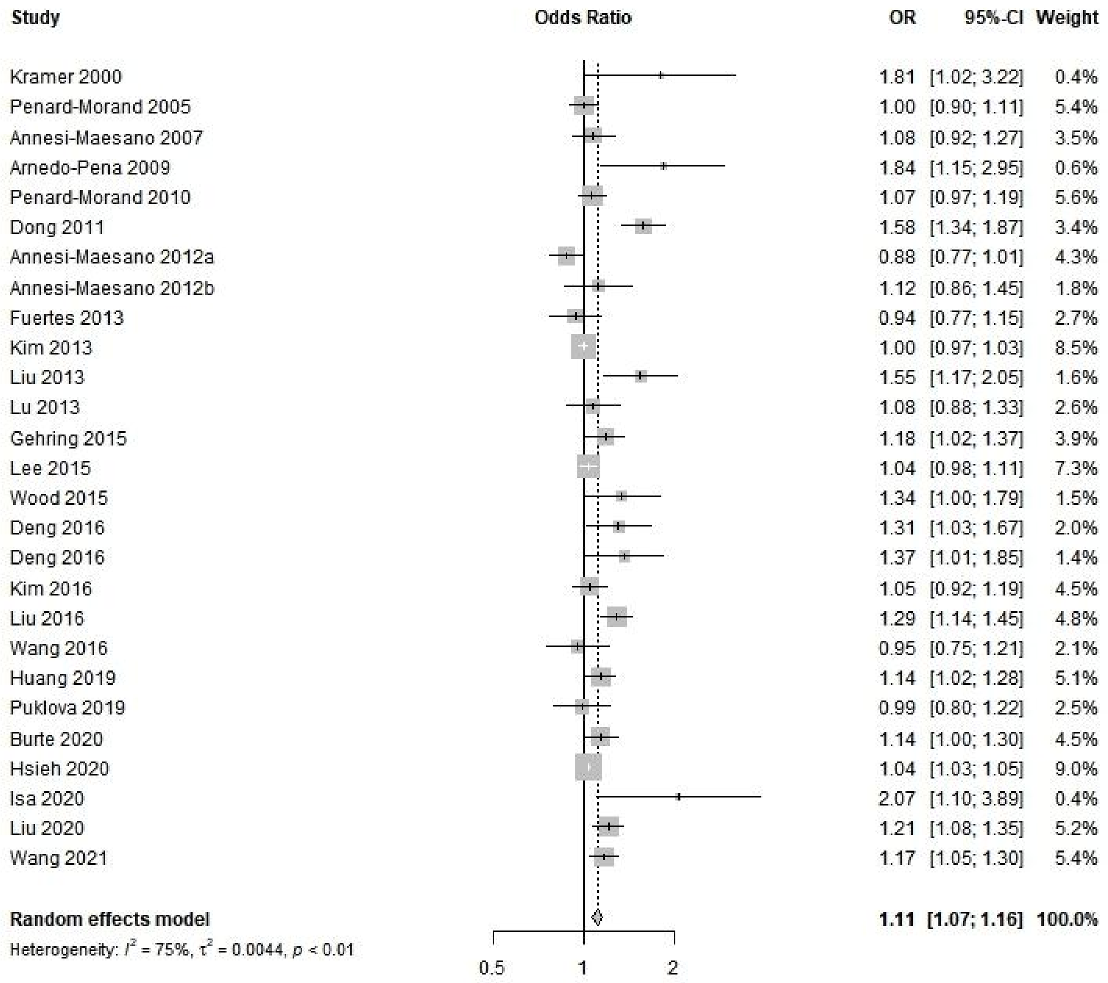

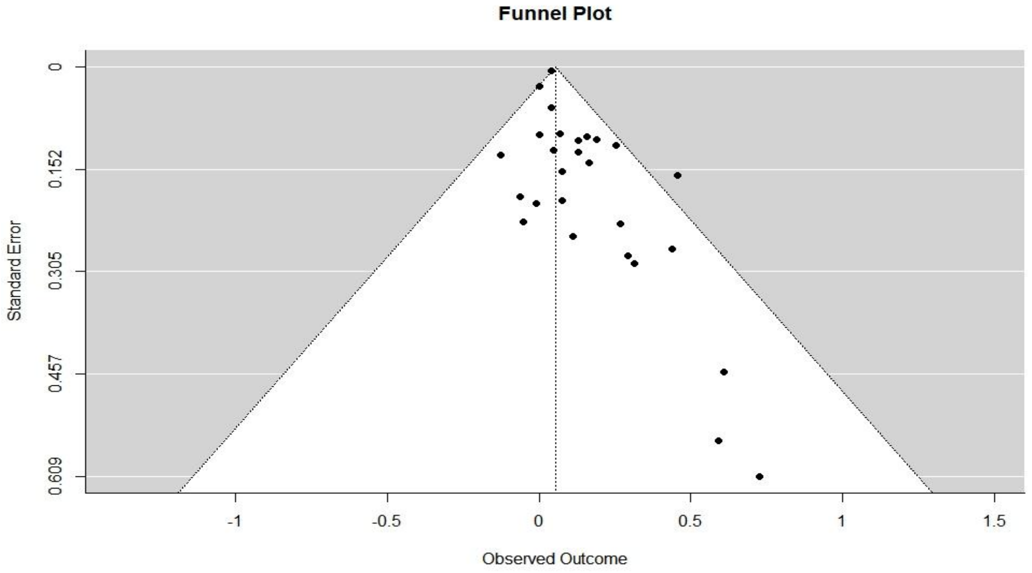

Figure S12. Random-effects meta-analysis of the association between farm milk consumption and AR (forest plot and funnel plot)

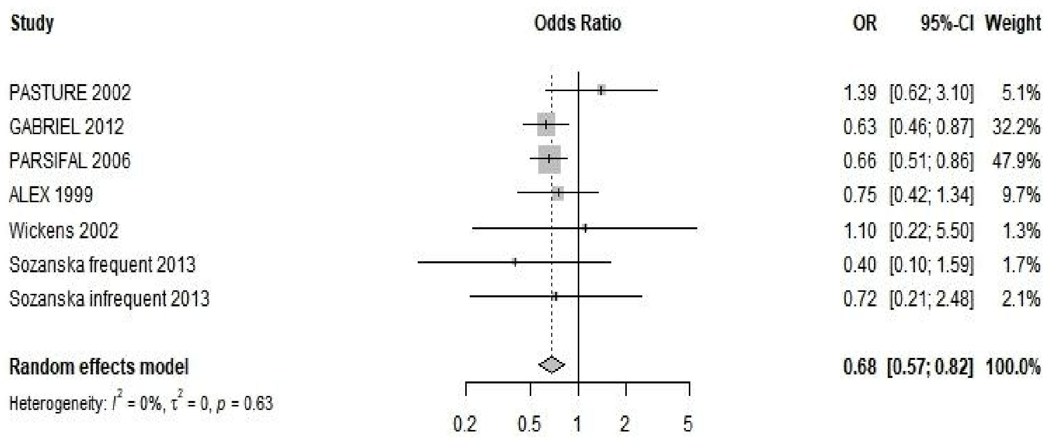

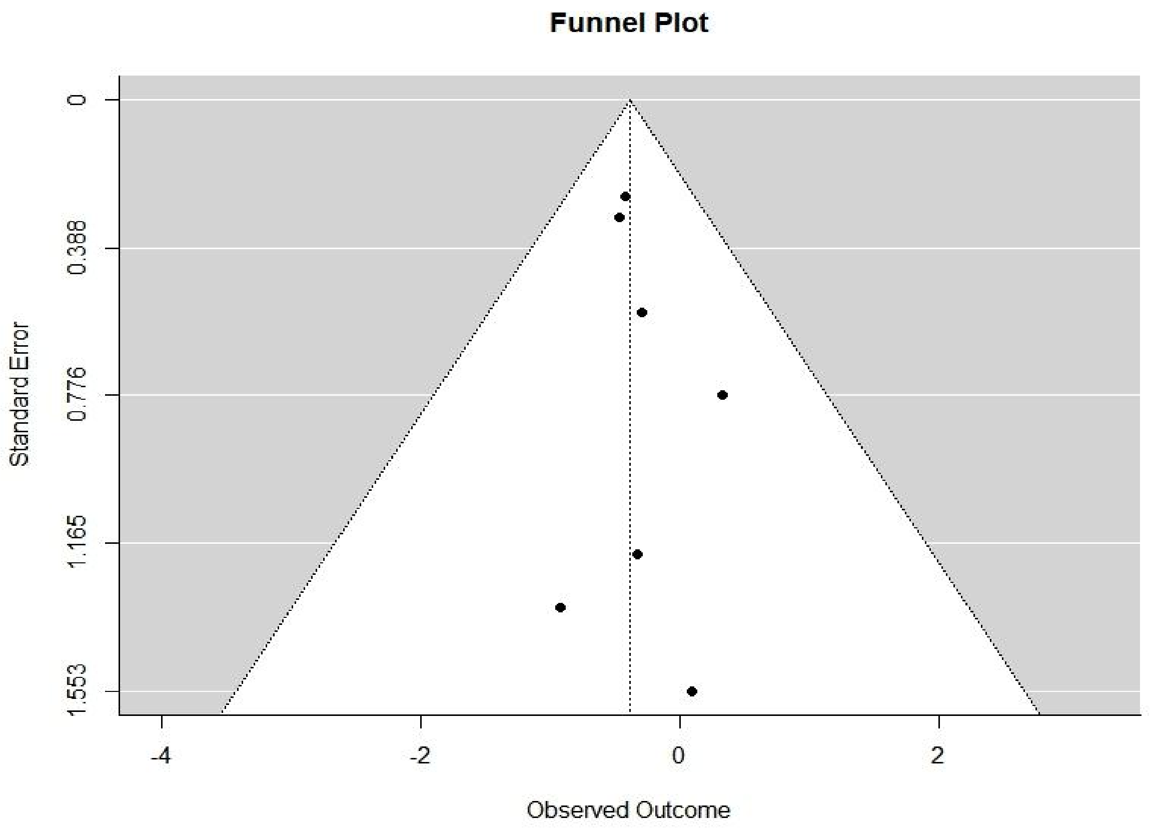

Figure S13. Random-effects meta-analysis of the association between passive exposure to tobacco smoking and AR (forest plot and funnel plot)

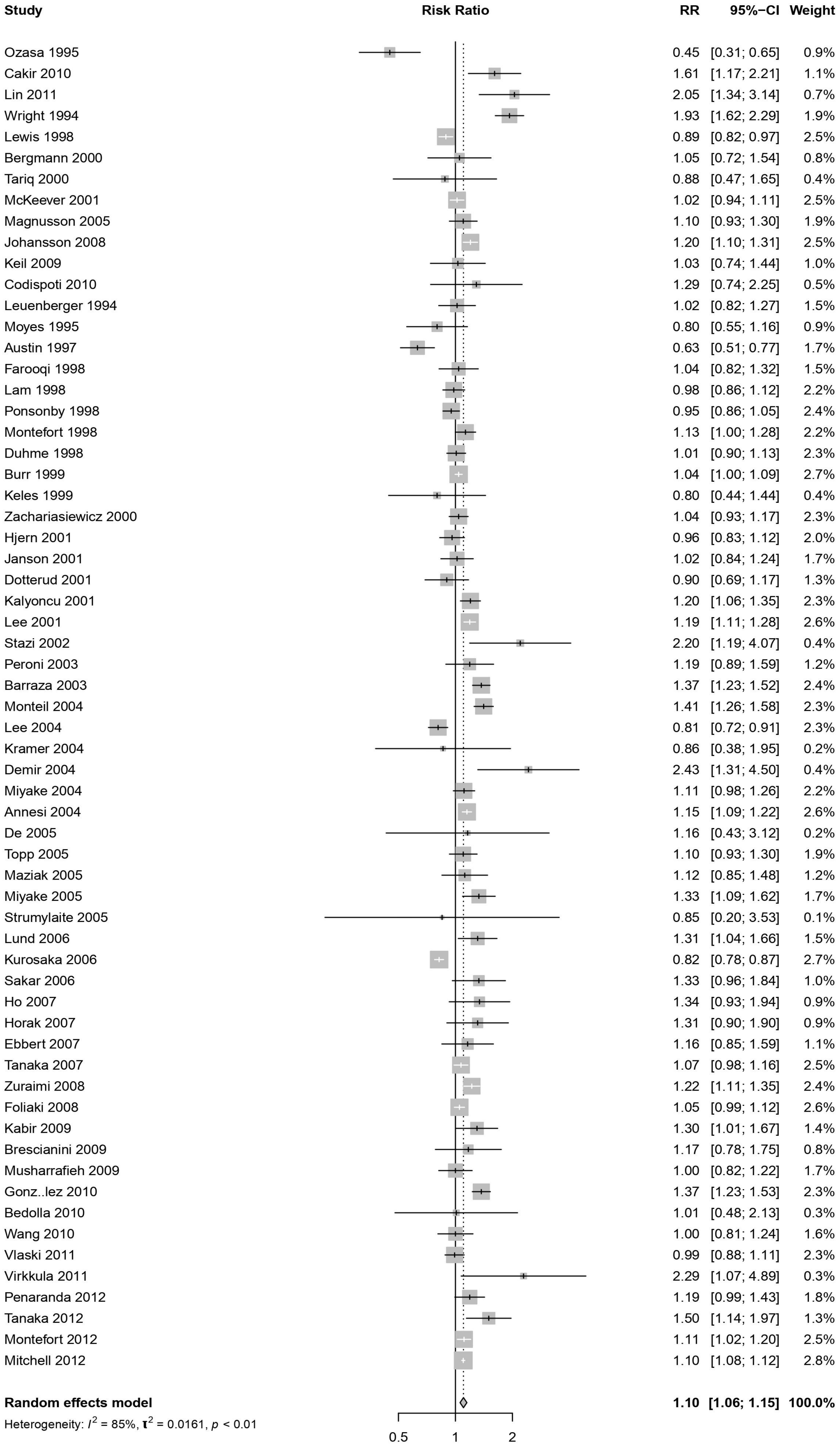

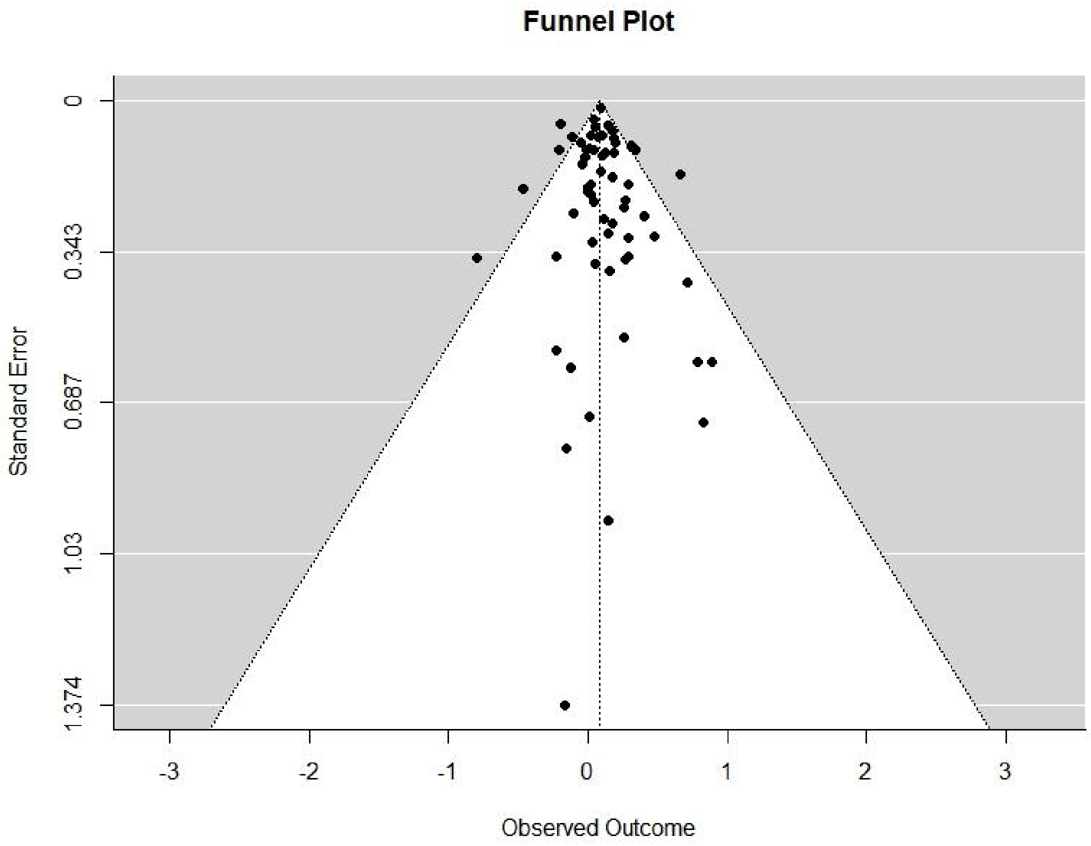

Funnel Plot
0
0.343
0.687
1.03
1.374
Standard Error
-3
-2
-1
0
1
2
3
Observed Outcome

Figure S14. Random-effects meta-analysis of the association between early dietary introduction of fish and AR (forest plot and funnel plot)

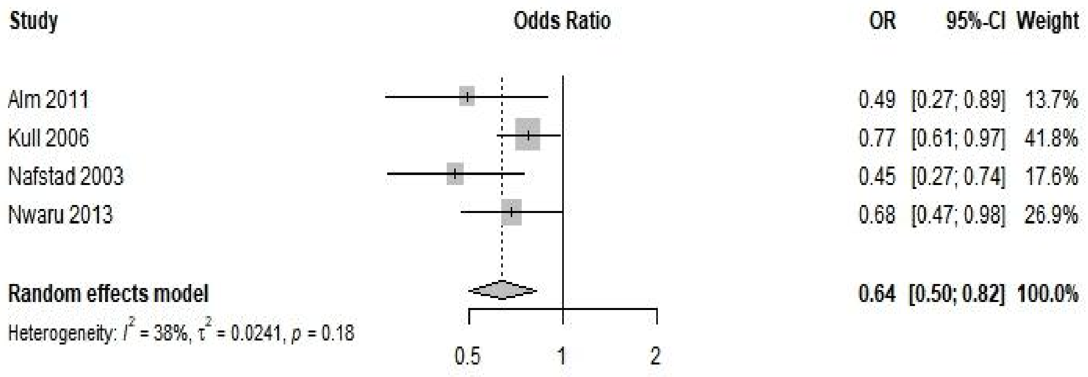

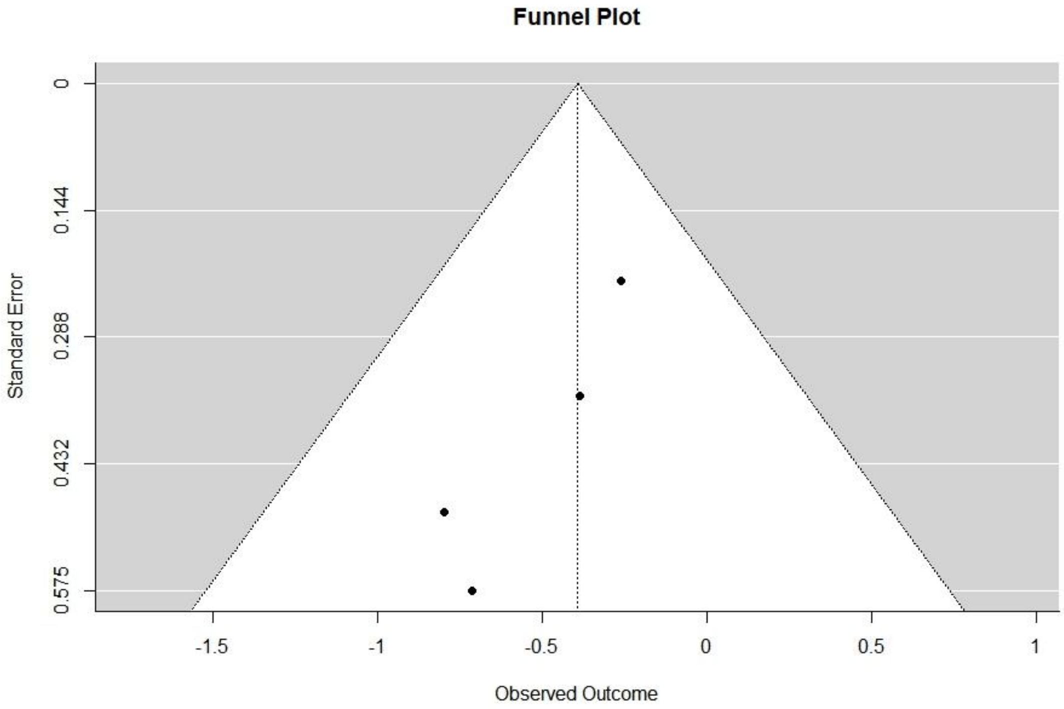

Figure S15. Random-effects meta-analysis of the association between history of Kawasaki disease and AR (forest plot and funnel plot)

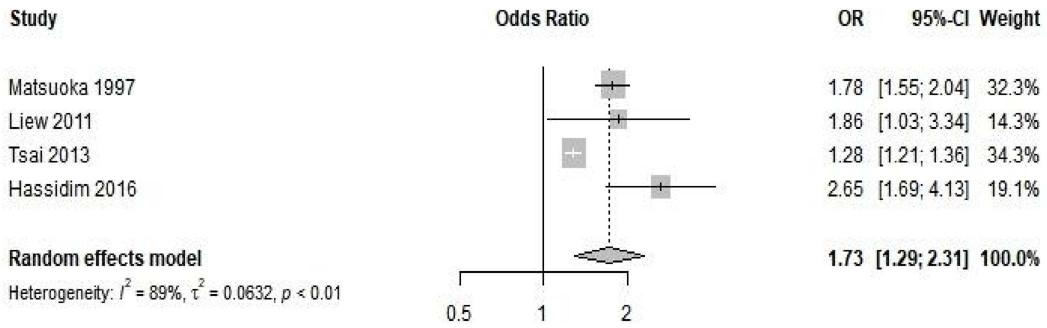

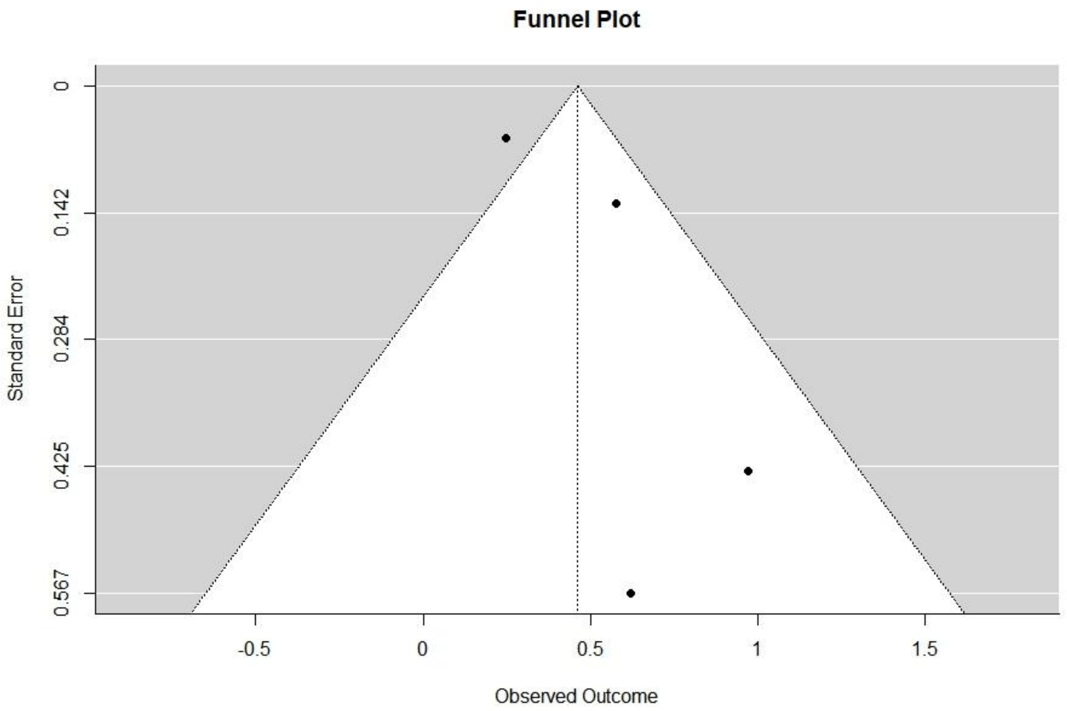

Figure S16. Random-effects meta-analysis of the association between educational level and AR (forest plot and funnel plot)

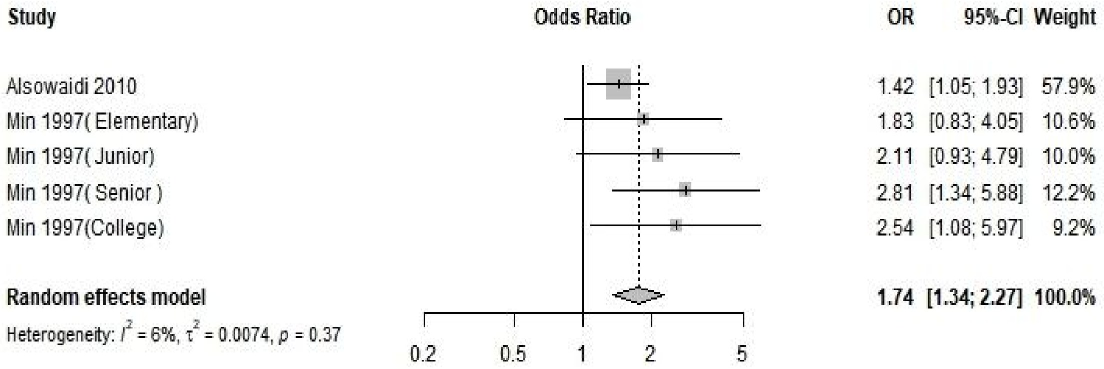

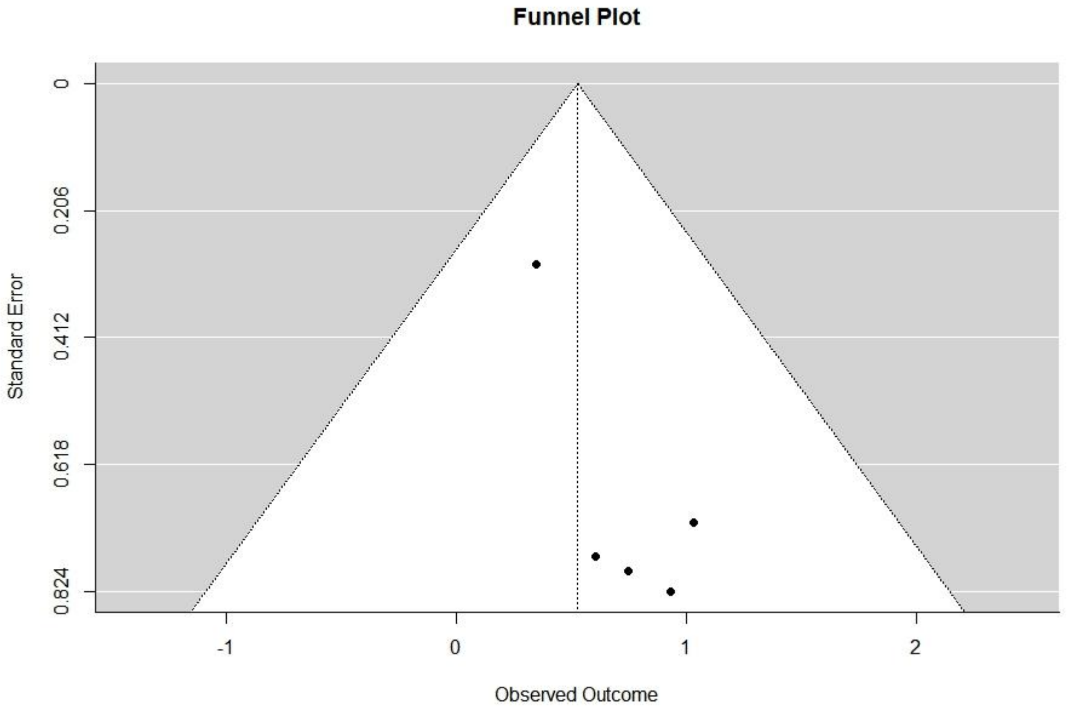

Figure S17. Random-effects meta-analysis of the association between family history of allergic diseases and AR (forest plot and funnel plot)

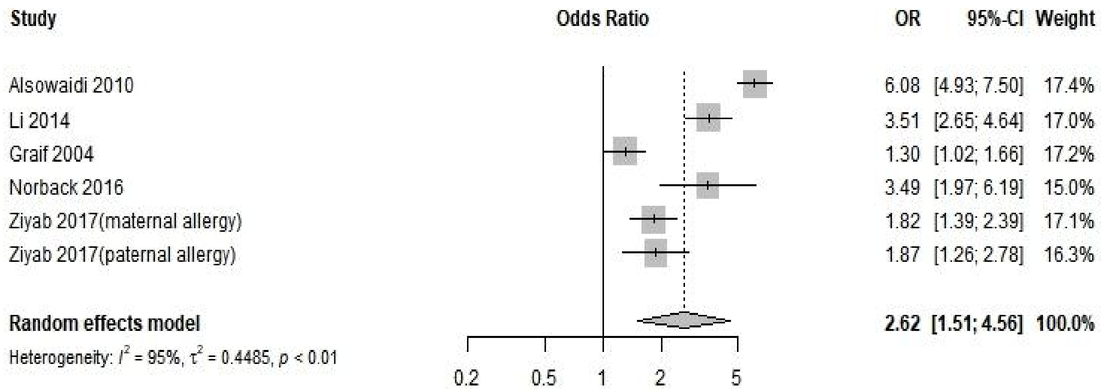

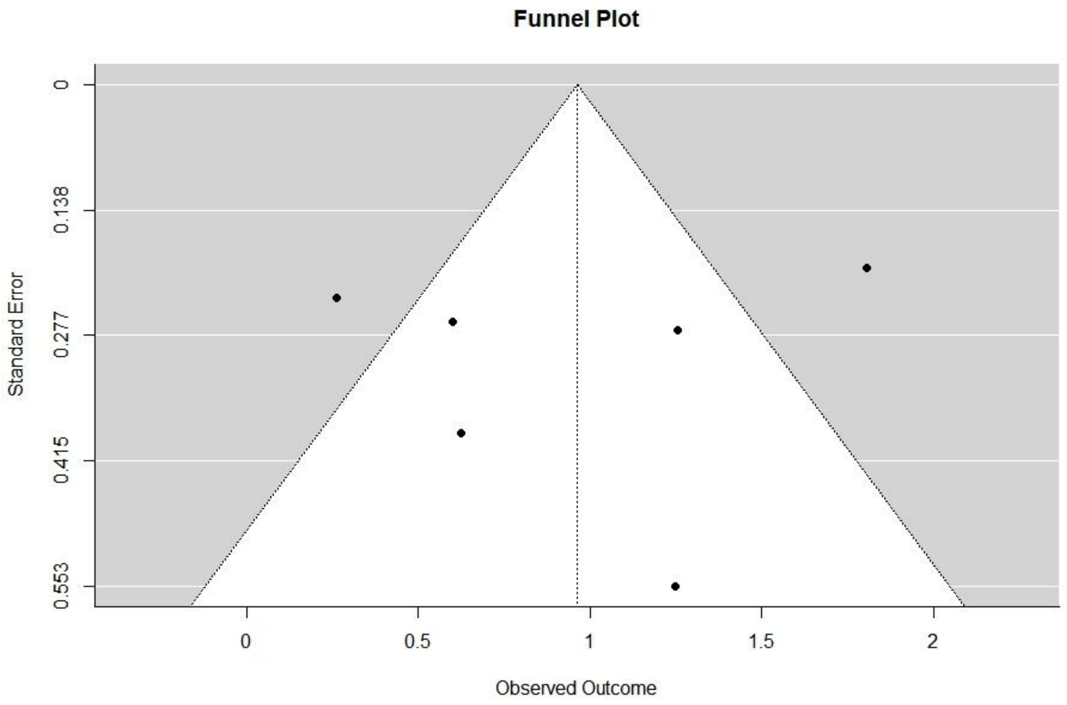

Figure S18. Random-effects meta-analysis of the association between ambient particulate matter(PM2.5) and AR (forest plot and funnel plot)

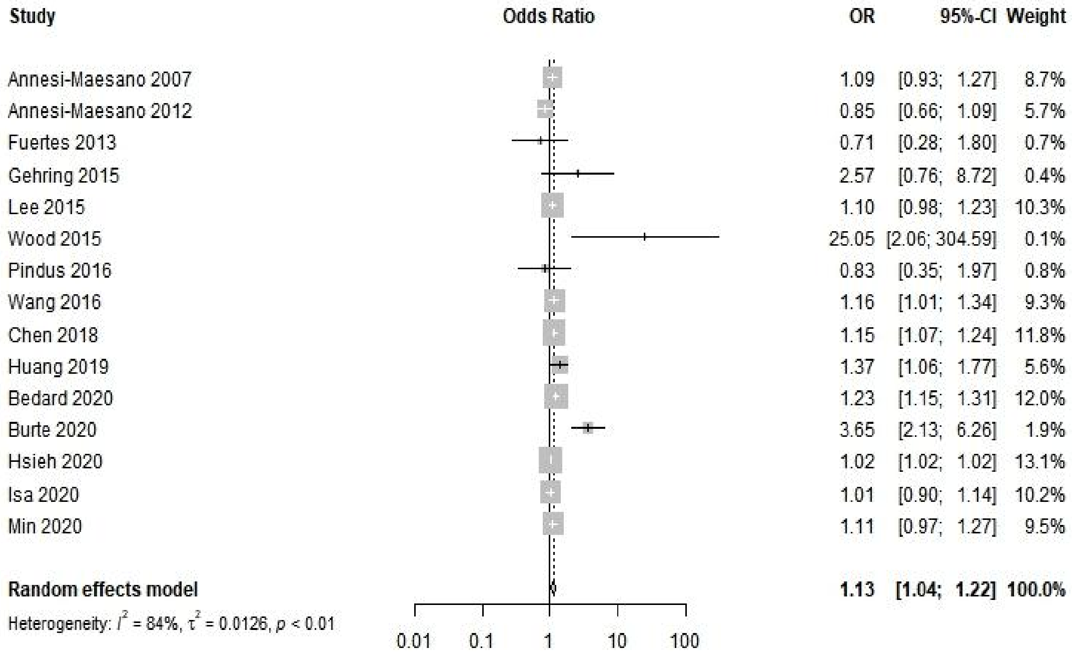

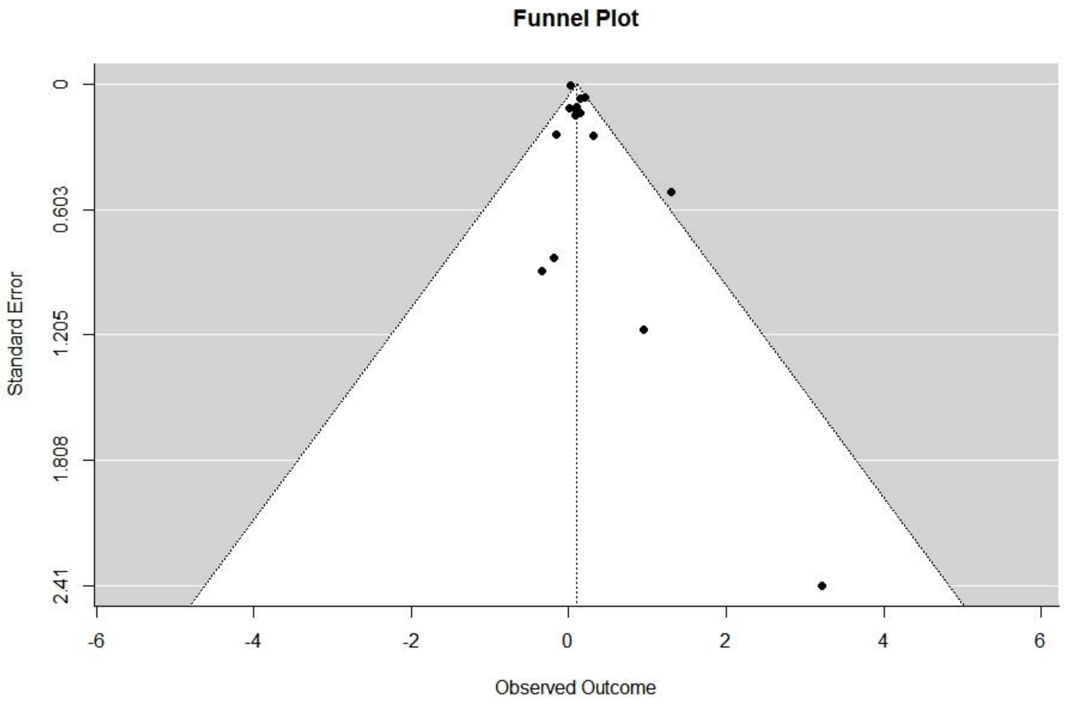

Figure S19. Random-effects meta-analysis of the association between carbon monoxide and AR (forest plot and funnel plot)

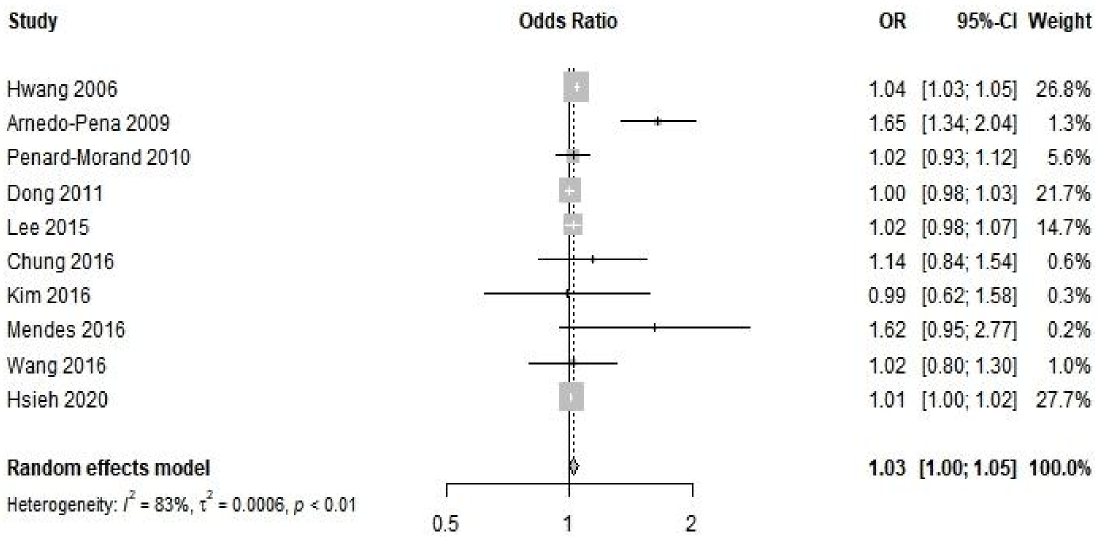

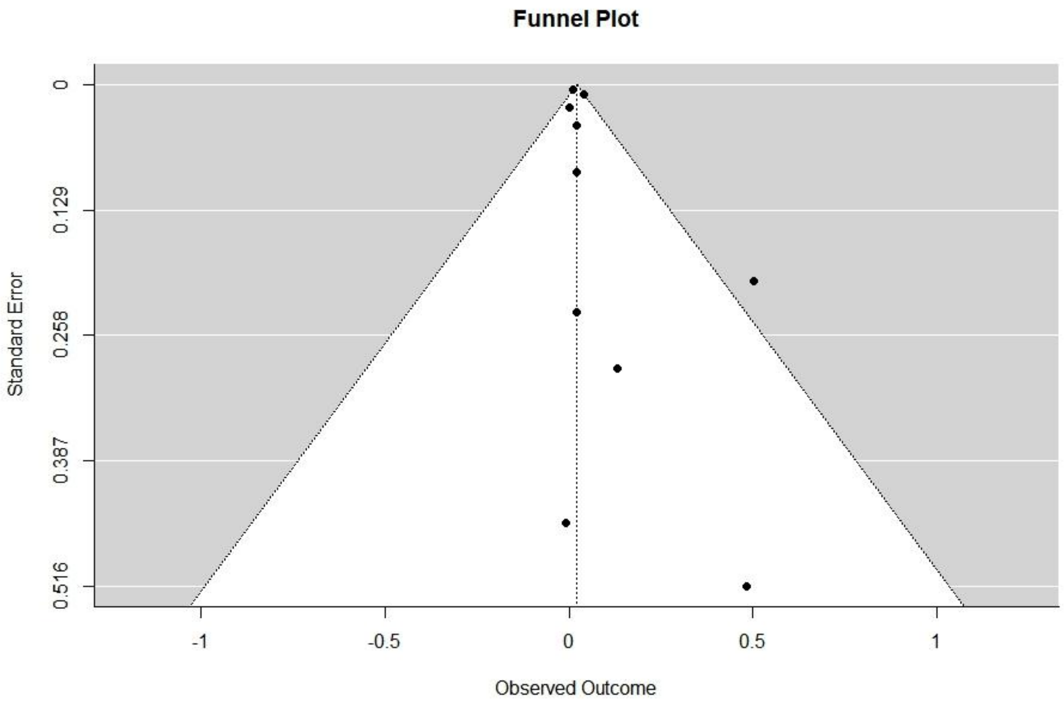

Figure S20. Random-effects meta-analysis of the association between ozone and AR (forest plot and funnel plot)

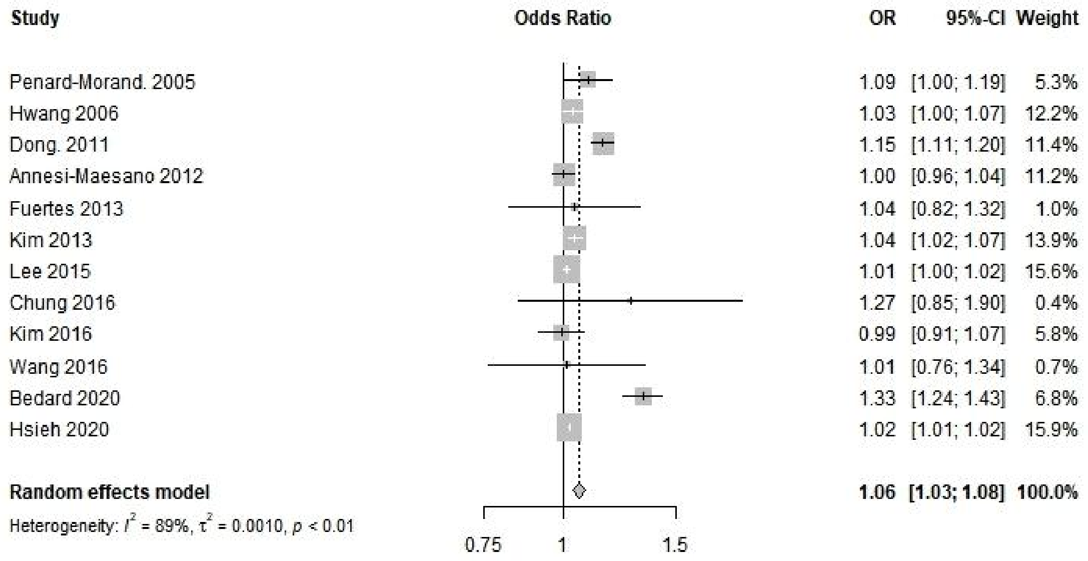

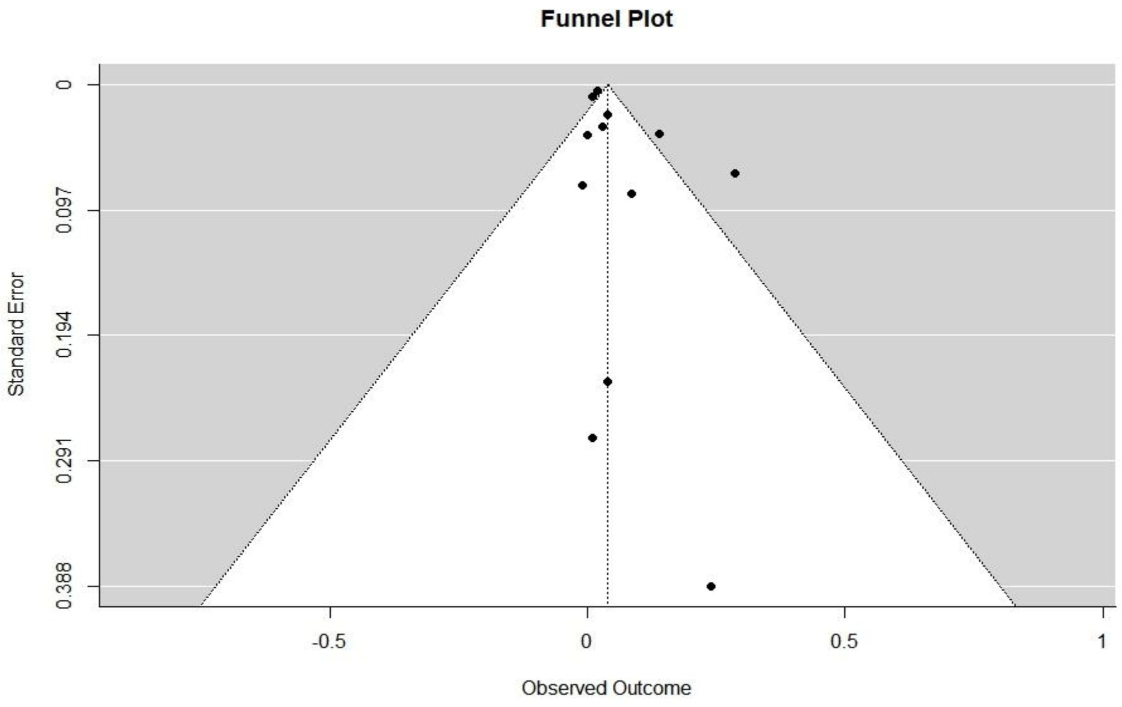

Figure S21. Random-effects meta-analysis of the association between prenatal smoke exposure and AR (forest plot and funnel plot)

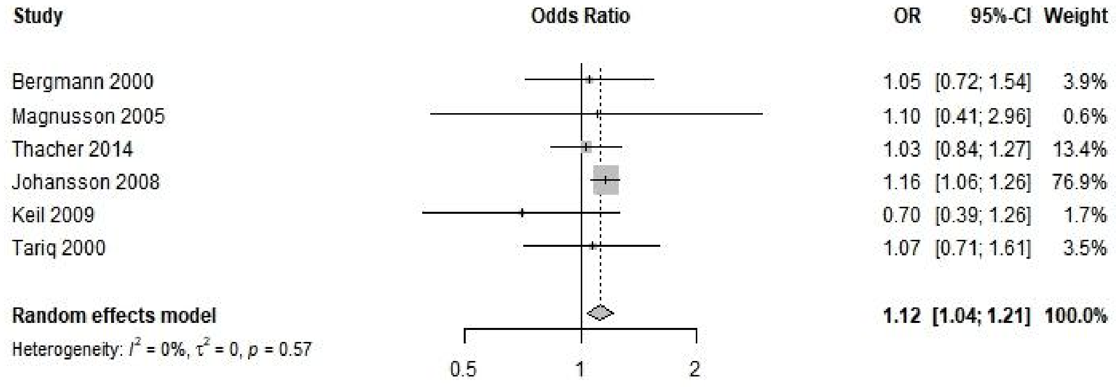

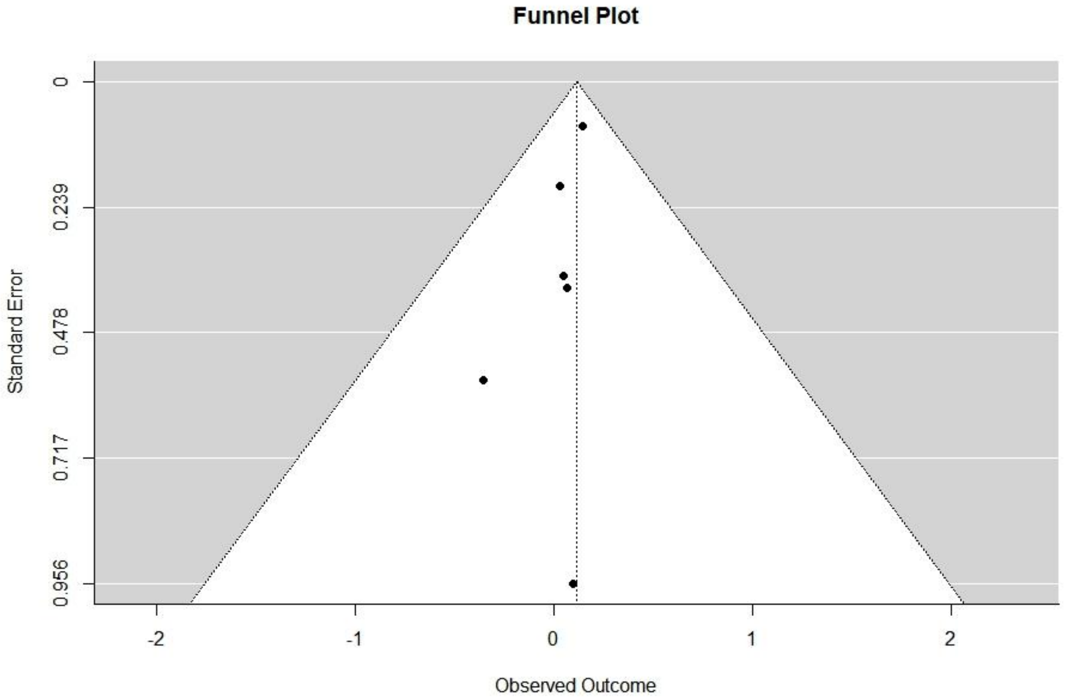

Figure S22. Random-effects meta-analysis of the association between early life food sensitization and AR (forest plot and funnel plot)

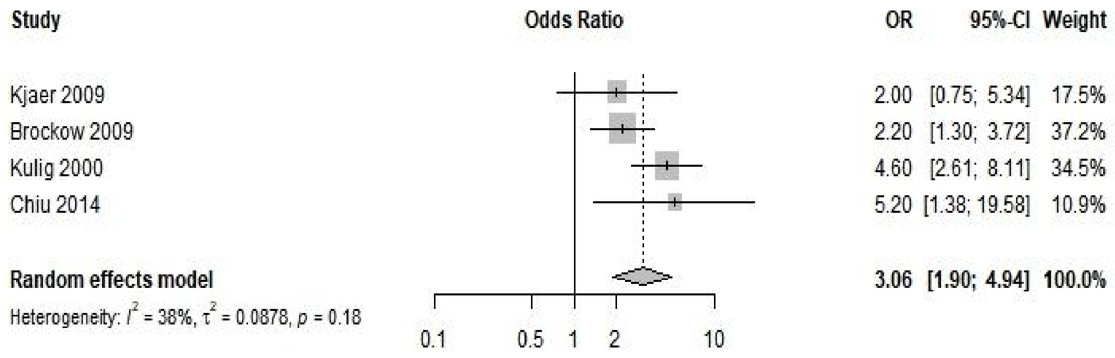

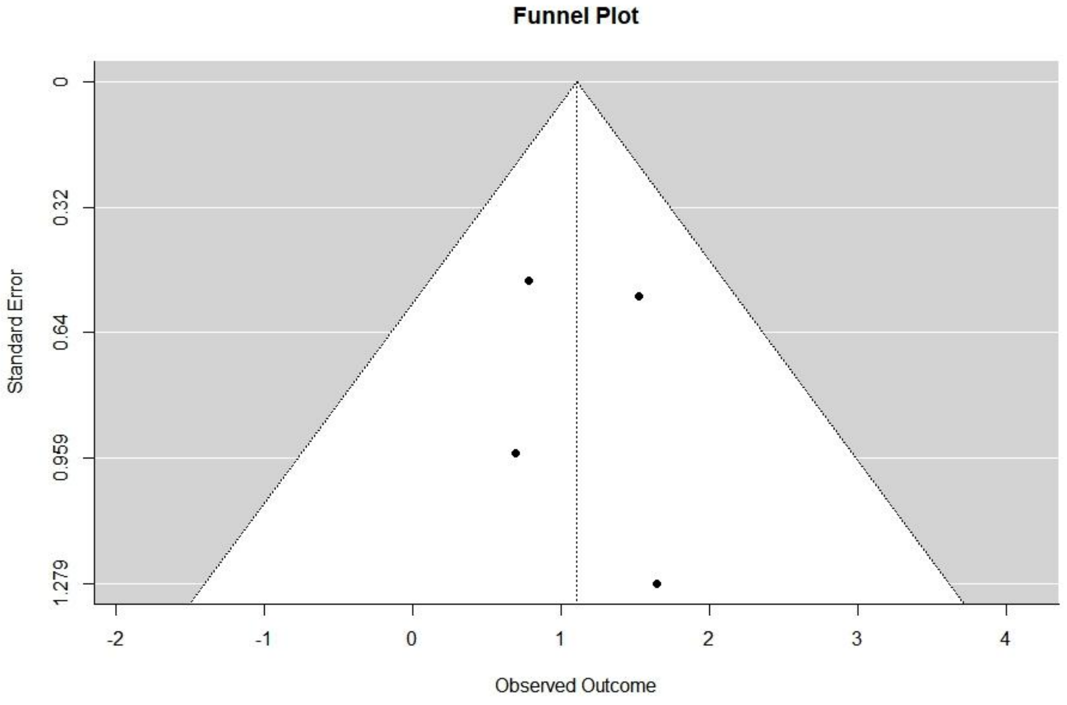

Figure S23. Random-effects meta-analysis of the association between postpartum smoke exposure and AR (forest plot and funnel plot)

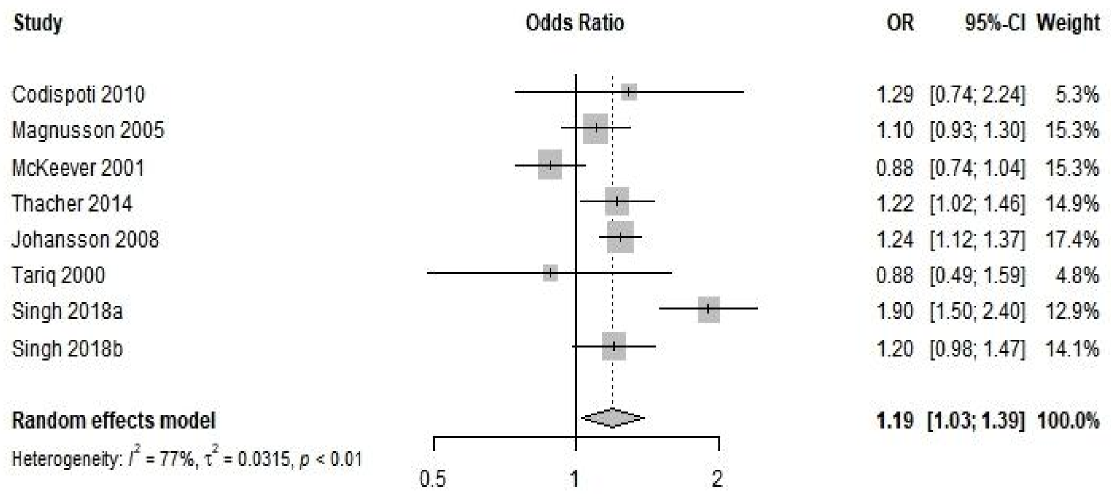

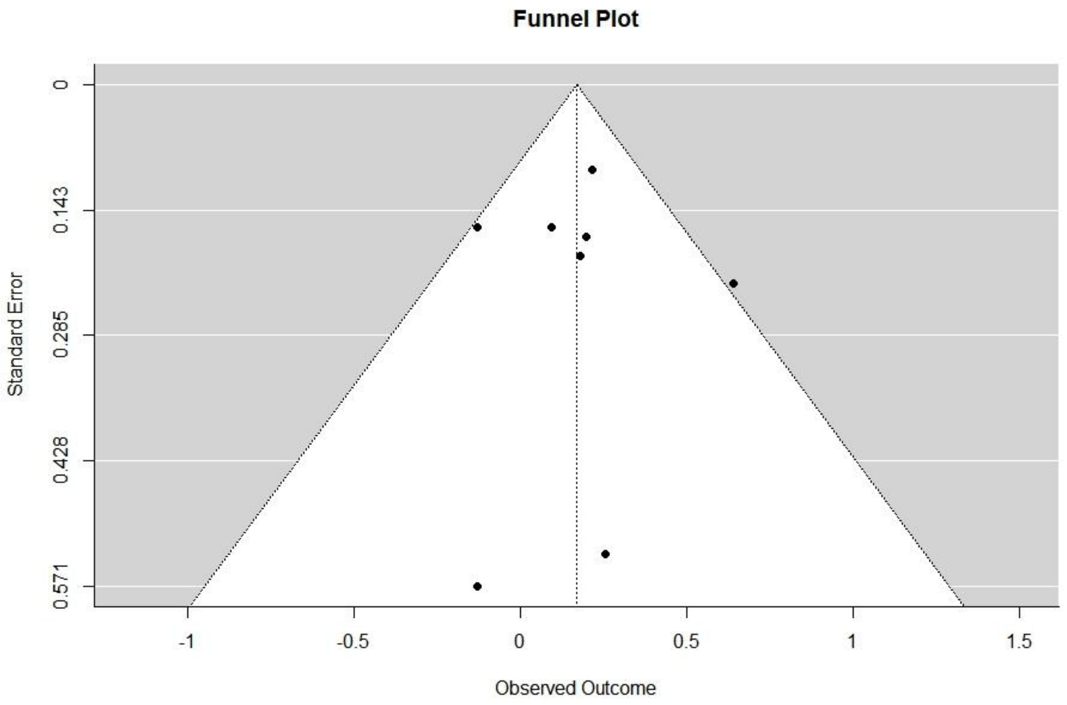

Figure S24. Random-effects meta-analysis of the association between vitamin D status and AR (forest plot and funnel plot)

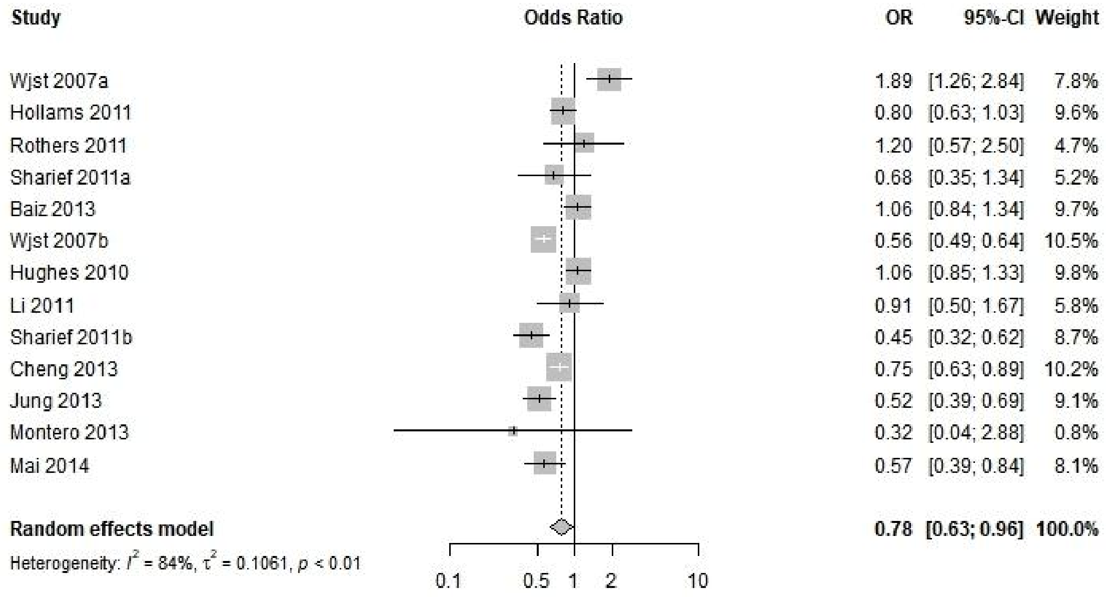

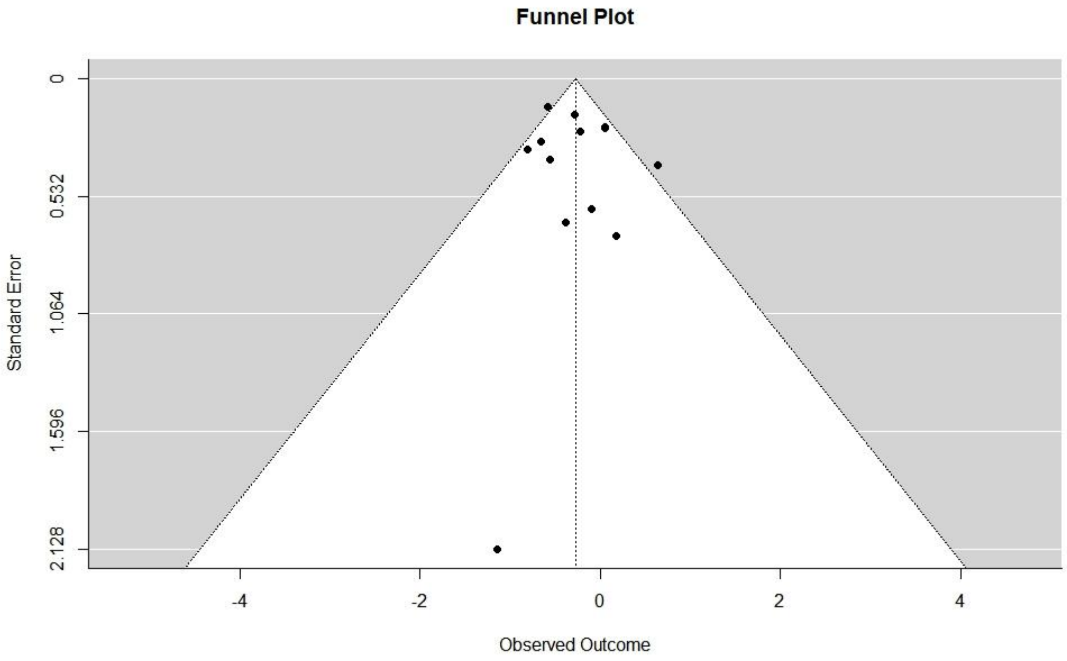

Figure S25. Random-effects meta-analysis of the association between obstructive sleep apnea and AR (forest plot and funnel plot)

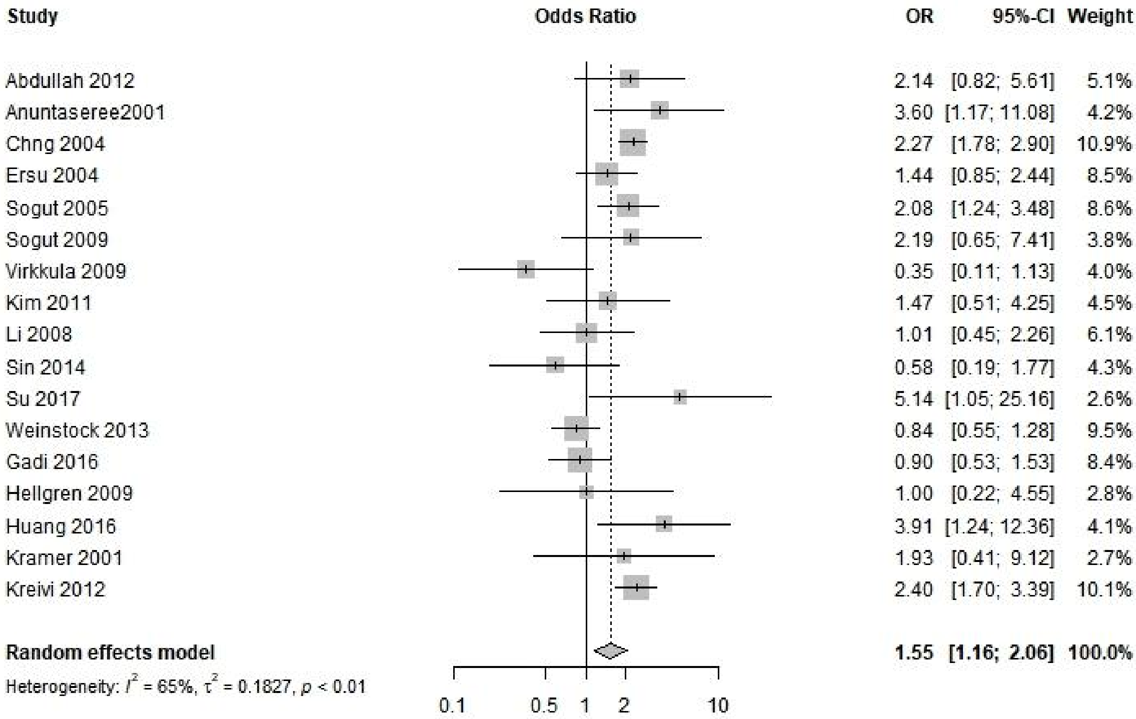

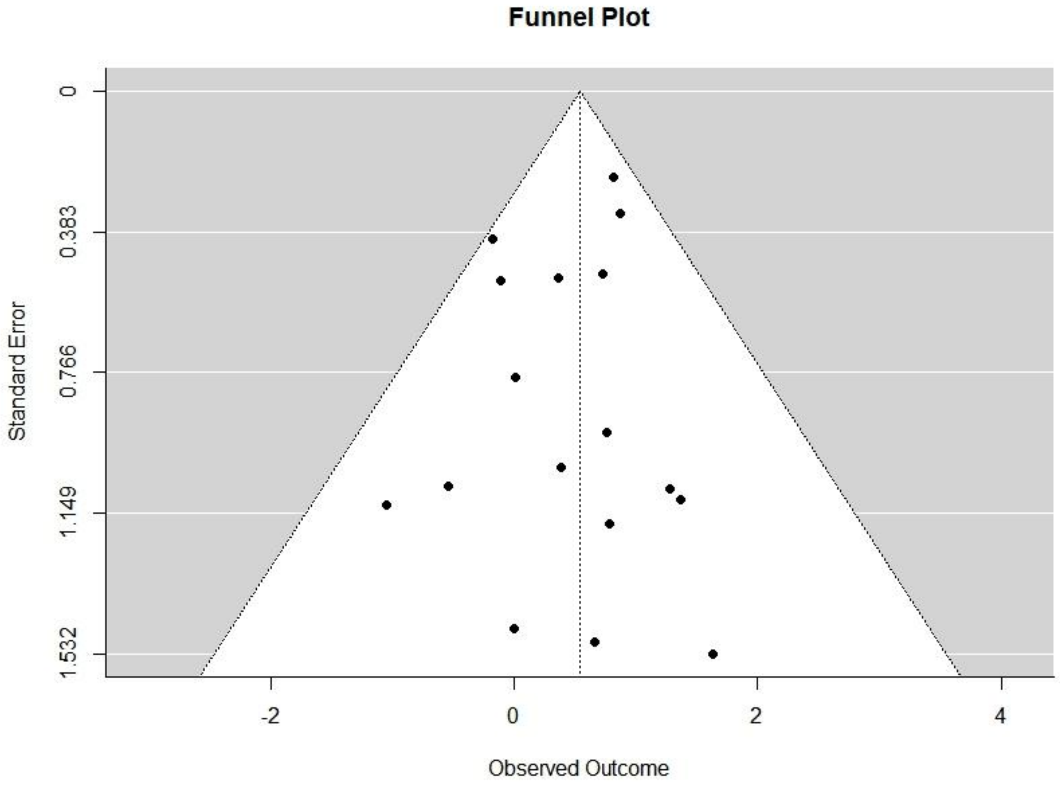

Figure S26. Random-effects meta-analysis of the association between prenatal maternal psychosocial stress and AR (forest plot and funnel plot)

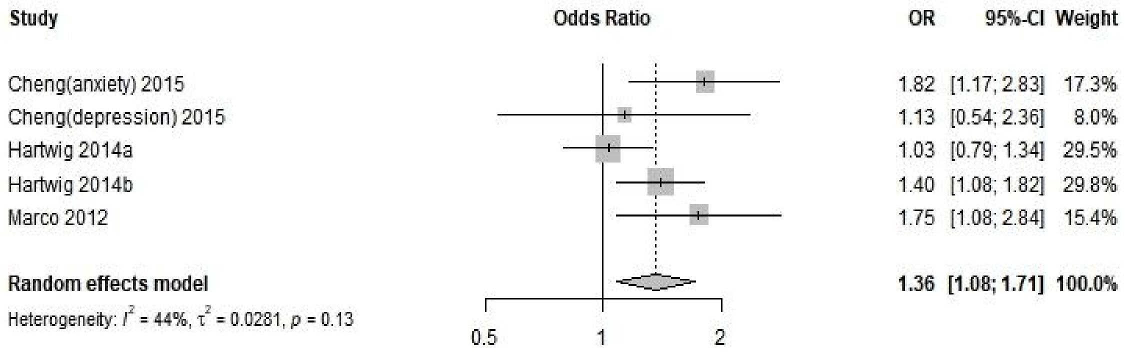

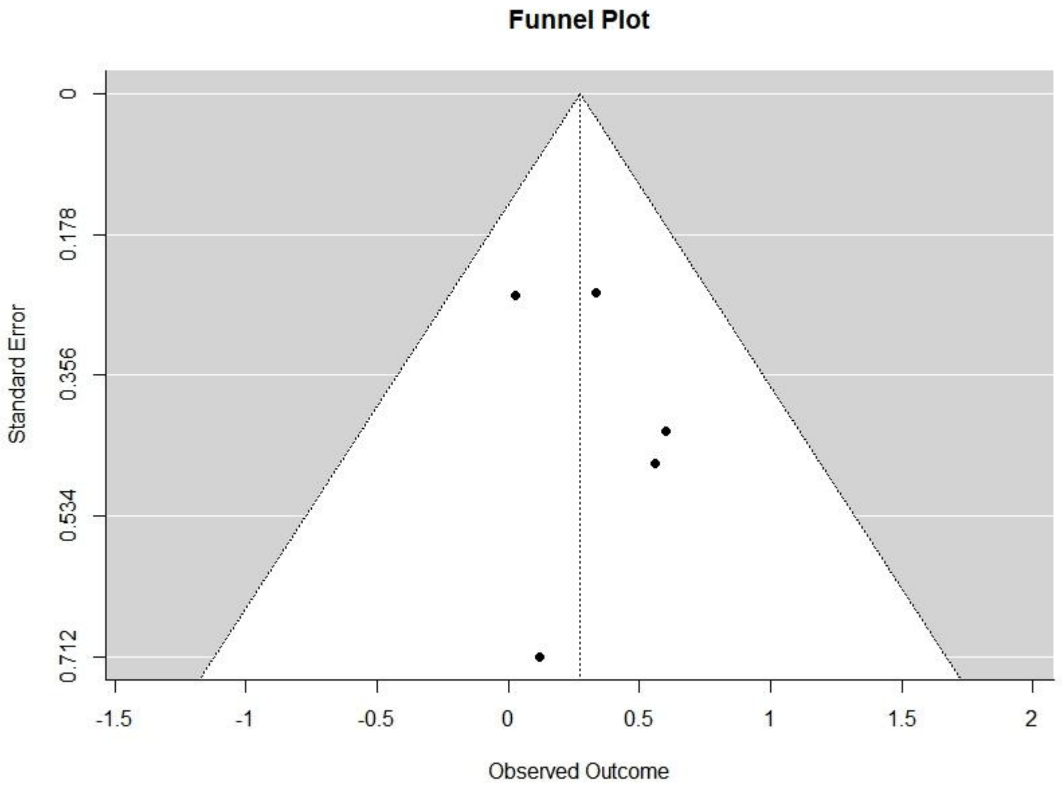

Figure S27. Random-effects meta-analysis of the association between maternal oral contraceptive pill and AR (forest plot and funnel plot)

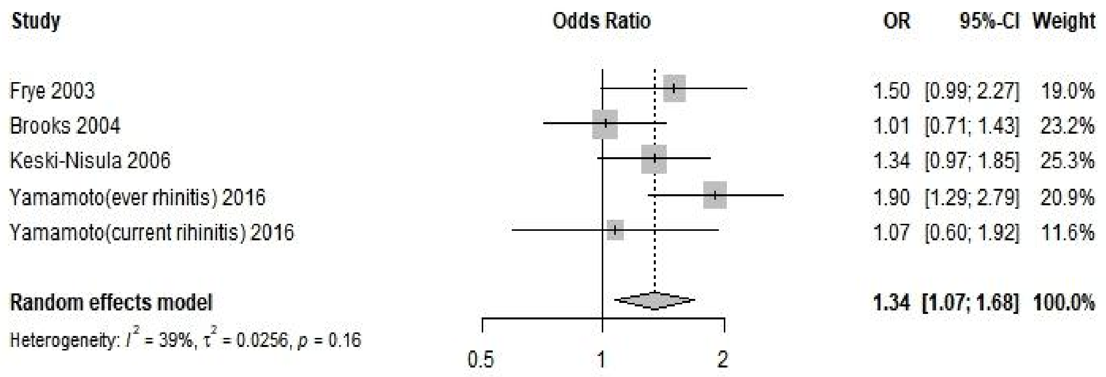

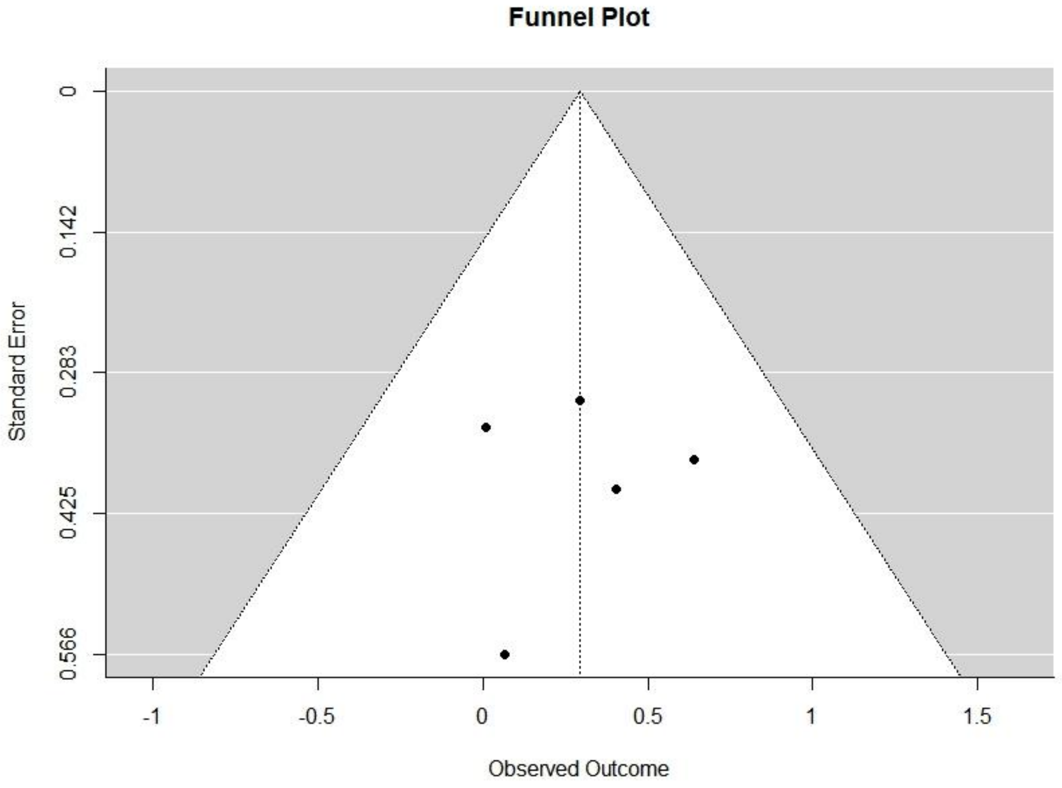

Figure S28. Random-effects meta-analysis of the association between caesarean delivery and AR (forest plot and funnel plot)

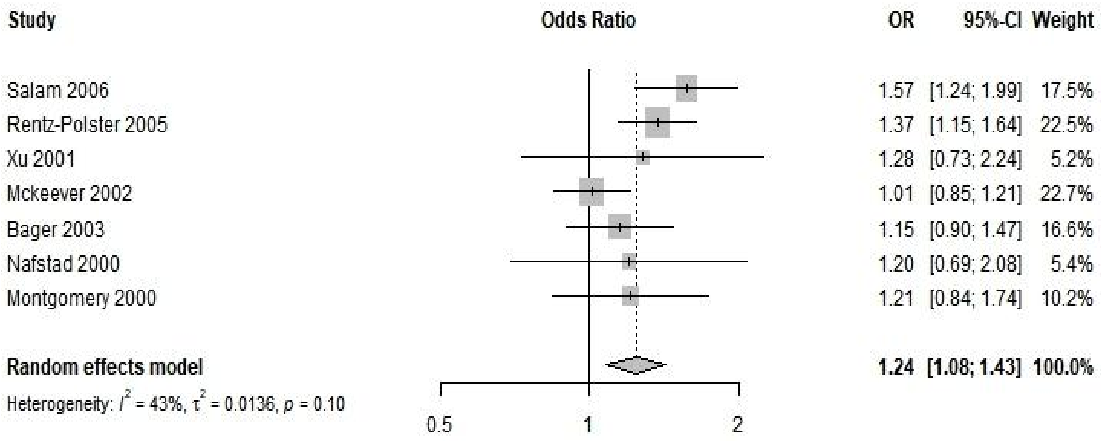

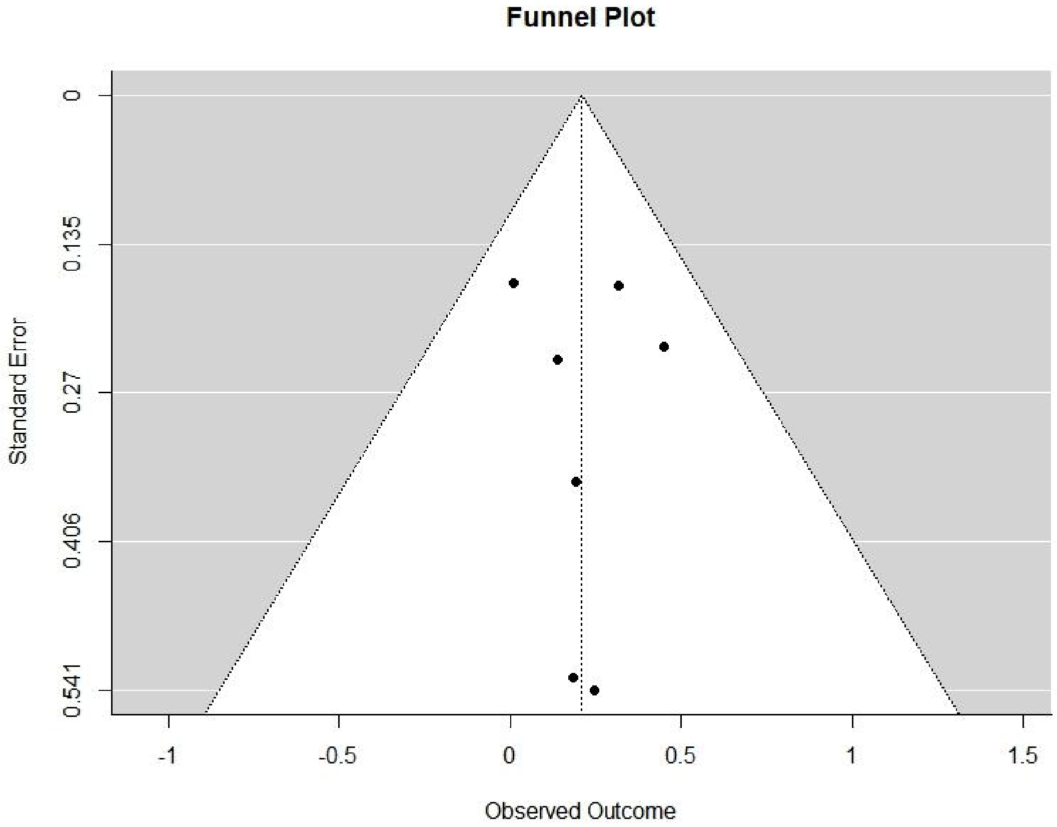

Figure S29. Random-effects meta-analysis of the association between attention deficit hyperactivity disorder and AR (forest plot and funnel plot)

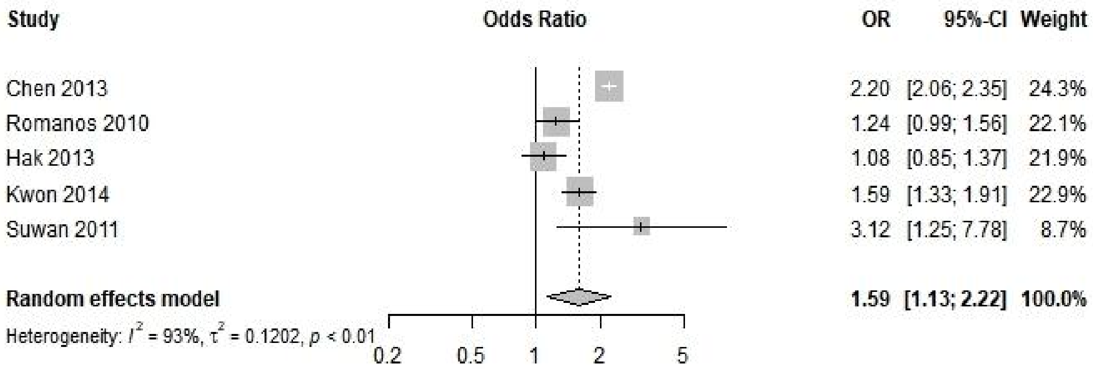

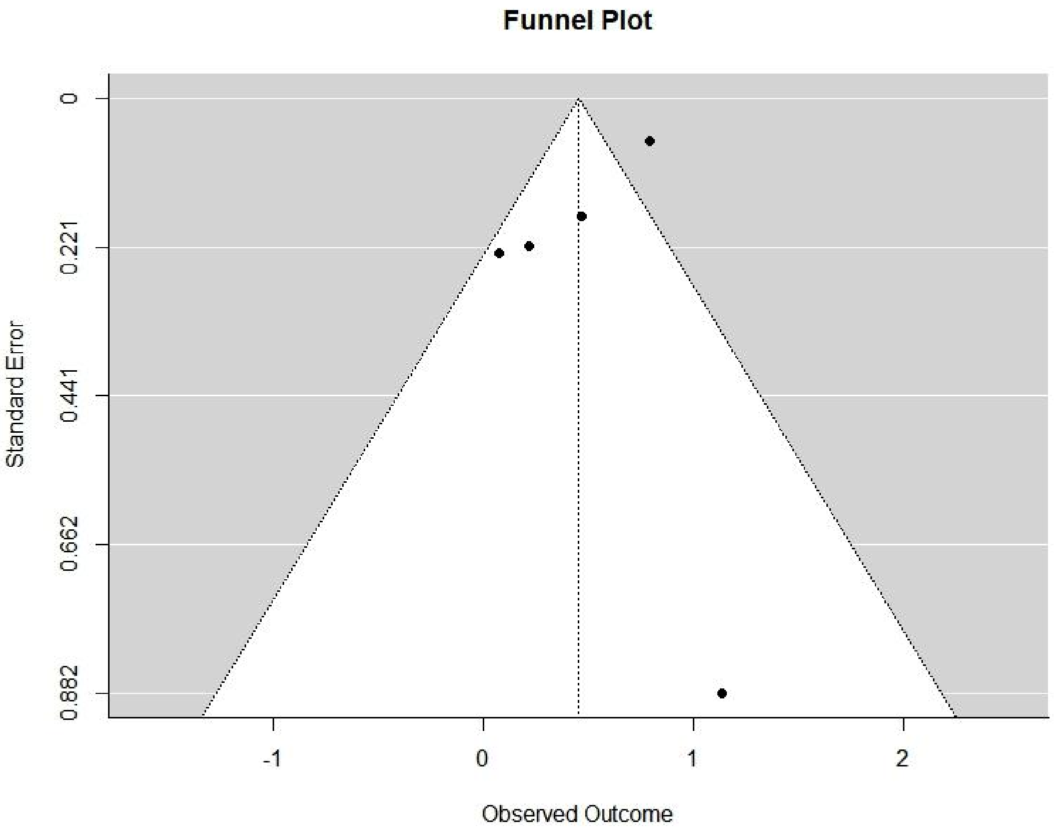

Figure S30. Random-effects meta-analysis of the association between house dust mite and AR (forest plot and funnel plot)

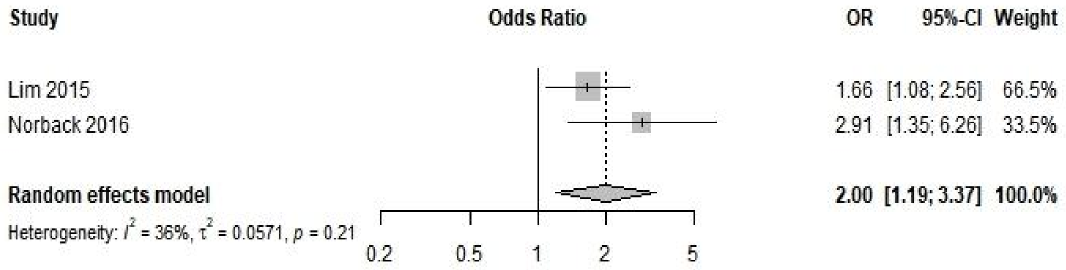

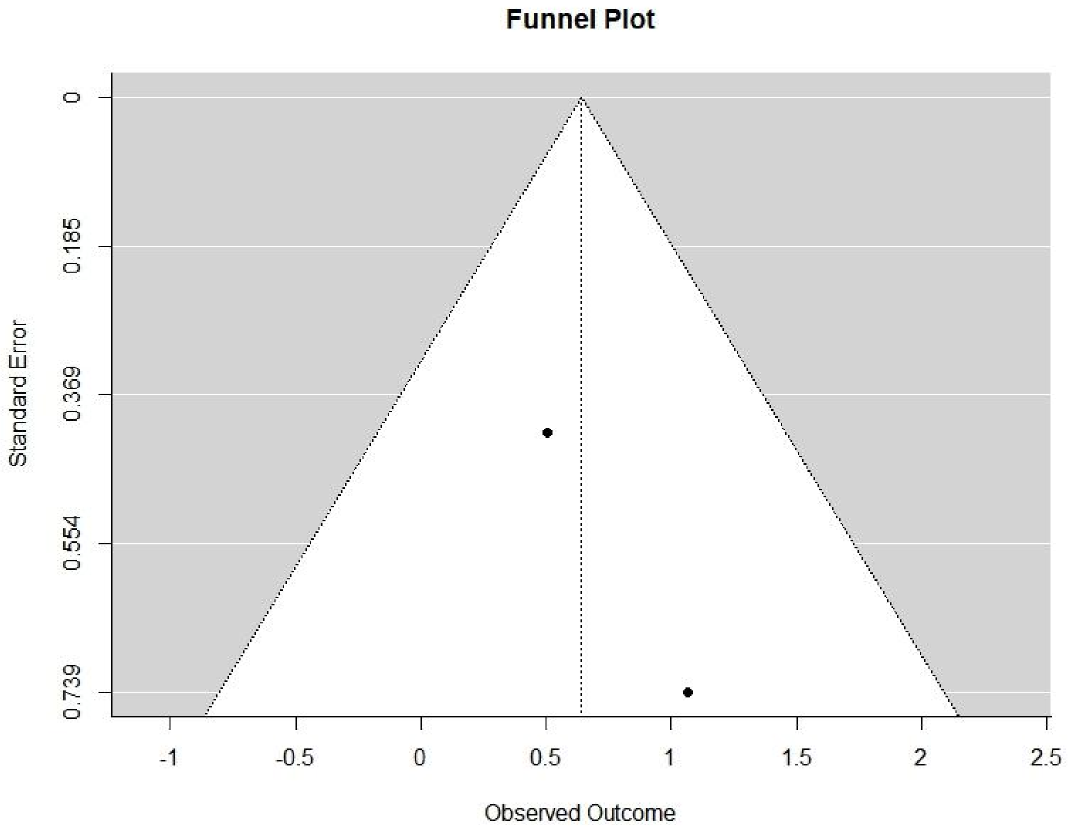

Figure S31. Random-effects meta-analysis of the association between exposure to cats and AR (forest plot and funnel plot)

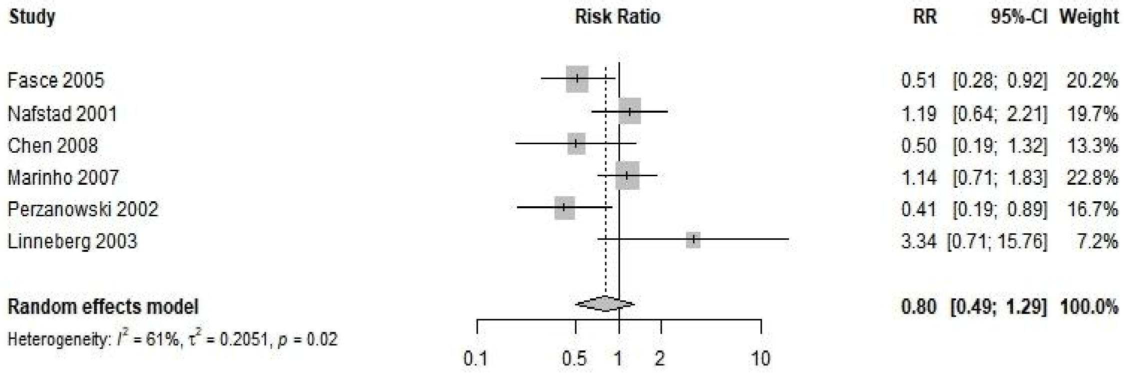

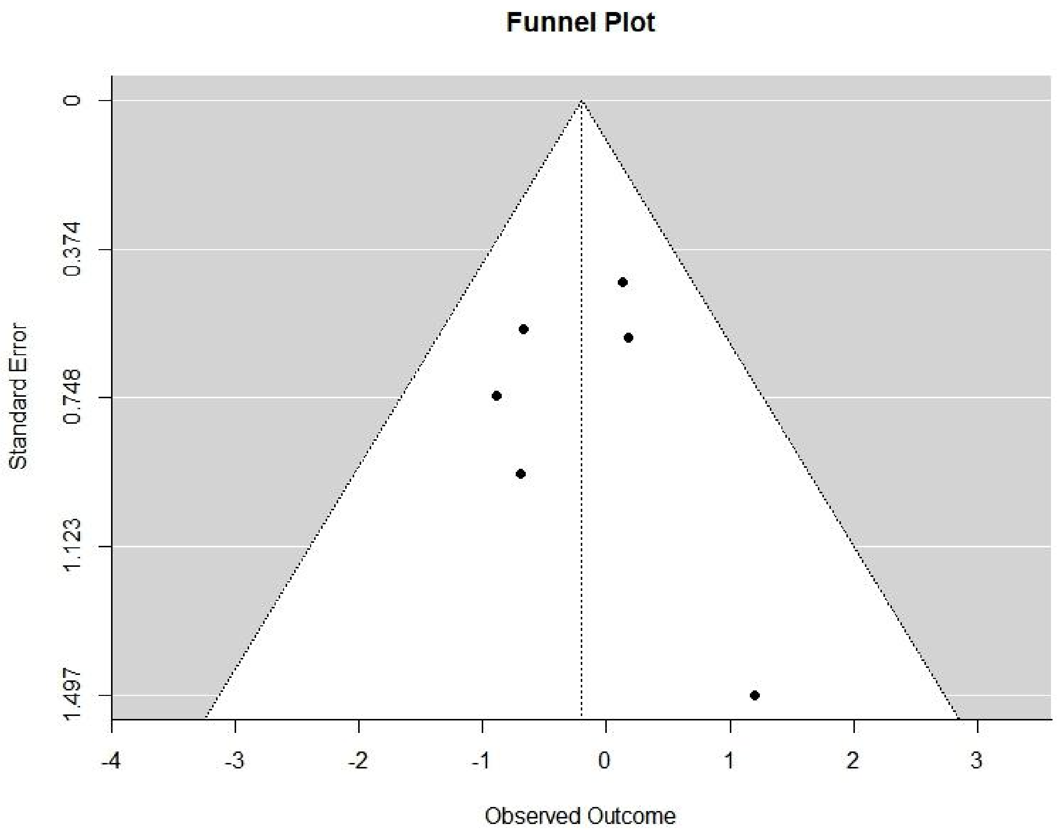

Figure S32. Random-effects meta-analysis of the association between exposure to dogs and AR (forest plot and funnel plot)

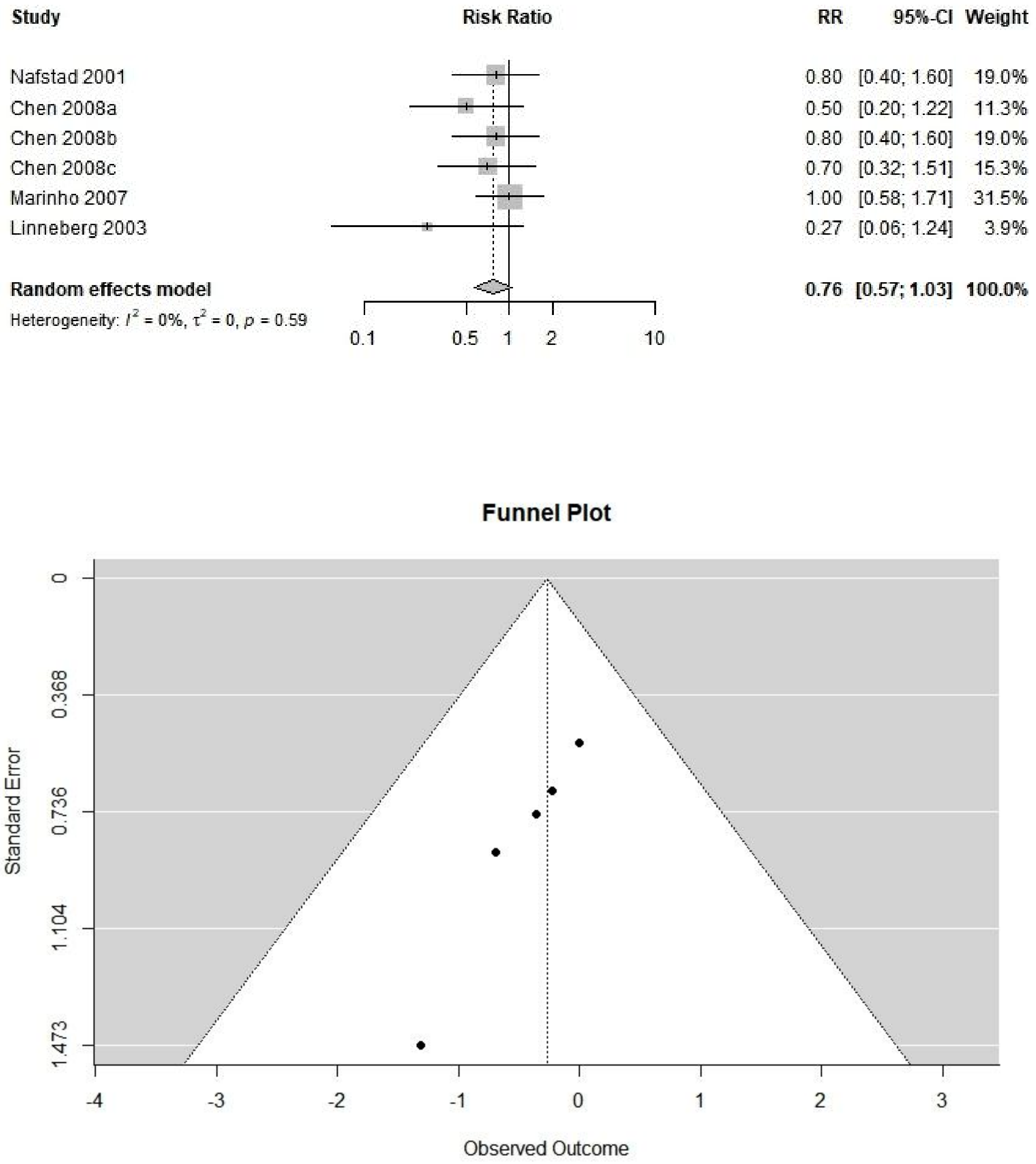

Figure S33. Random-effects meta-analysis of the association between greenness and AR (forest plot and funnel plot)

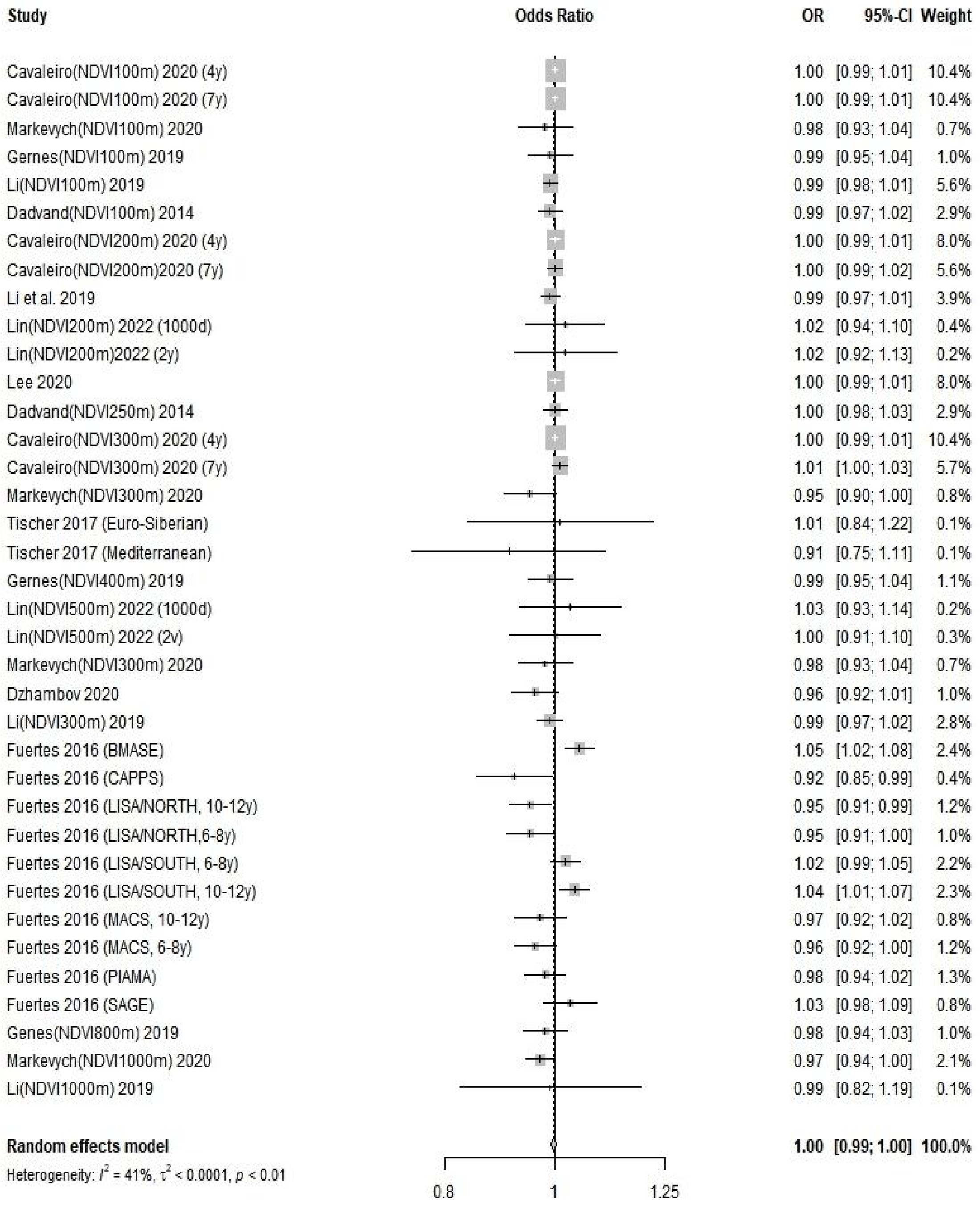

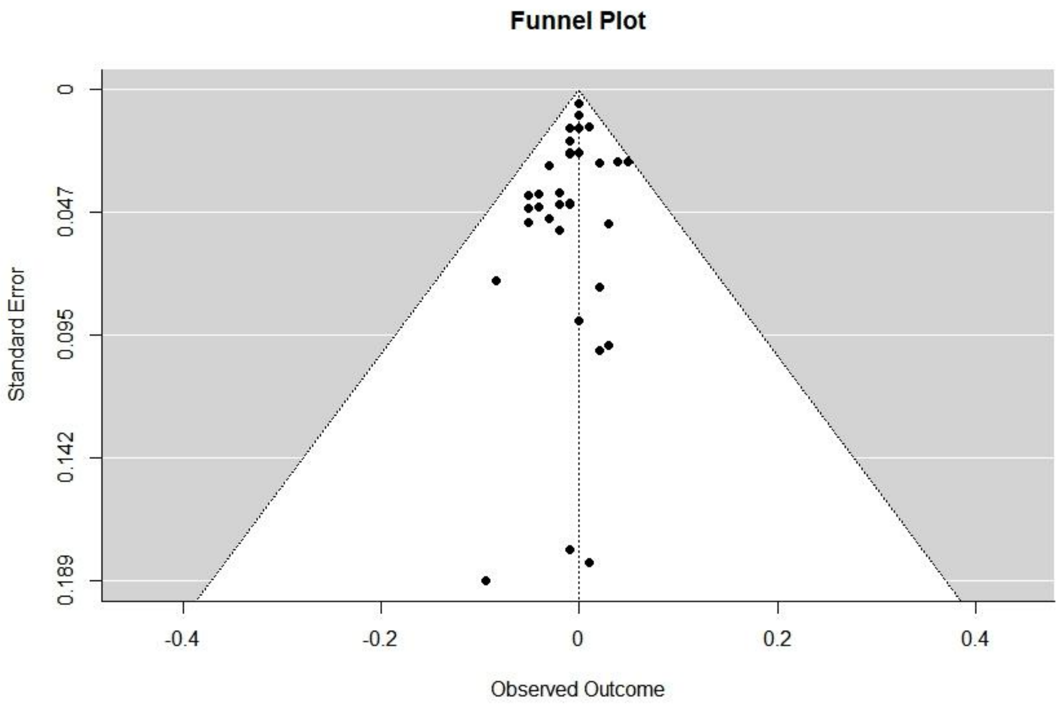
Funnel Plot
Standard Error
0
0.047
0.095
0.142
0.189
-0.4
-0.2
0
0.2
0.4
Observed Outcome

Figure S34. Random-effects meta-analysis of the association between maternal fish intake during pregnancy and AR (forest plot and funnel plot)

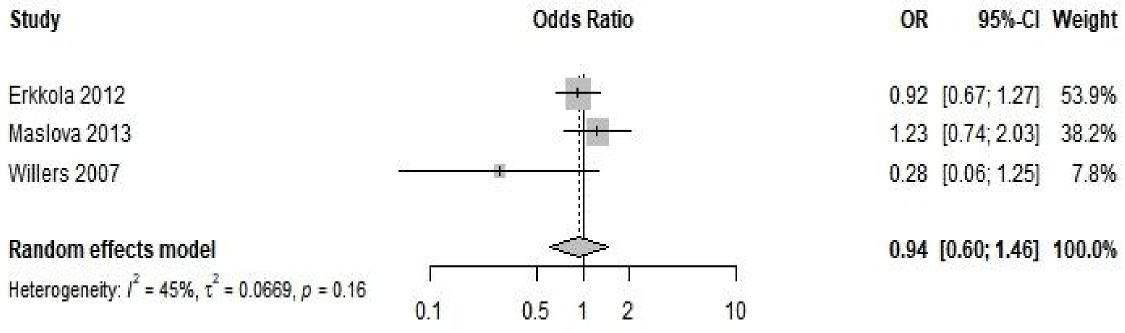

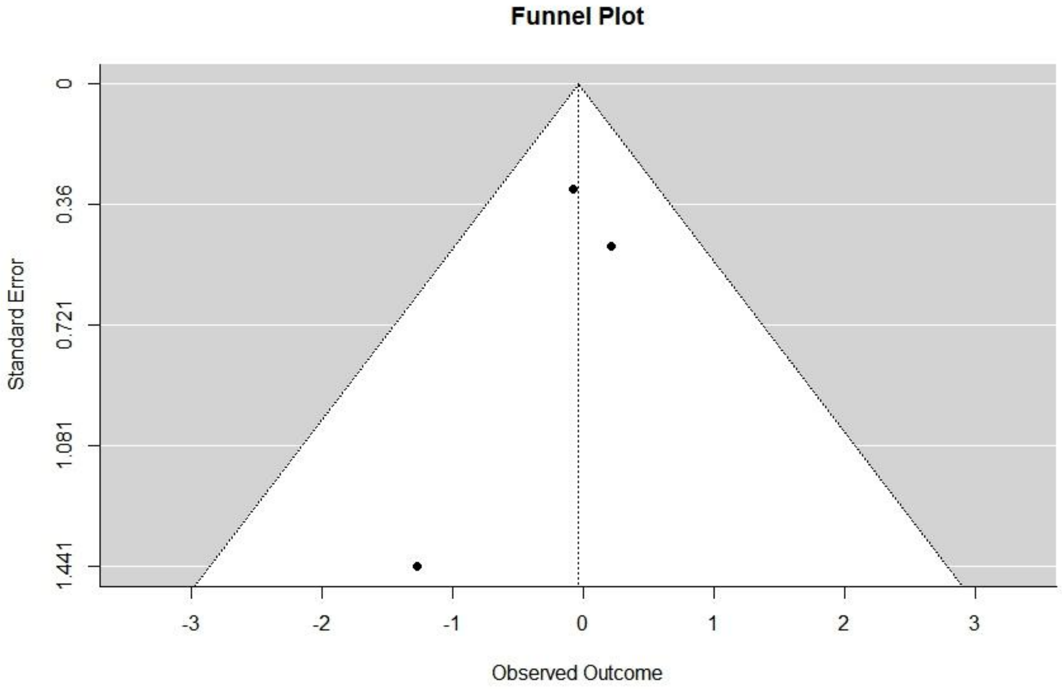

Figure S35. Random-effects meta-analysis of the association between prenatal exposure to vitamin D and AR (forest plot and funnel plot)

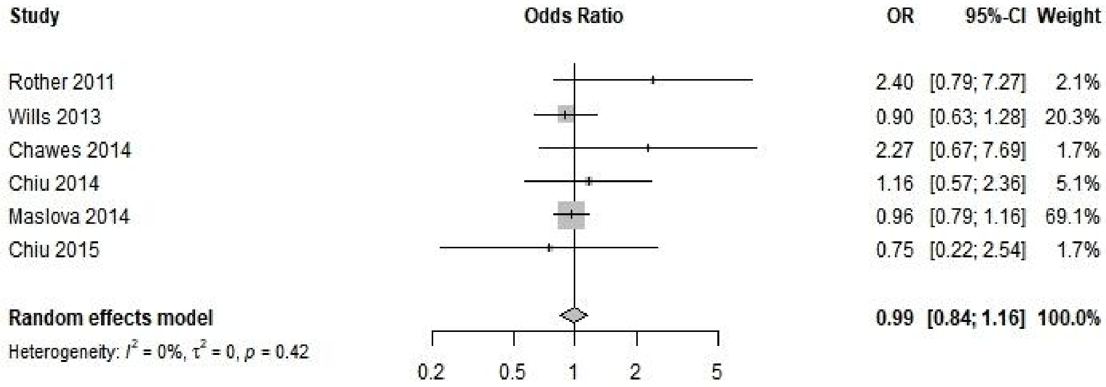

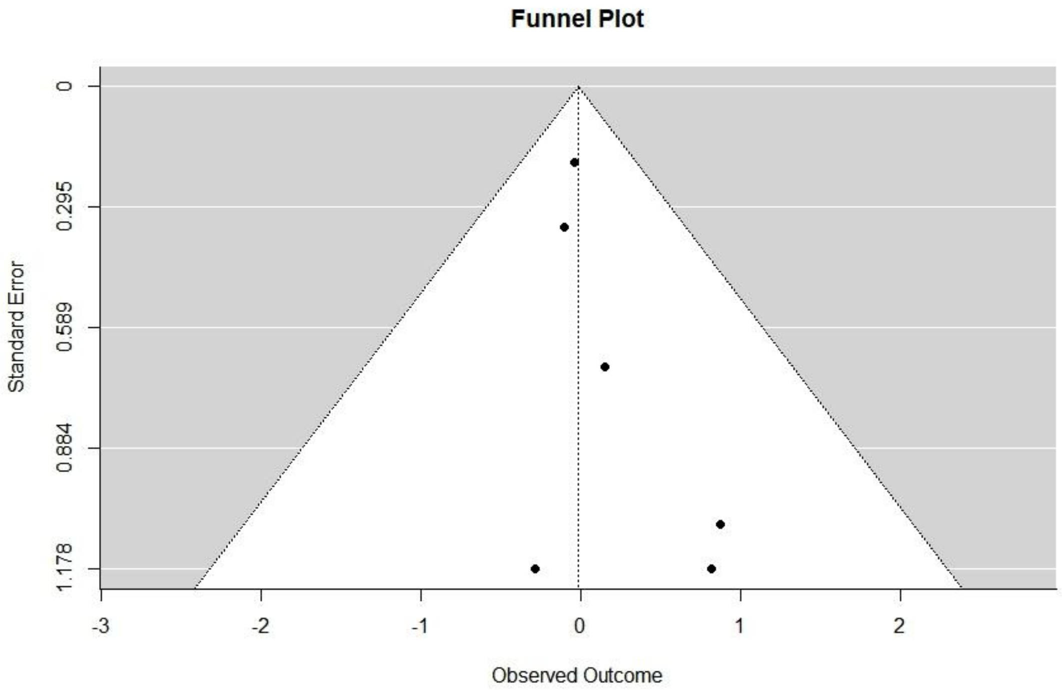

Figure S36. Random-effects meta-analysis of the association between neonatal jaundice and AR (forest plot and funnel plot)

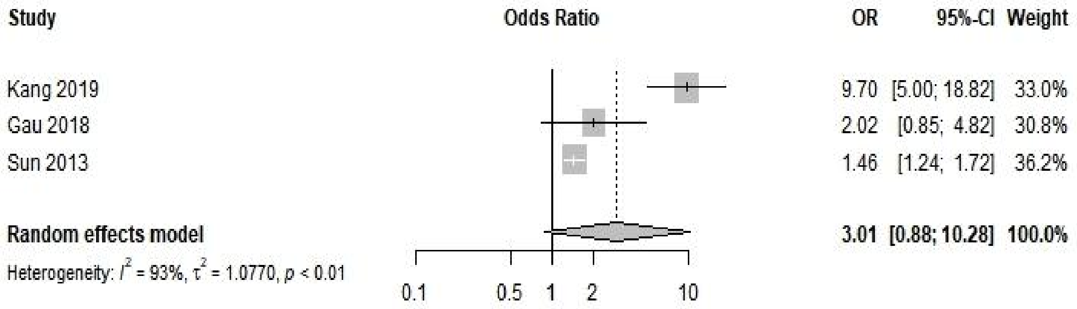

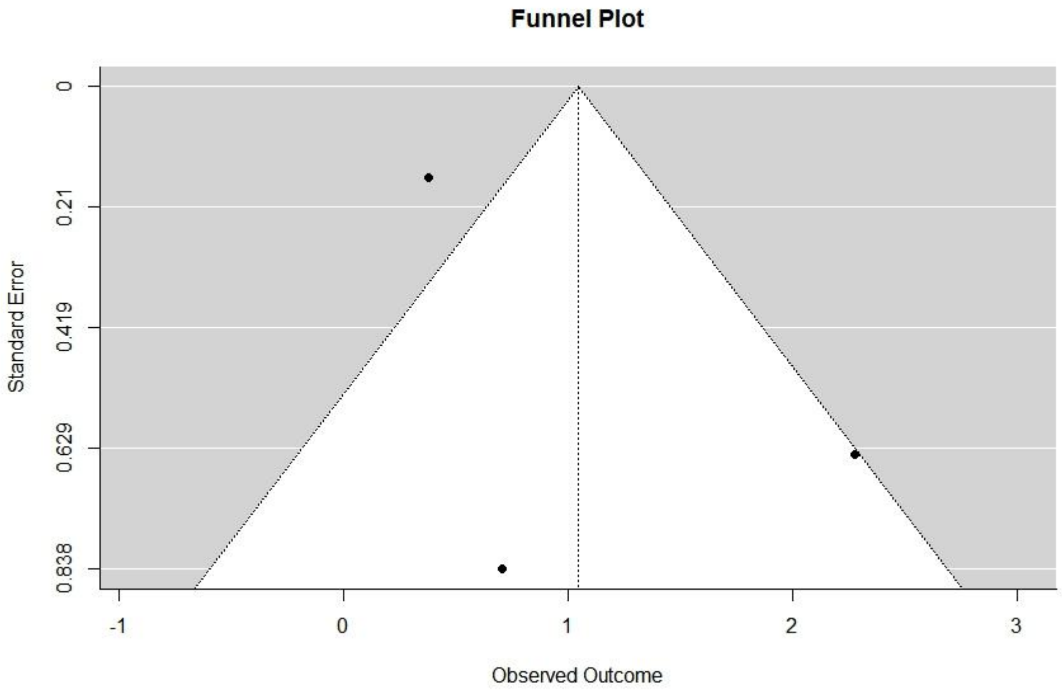

Figure S37. Random-effects meta-analysis of the association between phototherapy and AR (forest plot and funnel plot)

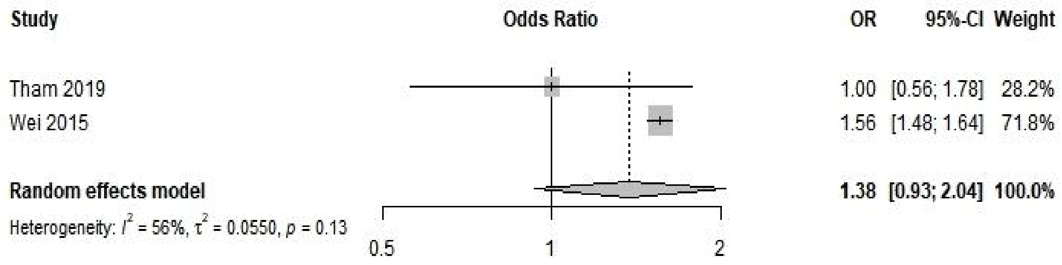

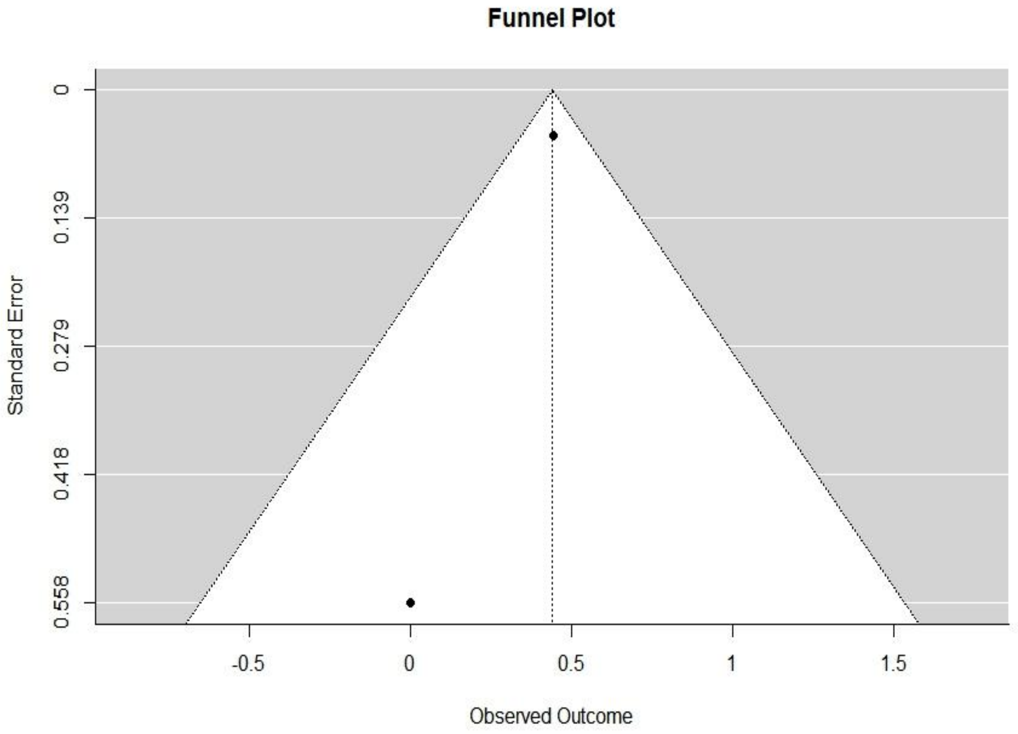

Figure S38. Random-effects meta-analysis of the association between active exposure to tobacco smoking and AR (forest plot and funnel plot)

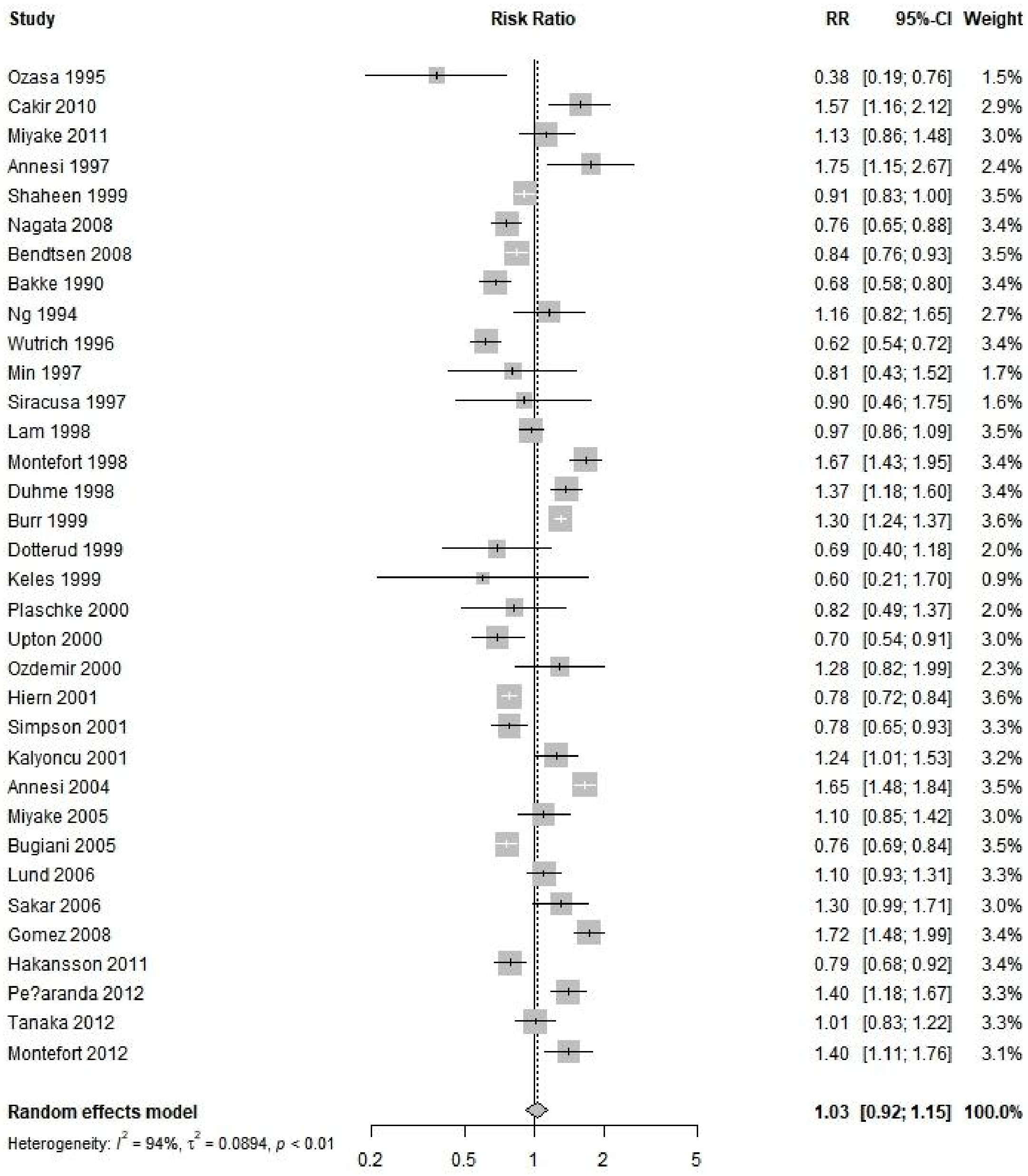

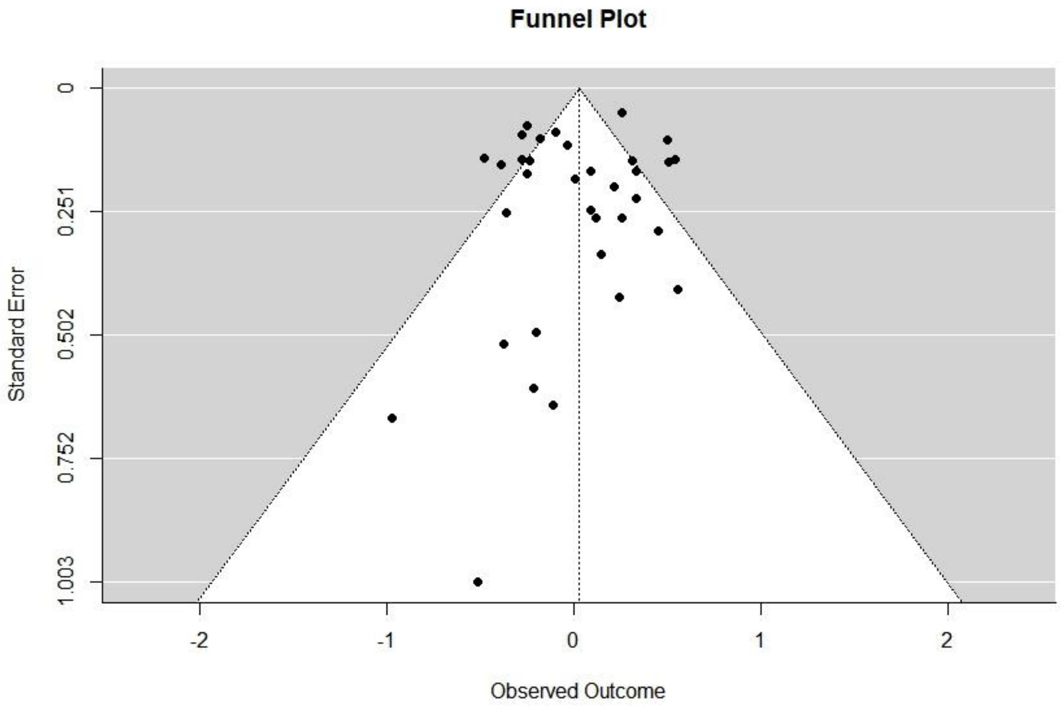
Funnel Plot
Standard Error
0
0.251
0.502
0.752
1.003
-2
-1
0
1
2
Observed Outcome

Figure S39. Random-effects meta-analysis of the association between childhood type 1 diabetes and AR (forest plot and funnel plot)

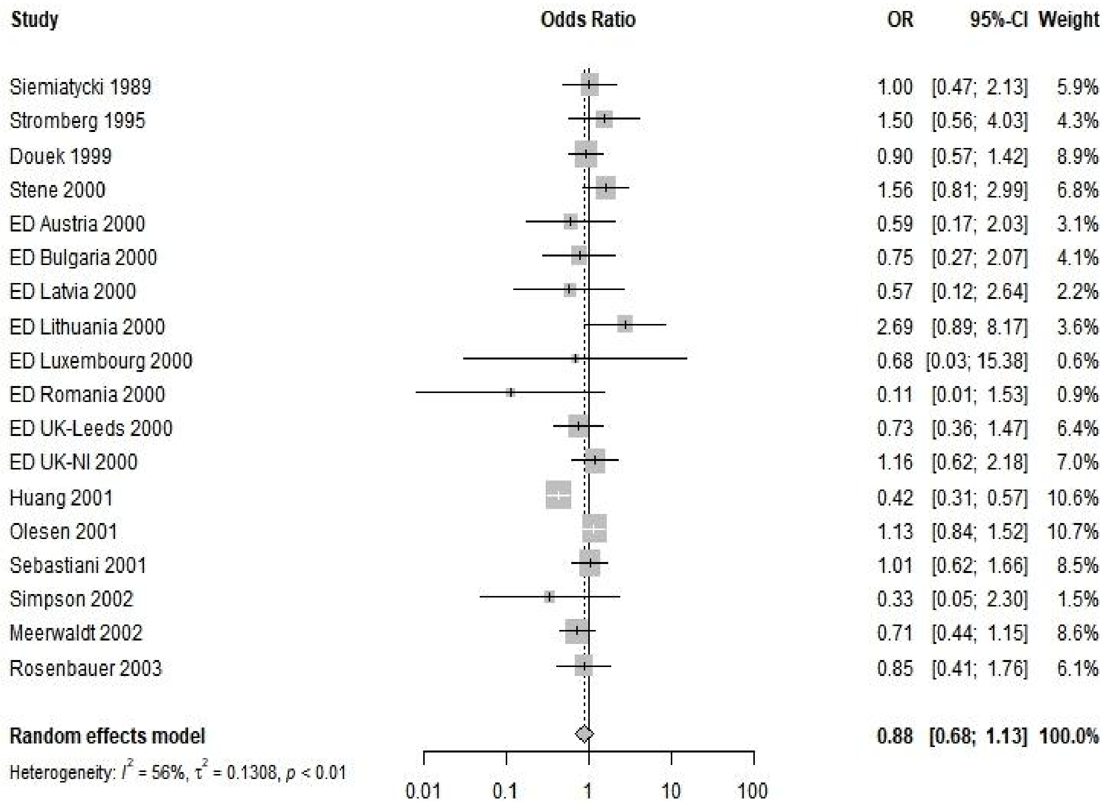

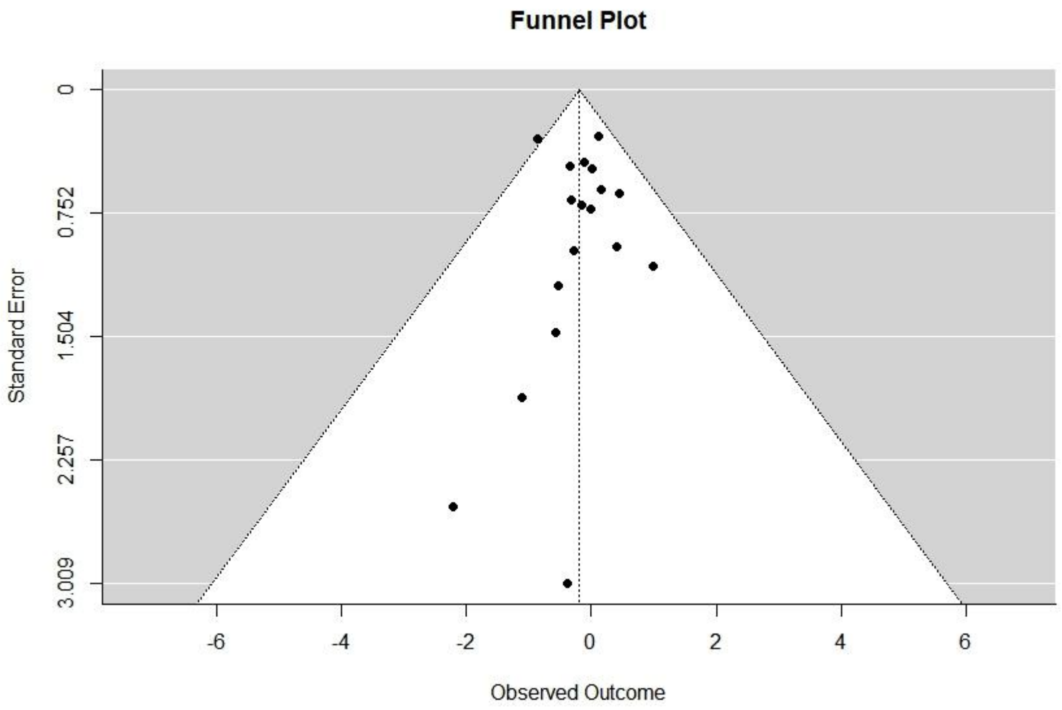

Figure S40. Random-effects meta-analysis of the association between children and adolescents exposed to pesticides and AR (forest plot and funnel plot)

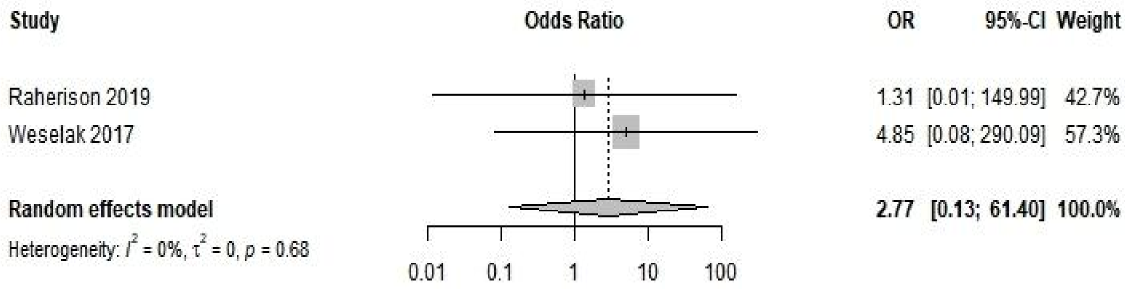

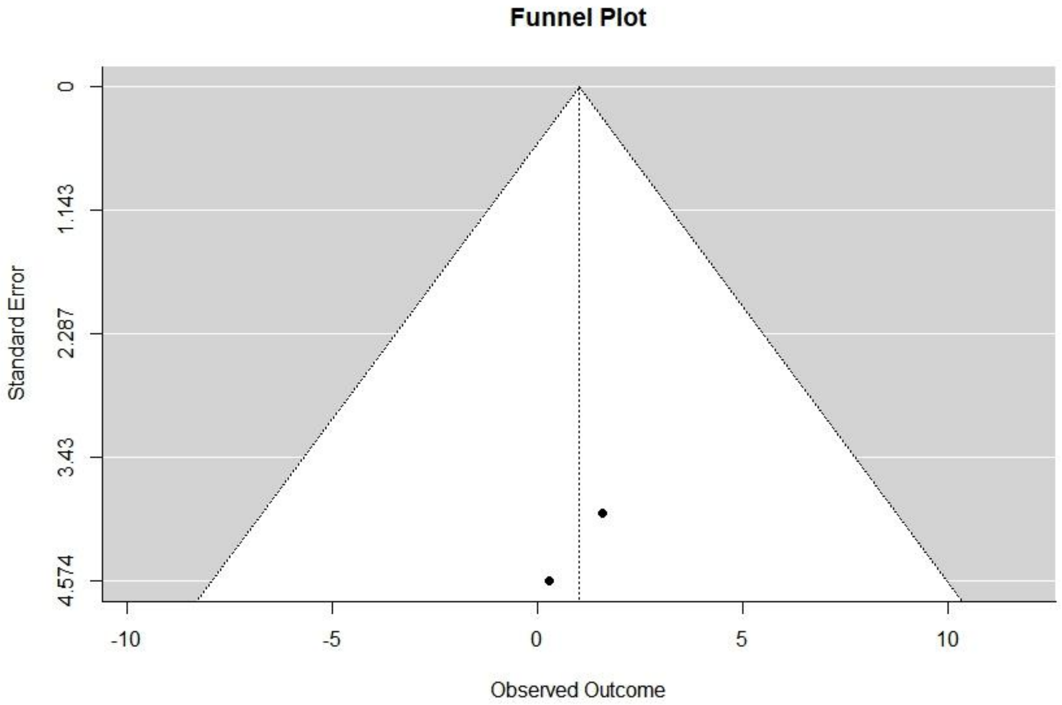

Figure S41. Random-effects meta-analysis of the association between exposure to perfluoroalkyl substances and AR (forest plot and funnel plot)

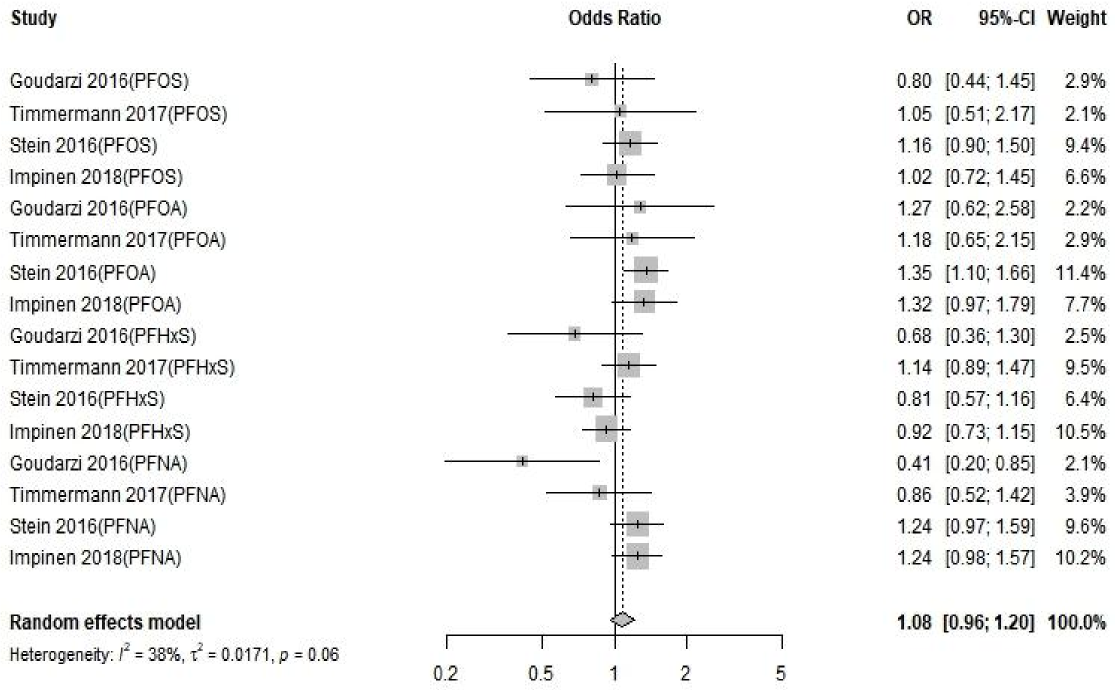

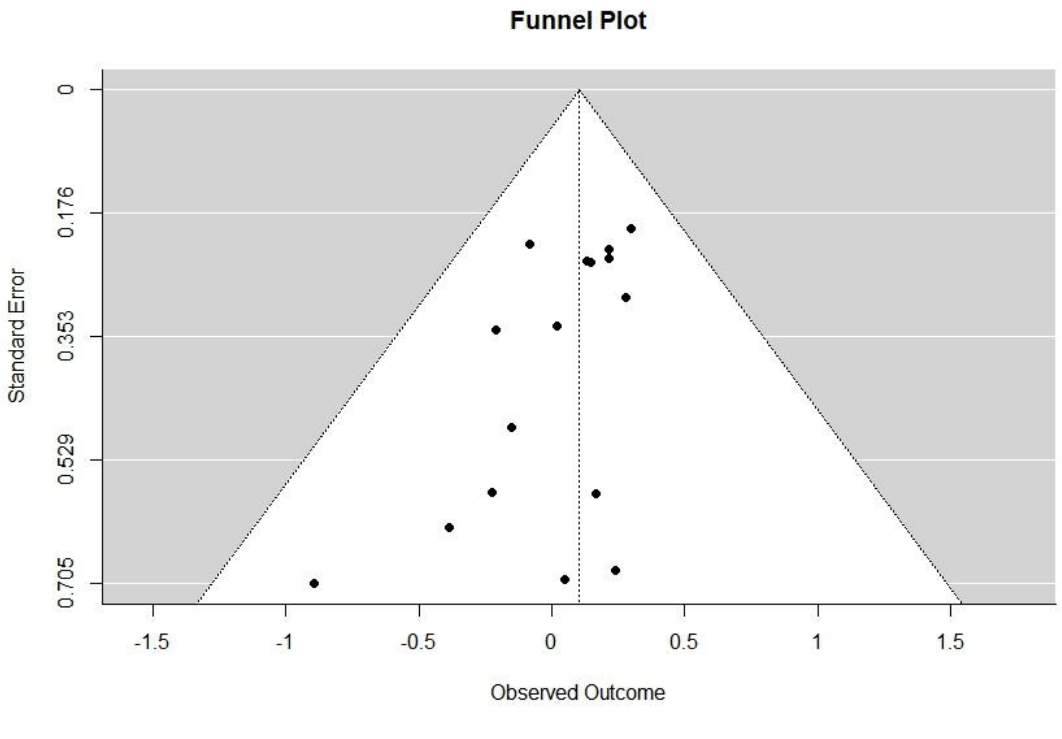

Figure S42. Random-effects meta-analysis of the association between multiple sclerosis and AR (forest plot and funnel plot)

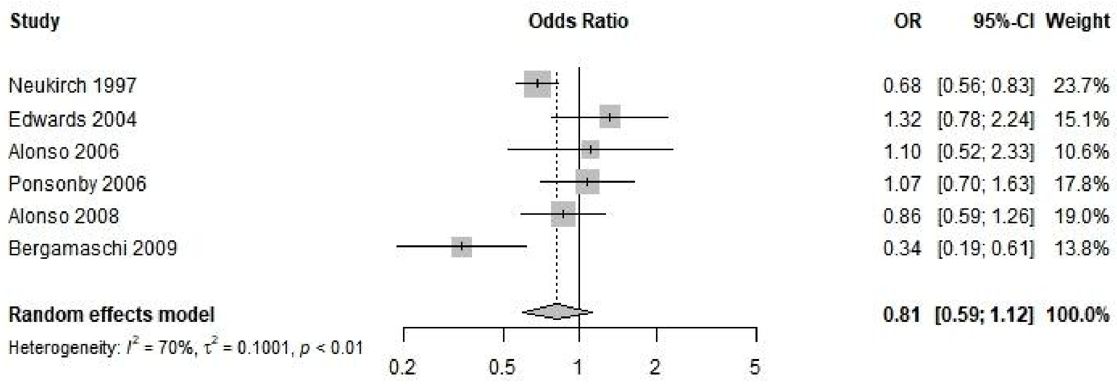

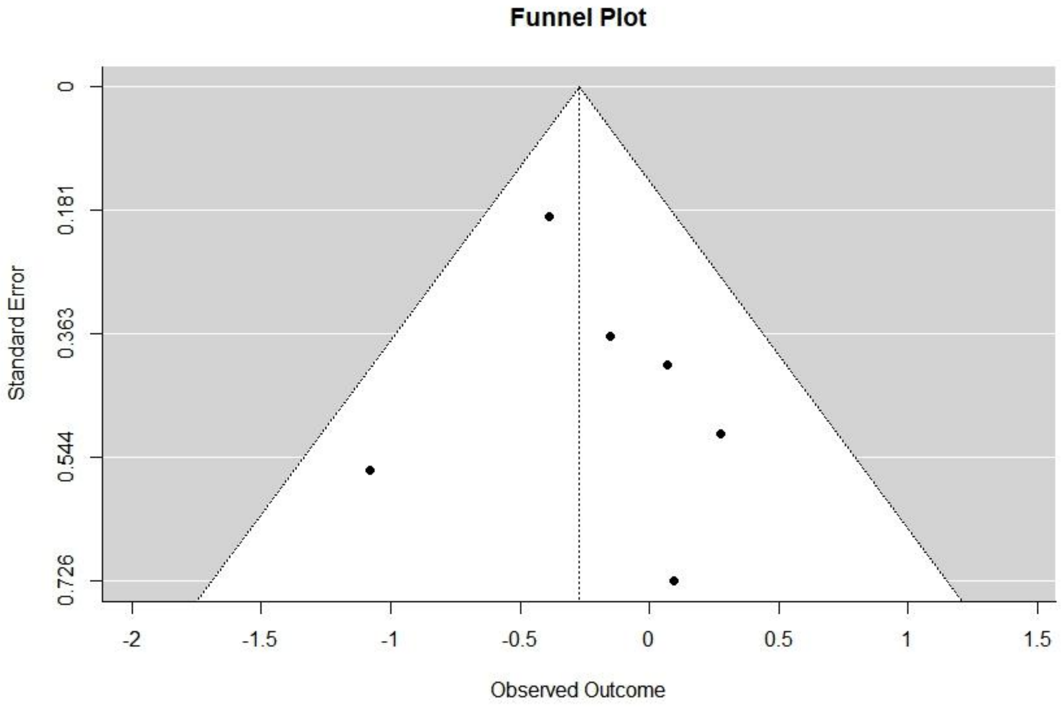

Figure S43. Random-effects meta-analysis of the association between indoor microbial aerosols exposures and AR (forest plot and funnel plot)

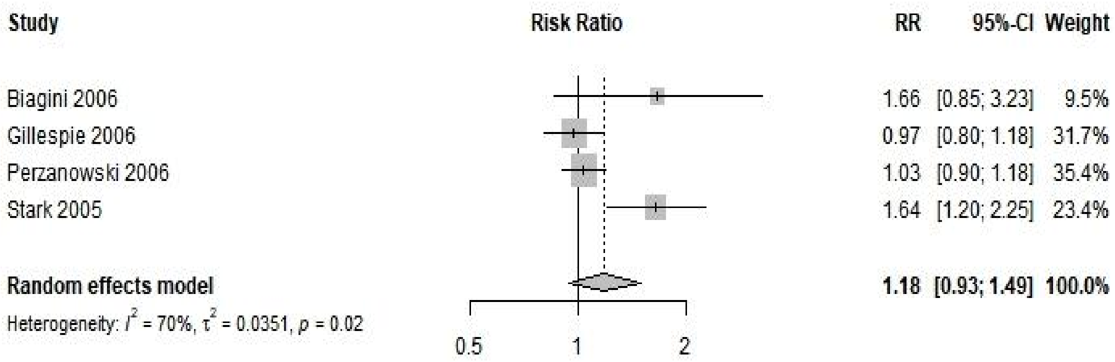

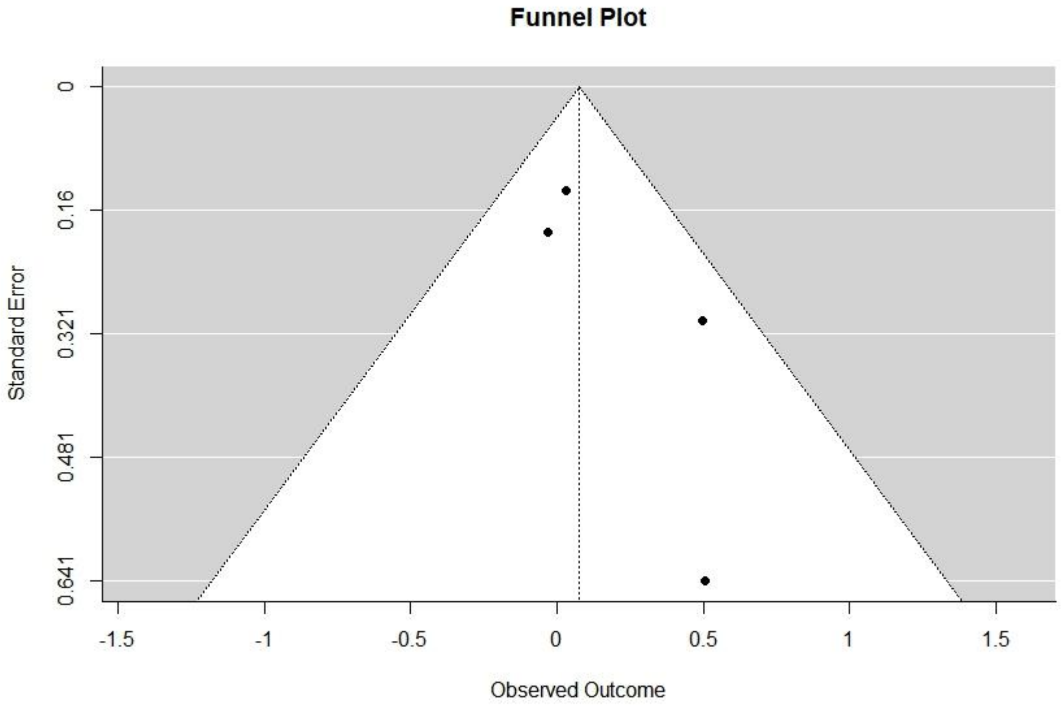

Figure S44. Random-effects meta-analysis of the association between nasal nitric oxide and AR (forest plot and funnel plot)

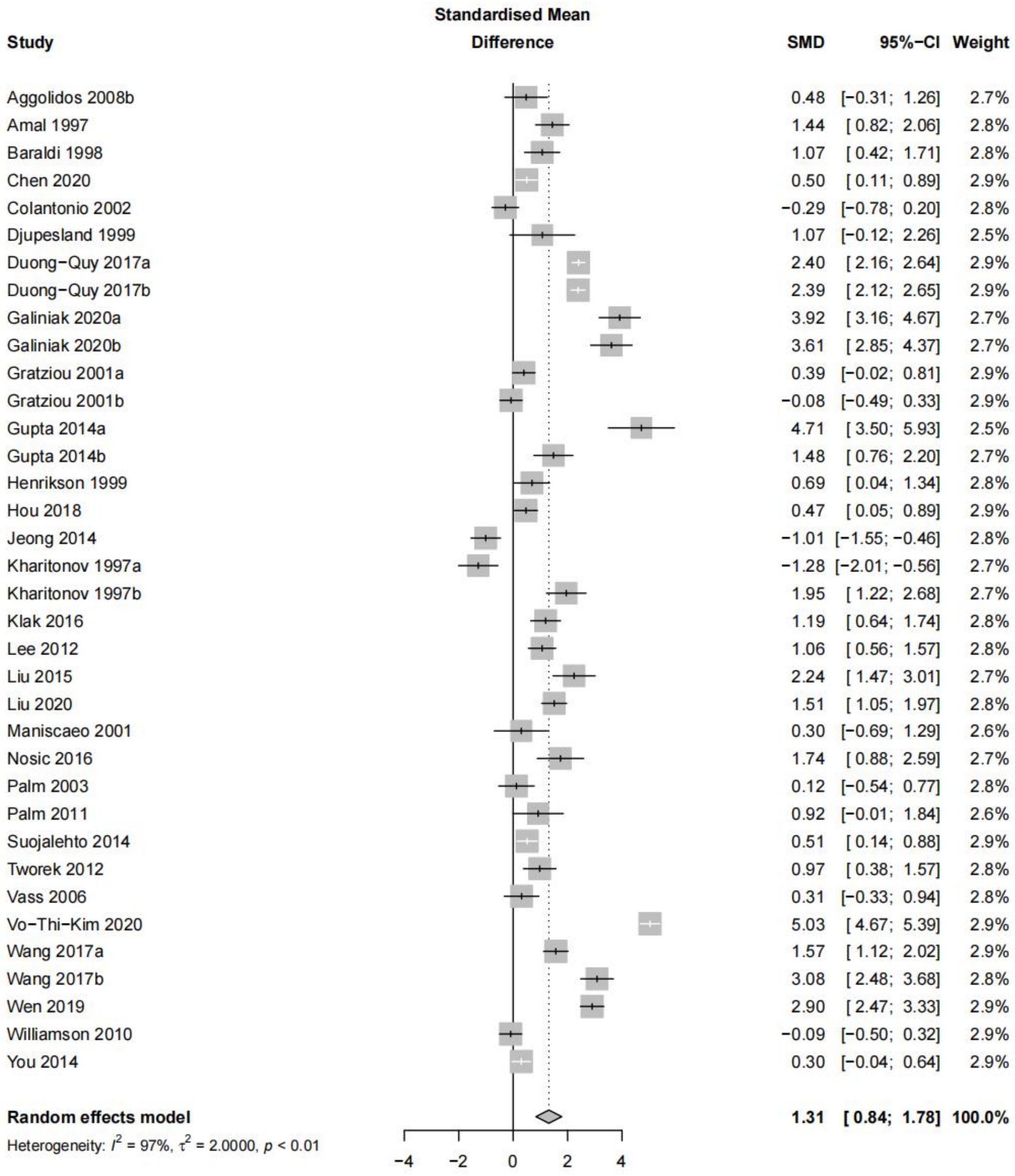

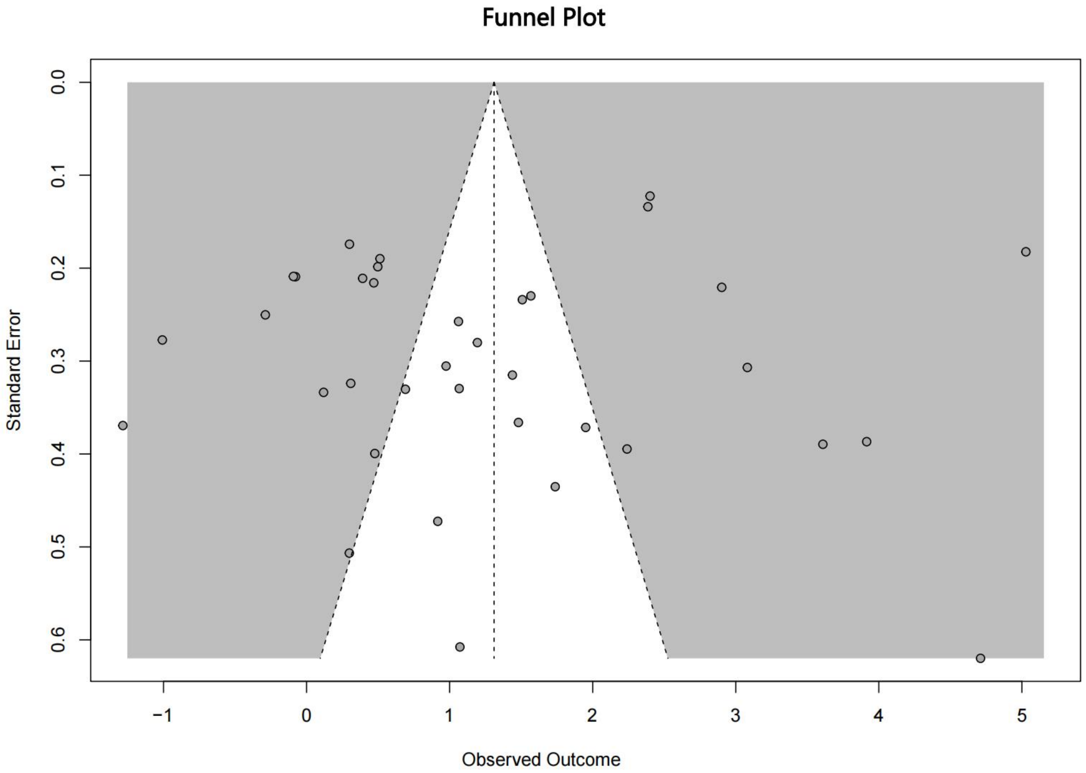
Funnel Plot
Standard Error
0.0
0.1
0.2
0.3
0.4
0.5
0.6
−1
0
1
2
3
4
5
Observed Outcome

Figure S45. Random-effects meta-analysis of the association between IL-13 rs20541 polymorphism and AR (forest plot and funnel plot)

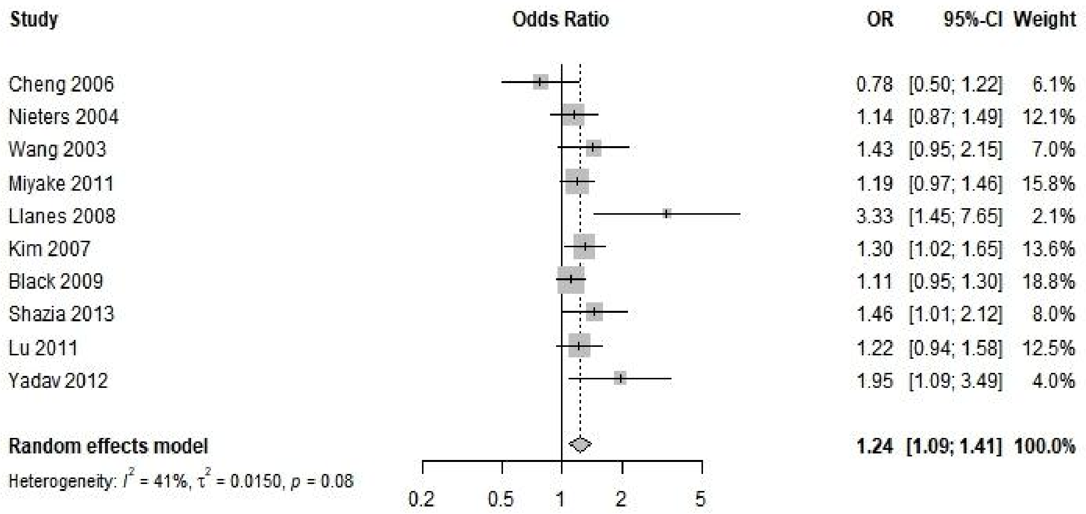

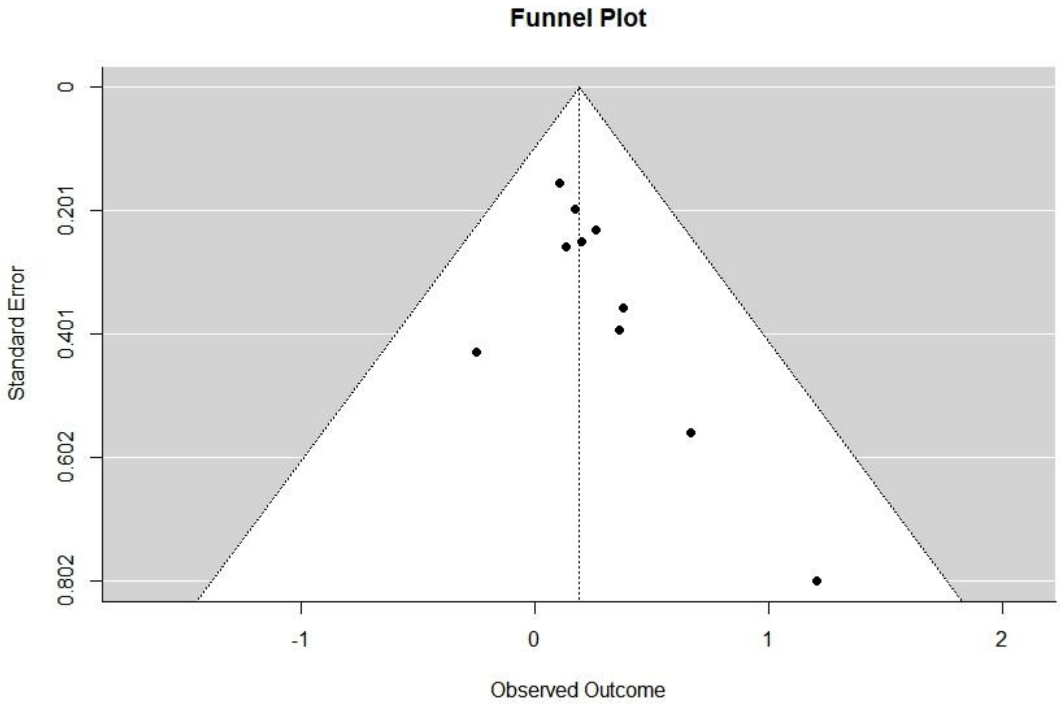

Figure S46. Random-effects meta-analysis of the association between serum IL-4 level and AR (forest plot and funnel plot)

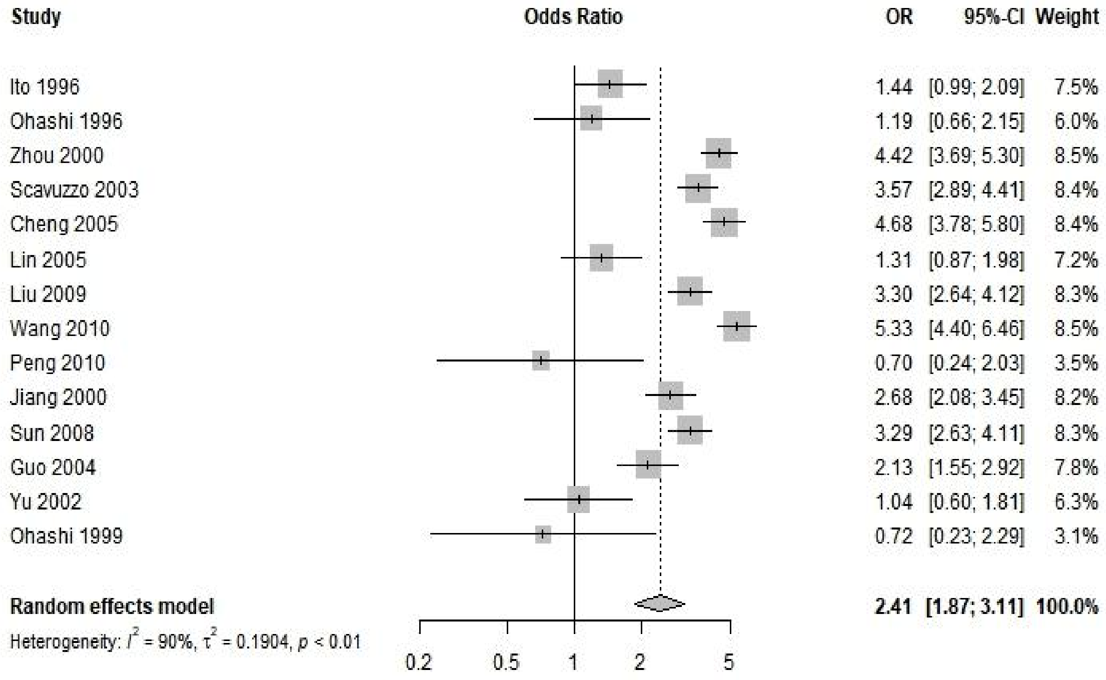

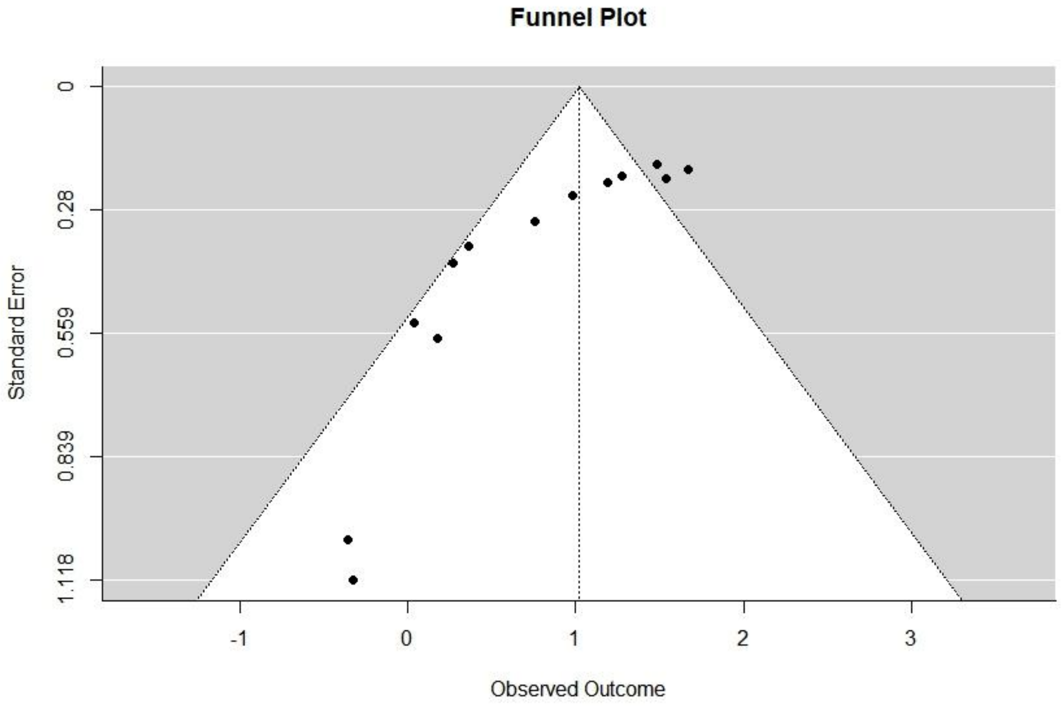

Figure S47. Random-effects meta-analysis of the association between ADAM33 S2 polymorphism and AR (forest plot and funnel plot)

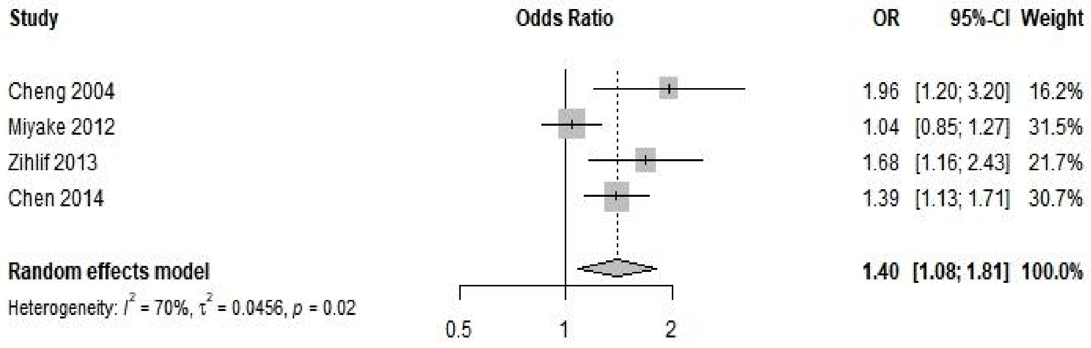

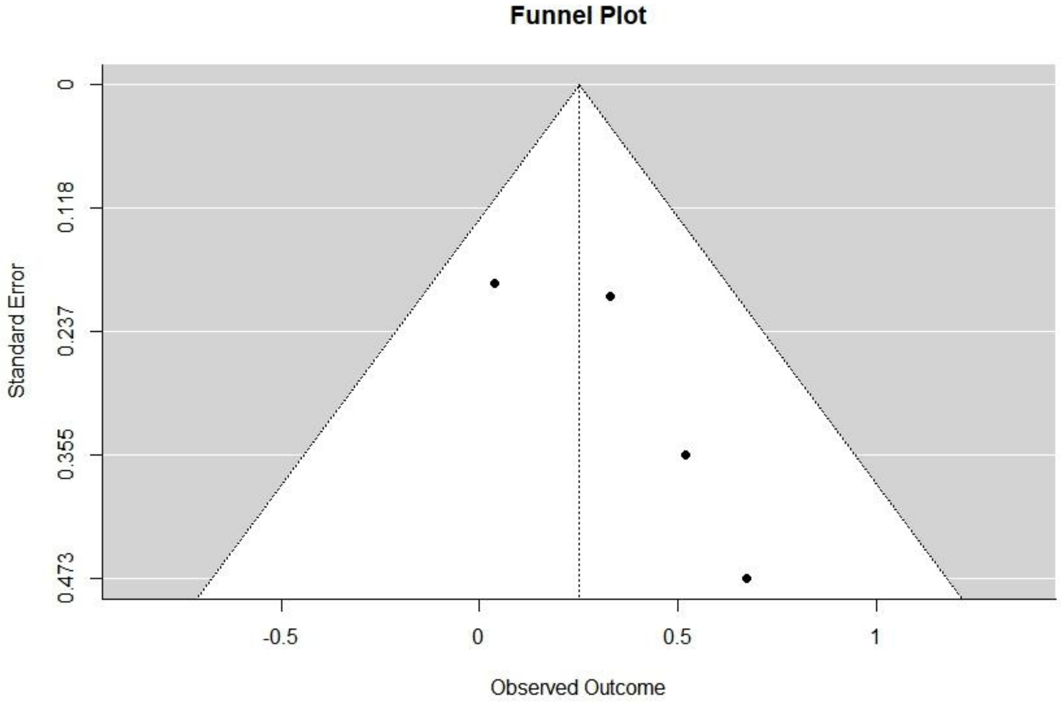

Figure S48. Random-effects meta-analysis of the association between ADAM33 V4 polymorphism and AR (forest plot and funnel plot)

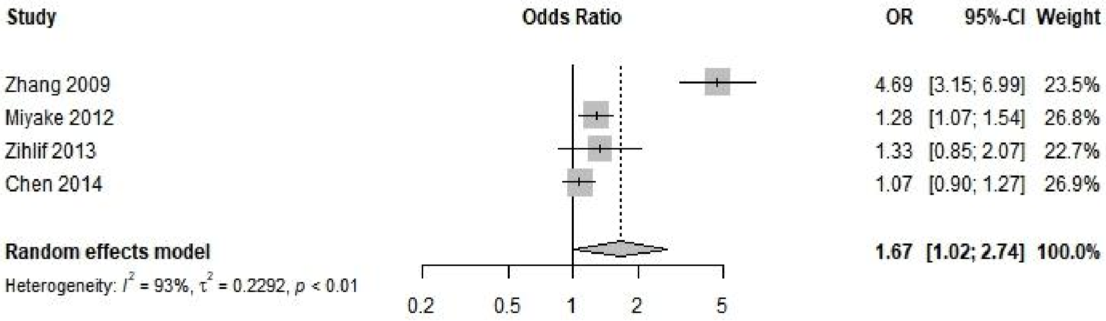

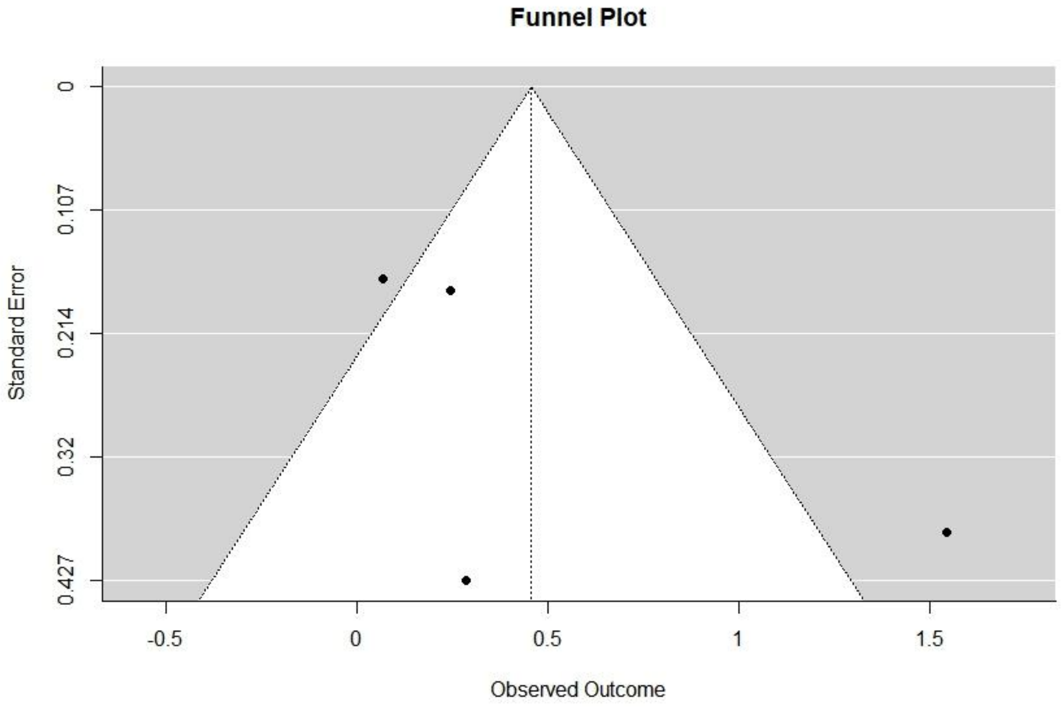

Figure S49. Random-effects meta-analysis of the association between ADAM33 Q−1 polymorphism and AR (forest plot and funnel plot)

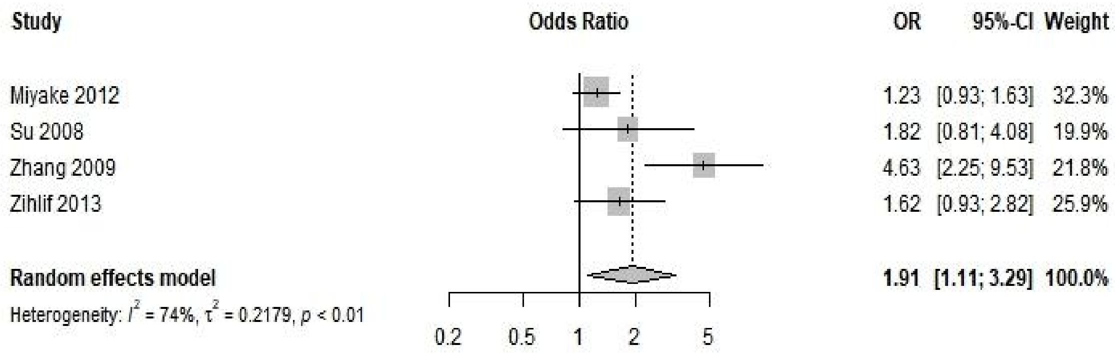

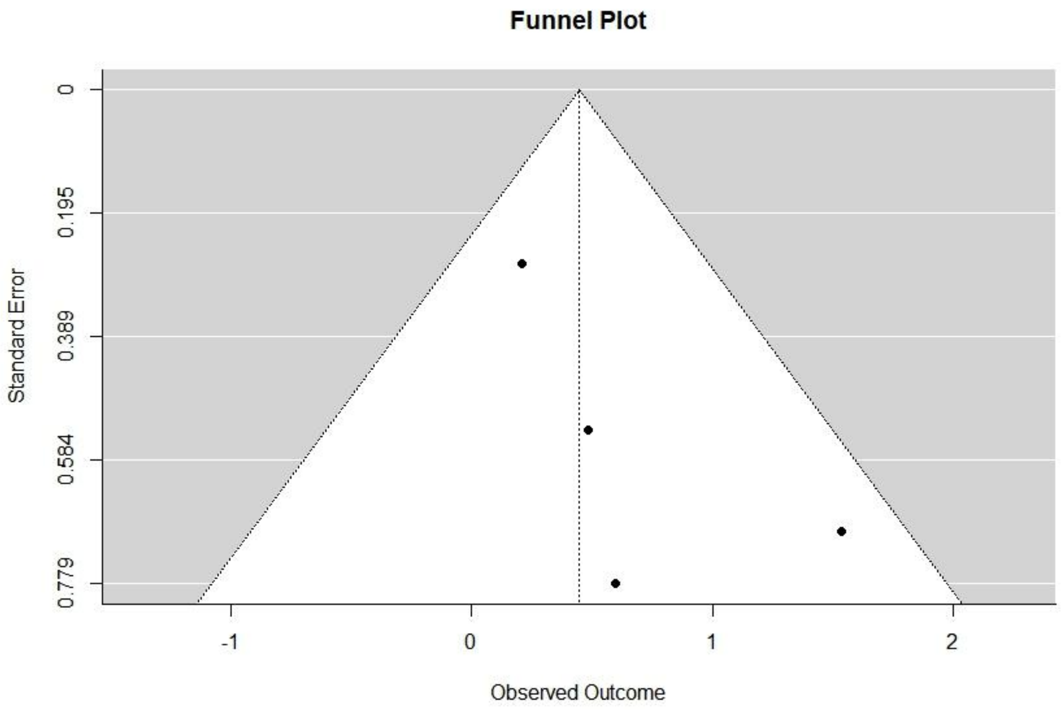

Figure S50. Random-effects meta-analysis of the association between ACEI/D polymorphism and AR (forest plot and funnel plot)

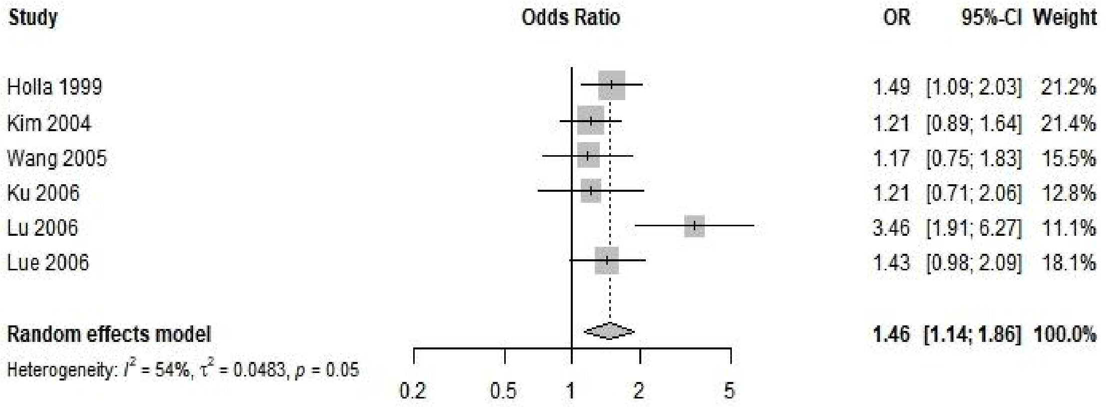

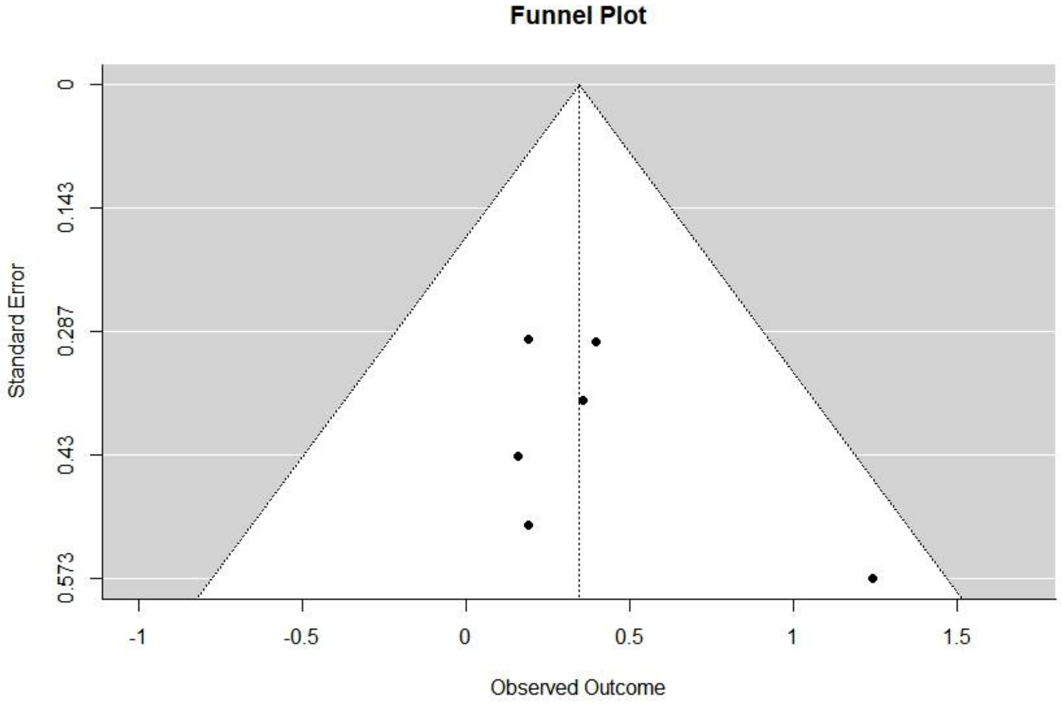

Figure S51. Random-effects meta-analysis of the association between TNF α rs1800629 polymorphism and AR (forest plot and funnel plot)

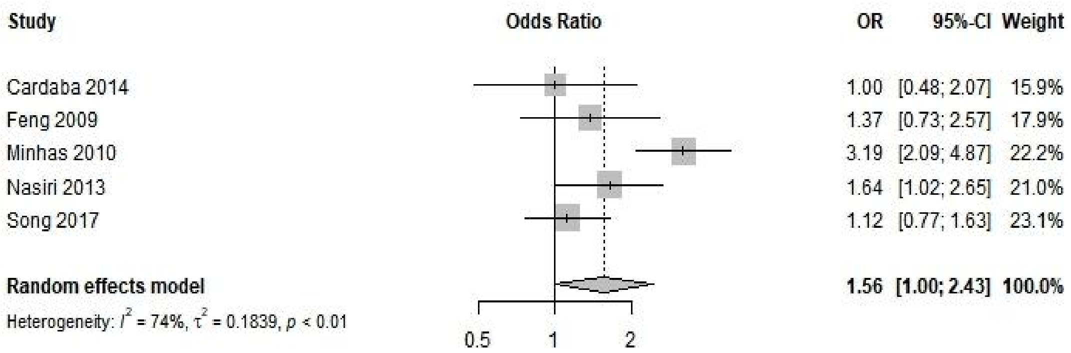

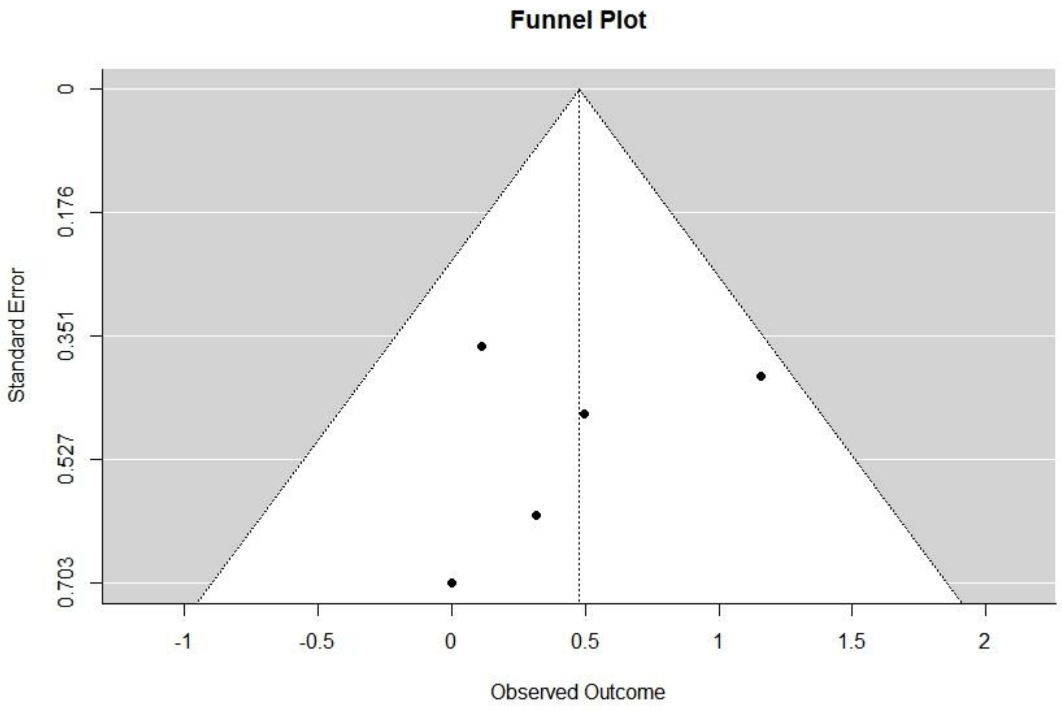

Figure S52. Random-effects meta-analysis of the association between CTLA-4 rs11571302 polymorphism and AR (forest plot and funnel plot)

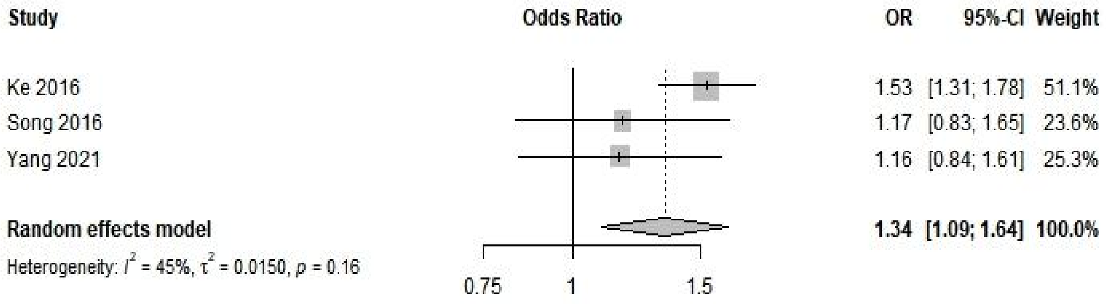

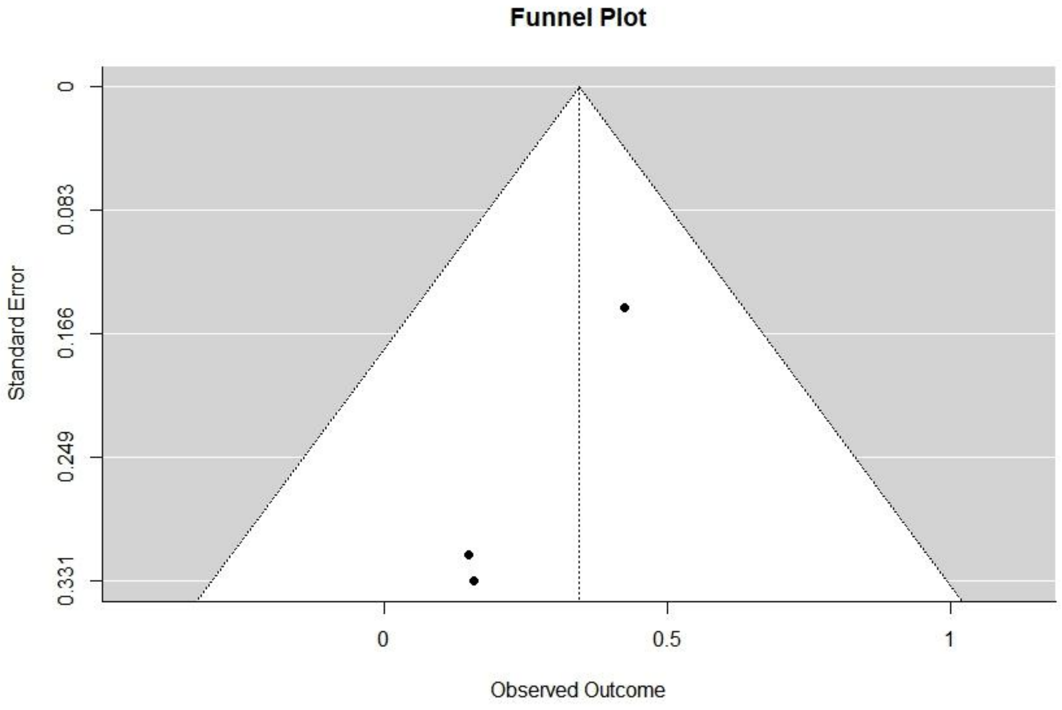

Figure S53. Random-effects meta-analysis of the association between IL-4R rs1801275 polymorphism and AR (forest plot and funnel plot)

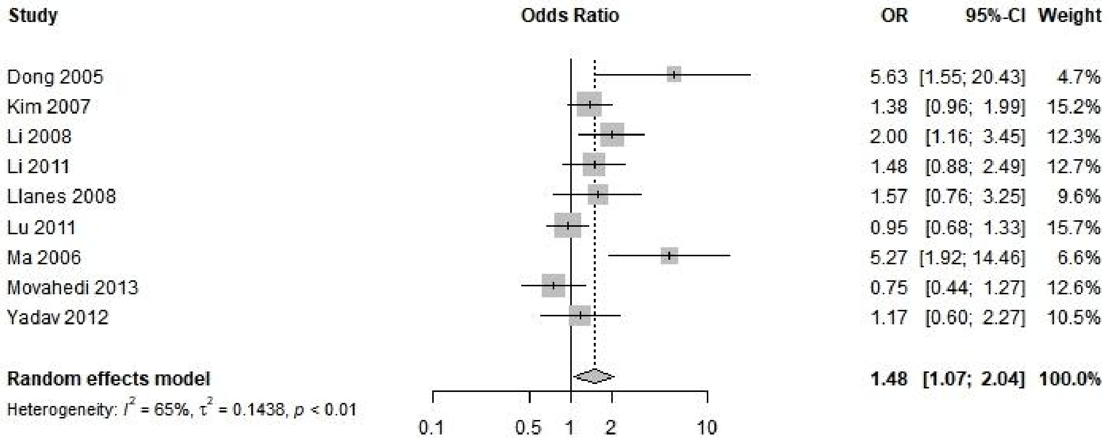

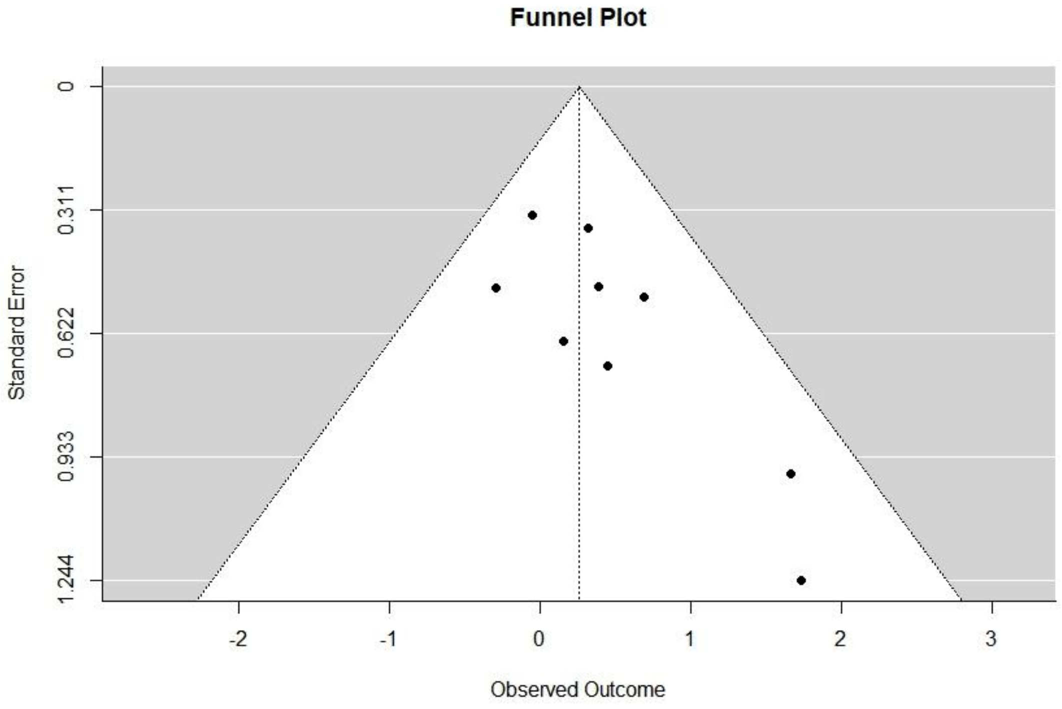

Figure S54. Random-effects meta-analysis of the association between ADAM33 T1 polymorphism and AR (forest plot and funnel plot)

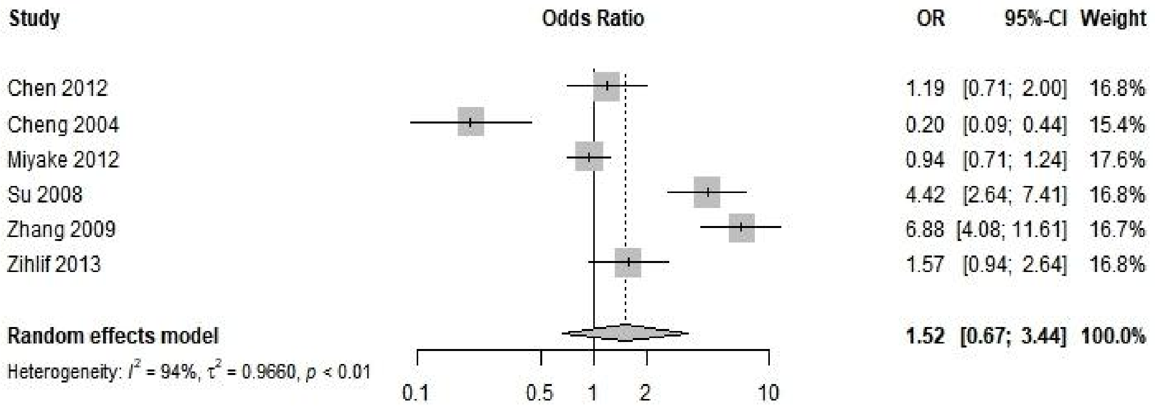

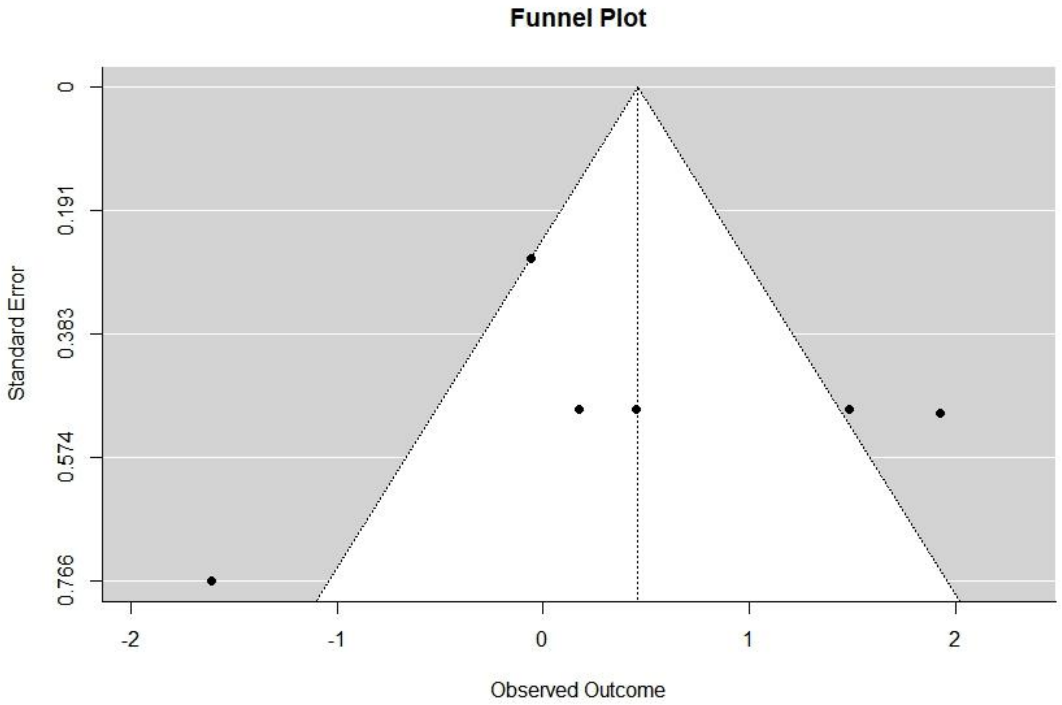

Figure S55. Random-effects meta-analysis of the association between ADAM33 T2 polymorphism and AR (forest plot and funnel plot)

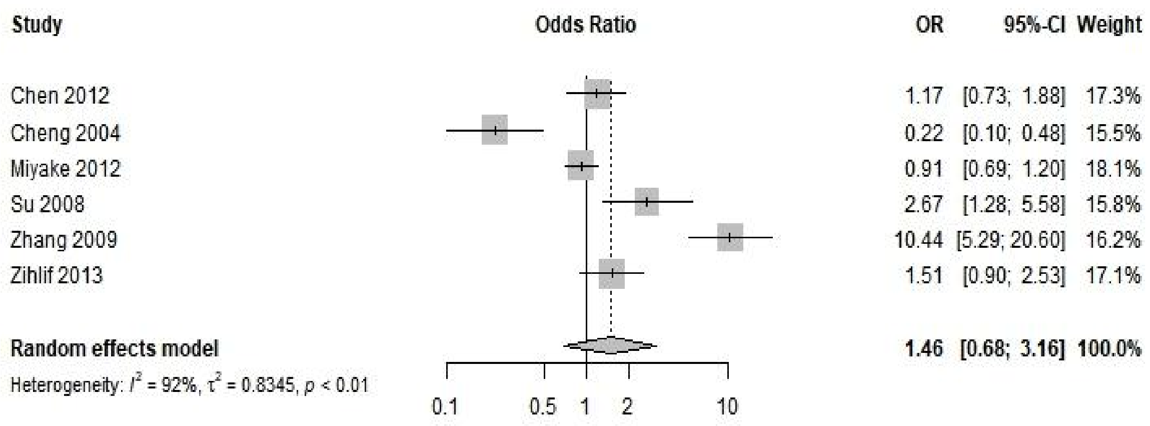

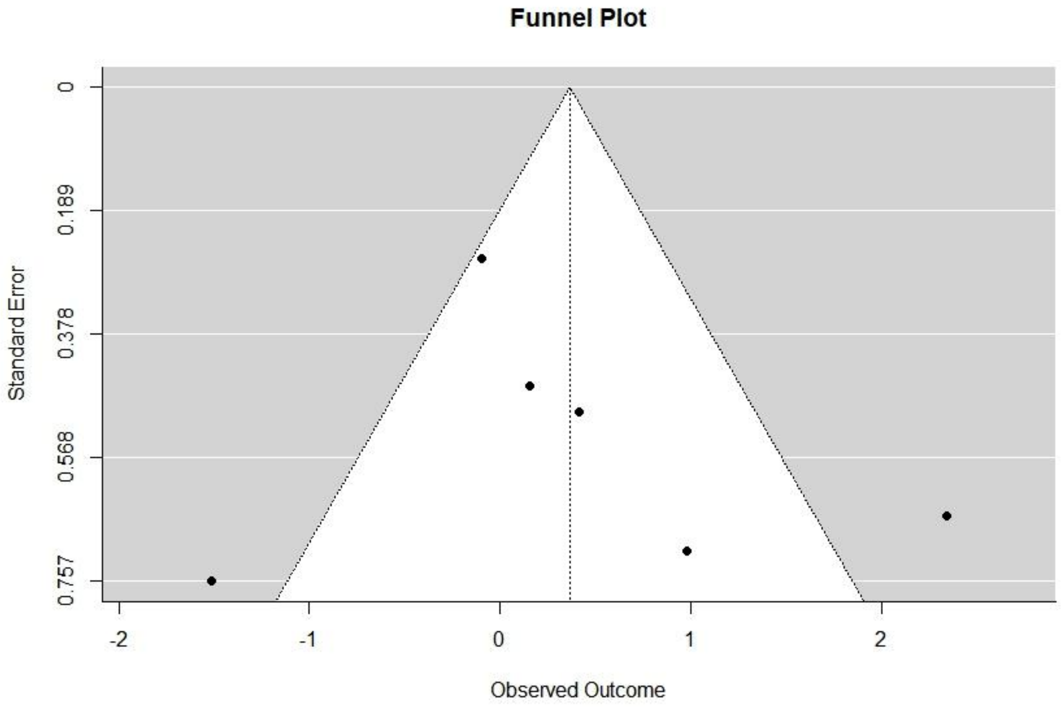

Figure S56. Random-effects meta-analysis of the association between IL-4 rs2243250 polymorphism and AR (forest plot and funnel plot)

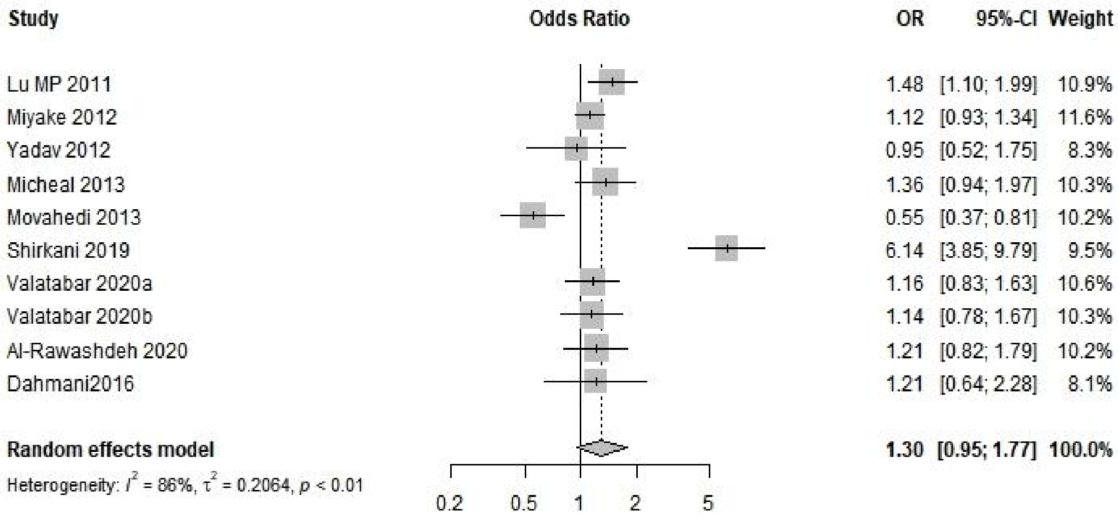

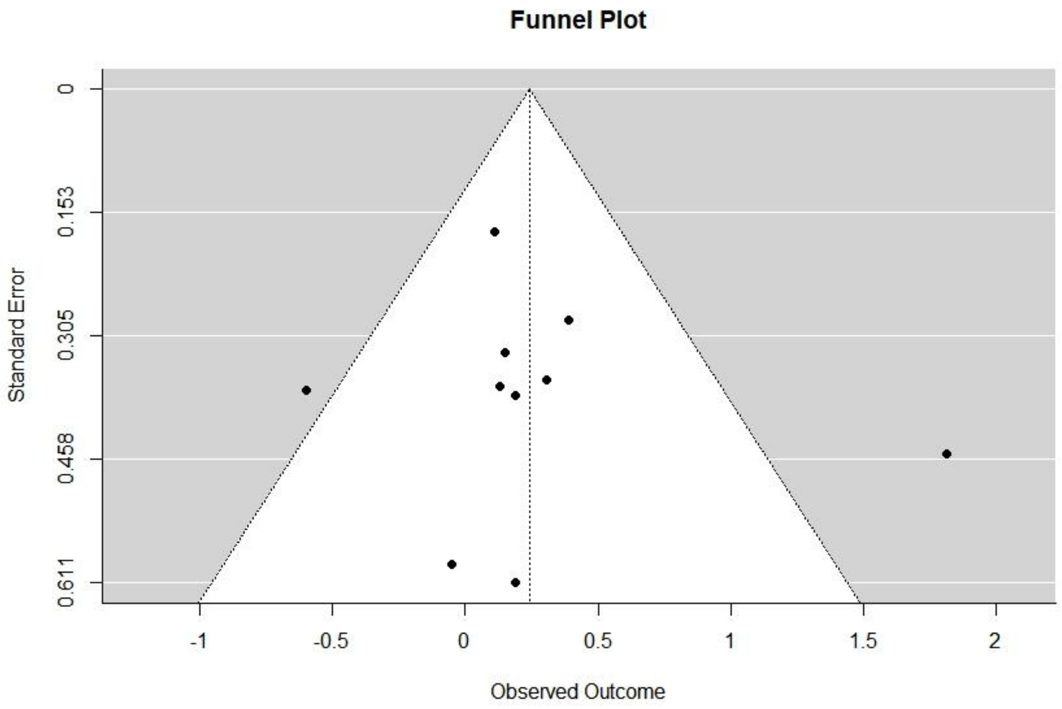

Figure S57. Random-effects meta-analysis of the association between IL-4RA Ile50Val polymorphism and AR (forest plot and funnel plot)

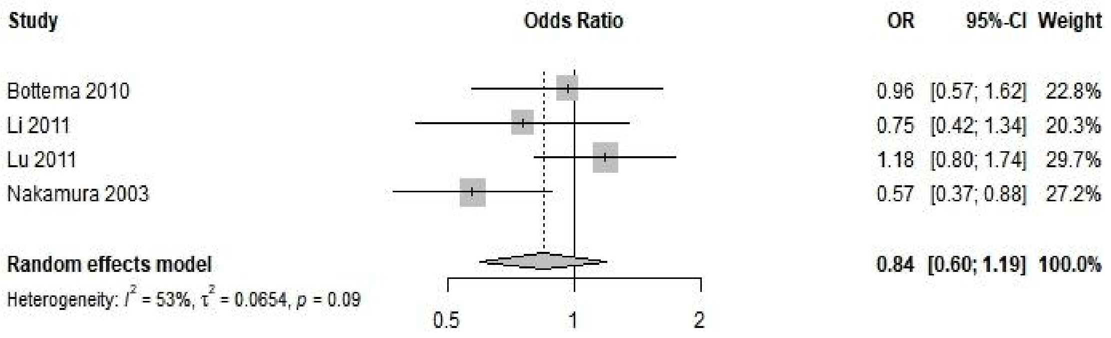

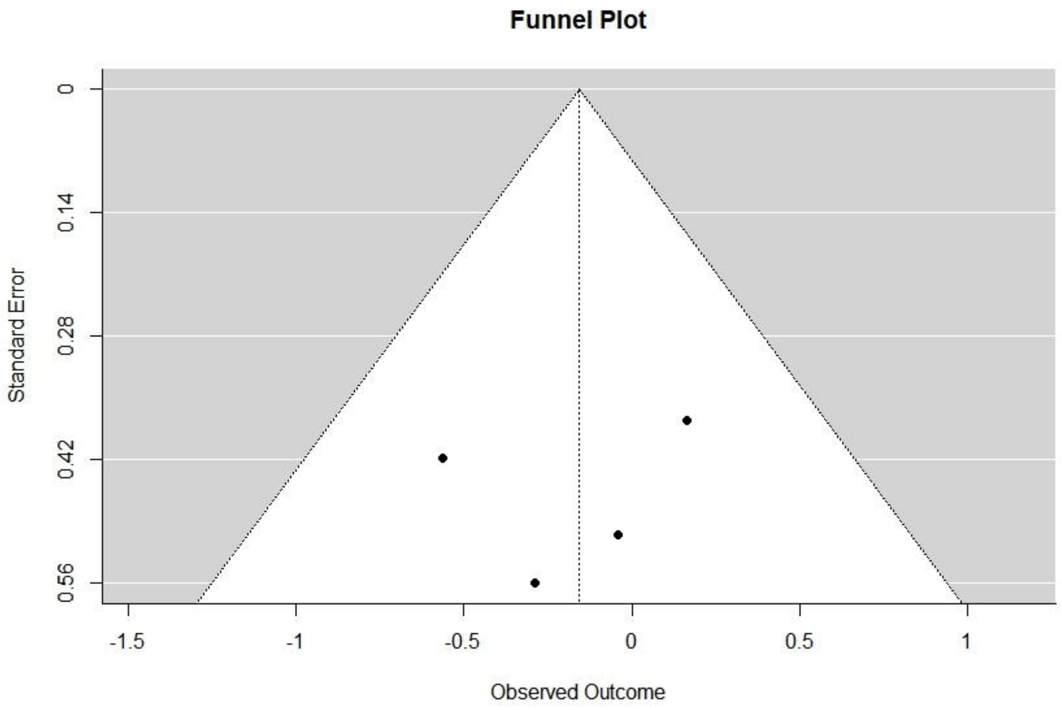

Figure S58. Random-effects meta-analysis of the association between IL-4RA Ser478Pro polymorphism and AR (forest plot and funnel plot)

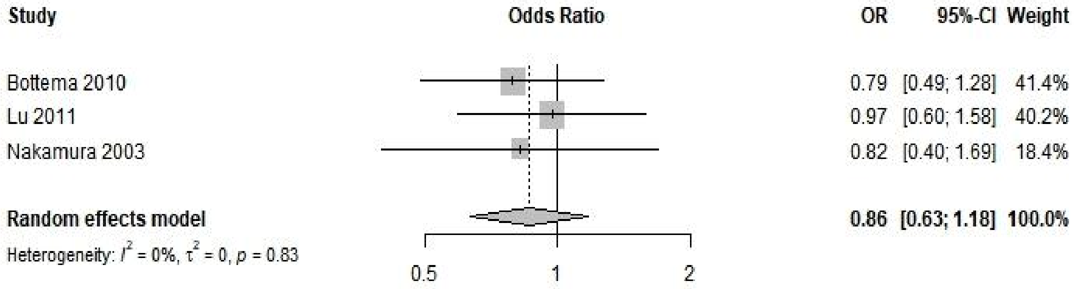

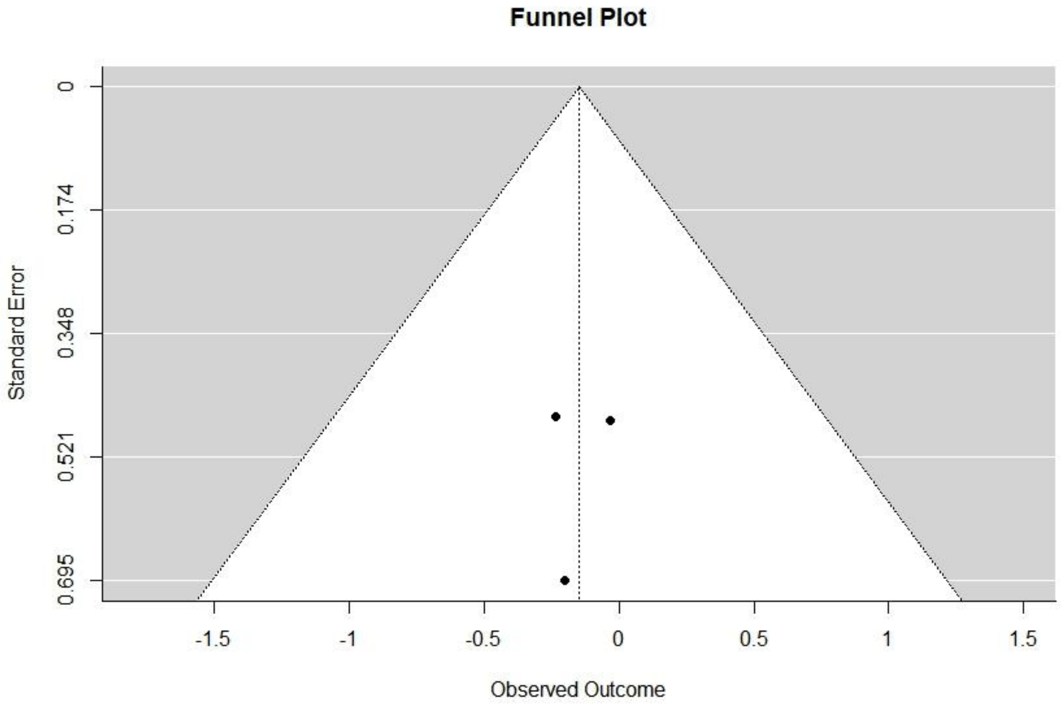

Figure S59. Random-effects meta-analysis of the association between IL-4RA Gln551Arg polymorphism and AR (forest plot and funnel plot)

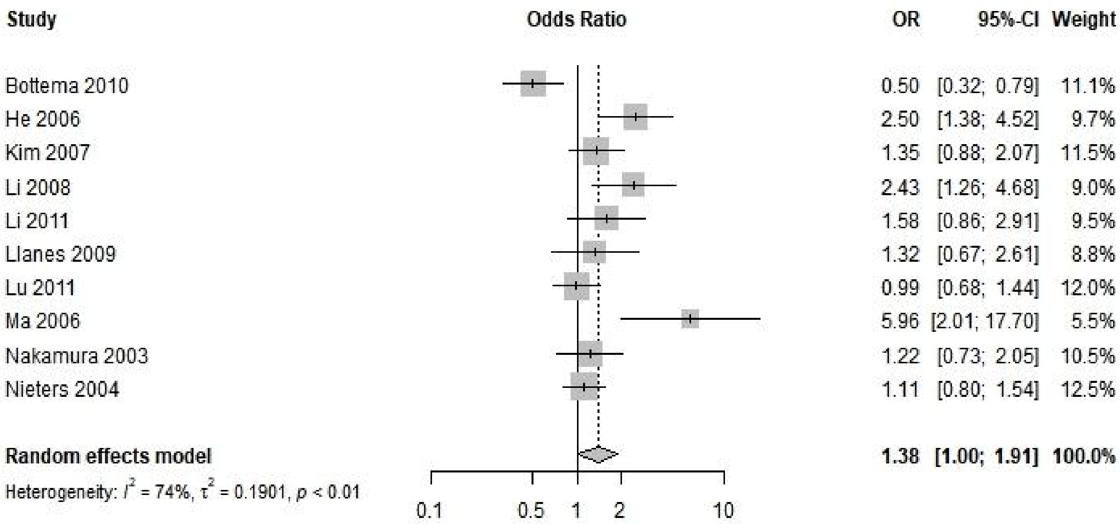

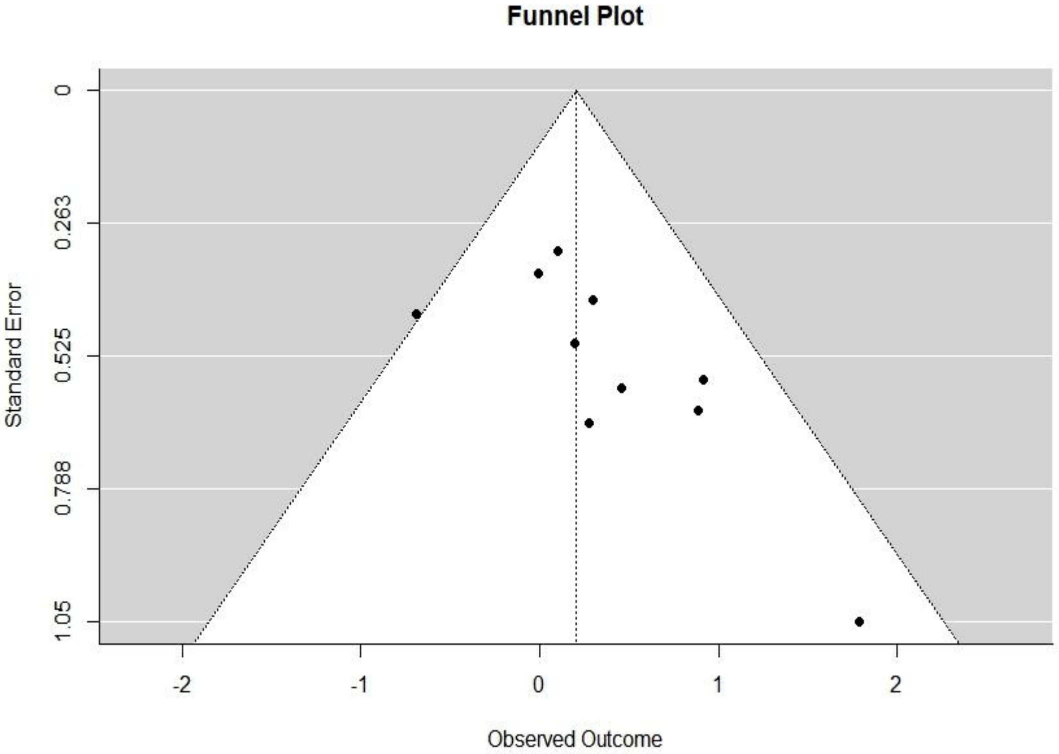

Figure S60. Random-effects meta-analysis of the association between IL-13 rs1800925 polymorphism and AR (forest plot and funnel plot)

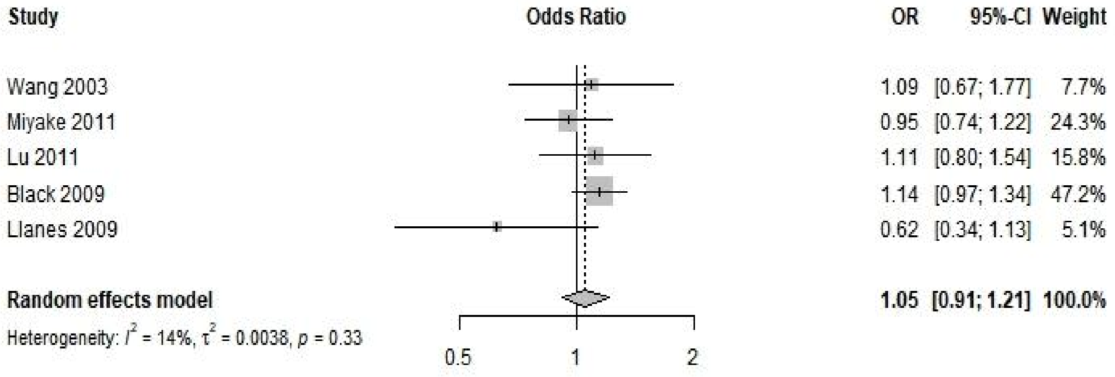

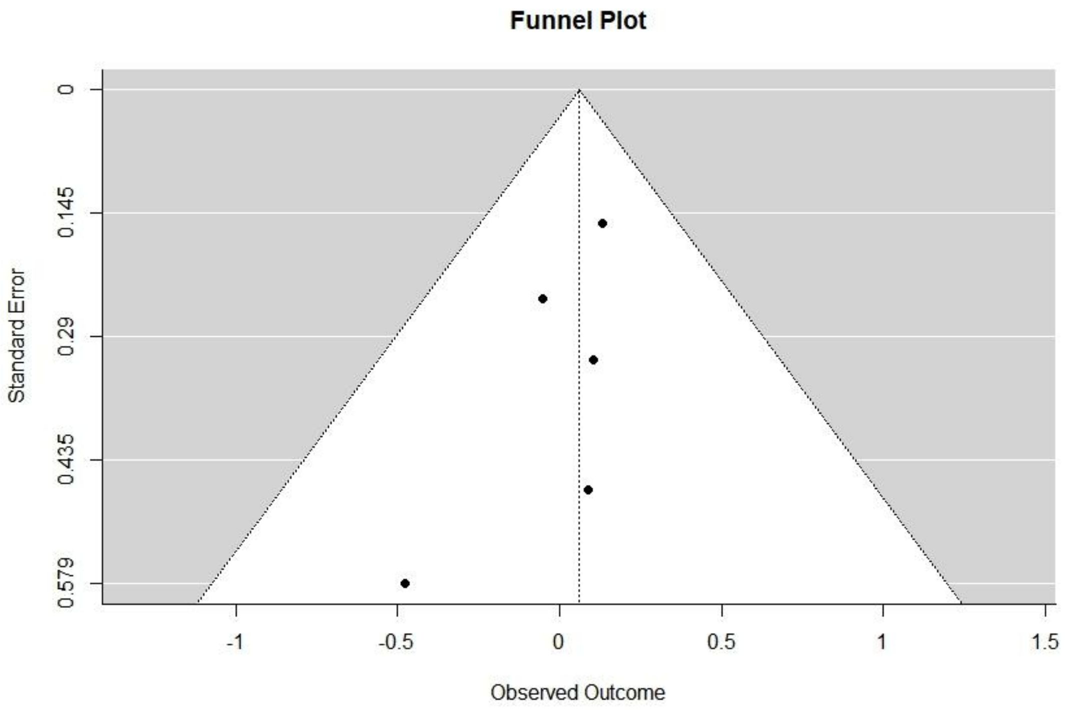

Figure S61. Random-effects meta-analysis of the association between CD14 rs2569190 polymorphism and AR (forest plot and funnel plot)

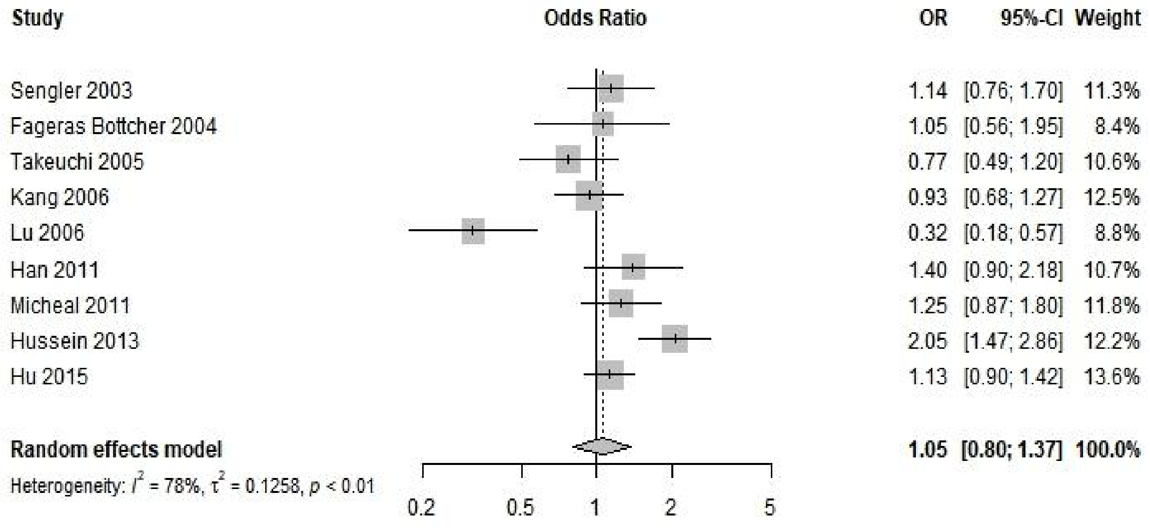

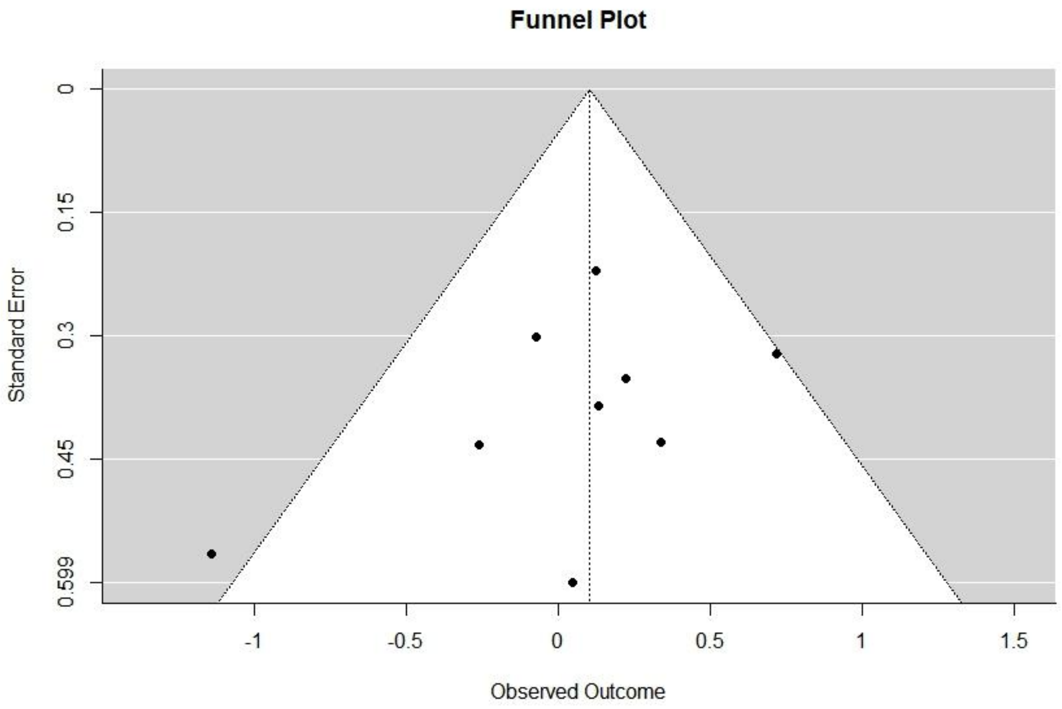

Figure S62. Random-effects meta-analysis of the association between TGF β1 rs1800469 polymorphism and AR (forest plot and funnel plot)

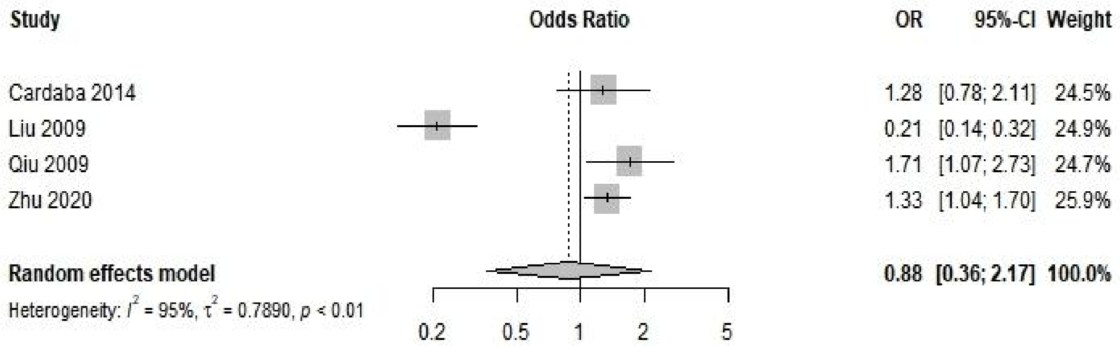

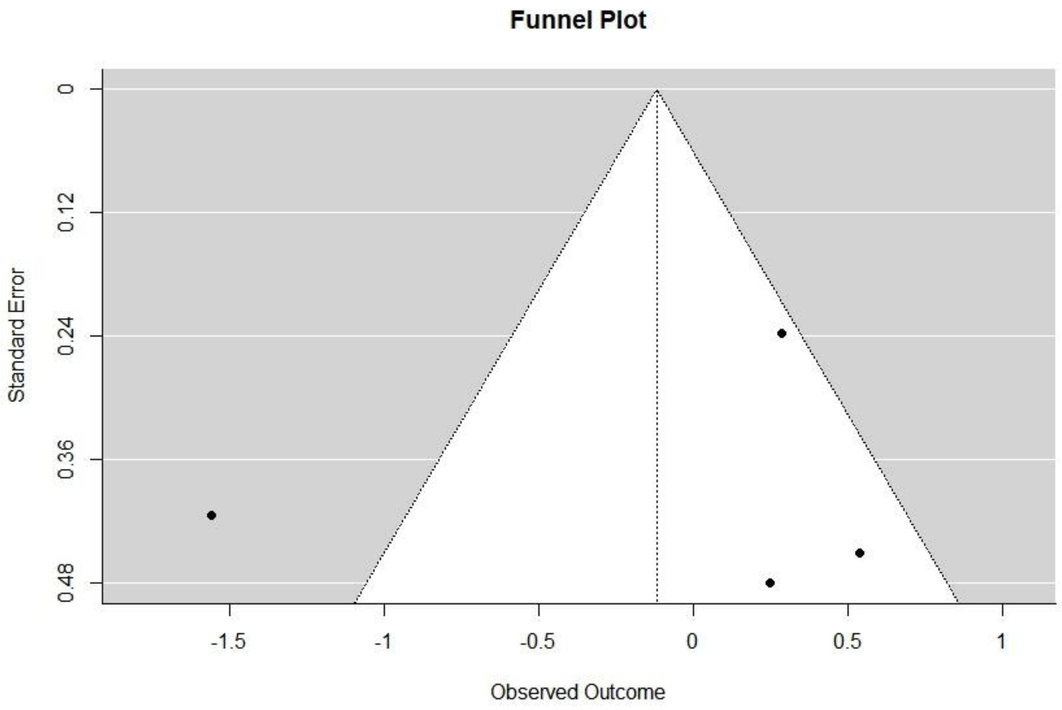

Figure S63. Random-effects meta-analysis of the association between CTLA-4 rs3087243 polymorphism and AR (forest plot and funnel plot)

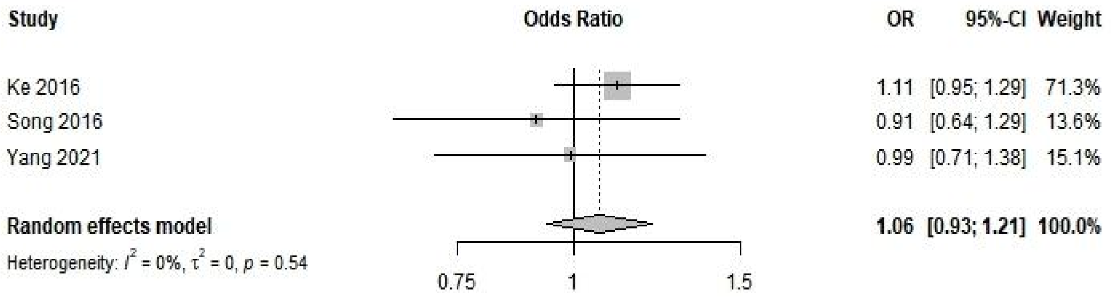

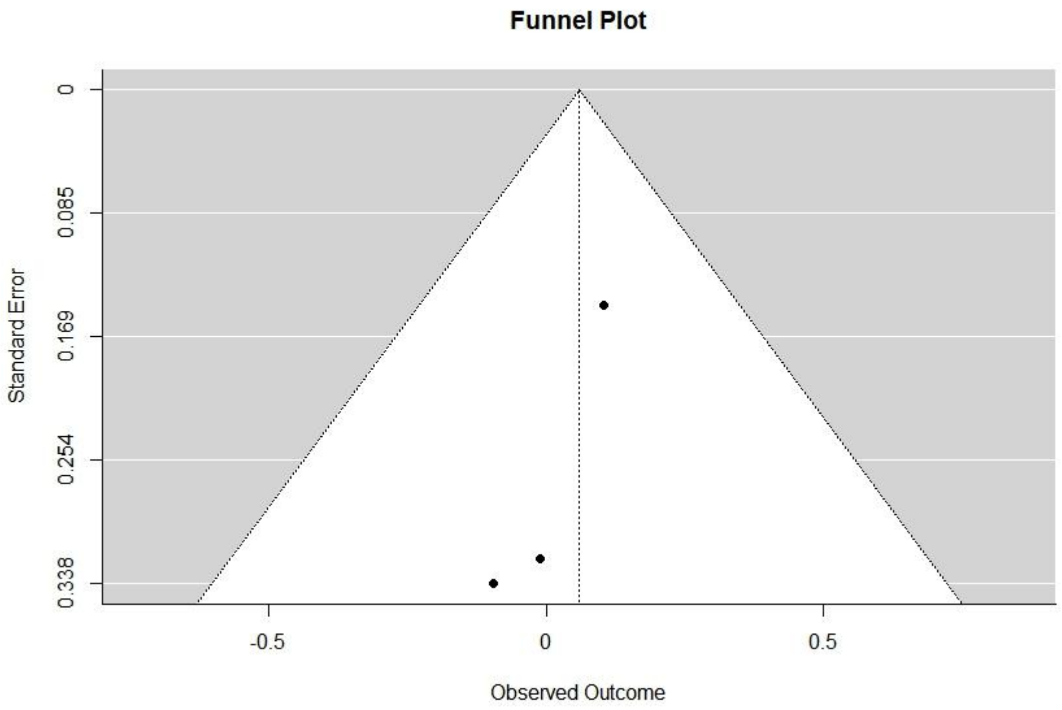

Figure S64. Random-effects meta-analysis of the association between CTLA-4 rs231725 polymorphism and AR (forest plot and funnel plot)

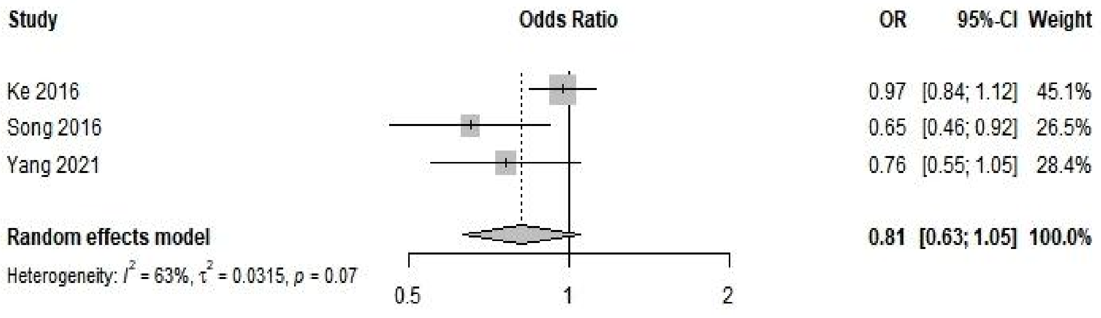

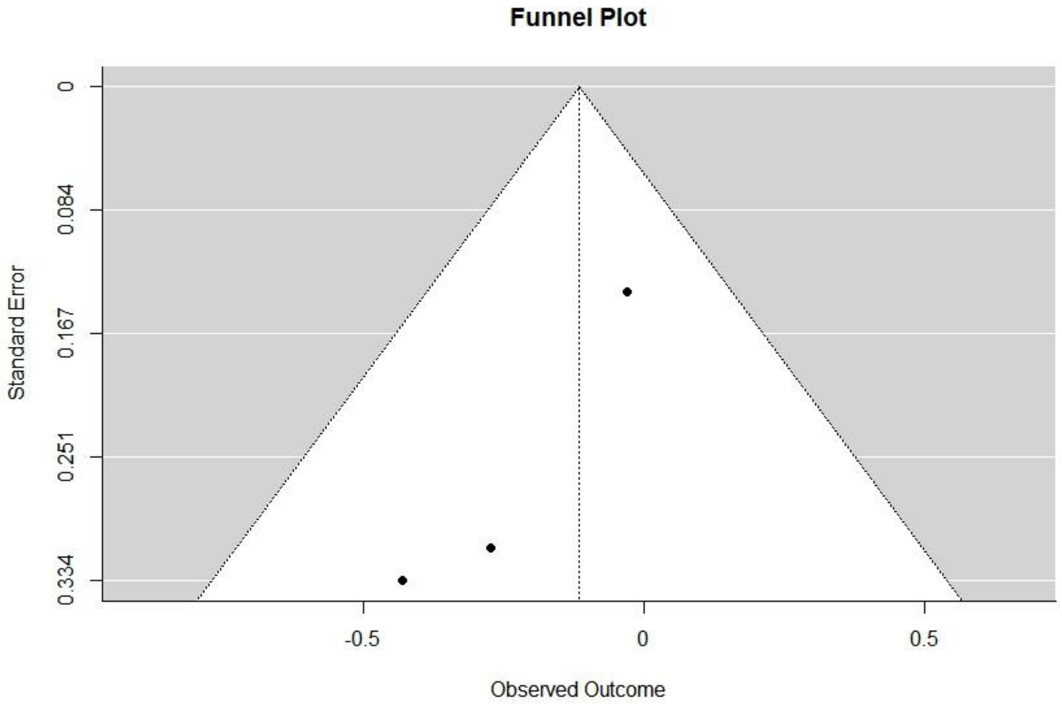

Figure S65. Random-effects meta-analysis of the association between CTLA-4 rs11571315 polymorphism and AR (forest plot and funnel plot)

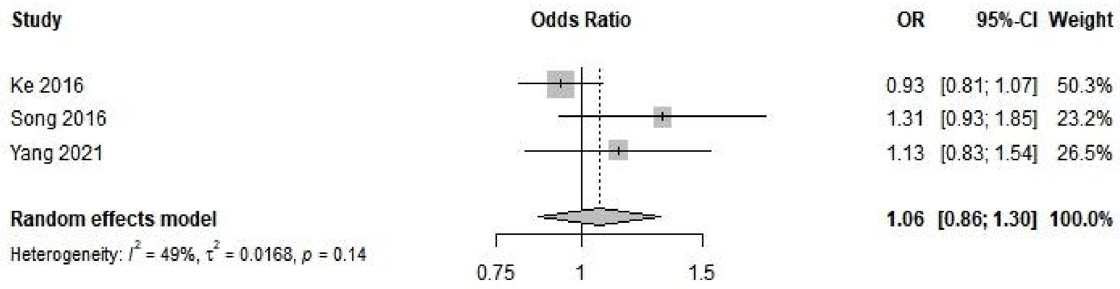

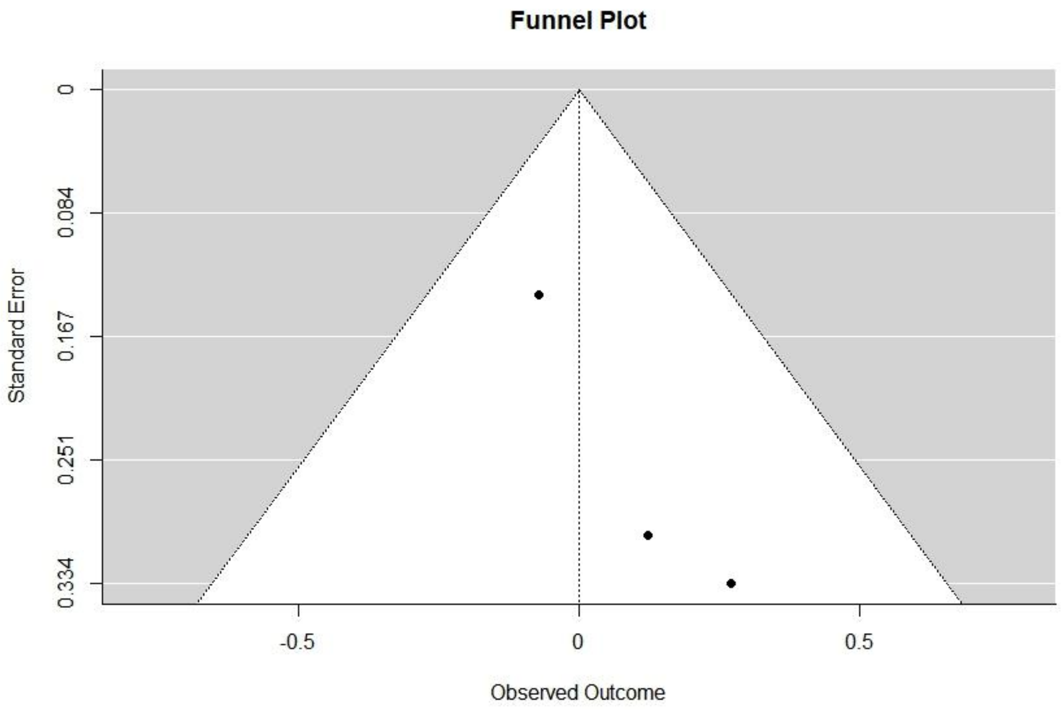

Figure S66. Random-effects meta-analysis of the association between IL-4 rs2227284 polymorphism and AR (forest plot and funnel plot)

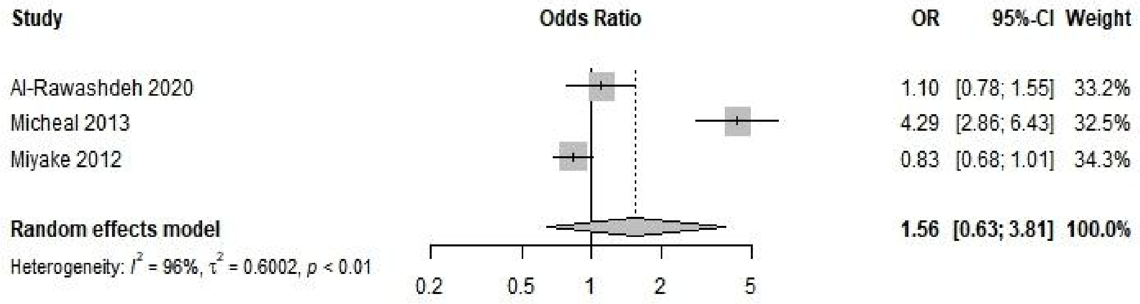

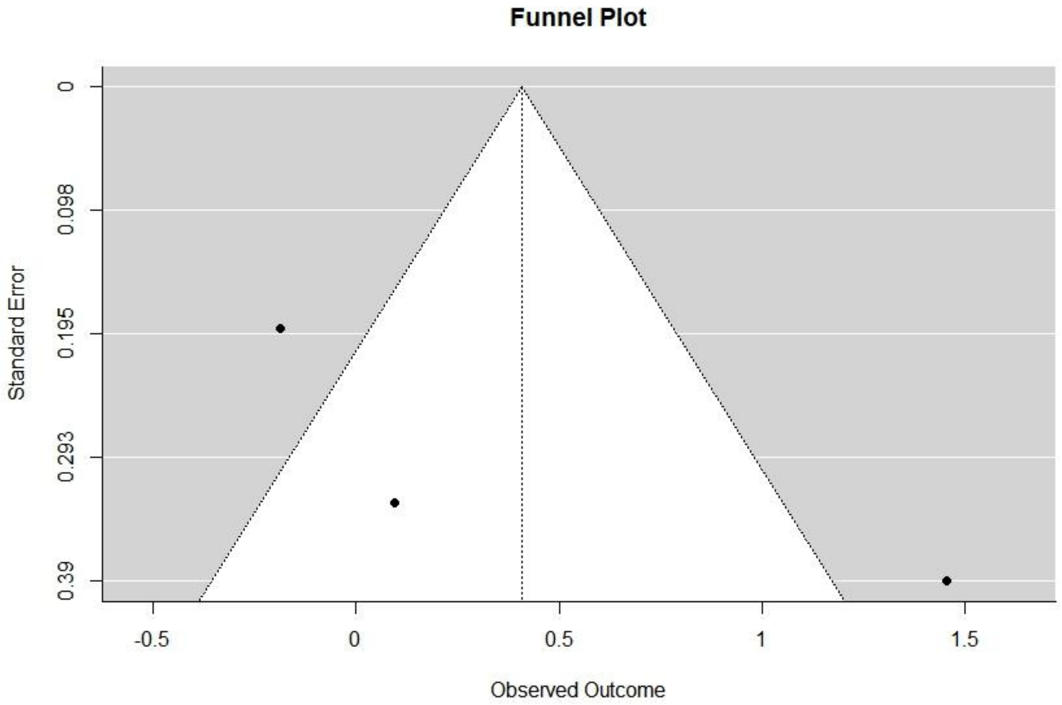

Figure S67. Random-effects meta-analysis of the association between IL-4 rs2070874 polymorphism and AR (forest plot and funnel plot)

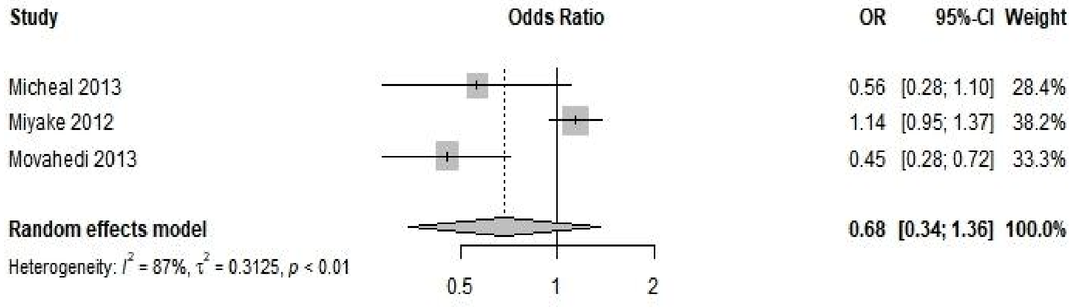

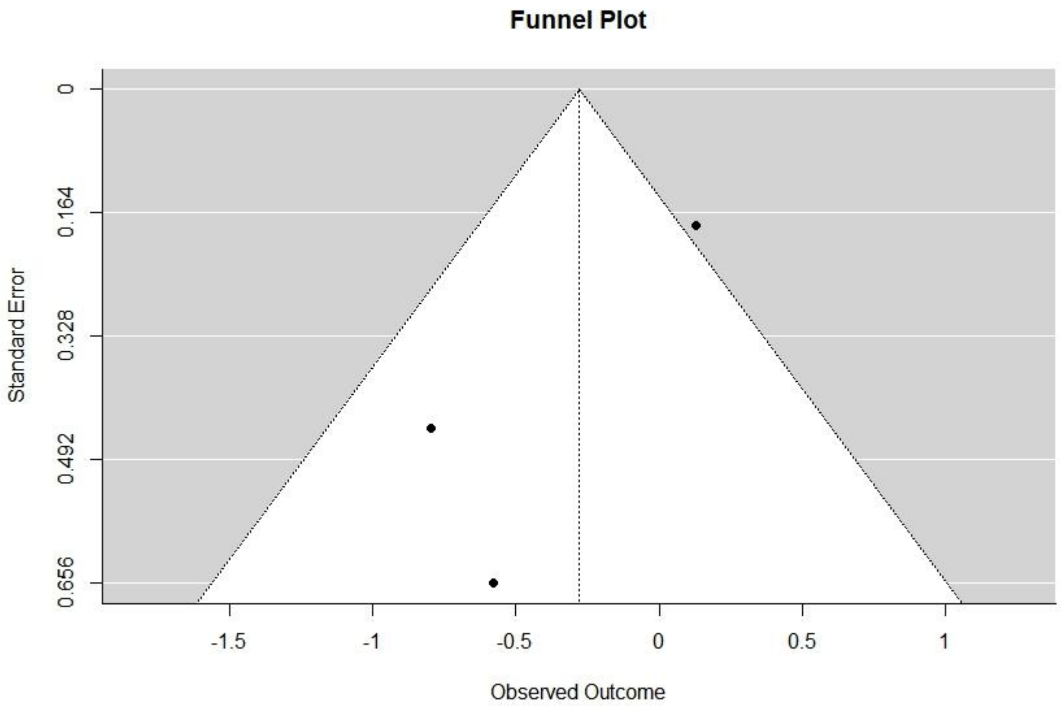

Figure S68. Random-effects meta-analysis of the association between FOX P3 rs3761548 polymorphism and AR (forest plot and funnel plot)

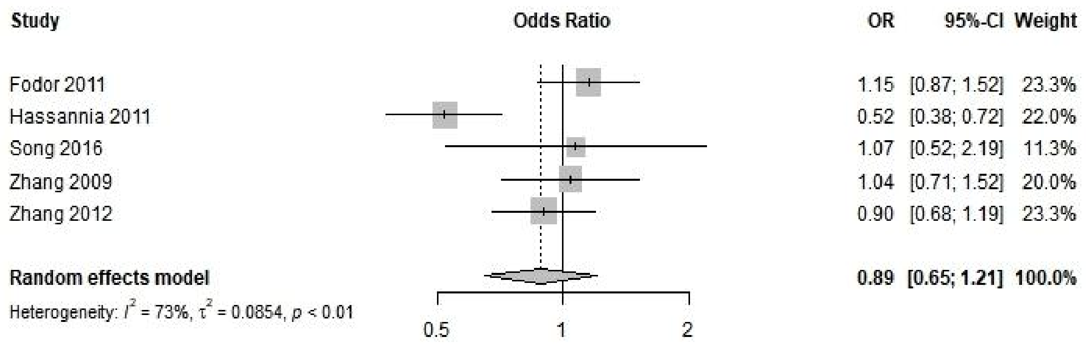

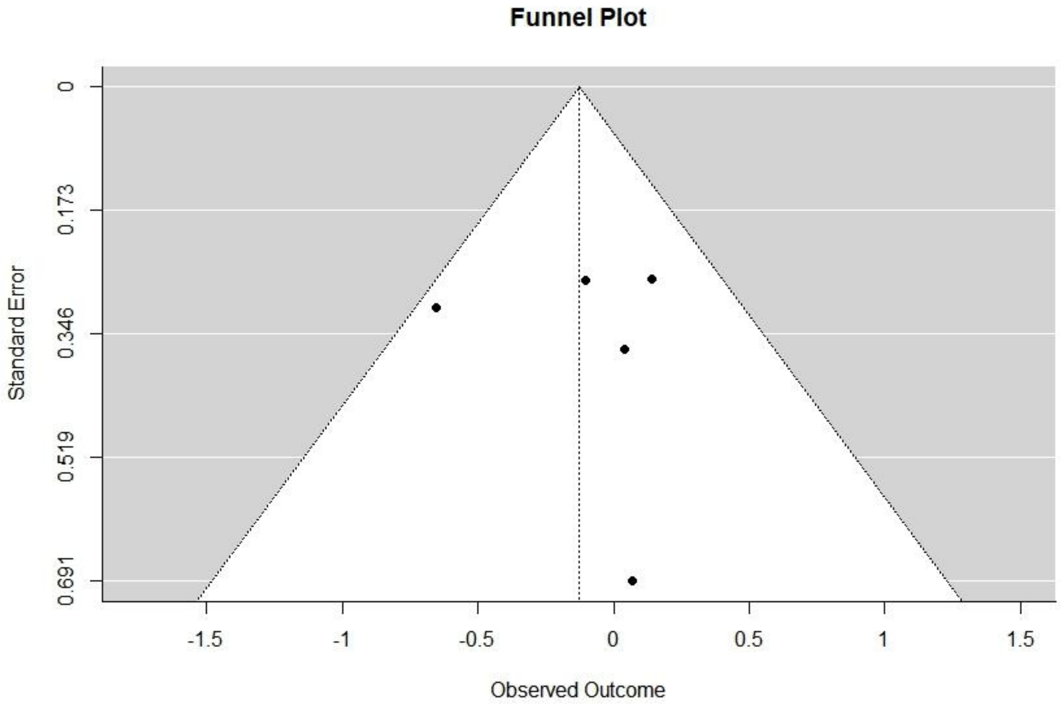

Figure S69 Random-effects meta-analysis of the association between FOX P3 rs2232365 polymorphism and AR (forest plot and funnel plot)

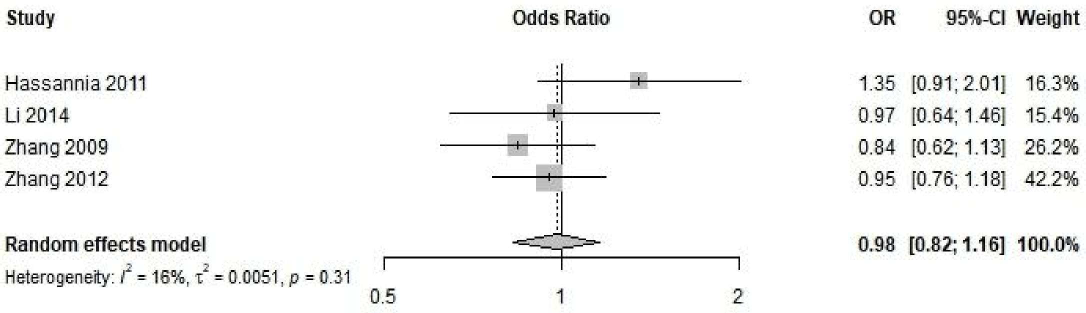

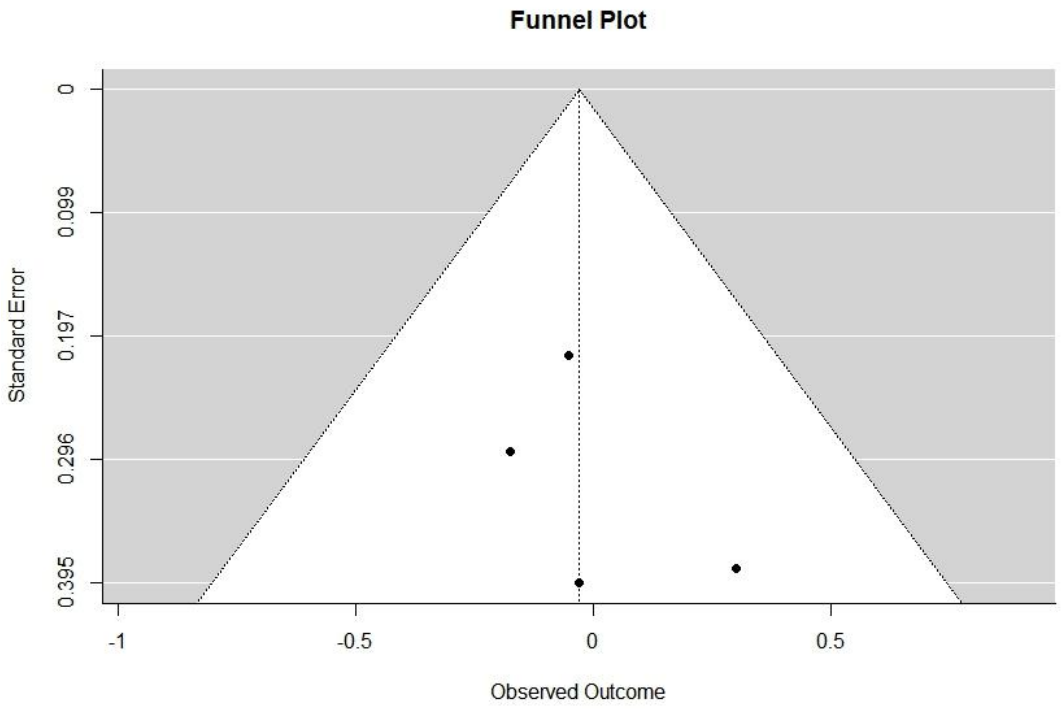

Figure S70 Random-effects meta-analysis of the association between IL-18 rs1946518 polymorphism and AR (forest plot and funnel plot)

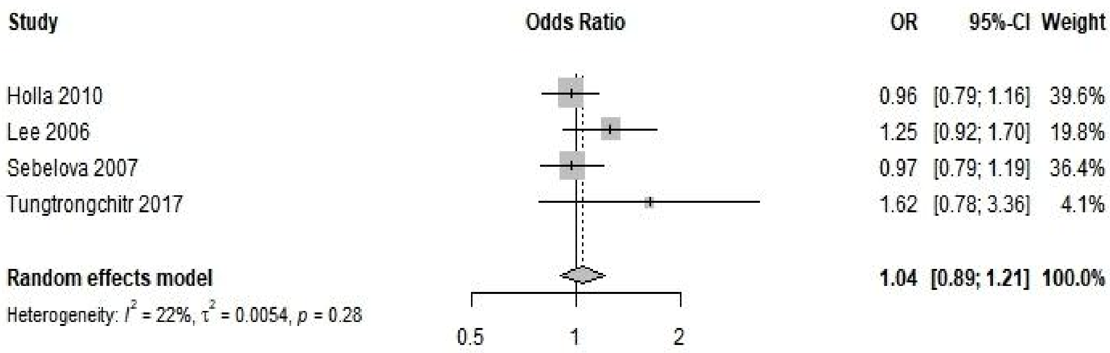

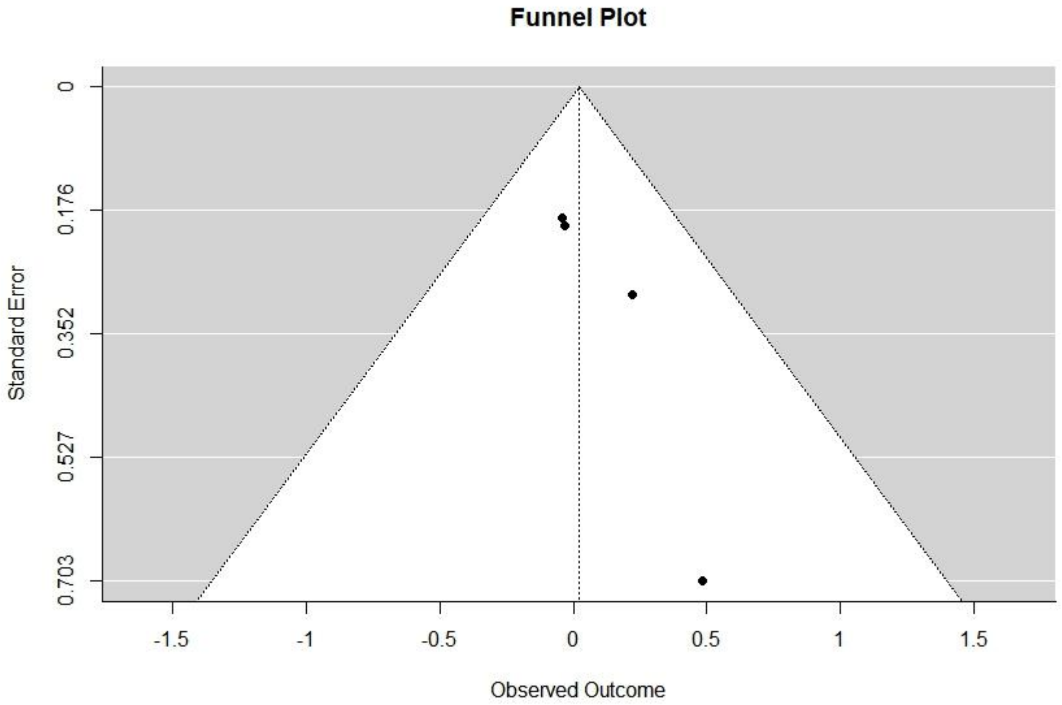

Figure S71 Random-effects meta-analysis of the association between IL-18 rs187238 polymorphism and AR (forest plot and funnel plot)

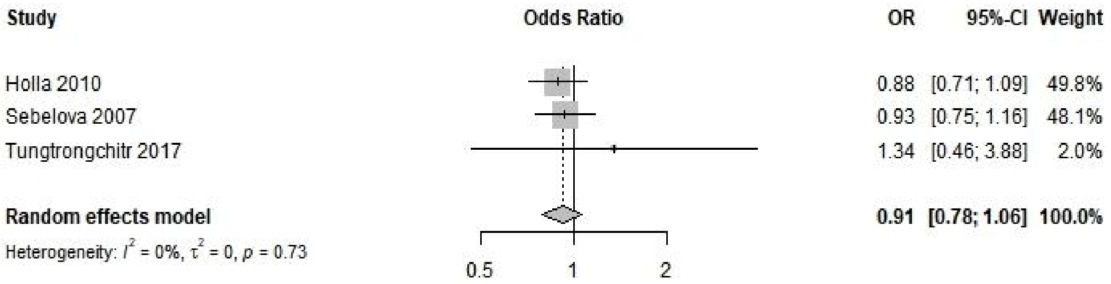

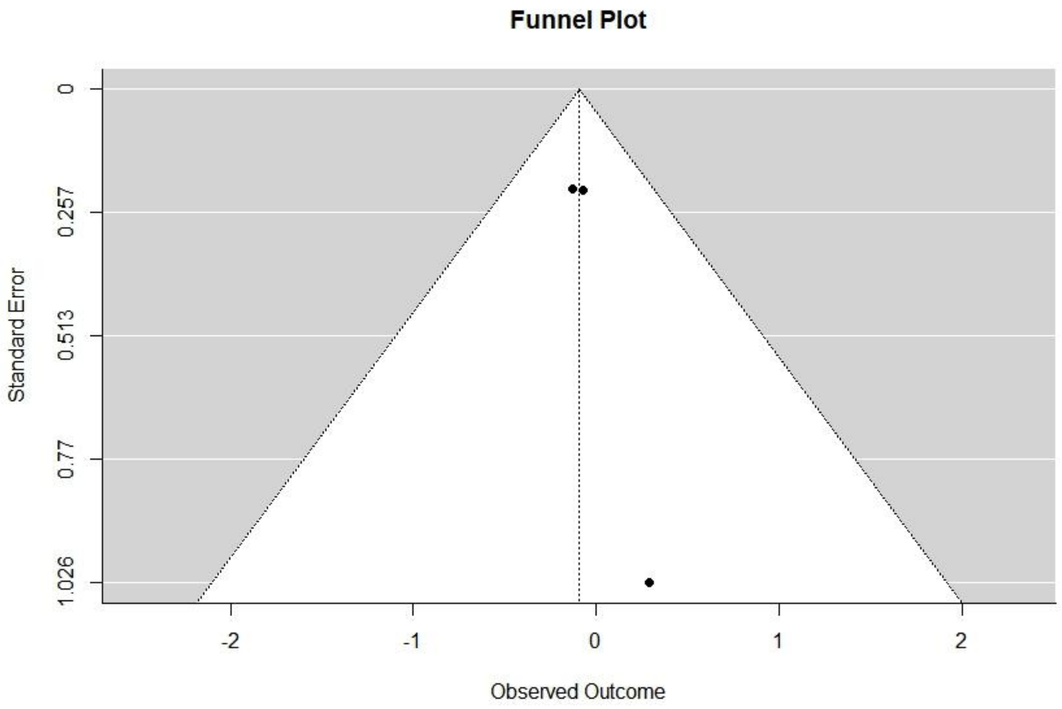

Figure S72 Random-effects meta-analysis of the association between IL-18 rs4988359 polymorphism and AR (forest plot and funnel plot)

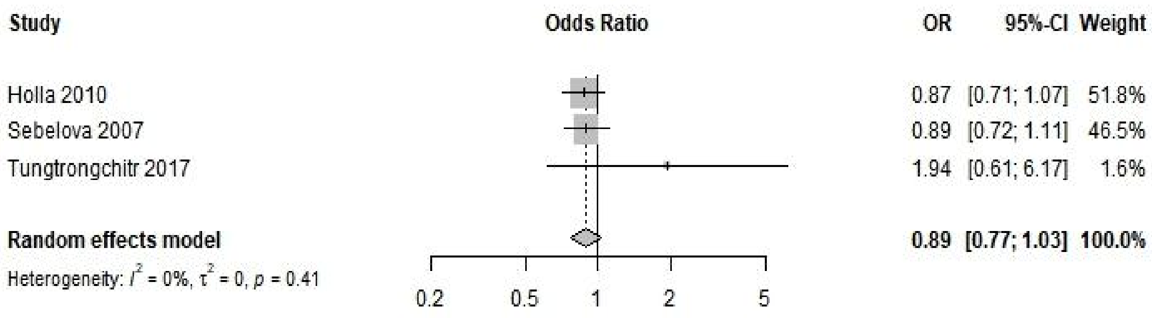

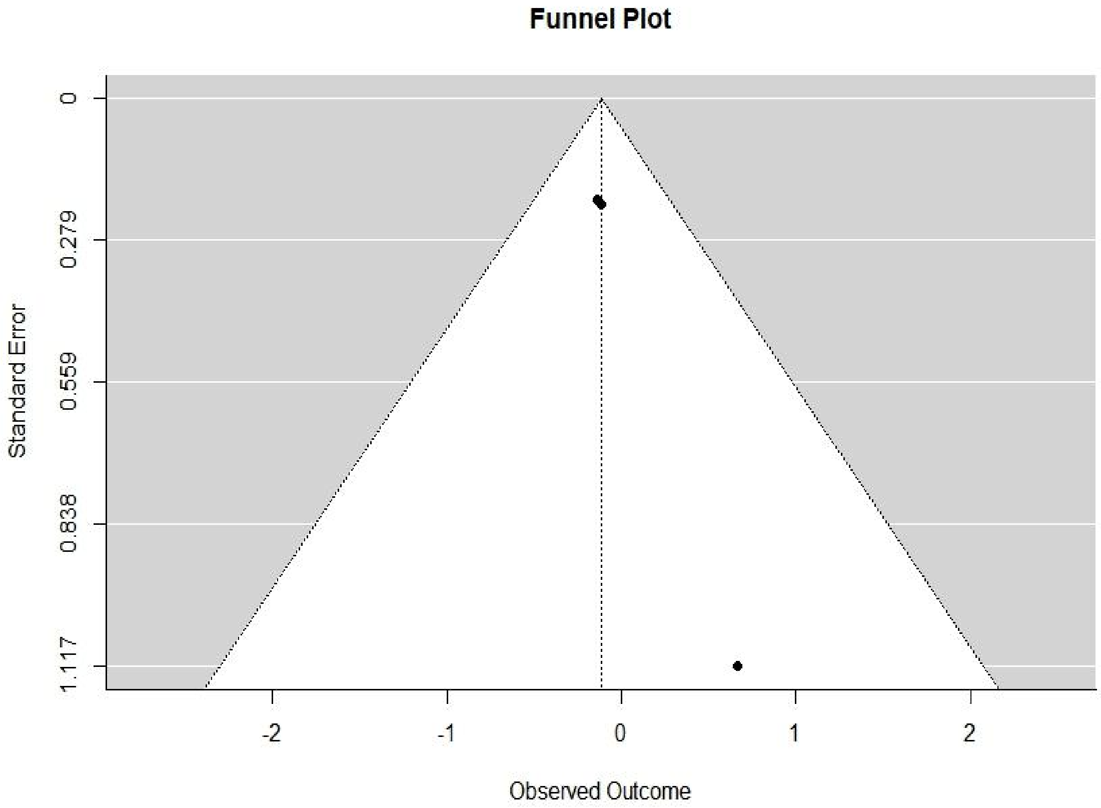

Figure S73 Random-effects meta-analysis of the association between Tim-3 rs10515746 polymorphism and AR (forest plot and funnel plot)

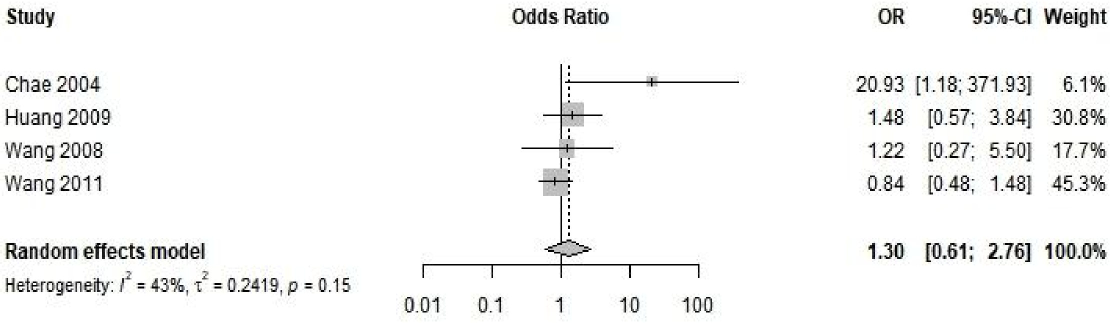

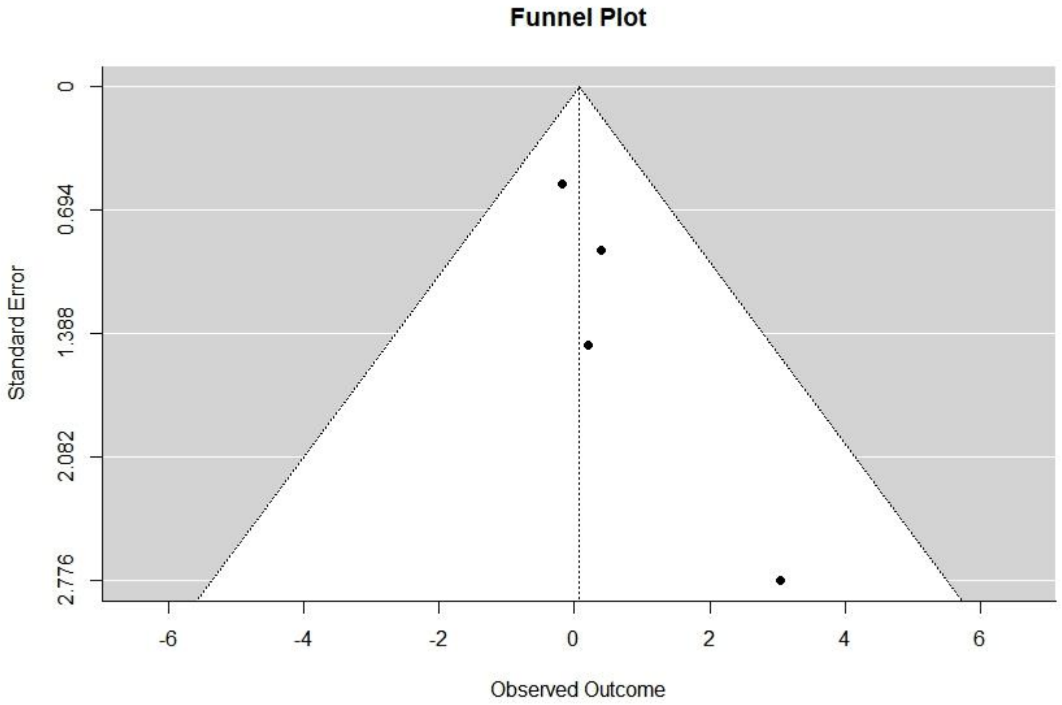

Figure S74 Random-effects meta-analysis of the association between IL-4R rs1805010 polymorphism and AR (forest plot and funnel plot)

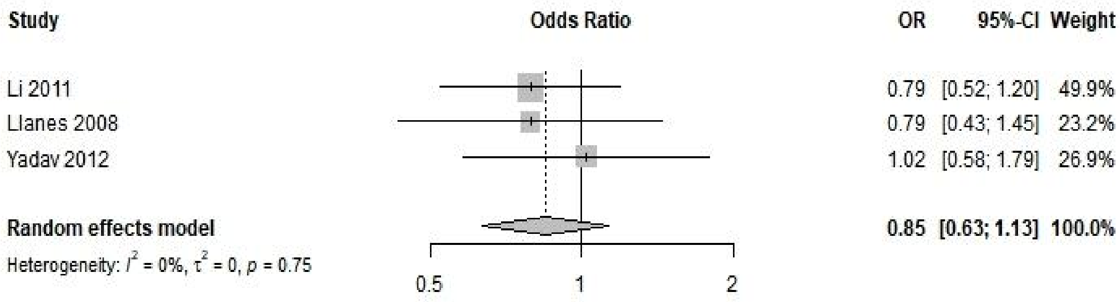

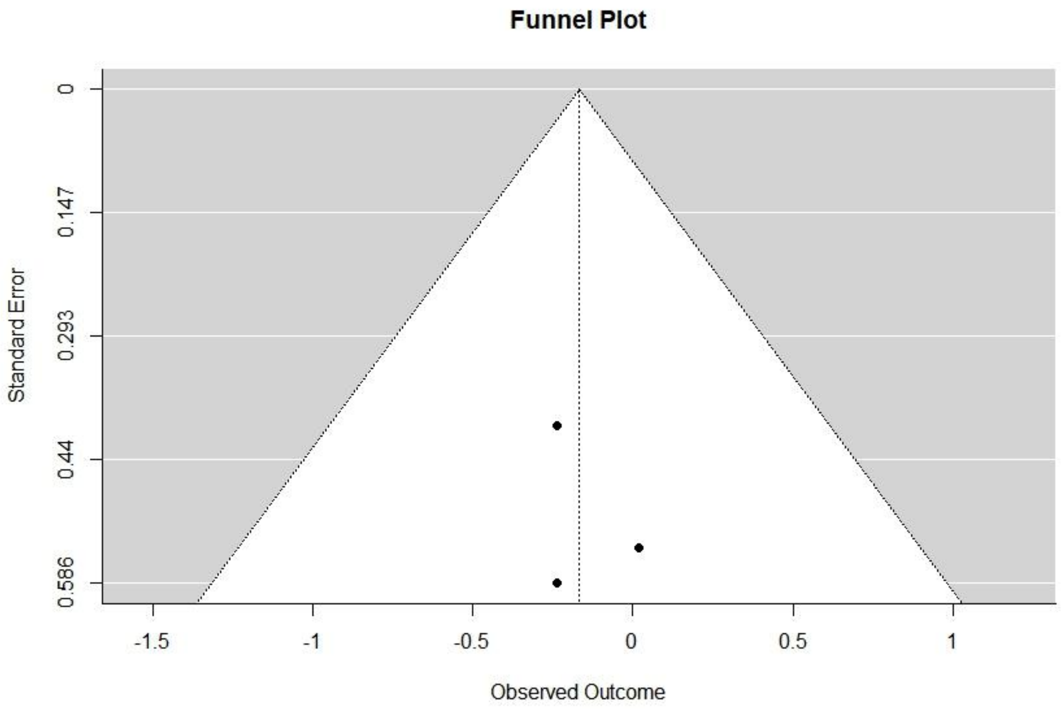

Figure S75 Random-effects meta-analysis of the association between TAP1 333 polymorphism and AR (forest plot and funnel plot)

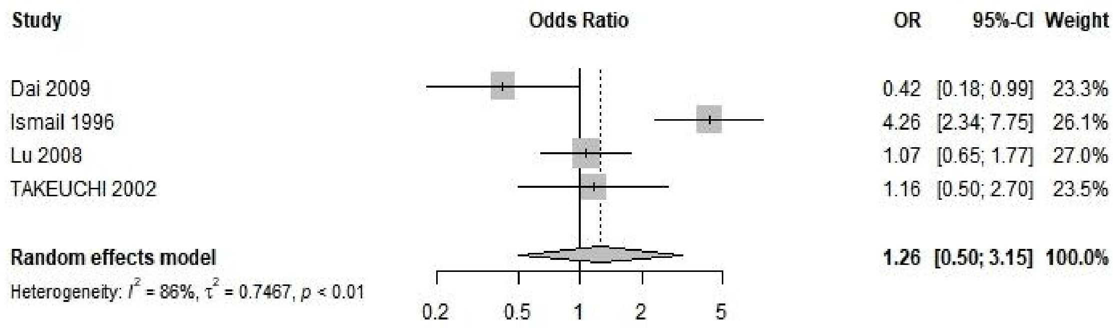

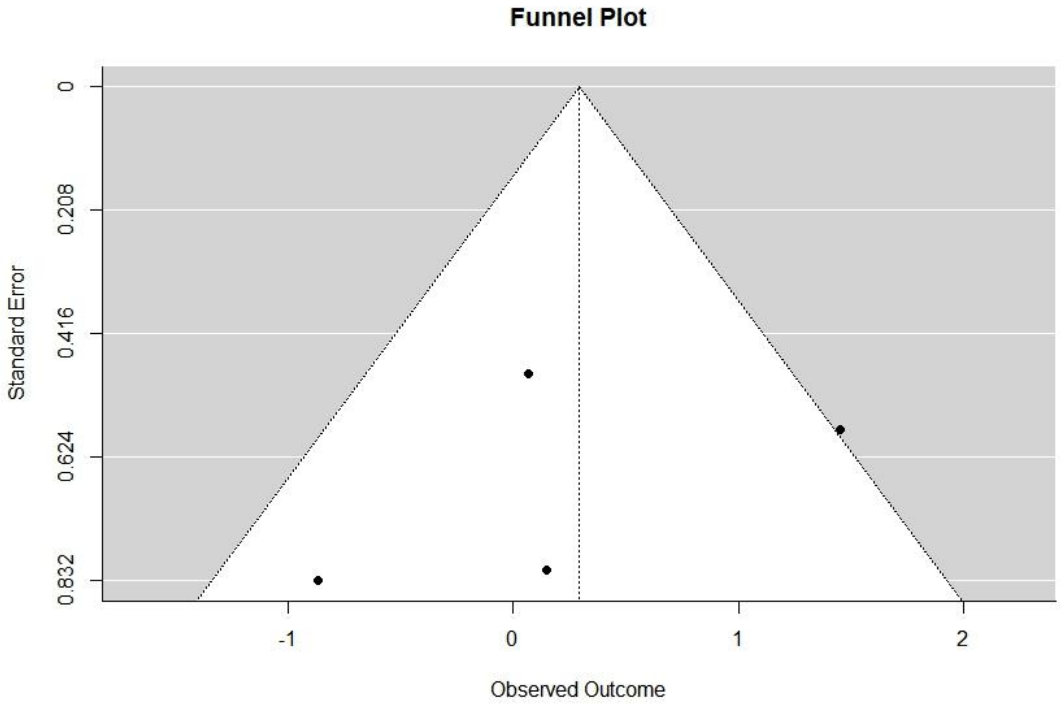

Figure S76 Random-effects meta-analysis of the association between TAP1 637 polymorphism and AR (forest plot and funnel plot)

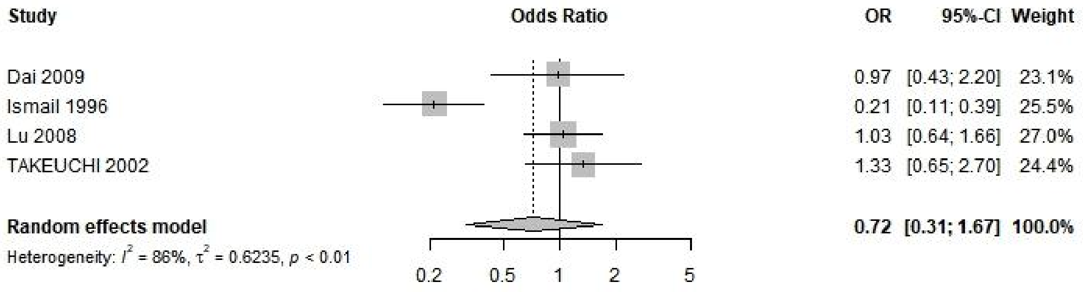

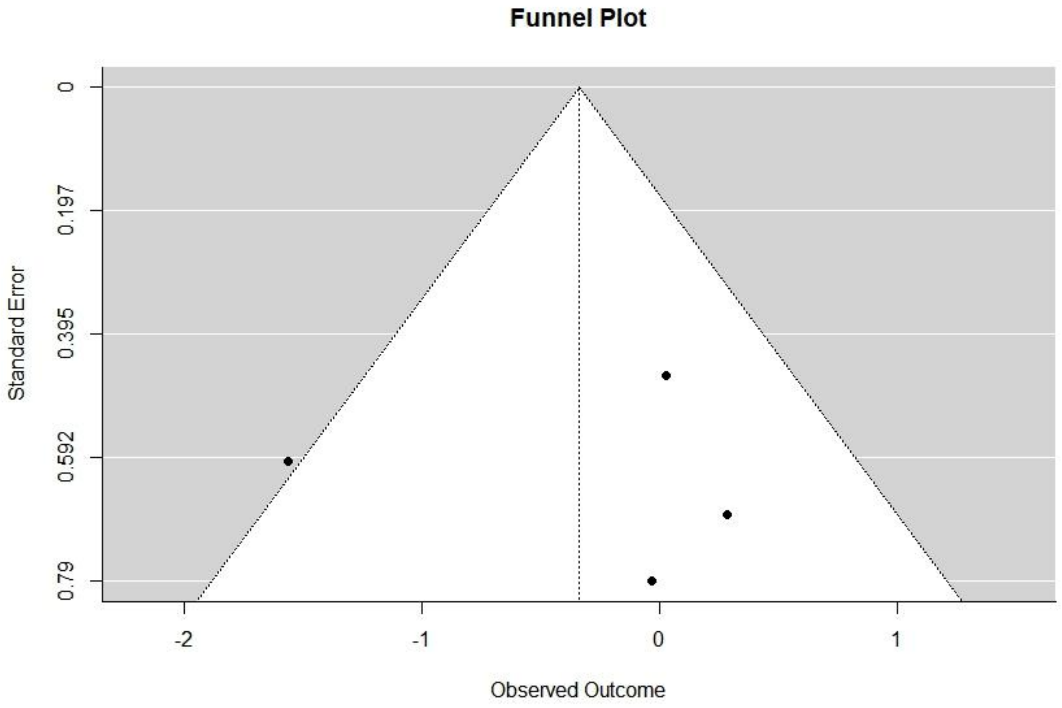

Figure S77 Random-effects meta-analysis of the association between TSL rs1898671 polymorphism and AR (forest plot and funnel plot)

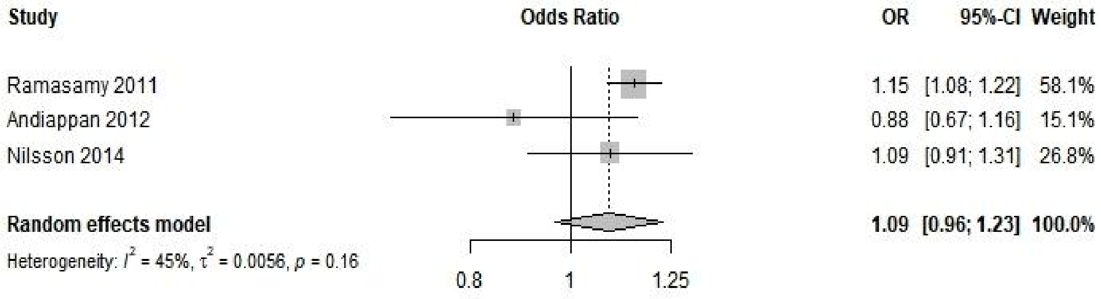

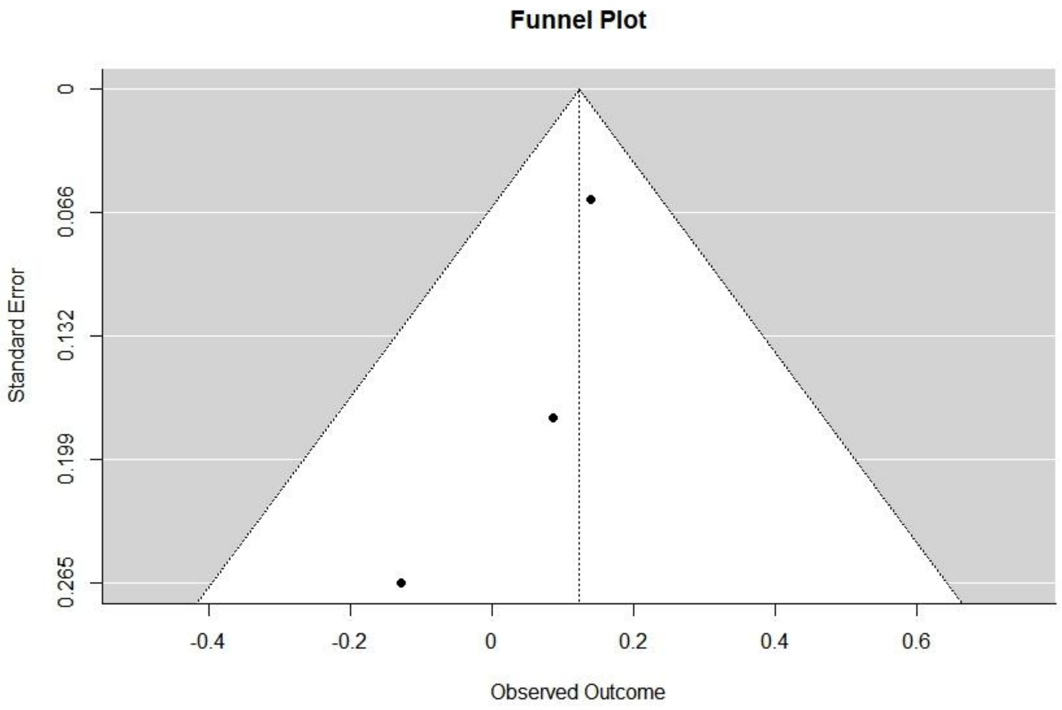

Supplement: Supplementary file 1 — Supplementary file1 (PDF 6487 KB) [file 12016_2023_8964_MOESM1_ESM.pdf]
